# Alkylation of Nitropyridines via Vicarious Nucleophilic Substitution

**DOI:** 10.1021/acs.orglett.1c03920

**Published:** 2022-01-03

**Authors:** Damian Antoniak, Michał Barbasiewicz

**Affiliations:** Faculty of Chemistry, University of Warsaw, Pasteura 1, 02-093 Warsaw, Poland

## Abstract

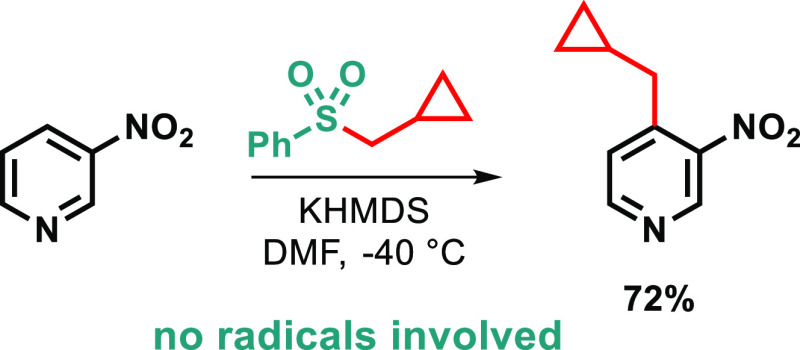

Electrophilic nitropyridines react with sulfonyl-stabilized carbanions to give products of C–H alkylation via vicarious nucleophilic substitution. The process consists of formation of the Meisenheimer-type adduct followed by base-induced β-elimination of the sulfinic acid (e.g., PhSO_2_H). Mechanistic studies reveal that in the latter step alkyl substituent and adjacent nitro group tend to planarize for effective stabilization of benzyl anion, and thus, adduct of hindered isopropyl carbanion remains stable toward elimination for steric reasons.

Alkylation of aromatic compounds is a key synthetic transformation that was discovered over century ago^1^ and has been continuously explored in search for more selective, tolerant, and efficient reagents and conditions.^2^ The most common variant of aromatic substitution, the electrophilic Friedel–Crafts reaction, is known to work well on electron-rich substrates, whereas nitroarenes and azaarenes usually fail to react because of diminished π-electron density and complexation with the catalyst.^3,4^ To address these issues numerous methods based on radical (Minisci),^5^ transition-metal-catalyzed, photochemical,^2^ and electrochemical^6^ mechanisms were developed. At the same time, nucleophilic processes seem to be less common and are often connoted with S_N_Ar reactions on, e.g., halonitroarenes, rather than tools for direct C–H functionalization. However, recent studies have revealed that in such aromatics addition of the nucleophile is fastest at the position occupied by hydrogen and that products of halogen substitution are formed only via equilibration of the initial addition step.^7^ Accordingly, a key difference between electrophilic and nucleophilic aromatic substitution of hydrogen arises from different departure abilities of proton and hydride anion from the initially formed cationic and anionic σ adducts, respectively.^8^ When in *electrophilic* variant the rearomatization step usually runs spontaneously,^4^ attack of *nucleophile* on nitroarene at the position occupied by hydrogen gives Meisenheimer-type adduct.^9^ Two general routes to transform such adducts into the desired substitution products were developed: oxidative rearomatization^10^ and vicarious nucleophilic substitution (VNS), where the nucleophile possesses a leaving group (e.g., halogen; Scheme 1, top).^11^

**Scheme 1:**
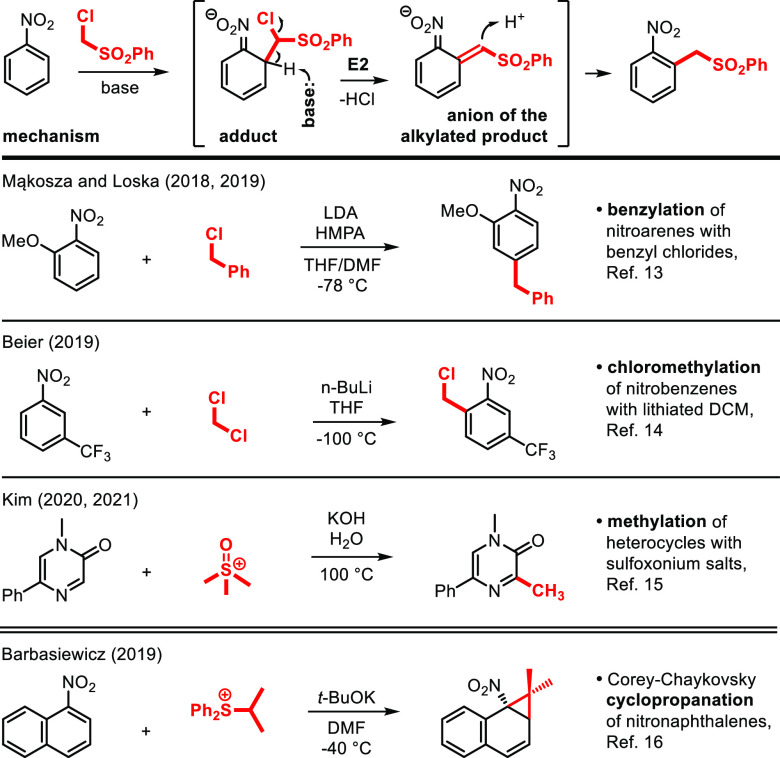
(top) Mechanism of Vicarious Nucleophilic Substitution of Hydrogen and (bottom) Recent Examples of Related Processes

Mechanism of the VNS on a model reaction with chloromethyl sulfone consists of addition of the carbanion to nitroarene followed by base-induced β-elimination of HCl to give anion of the alkylated product.^7,9,12^ Interestingly, in this scenario leaving group is attached at the α-position of the alkylating agent, mimicking substrates for electrophilic Lewis acid-catalyzed reactions. Therefore, the reversed-polarity, base-induced variant of the aromatic substitution is sometimes called *umpoled Friedel–Crafts reaction*.^13^ The methodology, developed by Mąkosza,^11a^ usually works well on carbanion precursors bearing two separate functions: an electron-withdrawing group (EWG) and a leaving group (LG), as α-chloroalkyl sulfones, α-chloro esters, etc. Therefore, products of the transformation possess the EWG substituent at the benzylic position of the newly introduced alkyl group. Only recently a few attempts to extend the methodology have been reported in the literature.^11c^ Mąkosza and Loska^13^ and then Beier^14^ reported introduction of alkyl groups that can partially stabilize the negative charge (i.e., act as an EWG), such as benzyl, chlorobenzyl, and chloromethyl. In turn, Kim and co-workers demonstrated alkylation of quinoline *N*-oxides and pyrazinones with sulfonium and sulfoxonium salts at elevated temperatures.^15^ Interestingly, our own exploration of related Corey–Chaykovsky reagents (in which EWG = LG), such as alkyl phenyl selenones and alkyldiphenyl sulfonium salts, revealed different reactivity in which intermediate adducts spontaneously cyclized (γ-eliminated) to cyclopropanes, giving dearomatized benzonorcaradienes (Scheme 1, bottom).^16^ We reckoned that precursors with worse leaving group ability, such as alkyl sulfones and sulfonates, may suppress the cyclization but still undergo base-induced β-elimination to give alkylated products. In this report we present alkylation of nitropyridines and explain how branching of the carbanion precursor and electrophilicity of the arene determine the process.

In our synthetic studies, we applied reaction conditions reported previously (DMF, −40 °C, 30 min),^16^ using an excess of strong base (KHMDS). After preliminary screening of nitroarenes, we observed that electrophilic 3-nitropyridines react efficiently with alkyl phenyl sulfones **2** and neopentyl alkanesulfonates **3**(17) to give alkylated products **4a**–**t** (Scheme 2). Under these conditions, products with various alkyls, such as Me, Et, iBu, Oct, neopentyl, etc., were isolated as single or predominant isomers, whereas minor isomers, formed in a few cases, contained alkyls attached at different positions of the aromatic ring (shown with red arrows in Scheme 2). In general, sulfonate precursors **3** gave slightly better yields than sulfones **2**, but their preparation utilized alkanesulfonyl chlorides, which are either commercially available only for selected alkyls or expensive.^18^ Formation of products **4f** and **4o** deserves special attention, as the cyclopropylmethyl motif is often used to study radical reactions because of fast, spontaneous ring opening to give the but-1-en-4-yl system.^19^ Although radicals were excluded as intermediates in VNS reactions,^12^ we repeated preparation of **4f** with 150 mol % TEMPO and obtained essentially the same yield of main isomer of the alkylated pyridine (71% vs 72%).^20^

**Scheme 2:**
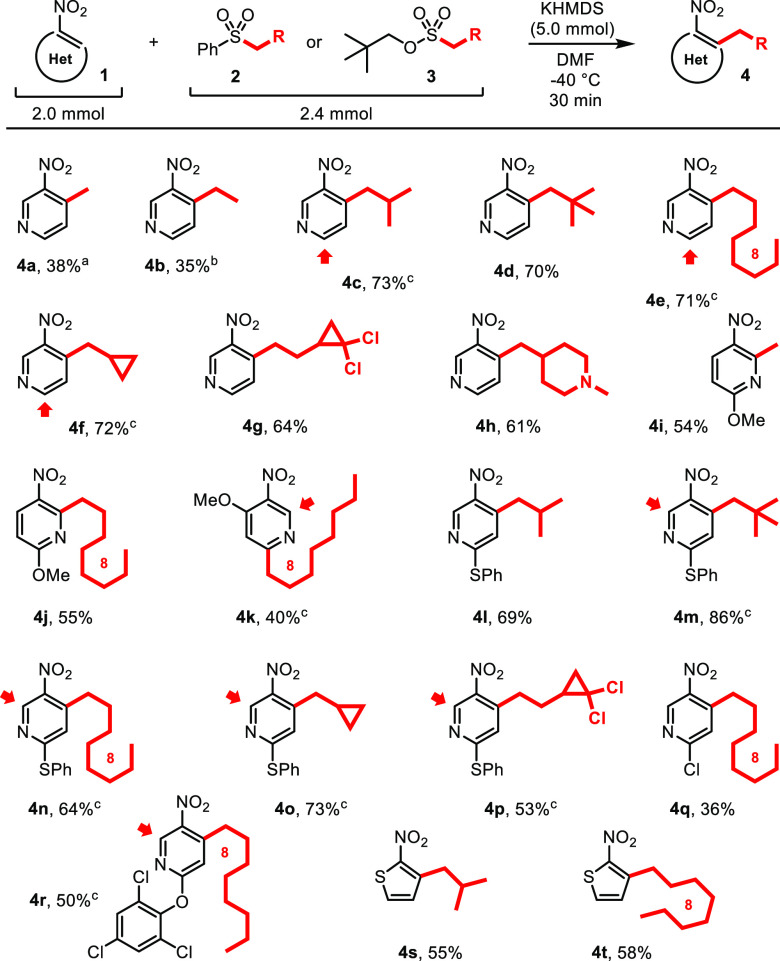
Alkylation of Nitroarenes **1** via Vicarious Nucleophilic Substitution with Sulfones **2** and Sulfonates **3** The reaction was carried out at −60 °C for 3 min. The reaction was carried out for 3 min. Minor isomers of the alkylated products were isolated in a few cases (the positions of substitution are shown with red arrows): **4c′**, 9%; **4e′**, 9%; **4f′**, 9%; **4k′**, 14%; **4m′**, 5%; **4n′**, 6%; **4o′**, 8%; **4p′**, 9%; **4r′**, 26%.

The scope and limitation studies also revealed other consequences of the reaction mechanism. Whereas methyl and primary alkyl groups were easily introduced into the substrates, reaction of secondary carbanion of isopropyl phenyl sulfone with 3-nitropyridine failed to give even traces of the alkylated product; instead, N-protonated Meisenheimer-type adduct **5a** was isolated in 43% yield and characterized with X-ray studies (Scheme 3, middle).

**Scheme 3:**
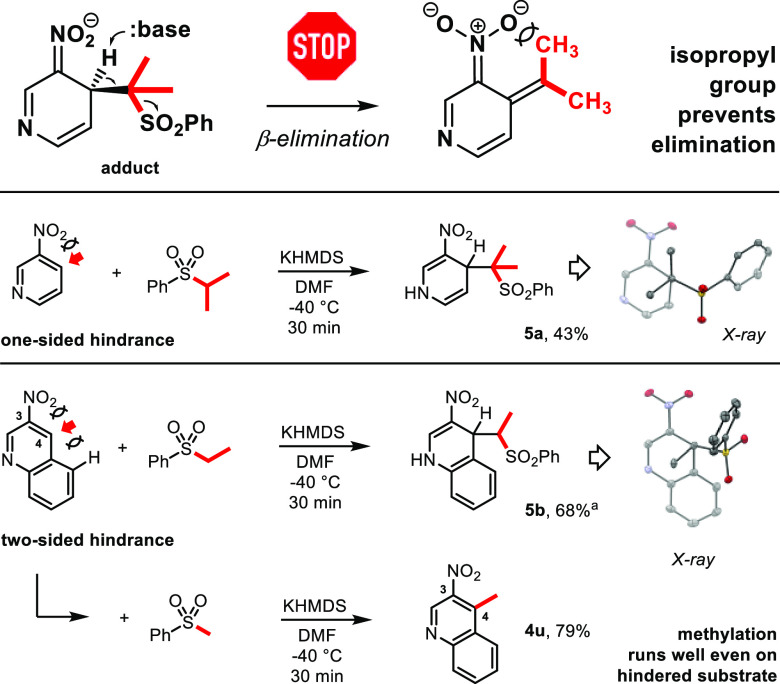
Mechanistic Studies: Effect of Sulfone Carbanion Branching and Nitroarene Hindrance on the Reaction Course Traces of the alkylated product (4-ethyl-3-nitroquinoline) were formed.

The intriguing observation suggested that although addition of the secondary carbanion runs unperturbed, steric hindrance develops in the course of the elimination step. Indeed, consideration of structure of the expected alkylation product *anion* revealed that negative charge at the benzylic position can be stabilized exclusively by resonance with the aromatic ring, and thus, both alkyl substituent and adjacent nitro group must be coplanar with the ring for effective orbital overlap (Scheme 3, top). As the isopropyl group is symmetrical, one of its methyls always collides with adjacent oxygen atom of the NO_2_ (“one-sided hindrance” of 3-nitropyridine; Scheme 3, middle), which stops second step of the reaction. Demand for planarization of the product anion was further supported by reactivity of 3-nitroquinoline, in which electrophilic 4-position is hindered on *both sides* by NO_2_ and distant aromatic ring (“two-sided hindrance”; Scheme 3, bottom). In this case, also primary carbanion precursor, ethyl phenyl sulfone, gave predominantly N-protonated adduct **5b** and only traces of the alkylated product. Despite the presence of only one methyl at the carbanionic center, elimination step was virtually impossible, independent of which geometrical isomer of the product anion would be produced. In turn, the same nitroarene methylation ran in high yield (79% yield of **4u**), as expected for small steric demands of exocyclic methylene group (=CH_2_ vs =C(CH_3_)_2_; cf. Scheme 3, top). The result was also consistent with our previous observations on related reactions of nitronaphthalenes, in which isopropyl diphenyl sulfonium salt gave benzonorcaradienes in excellent yields (Scheme 1, bottom) without contaminant formation of the alkylated products (as was the case for, e.g., ethyl precursor).^16^ Evidently, the postulated demand for planarization appears exclusively in the course of β-elimination, whereas cyclization to nonplanar, dearomatized benzonorcaradienes remains unconstrained.

Successful synthetic data (Scheme 2) and mechanistic understanding (Scheme 3) inspired us to apply the method to less electrophilic nitroarenes.^21,22^ When we repeated reaction of neopentyl *octane*sulfonate with 4-chloro-1-nitrobenzene, octylated product (**4v**, 15%) and its dimer **6a** (39%) were isolated (Scheme 4, top).^23^ Moreover, analogous dimer **6b** was synthesized by reaction of neopentyl *ethane*sulfonate, and its structure was confirmed by X-ray studies.^20^ We reasoned that the dimers are formed in a secondary process from the alkylated product anions (which are likely less stabilized than nitropyridine derivatives). Indeed, when we subjected **4v** to standard basic conditions, a clean transformation to **6a** was observed in 66% conversion according to ^1^H NMR analysis. Importantly, similar coupling (dimerization) of anions of 4-alkyl-3-nitropyridines was observed practically only with the least-hindered methyl derivative, **4a**.^20,23^ It is clear from this that the alkylation process is complicated by side reactions of the product anions lacking the EWG substituent (e.g., PhSO_2_; cf. Scheme 1, top), and thus, combined stabilization by nitroarene ring (electronic) and substitution at the benzyl position (steric, R≠H) is required to obtain high yields of the alkylated products.^22^

**Scheme 4:**
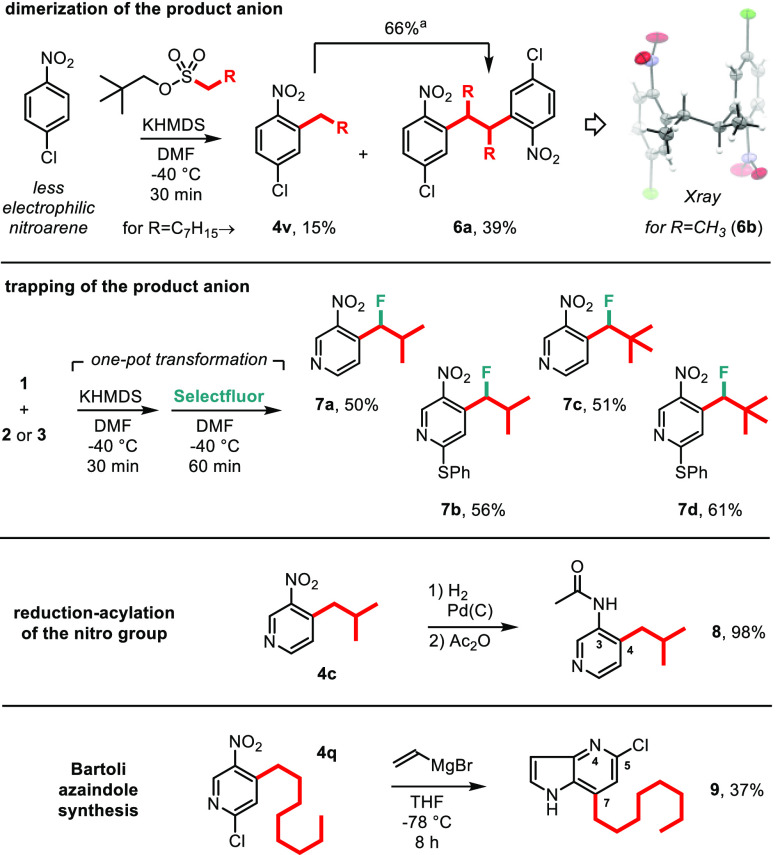
Follow-Up Studies: Transformations of Alkylated Nitroarenes in (top) Anionic and (bottom) Neutral Forms Conversion of **4v** to **6a** under standard reaction conditions (KHMDS, DMF, −40 °C, 30 min) according to ^1^H NMR analysis.

Increased stability of anions of the alkylated nitropyridines **4** prompted us to attempt their quench with an external electrophile such as Selectfluor (Scheme 4, middle).^24^ By means of the one-pot alkylation–fluorination procedure, benzyl fluorides **7a**–**d** were produced in 50–61% yield. In the last part of our studies, we tested also postsynthetic transformations of selected pyridines. 3-Acetamido-4-isobutylpyridine (**8**) was obtained in 98% yield from reduction–acylation of the nitro group in **4c**, and 5-chloro-7-octyl-4-azaindole (**9**) was formed from pyridine **4q** in 37% yield under Bartoli conditions (Scheme 4, bottom).^25^

In conclusion, we have presented transition-metal-free alkylation of electrophilic nitropyridines, which expands methodology of vicarious nucleophilic substitution. The reaction runs via addition of the carbanion in the vicinity of the nitro group followed by base-induced β-elimination, which demands planarization of produced benzyl anion. In effect, for selected combinations of substrates steric hindrance may inhibit the latter step, and then only protonated Meisenheimer-type adduct is isolated. Moreover, anions of the alkylated products can be quenched with Selectfluor, giving substituted benzyl fluorides. The presented transformations are of particular meaning because of the remarkable therapeutic potential of pyridine derivatives and their application as agrochemicals, building blocks, ligands, and functional materials.^26^

## Supporting Information Available

Experimental procedures, characterization data, and reproductions of NMR spectra of the synthesized compounds (PDF)

## Accession Codes

CCDC 2120752–2120754 contain the supplementary crystallographic data for this paper. These data can be obtained free of charge via www.ccdc.cam.ac.uk/data_request/cif, or by emailing data_request@ccdc.cam.ac.uk, or by contacting The Cambridge Crystallographic Data Centre, 12 Union Road, Cambridge CB2 1EZ, U.K.; fax: +44 1223 336033.
