# Supplementary material for: Alkylation of Nitropyridines via Vicarious Nucleophilic Substitution
Source: Org Lett. 2022 Jan 3;24(2):516–9. doi: 10.1021/acs.orglett.1c03920 (PMC8787753; doi:10.1021/acs.orglett.1c03920)

Supporting Information

Alkylation of Nitropyridines via Vicarious Nucleophilic Substitution

Damian Antoniak[1] and Michał Barbasiewicz[1,*]

[1] Faculty of Chemistry, University of Warsaw
Pasteura 1, 02-093 Warsaw, Poland

*barbasiewicz@chem.uw.edu.pl
www.aromaticity.pl

Table of content

1. General information 3
2. Syntheses of substrates 4
   2.1. Syntheses of alkyl phenyl sulfones (2a-2i) 4
   2.2. Syntheses of neopentyl alkanesulfonates (3a-3c, 3e) 15
   2.3. Syntheses of nitroarenes (1a-1i) 19
3. Alkylation of nitroarenes 23
   3.1. General Procedure for alkylation of nitroarenes 23
   3.2. Preparative alkylation of nitroarenes 24
   3.3. Informations concerning isolation of nitroarenes 25
   3.4. Characterization of the alkylated products 26
   3.5. Characterization of protonated σ^H-adducts 43
   3.6. Characterization of side products 45
4. Further studies 53
   4.1. Stability of the alkylated products anions 53
   4.2. Experiment with radical scavenger (TEMPO) 54
5. One-pot alkylation-fluorination of nitroarenes 55
6. Postsynthetic transformations of the alkylated pyridines 58
   6.1. Reduction of the NO_2 group with subsequent acylation 58
   6.2. Synthesis of azaindole under Bartoli conditions 59
7. References 60
8. Reproductions of NMR spectra 61

1. General information

Methyl iodide, isopropyl bromide, and 4-chloronitrobenzene were purchased from Acros Organics.

Diethyl azodicarboxylate and tert-butyl hydroperoxide (70% solution in H_2O) were purchased from Alfa Aesar.

1-Methyl-4-piperidinemethanol, 2-chloro-5-nitropyridine, 4-methoxy-3-nitropyridine, and TEMPO were purchased from AmBeed.

K_2CO_3, KOH, Na_2CO_3, Na_2SO_3, $MgSO_4$, NH_4Cl, acetic acid, and acetic anhydride were purchased from Chempur, Poland.

Methanesulfonyl chloride and Pd/C were purchased from Fluka.

LiCl, cyclopropylmethyl bromide, octanesulfonyl chloride, and 2-nitrothiophene were purchased from Fluorochem.

Triethylamine, isobutyl iodide, NaCl, and NaOH were purchased from POCh, Poland.

Thiophenol, neopentyl alcohol, ethyl bromide, ethanesulfonyl chloride, isobutanesulfonyl chloride, 3-aminoquinoline, tetrabutylammonium chloride, benzyltriethylammonium chloride, NaH (60% in oil), sodium benzenesulfinate, 4-bromobut-1-ene, octyl bromide, triphenylphosphine, 3-nitropyridine, 2,4,6-trichlorophenol, 2-bromo-5-nitropyridine, vinylmagnesium bromide (1.0 M in THF), $CHCl_3$, MeOH, dry DMF, dry THF and dry DMSO were purchased from Sigma-Aldrich.

KHMDS (solution in THF) was purchased from Sigma-Aldrich and Fluorochem (1 M from Sigma-Aldrich and 20% w/w from Fluorochem).

Hydrogen peroxide (30% in H_2O) and KI were purchased from Stanlab, Poland.

Selectfluor was purchased from Apollo.

Commercially available solvents and materials were used without further purification.

Column chromatography was performed on silica gel (high-purity grade, pore size 60 Å, 230–400 mesh particle size, 40–63 μm).

Thin layer chromatography (TLC) was performed on Supelco Silica gel 60 F_{254} aluminium sheets and was visualized under UV lamp. In some cases, solution of $KMnO_4$ was used for visualization (prepared form 0.6 g $KMnO_4$, 4 g K_2CO_3, 5 mg NaOH and 80 mL H_2O).

Analytical GLC was performed on a PerkinElmer Clarus 580 chromatograph equipped with a flame ionization detector, and a GL Sciences InertCap 5MS/Sil column with He as a carrier gas (column 0.25 mm × 30 m, carrier flow 1.5 mL/min, method parameters 50 °C, +10 °C/min to 300 °C, then 15 min at 300 °C).

^{1}H, ^{19}F, and ^{13}C NMR spectra were recorded on Agilent 400 MHz NMR spectrometer. Chemical shifts (δ) are given in parts per million (ppm) with solvent resonance as internal standard (for $CDCl_3$: 7.24 and 77.0 ppm, for ^{1}H and ^{13}C NMR, respectively; for DMSO-d_6: 2.50 and 40.0 ppm, for ^{1}H and ^{13}C NMR, respectively) or with $CFCl_3$ (0.0 ppm for ^{19}F NMR). Spin multiplicity was abbreviated as follows: s, singlet; d, doublet; t, triplet; q, quartet; hept, heptet; m, multiplet; br s, broad singlet.

Melting points were uncorrected.

2. Syntheses of substrates

2.1. Syntheses of alkyl phenyl sulfones (2a-2i)

Syntheses of alkyl phenyl sulfones (**2a-2i**) were described below:

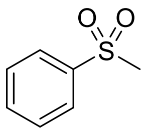

2a

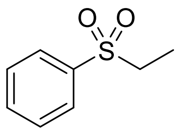

2b

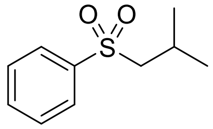

2c

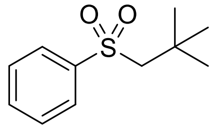

2d

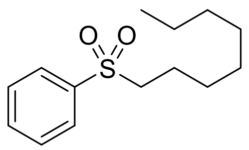

2e

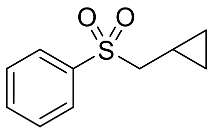

2f

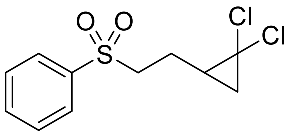

2g

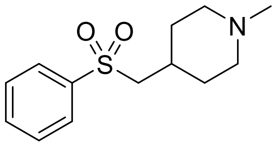

2h

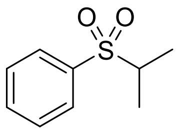

2i

Synthesis of methyl phenyl sulfone (2a)

Methyl phenyl sulfone was prepared according to the reported procedure.[1]

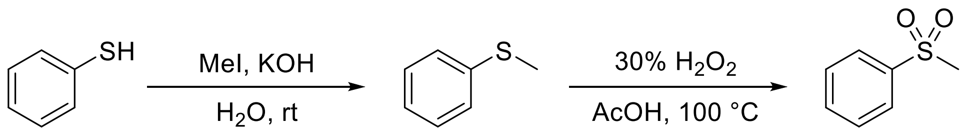

2a, 82%
over 2 steps

A 100 mL round-bottom flask was charged with thiophenol (5.50 g, 49.9 mmol, 1.00 equiv.) and immersed in water bath (rt). To the stirred reaction mixture solution of KOH (4.22 g, 75.2 mmol, 1.51 equiv.) in H_2O (35 mL) was added portionwise, then methyl iodide (7.75 g, 54.6 mmol, 1.09 equiv.) was added dropwise. After 2 h reaction mixture was extracted with ethyl acetate (3 × 50 mL), combined organic phases were washed with H_2O (50 mL), brine (50 mL), and dried over anhydrous $MgSO_4$. The mixture was filtered and evaporated. Crude methyl phenyl sulfide was placed in 100 mL round-bottom flask, acetic acid (20 mL) was added, to the stirred reaction mixture $H_2O_{2\ aq}$ (22 mL, 216 mmol, 30% v/v, 4.33 equiv.) was added dropwise, and reaction was heated to ca. 100 °C (oil bath). After 3 h reaction mixture was poured onto water/ice (300 mL), solid Na_2CO_3 was added to pH = 7, mixture was extracted with ethyl acetate (3 × 50 mL), combined organic phases were washed with $Na_2SO_{3\ aq}$ (50 mL, 10% w/v), brine (50 mL), and dried over anhydrous $MgSO_4$. Mixture was filtered, evaporated, and crude product was recrystallized form ethanol yielding **2a** (6.63 g, 41.1 mmol, **82%** over 2 steps) as white crystals.

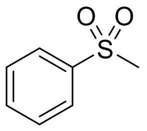

methyl phenyl sulfone, **2a**

white crystals, **mp** 86.5 – 88.0 °C

^{1}H NMR (400 MHz, CDCl$_3$): δ = 7.95 – 7.91 (m, 2H), 7.67 – 7.62 (m, 1H), 7.58 – 7.53 (m, 2H), 3.04 (s, 3H).

^{13}C NMR (100 MHz, CDCl$_3$): δ = 140.2, 133.4, 129.1, 126.9, 44.1.

^{1}H NMR, ^{13}C NMR spectra and mp were consistent with those reported in the literature.[2]

Synthesis of ethyl phenyl sulfone (2b)

Ethyl phenyl sulfone was prepared according to the reported procedure.[1]

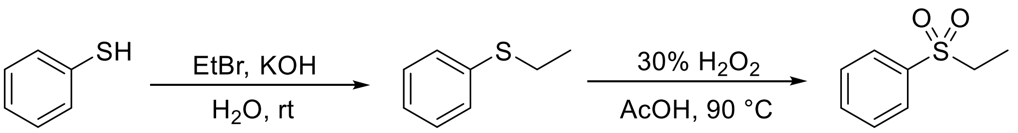

2b, 79%
over 2 steps

A 100 mL round-bottom flask was charged with thiophenol (5.50 g, 49.9 mmol, 1.00 equiv.) and immersed in water bath (rt). To the stirred reaction mixture solution of KOH (4.21 g, 75.1 mmol, 1.51 equiv.) in H_2O (35 mL) was added portionwise, and then ethyl bromide (6.00 g, 54.9 mmol, 1.10 equiv.) was added dropwise. After 2 h mixture was extracted with ethyl acetate (3 × 50 mL), combined organic phases were washed with H_2O (50 mL), brine (50 mL), and dried over anhydrous $MgSO_4$. The mixture was filtered and evaporated. Crude ethyl phenyl sulfide was placed in 100 mL round-bottom flask, acetic acid (20 mL) was added, to the stirred reaction mixture $H_2O_{2\,aq}$ (16 mL, 157 mmol, 30% v/v, 3.15 equiv.) was added dropwise, and reaction mixture was heated to ca. 90 °C (oil bath). After 3 h reaction mixture was poured onto water/ice (300 mL), solid Na_2CO_3 was added to pH = 7, mixture was extracted with ethyl acetate (3 × 50 mL), combined organic phases were washed with $Na_2SO_{3\,aq}$ (50 mL, 10% w/v), brine (50 mL), and dried over anhydrous $MgSO_4$. Mixture was filtered, evaporated, and crude product was recrystallized from ethanol yielding **2b** (6.71 g, 39.4 mmol, **79%** over 2 steps) as white crystals.

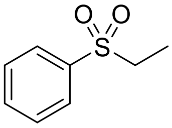

ethyl phenyl sulfone, **2b**

white crystals, **mp** 41.0 – 42.5 °C

^{1}H NMR (400 MHz, $CDCl_3$): δ = 7.92 – 7.86 (m, 2H), 7.67 – 7.60 (m, 1H), 7.58 – 7.46 (m, 2H), 3.09 (q, *J* = 7.4 Hz, 2H), 1.25 (t, *J* = 7.4 Hz, 3H).

^{13}C NMR (100 MHz, $CDCl_3$): δ = 138.3, 133.5, 129.1, 128.0, 50.4, 7.3.

^{1}H NMR, ^{13}C NMR spectra were consistent with those reported in the literature.[1]

Synthesis of isobutyl phenyl sulfone (2c)

Isobutyl phenyl sulfone was prepared according to the reported procedure,[1] modified by addition of phase transfer catalyst at the alkylation step.

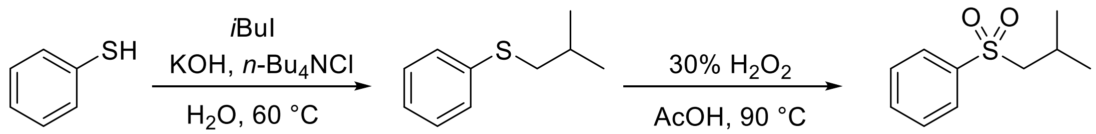

2c, 74%
over 2 steps

A 250 mL round-bottom flask was charged with thiophenol (11.3 g, 103 mmol, 1.00 equiv.) and immersed in water bath (rt). To vigorously stirred reaction mixture solution of KOH (8.63 g, 154 mmol, 1.50 equiv.) in H_2O (70 mL) was added portionwise, then isobutyl iodide (27.2 g, 148 mmol, 1.44 equiv.) was added dropwise. Tetrabutylammonium chloride (0.28 g, 1.01 mmol, 1.0 mol%) was added, and reaction mixture was heated to 60 °C (oil bath). After 16 h reaction mixture was extracted with ethyl acetate (3 × 50 mL), combined organic phases were washed with H_2O (50 mL), brine (50 mL), and dried over anhydrous $MgSO_4$. The mixture was filtered and evaporated. Crude isobutyl phenyl sulfide was placed in 250 mL round-bottom flask, acetic acid (40 mL) was added, to the stirred reaction mixture $H_2O_{2\,aq}$ (32 mL, 314 mmol, 30% v/v, 3.05 equiv.) was added dropwise, and reaction mixture was heated to ca. 90 °C (oil bath). After 2.5 h reaction mixture was poured onto water/ice (400 mL), solid Na_2CO_3 was added to pH = 7, mixture was extracted with ethyl acetate (3 × 100 mL), combined organic phases were washed with $Na_2SO_{3\,aq}$ (100 mL, 10% w/v), brine (100 mL), and dried over anhydrous $MgSO_4$. The mixture was filtered, evaporated, and product was separated by distillation (114-118 °C, 4×10^{-2} mbar) yielding **2c** (15.1 g, 76.0 mmol, **74%** over 2 steps) as a light orange oil.

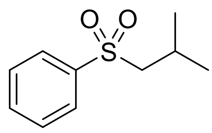

isobutyl phenyl sulfone, **2c**

light orange oil

^{1}H NMR (400 MHz, $CDCl_3$): δ = 7.91 – 7.81 (m, 2H), 7.64 – 7.58 (m, 1H), 7.56 – 7.48 (m, 2H), 2.95 (d, *J* = 6.4 Hz, 2H), 2.26 – 2.11 (m, 1H), 1.01 (d, *J* = 6.8 Hz, 6H).

^{13}C NMR (100 MHz, $CDCl_3$): δ = 140.0, 133.5, 129.2, 127.7, 63.8, 24.0, 22.6.

^{1}H NMR, ^{13}C NMR spectra were consistent with those reported in the literature.[3]

Synthesis of neopentyl phenyl sulfone (2d)

Neopentyl phenyl sulfone was prepared according to modified procedure reported in the literature.[4]

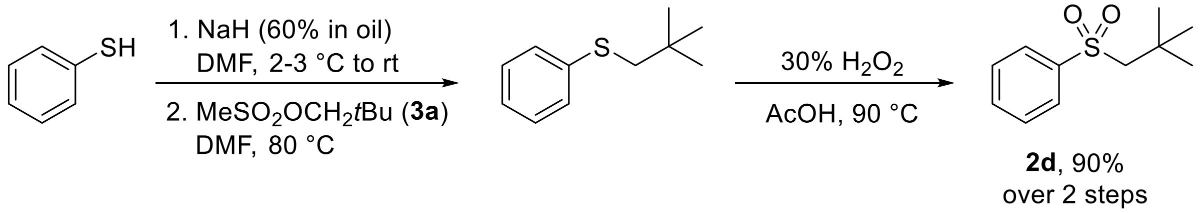

A 100 mL round-bottom flask was charged with thiophenol (3.71 g, 33.6 mmol, 1.12 equiv.) and flushed with argon. Thiophenol was dissolved in dry DMF (20 mL), and flask was immersed in ice/water bath (2-3 °C). To the stirred reaction mixture NaH (1.37 g, 34.3 mmol, 60% in mineral oil, 1.14 equiv.) was added portionwise. After 15 min cooling bath was removed and mixture was stirred for 45 min at rt. Then solution of neopentyl methanesulfonate (**3a**) (4.99 g, 30.0 mmol, 1.00 equiv.) in dry DMF (10 mL) was added, and reaction mixture was stirred at 80 °C (oil bath). After 20 h mixture was transferred to separatory funnel, NH_4Cl_{aq} (50 mL, 10% w/v) was added, mixture was extracted with ethyl acetate (3 × 50 mL), combined organic phases were washed with $LiCl_{aq}$ (50 mL, 1.0 M), $Na_2CO_{3\,aq}$ (50 mL, 10% w/v), brine (50 mL), and dried over anhydrous $MgSO_4$. The mixture was filtered and evaporated. Crude neopentyl phenyl sulfide was placed in 100 mL round-bottom flask, acetic acid (12 mL) was added, to the stirred reaction mixture $H_2O_{2\,aq}$ (7.0 mL, 68.6 mmol, 30% v/v, 2.29 equiv.) was added, and reaction mixture was heated to 90 °C (oil bath). After 2 h reaction mixture was poured onto water/ice (200 mL), solid Na_2CO_3 (11.3 g) was added, mixture was extracted with ethyl acetate (3 × 100 mL), combined organic phases were washed with $Na_2SO_{3\,aq}$ (100 mL, 10% w/v), brine (100 mL), and dried over anhydrous $MgSO_4$. The mixture was filtered, evaporated, and separated with column chromatography (eluent: cyclohexane/ethyl acetate 20:1 to 5:1) yielding **2d** (5.73 g, 27.0 mmol, **90%** over 2 steps) as a white solid.

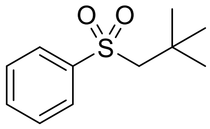

neopentyl phenyl sulfone, **2d**

white solid, **mp** 39.0 – 40.0 °C
eluent: cyclohexane/ethyl acetate 20:1 to 5:1

^{1}H NMR (400 MHz, $CDCl_3$): δ = 7.89 – 7.83 (m, 2H), 7.60 – 7.54 (m, 1H), 7.53 – 7.46 (m, 2H), 3.00 (s, 2H), 1.14 (s, 9H).

^{13}C NMR (100 MHz, $CDCl_3$): δ = 141.7, 133.2, 129.1, 127.4, 67.5, 32.4, 29.7.

^{1}H NMR spectrum[5] and mp[6] were consistent with those reported in the literature.

Synthesis of octyl phenyl sulfone (2e)

Octyl phenyl sulfone was prepared according to the reported procedure,[1] modified by addition of phase transfer catalyst at the alkylation step.

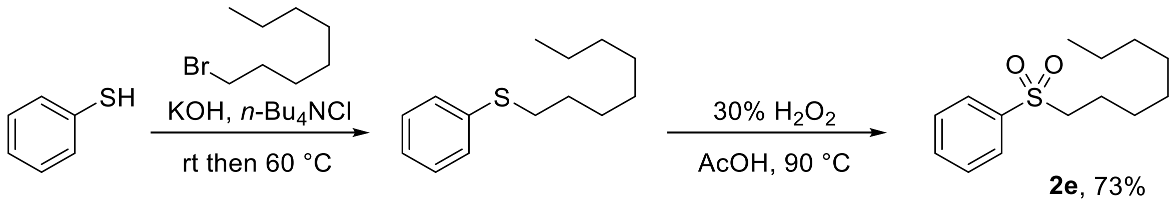

2e, 73%
over 2 steps

A 100 mL round-bottom flask was charged with thiophenol (5.75 g, 52.2 mmol, 1.00 equiv.) and immersed in water bath (rt). To the stirred reaction mixture solution of KOH (4.35 g, 78.3 mmol, 1.50 equiv.) in H_2O (36 mL) was added portionwise, then octyl bromide (11.2 g, 57.9 mmol, 1.11 equiv.) was added dropwise. Tetrabutylammonium chloride (83.0 mg, 1.01 mmol, 1.0 mol%) was added, and reaction mixture was vigorously stirred for 19 h at rt, and for 4h at 60 °C (oil bath). Then reaction mixture was extracted with ethyl acetate (3 × 50 mL), combined organic phases were washed with H_2O (50 mL), brine (50 mL), and dried over anhydrous $MgSO_4$. The mixture was filtered and evaporated. Crude octyl phenyl sulfide was placed in 100 mL round-bottom flask, acetic acid (20 mL) was added, to the stirred reaction mixture $H_2O_{2\ aq}$ (15 mL, 147 mmol, 30% v/v, 2.82 equiv.) was added dropwise, and reaction mixture was heated to ca. 90 °C (oil bath). After 2 h reaction mixture was poured onto water/ice (200 mL), solid Na_2CO_3 was added to pH = 7, mixture was extracted with ethyl acetate (3 × 50 mL), combined organic phases were washed with $Na_2SO_{3\ aq}$ (50 mL, 10% w/v), brine (50 mL), and dried over anhydrous $MgSO_4$. The mixture was filtered, evaporated, and product was dried under high vacuum yielding **2e** (9.69 g, 38.1 mmol, **73%** over 2 steps) as a colorless oil.

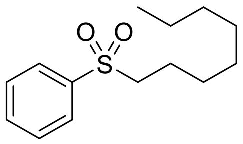

octyl phenyl sulfone, **2e**

colorless oil

^{1}H NMR (400 MHz, $CDCl_3$): δ = 7.90 – 7.85 (m, 2H), 7.65 – 7.60 (m, 1H), 7.56 – 7.51 (m, 2H), 3.08 – 3.01 (m, 2H), 1.71 – 1.62 (m, 2H), 1.36 – 1.14 (m, 10H), 0.82 (t, *J* = 7.1 Hz, 3H).

^{13}C NMR (100 MHz, $CDCl_3$): δ = 139.0, 133.4, 129.0, 127.8, 56.0, 31.4, 28.7, 28.6, 28.0, 22.4, 22.3, 13.8.

^{1}H NMR, ^{13}C NMR spectra were consistent with those reported in the literature.[7]

Synthesis of cyclopropylmethyl phenyl sulfone (2f)

Cyclopropylmethyl phenyl sulfone was prepared from sodium benzenesulfinate and cyclopropylmethyl bromide in DMF at rt.

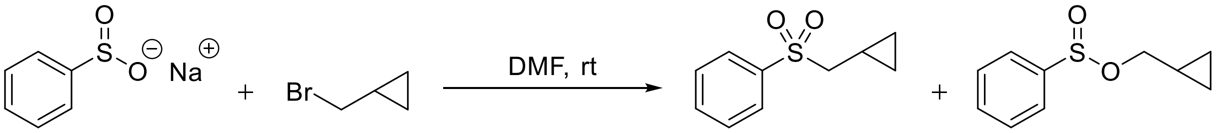

2f, 62% 10%

A 50 mL round-bottom flask was charged with sodium benzenesulfinate (4.11 g, 25.0 mmol, 1.00 equiv.), DMF (15 mL), and immersed in water bath (rt). To the stirred reaction mixture cyclopropylmethyl bromide (4.04 g, 29.9 mmol, 1.20 equiv.) was added dropwise. After 22 h H_2O (50 mL) was added, mixture was extracted with ethyl acetate (3 × 50 mL), combined organic phases were washed with $LiCl_{aq}$ (50 mL, 1.0 M), H_2O (50 mL), brine (50 mL), and dried over anhydrous $MgSO_4$. The mixture was filtered, evaporated and separated with column chromatography (eluent: cyclohexane/ethyl acetate 10:1 to 3:1) yielding **2f** (3.06 g, 15.6 mmol, **62%**, more polar fraction) as a colorless oil, and cyclopropylmethyl benzenesulfinate (0.483 g, 2.46 mmol, **10%**, less polar fraction) as a pale yellow oil.

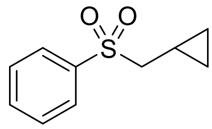

cyclopropylmethyl phenyl sulfone, **2f**

colorless oil
eluent: cyclohexane/ethyl acetate 10:1 to 3:1

^{1}H NMR (400 MHz, $CDCl_3$): δ = 7.83 – 7.78 (m, 2H), 7.56 – 7.51 (m, 1H), 7.47 – 7.41 (m, 2H), 2.91 (d, *J* = 7.2 Hz, 2H), 0.92 – 0.79 (m, 1H), 0.45 – 0.37 (m, 2H), 0.04 – -0.04 (m, 2H).

^{13}C NMR (100 MHz, $CDCl_3$): δ = 138.9, 133.4, 128.9, 128.1, 60.9, 4.5, 4.1.

^{1}H NMR, ^{13}C NMR spectra were consistent with those reported in the literature.[8]

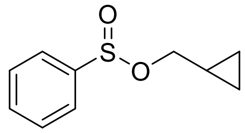

cyclopropylmethyl benzenesulfinate

pale yellow oil
eluent: cyclohexane/ethyl acetate 10:1 to 3:1

^{1}H NMR (400 MHz, $CDCl_3$): δ = 7.66 – 7.60 (m, 2H), 7.48 – 7.41 (m, 3H), 3.76 (dd, *J* = 10.7, 7.4 Hz, 1H), 3.41 (dd, *J* = 10.7, 7.1 Hz, 1H), 1.06 – 0.95 (m, 1H), 0.54 – 0.43 (m, 2H), 0.22 – 0.06 (m, 2H).

Synthesis of 2-(2,2-dichlorocyclopropyl)ethyl phenyl sulfone (2g)

2-(2,2-Dichlorocyclopropyl)ethyl phenyl sulfone was prepared by dichlorocarbene addition to but-3-enyl phenyl sulfone (**2g'**).[9] But-3-enyl phenyl sulfone was prepared from sodium benzenesulfinate and 4-bromobut-1-ene in DMF at rt.

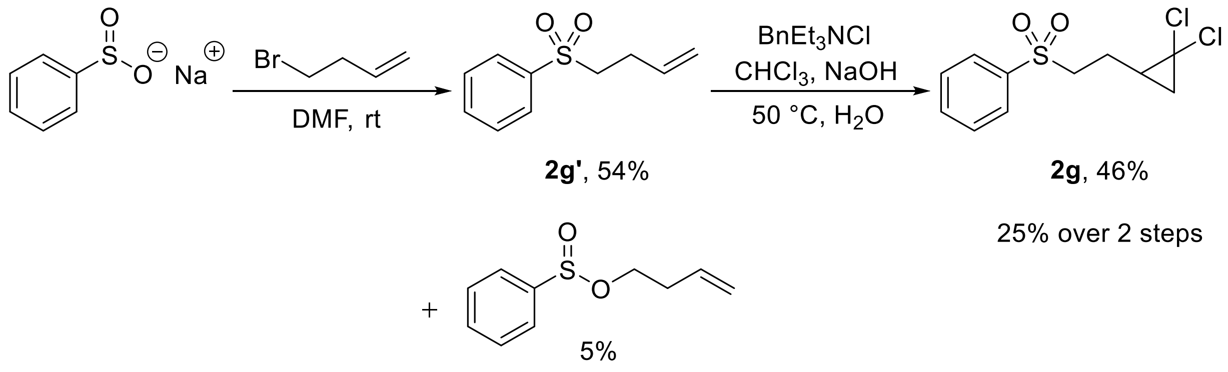

A 50 mL round-bottom flask was charged with sodium benzenesulfinate (8.21 g, 50.0 mmol, 1.00 equiv.), DMF (20 mL), and immersed in water bath (rt). To the stirred reaction mixture 4-bromobut-1-ene (8.11 g, 60.1 mmol, 1.20 equiv.) was added dropwise. After 23 h H_2O (100 mL) was added, mixture was extracted with ethyl acetate (3 × 100 mL), combined organic phases were washed with $LiCl_{aq}$ (100 mL, 1.0 M), $Na_2SO_{3\,aq}$ (100 mL, 10% w/v), brine (100 mL), and dried over anhydrous $MgSO_4$. The mixture was filtered, evaporated and separated with column chromatography (eluent: cyclohexane/ethyl acetate 6:1 to 2:1) yielding **2g'** (5.31 g, 27.1 mmol, **54%**, more polar fraction) as a colorless oil, and but-1-en-4-yl benzenesulfinate (511 mg, 2.60 mmol, **5%**, less polar fraction) as a pale yellow oil.

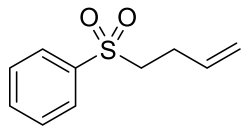

but-1-en-4-yl phenyl sulfone, **2g'**

colorless oil
eluent: cyclohexane/ethyl acetate 6:1 to 2:1

^{1}H NMR (400 MHz, CDCl$_3$): δ = 7.85 – 7.81 (m, 2H), 7.61 – 7.56 (m, 1H), 7.52 – 7.47 (m, 2H), 5.65 (ddt, *J* = 16.8, 10.2, 6.5 Hz, 1H), 5.00 – 4.92 (m, 2H), 3.13 – 3.07 (m, 2H), 2.41 – 2.34 (m, 2H).

^{13}C NMR (100 MHz, CDCl$_3$): δ = 138.7, 133.6, 133.5, 129.1, 127.8, 116.9, 55.0, 26.6.

^{1}H NMR, ^{13}C NMR spectra were consistent with those reported in the literature.[10]

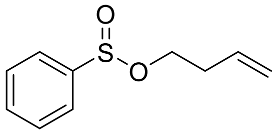

but-1-en-4-yl benzenesulfinate

pale yellow oil
eluent: cyclohexane/ethyl acetate 6:1 to 2:1

^{1}H NMR (400 MHz, CDCl$_3$): δ = 7.72 – 7.67 (m, 2H), 7.55 – 7.48 (m, 3H), 5.71 (ddt, *J* = 17.0, 10.3, 6.7 Hz, 1H), 5.10 – 5.02 (m, 2H), 4.07 (dt, *J* = 9.9, 6.8 Hz, 1H), 3.63 (dt, *J* = 9.9, 6.7 Hz, 1H), 2.37 (qt, *J* = 6.7, 1.4 Hz, 2H).

^{13}C NMR (100 MHz, CDCl$_3$): δ = 144.2, 133.1, 131.7, 128.6, 124.8, 117.1, 63.0, 33.6.

A 50 mL round-bottom flask was charged with **2g’** (2.60 g, 13.3 mmol, 1.00 equiv.), benzyltriethylammonium chloride (65.0 mg, 0.285 mmol, 2 mol%), and $CHCl_3$ (32 mL). To vigorously stirred reaction mixture solution of NaOH (15.9 g, 397 mmol, 29.8 equiv.) in H_2O (16 mL) was added, and mixture was heated to 50 °C (oil bath). After 60 h, mixture was extracted with dichloromethane (3 × 50 mL), combined organic phases were washed with H_2O (2 × 50 mL), brine (50 mL), and dried over anhydrous $MgSO_4$. The mixture was filtered, evaporated, and separated with column chromatography (eluent: cyclohexane/ethyl acetate 10:1 to 1:3) yielding **2g** (1.71 g, 6.13 mmol, **46%**, **25%** over 2 steps) as a pale yellow solid.

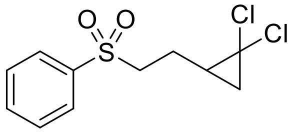

2-(2,2-dichlorocyclopropyl)ethyl phenyl sulfone, **2g**

pale yellow solid, **mp** 63.0 – 64.5 °C
eluent: cyclohexane/ethyl acetate 10:1 to 1:3

^{1}H NMR (400 MHz, CDCl$_3$): δ = 7.93 – 7.88 (m, 2H), 7.68 – 7.62 (m, 1H), 7.61 – 7.52 (m, 2H), 3.35 – 3.17 (m, 2H), 2.11 – 2.00 (m, 1H), 1.90 – 1.79 (m, 1H), 1.69 – 1.55 (m, 2H), 1.12 – 1.06 (m, 1H).

^{13}C NMR (100 MHz, CDCl$_3$): δ = 138.8, 133.9, 129.4, 128.0, 60.2, 54.7, 28.6, 26.7, 24.0.

MS (EI): m/z (%) = 283, 281, 279 (1, 1, 2, $[M+H]^+$), 282, 280, 278 (1, 5, 7, $[M]^+$), 195 (3, $[M-CHCl_2]^+$), 142, 138, 136 (5, 18, 100, $[C_5H_6Cl_2]^+$), 103, 101 (33, 67, $[C_5H_6Cl]^+$), 77 (68, $[C_6H_5]^+$).

HRMS (EI): m/z $[M]^+$ calcd for $C_{11}H_{12}O_2S^{35}Cl_2$: 277.9935; found: 277.9943.

Synthesis of (1-methyl-4-piperidinyl)methyl phenyl sulfone (2h)

Preparation of (1-methyl-4-piperidinyl)methyl phenyl sulfide was performed according to Mitsunobu reaction protocol.[11] Oxidation step was performed according to the reported procedure.[1]

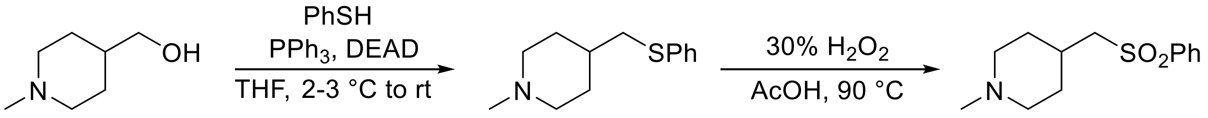

2h, 29%
over 2 steps

A 100 mL Schlenk flask was charged with PPh_3 (4.14 g, 15.8 mmol, 1.05 equiv.) and flushed with argon. Dry THF (56 mL) was added and mixture was immersed in ice/water bath (2-3 °C). To the stirred reaction mixture DEAD (2.78 g, 15.9 mmol, 1.06 equiv.) was added dropwise and then mixture of 1-methyl-4-piperidinemethanol (1.94 g, 15.0 mmol, 1.00 equiv.) in dry THF (10 mL) was added. After 30 min PhSH (1.98 g, 18.0 mmol, 1.20 equiv.) was added, and reaction mixture was stirred for 72 h. Then mixture was evaporated and separated with column chromatography (eluent: ethyl acetate, then dichloromethane, then dichloromethane/MeOH 50:1 to 10:1) yielding crude (1-methyl-4-piperidinyl)methyl phenyl sulfide. A 50 mL round-bottom flask was charged with crude (1-methyl-4-piperidinyl)methyl phenyl sulfide, AcOH (10 mL) was added, to the stirred reaction mixture $H_2O_{2\ aq}$ (3.07 mL, 30.1 mmol, 30% v/v, 2.01 equiv.) was added, and mixture was heated to 90 °C (oil bath). After 8 h mixture was evaporated with toluene (3 × 25 mL), K_2CO_3 (2.07 g, 15.0 mmol, 1.00 equiv.) and dichloromethane (10 mL) were added, and mixture was stirred overnight. Then, the mixture was passed through a pad of celite, evaporated, and separated with column chromatography (eluent: dichloromethane/MeOH 10:1 with 1% v/v NEt_3) yielding **2h** (1.11 g, 4.37 mmol, **29%** over 2 steps) as a yellow oil.

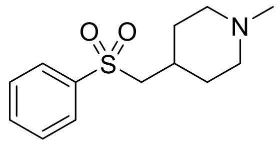

(1-methyl-4-piperidinyl)methyl phenyl sulfone, **2h**

yellow oil
eluent: dichloromethane/MeOH 10:1 with 1% v/v NEt_3

^{1}H NMR (400 MHz, $CDCl_3$): δ = 7.82 – 7.77 (m, 2H), 7.57 – 7.52 (m, 1H), 7.50 – 7.41 (m, 2H), 2.92 (d, *J* = 6.3 Hz, 2H), 2.75 (d, *J* = 12.1 Hz, 2H), 2.18 (s, 3H), 1.99 – 1.86 (m, 3H), 1.82 – 1.74 (m, 2H), 1.44 – 1.29 (m, 2H).

^{13}C NMR (100 MHz, $CDCl_3$): δ = 139.8, 133.5, 129.1, 127.4, 61.6, 54.7, 45.7, 31.5, 29.9.

MS (EI): m/z (%) = 254 (28, $[M+H]^+$), 253 (65, $[M]^+$), 252 (75, $[M-H]^+$), 238 (18, $[M-CH_3]^+$), 112 (100, $[M-PhSO_2]^+$).

HRMS (EI): m/z $[M]^+$ calcd for $C_{13}H_{19}NO_2S$: 253.1137; found: 253.1127.

Synthesis of isopropyl phenyl sulfone (2i)

Isopropyl phenyl sulfone was prepared according to the reported procedure.[1] Alkylation step was performed using K_2CO_3 in MeCN.

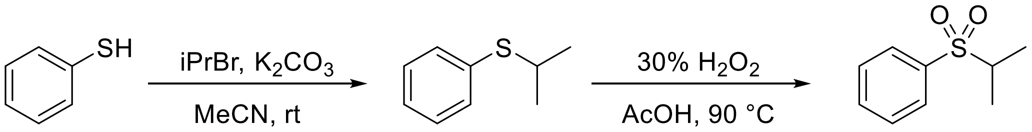

2i, 81%
over 2 steps

A 250 mL round-bottom flask was charged with K_2CO_3 (13.8 g, 100 mmol, 1.96 equiv.), MeCN (50 mL) and immersed in water bath (rt). Thiophenol (5.63 g, 51.1 mmol, 1.00 equiv.) and isopropyl bromide (9.17 g, 75.0 mmol, 1.47 equiv.) were added, and mixture was vigorously stirred. After 26 h H_2O (50 mL) was added, MeCN was evaporated, and afterwards mixture was extracted with ethyl acetate (3 × 50 mL). Combined organic phases were washed with H_2O (50 mL), brine (50 mL) and dried over anhydrous $MgSO_4$. Mixture was filtered, evaporated, and placed in 100 mL round-bottom flask. Acetic acid (20 mL) was added, flask was heated to 50 °C (oil bath), to the stirred reaction mixture $H_2O_{2\,aq}$ (15 mL, 147 mmol, 30% v/v, 2.88 equiv.) was added, and the mixture was heated to 90 °C (oil bath). After 2 h mixture was poured onto water/ice (200 mL), solid Na_2CO_3 was added to pH = 7. Mixture was extracted with ethyl acetate (3 × 50 mL), combined organic phases were washed with $Na_2SO_{3\,aq}$ (50 mL, 10% w/v), brine (50 mL) and dried over anhydrous $MgSO_4$. Mixture was filtered, evaporated, and dried under vacuum yielding **2i** (7.60 g, 41.2 mmol, **81%** over 2 steps) as a colorless oil.

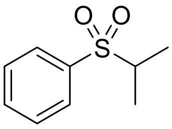

isopropyl phenyl sulfone, **2i**

colorless oil

^{1}H NMR (400 MHz, $CDCl_3$): δ = 7.84 – 7.78 (m, 2H), 7.62 – 7.55 (m, 1H), 7.53 – 7.46 (m, 2H), 3.13 (hept, *J* = 6.9 Hz, 1H), 1.21 (d, *J* = 6.9 Hz, 6H).

^{13}C NMR (100 MHz, $CDCl_3$): δ = 136.7, 133.5, 128.9, 128.8, 55.3, 15.5.

^{1}H NMR, ^{13}C NMR spectra were consistent with those reported in the literature.[12]

2.2. Syntheses of neopentyl alkanesulfonates (3a-3c, 3e)

Neopentyl alkanesulfonates were prepared from corresponding sulfonyl chlorides and neopentanol, according to the reported procedure.[13] Letter indexes of the compounds (**3**) refer to the same alkyl groups, as for alkyl phenyl sulfones (**2**). i.e.: **2a** and **3a** are methylating agents, **2b** and **3b** are ethylating agents, etc.

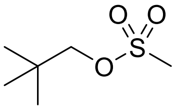

3a

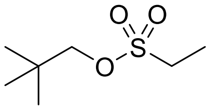

3b

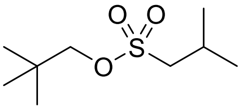

3c

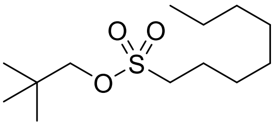

3e

Synthesis of neopentyl methanesulfonate (3a)

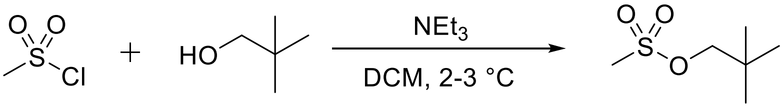

3a, 74%

A 250 mL round-bottom flask was charged with neopentyl alcohol (4.43 g, 50.3 mmol, 1.00 equiv.), dichloromethane (60 mL), triethylamine (5.80 g, 57.0 mmol, 1.13 equiv.), flushed with argon, and cooled in ice/water bath (ca. 2-3 °C). To the stirred reaction mixture methanesulfonyl chloride (6.49 g, 56.7 mmol, 1.13 equiv.) was added dropwise. After 2.5 h H_2O (50 mL) was added, phases were separated, and aqueous phase was extracted with ethyl acetate (3 × 50 mL). Combined organic phases were washed with H_2O (50 mL), brine (50 mL), and dried over anhydrous $MgSO_4$. Mixture was filtered, evaporated, and crude product was separated by distillation (78 °C, 4 mbar) yielding **3a** (6.17 g, 37.1 mmol, **74%**) as a colorless oil.

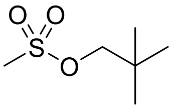

neopentyl methanesulfonate, **3a**

colorless oil

^{1}H NMR (400 MHz, $CDCl_3$): δ = 3.77 (s, 2H), 2.91 (s, 3H), 0.89 (s, 9H).

^{13}C NMR (100 MHz, $CDCl_3$): δ = 78.9, 36.6, 31.4, 25.7.

^{1}H NMR, ^{13}C NMR spectra were consistent with those reported in the literature.[1]

Synthesis of neopentyl ethanesulfonate (3b)

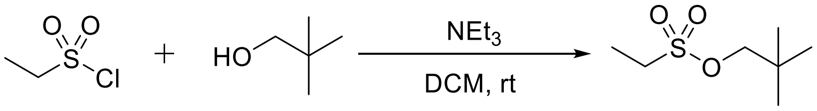

3b, 83%

A 250 mL round-bottom flask was charged with neopentyl alcohol (3.56 g, 40.4 mmol, 1.00 equiv.), dichloromethane (60 mL), triethylamine (5.81 g, 57.4 mmol, 1.43 equiv.), and immersed in water bath (rt). To the stirred reaction mixture ethanesulfonyl chloride (5.23 g, 40.6 mmol, 1.00 equiv.) was added dropwise. After 2 h NH_4Cl_{aq} (50 mL, 10% w/v) was added, phases were separated, and aqueous phase was extracted with ethyl acetate (3 × 50 mL). Combined organic phases were washed with H_2O (50 mL), brine (50 mL), and dried over anhydrous $MgSO_4$. Mixture was filtered, evaporated, and product was distilled (79 °C, 3.3 mbar) yielding **3b** (6.03 g, 33.4 mmol, **83%**) as a colorless oil.

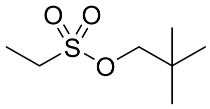

neopentyl ethanesulfonate, **3b**

colorless oil

^{1}H NMR (400 MHz, CDCl$_3$): δ = 3.78 (s, 2H), 3.06 (q, *J* = 7.4 Hz, 2H), 1.35 (t, *J* = 7.4 Hz, 3H), 0.92 (s, 9H).

^{13}C NMR (100 MHz, CDCl$_3$): δ = 78.5, 44.4, 31.6, 25.9, 8.0.

^{1}H NMR spectrum was consistent with that reported in the literature.[14]

Synthesis of neopentyl isobutanesulfonate (3c)

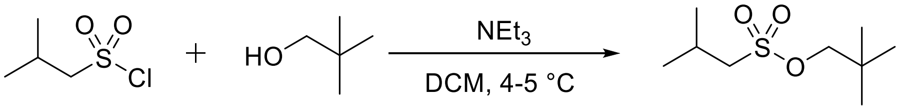

3c, 77%

A 250 mL round-bottom flask was charged with neopentyl alcohol (2.82 g, 32.0 mmol, 1.02 equiv.), dichloromethane (48 mL), isobutanesulfonyl chloride (5.01 g, 31.3 mmol, 1.00 equiv.), and immersed in water/ice bath (4-5 °C). To the stirred reaction mixture triethylamine (4.50 g, 44.5 mmol, 1.42 equiv.) was added dropwise. After 2 h H_2O (50 mL) was added, phases were separated, and aqueous phase was extracted with ethyl acetate (3 × 50 mL). Combined organic phases were washed with H_2O (50 mL), brine (50 mL), and dried over anhydrous $MgSO_4$. Mixture was filtered, evaporated, and product was distilled (93-95 °C, 4.0 mbar) yielding **3c** (5.05 g, 24.2 mmol, **77%**) as a colorless oil.

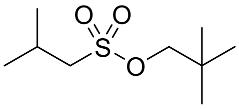

neopentyl isobutanesulfonate, **3c**

colorless oil

^{1}H NMR (400 MHz, $CDCl_3$): δ = 3.81 (s, 2H), 2.95 (d, *J* = 6.6 Hz, 2H), 2.34 – 2.19 (m, 1H), 1.08 (d, *J* = 6.8 Hz, 6H), 0.94 (s, 9H).

^{13}C NMR (100 MHz, $CDCl_3$): δ = 78.2, 57.6, 31.6, 26.0, 24.8, 22.3.

HRMS (ESI): m/z $[M+Na]^+$ calcd for $C_9H_{20}O_3SNa$: 231.1031; found: 231.1032.

Synthesis of neopentyl octanesulfonate (3e)

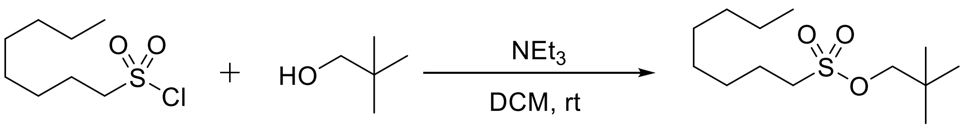

3e, 90%

A 250 mL round-bottom flask was charged with neopentyl alcohol (4.41 g, 50.0 mmol, 1.00 equiv.), dichloromethane (60 mL), triethylamine (7.11 g, 70.3 mmol, 1.41 equiv.), flushed with argon, and immersed in water bath (rt). To the stirred reaction mixture octanesulfonyl chloride (10.6 g, 50.0 mmol, 1.00 equiv.) was added dropwise. After 2 h H_2O (50 mL) was added, phases were separated, and aqueous phase was extracted with ethyl acetate (3 × 50 mL). Combined organic phases were washed with H_2O (50 mL), brine (50 mL), and dried over anhydrous $MgSO_4$. Mixture was filtered, evaporated, and product was separated with column chromatography (eluent: cyclohexane, then cyclohexane/ethyl acetate 20:1 to 10:1) yielding **3e** (11.9 g, 45.2 mmol, **90%**) as a pale yellow oil.

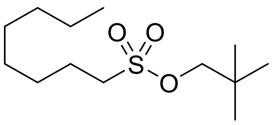

neopentyl octanesulfonate, **3e**

pale yellow oil
eluent: cyclohexane, then cyclohexane/ethyl acetate 20:1 to 10:1

^{1}H NMR (400 MHz, $CDCl_3$): δ = 3.79 (s, 2H), 3.05 – 3.00 (m, 2H), 1.84 – 1.72 (m, 2H), 1.42 – 1.31 (m, 2H), 1.31 – 1.16 (m, 8H), 0.92 (s, 9H), 0.81 (t, *J* = 6.9 Hz, 3H).

^{13}C NMR (100 MHz, $CDCl_3$): δ = 78.3, 49.9, 31.6, 31.5, 28.8, 28.7, 28.0, 25.9, 23.3, 22.4, 13.9.

^{1}H NMR, ^{13}C NMR spectra were consistent with those reported in the literature.[13]

2.3. Syntheses of nitroarenes (1a-1i)

Syntheses of nitroarenes **1d**, **1f**, and **1h** were described below. Other nitroarenes were purchased from commercial suppliers (see the General Information).

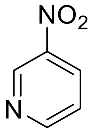

1a

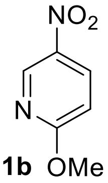

1b

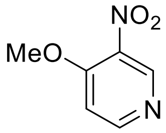

1c

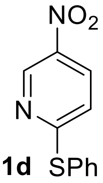

1d

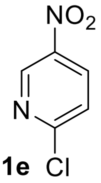

1e

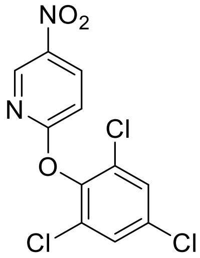

1f

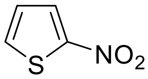

1g

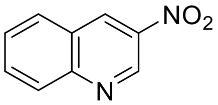

1h

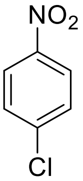

1i

Synthesis of 5-nitro-2-(phenylthio)pyridine (1d)

5-Nitro-2-(phenylthio)pyridine (**1d**) was prepared from 2-bromo-5-nitropyridine and thiophenol using NEt_3 in DMF at rt.

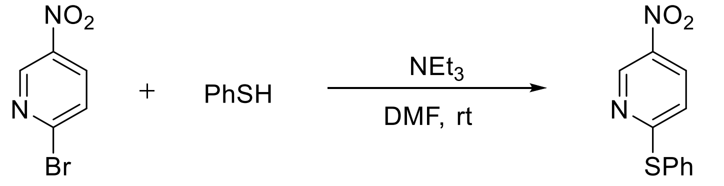

1d, 86%

A 30 mL Schlenk flask was charged with 2-bromo-5-nitropyridine (2.03 g, 10.0 mmol, 1.00 equiv.), thiophenol (1.16 g, 10.5 mmol, 1.05 equiv.), and flushed with argon. Dry DMF (12 mL) was added, and Schlenk flask was immersed in water bath (rt). To the stirred reaction mixture NEt_3 (1.52 g, 15.1 mmol, 1.51 equiv.) was added. After 30 min the mixture was transferred to separatory funnel, NH_4Cl_{aq} (50 mL, 10% w/v) was added, mixture was extracted with ethyl acetate (3 × 50 mL), combined organic phases were washed with $LiCl_{aq}$ (50 mL, 1.0 M), H_2O (50 mL), brine (50 mL), and dried over anhydrous $MgSO_4$. Mixture was filtered, evaporated, and recrystallized from dichloromethane/heptane mixture yielding **1d** (2.00 g, 8.63 mmol, **86%**) as off-white crystals.

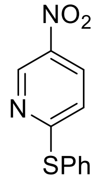

5-nitro-2-(phenylthio)pyridine, **1d**

off-white crystals, **mp** 118.0 – 119.0 °C

^{1}H NMR (400 MHz, $CDCl_3$): δ = 9.17 (d, *J* = 2.7 Hz, 1H), 8.16 (dd, *J* = 8.9, 2.7 Hz, 1H), 7.62 – 7.57 (m, 2H), 7.52 – 7.45 (m, 3H), 6.91 (dd, *J* = 8.9, 0.7 Hz, 1H).

^{13}C NMR (100 MHz, $CDCl_3$): δ = 170.3, 145.0, 141.0, 135.5, 131.2, 130.4, 130.1, 128.4, 119.7.

^{1}H NMR, ^{13}C NMR spectra[15] and mp[16] were consistent with those reported in the literature.

Synthesis of 2-(2,4,6-trichlorophenoxy)-5-nitropyridine (1f)

2-(2,4,6-Trichlorophenoxy)-5-nitropyridine (**1f**) was prepared according to modified procedure reported in the literature.[17]

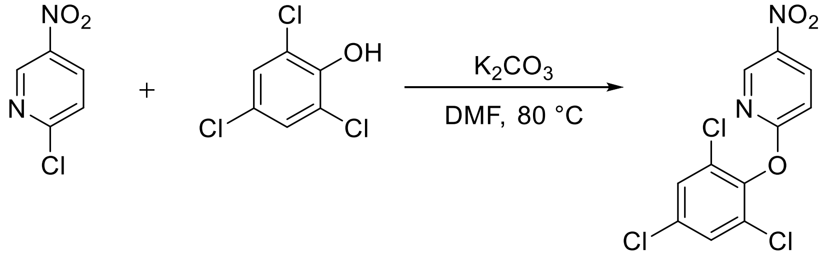

1f, 87%

A 100 mL round-bottom flask was charged with K_2CO_3 (8.31 g, 60.1 mmol, 3.00 equiv.), dried under vacuum, and flushed with argon. Solution of 2-chloro-5-nitropyridine (3.17 g, 20.0 mmol, 1.00 equiv.) and 2,4,6-trichlorophenol (4.15 g, 21.0 mmol, 1.05 equiv.) in dry DMF (20 mL) was added, and mixture was stirred at 80 °C (oil bath). After 20 h mixture was transferred to separatory funnel, H_2O (50 mL) was added, mixture was extracted with ethyl acetate (3 × 50 mL), combined organic phases were washed with $LiCl_{aq}$ (50 mL, 1.0 M), H_2O (50 mL), brine (50 mL), and dried over anhydrous $MgSO_4$. Mixture was filtered, evaporated, and recrystallized from dichloromethane/heptane mixture yielding in three crops **1f** (5.59 g, 17.5 mmol, **87%**) as light brown crystals.

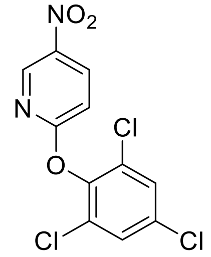

2-(2,4,6-trichlorophenoxy)-5-nitropyridine, **1f**

light brown crystals, **mp** 104.0 – 106.0 °C

^{1}H NMR (400 MHz, CDCl$_3$): δ = 8.94 (dd, *J* = 2.8, 0.6 Hz, 1H), 8.54 (dd, *J* = 9.0, 2.8 Hz, 1H), 7.41 (s, 2H), 7.22 (dd, *J* = 9.0, 0.6 Hz, 1H).

^{13}C NMR (100 MHz, CDCl$_3$): δ = 164.4, 144.6, 144.5, 141.1, 135.4, 132.0, 129.9, 128.8, 111.0.

MS (EI): m/z (%) = 287, 285, 283 (14, 77, 100, $[M\text{-}Cl]^+$), 241, 239, 237 (6, 39, 59, $[M\text{-}Cl\text{-}NO_2]^+$).

HRMS (EI): m/z $[M]^+$ calcd for $C_{11}H_5N_2O_3{}^{35}Cl_3$: 317.9366; found: 319.9360.

Synthesis of 3-nitroquinoline (1h)

3-Nitroquinoline (**1h**) was prepared according to procedure reported in the literature.[18]

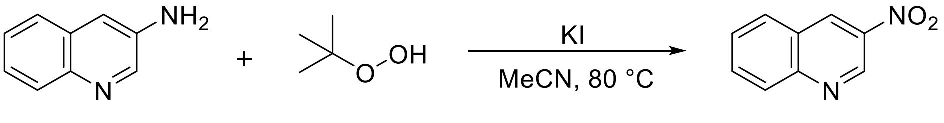

A 100 mL round-bottom flask was charged with 3-aminoquinoline (2.31 g, 16.0 mmol, 1.00 equiv.), KI (133 mg, 0.80 mmol, 5 mol%) and MeCN (40 mL). To the stirred reaction mixture solution of tert-butyl hydroperoxide (7.85 g, 61.0 mmol, 3.80 equiv., 70% w/w in H_2O) in MeCN (8 mL) was added dropwise over 25 min, and then mixture was heated to 80 °C (oil bath). After 24 h $Na_2S_2O_3$ aq (100 mL, sat.) was added, mixture was extracted with ethyl acetate (3 × 100 mL), combined organic phases were washed with brine (50 mL), and dried over anhydrous $MgSO_4$. Mixture was filtered, evaporated, and product was separated with column chromatography (eluent: cyclohexane/ethyl acetate 20:1 to 1:1) yielding **1h** (357 mg, 2.05 mmol, **13%**) as brown crystals.

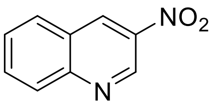

3-nitroquinoline, **1h**

brown crystals, **mp** 126.5 – 127.5 °C
eluent: cyclohexane/ethyl acetate 20:1 to 1:1

^{1}H NMR (400 MHz, CDCl$_3$): δ = 9.60 (d, *J* = 2.5 Hz, 1H), 9.00 (d, *J* = 2.5 Hz, 1H), 8.20 (d, *J* = 8.5 Hz, 1H), 8.01 (d, *J* = 8.4 Hz, 1H), 7.96 – 7.87 (m, 1H), 7.75 – 7.66 (m, 1H).

^{13}C NMR (100 MHz, CDCl$_3$): δ = 150.0, 144.0, 140.9, 133.4, 132.2, 129.8, 129.7, 128.8, 125.9.

^{1}H NMR, ^{13}C NMR spectra[19] and mp[20] were consistent with those reported in the literature.

3. Alkylation of nitroarenes

3.1. General Procedure for alkylation of nitroarenes

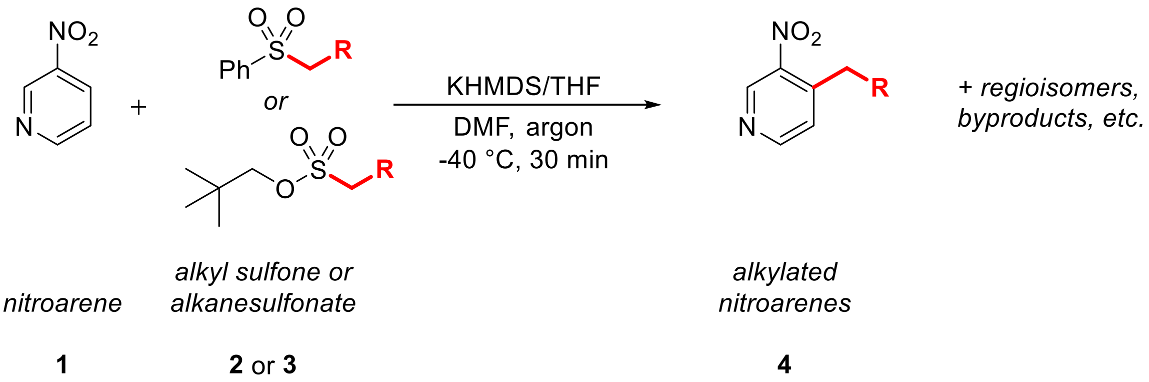

A 30 mL Schlenk flask was charged with nitroarene (**1**) (2.00 mmol, 1.00 equiv.), carbanion precursor (**2** or **3**) (2.40 mmol, 1.20 equiv.) and flushed with argon. Dry DMF (6 mL) was added, and after dissolution of substrates mixture was cooled to -40 °C.* To the stirred reaction mixture KHMDS (5.00 mmol, 2.50 equiv., solution in THF**) was added rapidly in one portion. After 30 min reaction mixture was quenched with NH_4Cl_{aq} (10 mL, 10% w/v). Mixture was transferred to separatory funnel, NH_4Cl_{aq} (50 mL, 10% w/v) was added, mixture was extracted with ethyl acetate (3 × 50 mL), combined organic phases were washed with $LiCl_{aq}$ (50 mL, 1.0 M), H_2O (50 mL), brine (50 mL), and dried over anhydrous $MgSO_4$. Mixture was filtered, evaporated and separated with column chromatography (length = ca. 40-50 cm, ø = 3 cm, 250-350 mL of silica gel), and eluted with eluents indicated hereinafter.

* - methylation and ethylation of 3-nitropyridine (**1a**) were performed under slightly different conditions (see Table on next page)

** - for concentrations of commercially-available solutions of KHMDS in THF used in this study see the General Information

3.2. Preparative alkylation of nitroarenes

entry	conditions			yields [%]			
	nitroarene	carbanion precursor	variation from General Procedure	alkylated products	σ^H-adduct	nitroarene recovered	side products
1	**1a**	**2a**	-	**4a**, 12%	-	17%	13%[a]; **6c**[b]
2	**1a**	**3a**	-	**4a**, 20%	-	10%	5%[a]; **6c**, 24%
3	**1a**	**3a**	**-60 °C, 3 min**	**4a**, 38%	-	5%	7%[a]; **6c**[b]
4	**1a**	**2b**	-	**4b**, 28%	-	4%	-
5	**1a**	**2b**	**3 min**	**4b**, 35%	15%[c]	5%	-[d]
6	**1a**	**2c**	-	**4c**, 71%; **4c'**, 10%	2%[c]	1%	-[d]
7	**1a**	**3c**	-	**4c**, 73%; **4c'**, 9%	-	1%	-[d]
8	**1a**	**2d**	-	**4d**, 70%	6%[c]	-	-[d]
9	**1a**	**3e**	-	**4e**, 71%; **4e'**, 9%	-	-	**6d**, 4%; -[d]
10	**1a**	**2e**	-	**4e**, 53%; **4e'**, 10%	-	-[b]	**6d**, 2%; -[d]
11	**1a**	**2f**	-	**4f**, 72%; **4f'**, 9%	-	-	-[d]
12	**1a**	**2g**	-	**4g**, 64%	-	-	-[d]
13	**1a**	**2h**	-	**4h**, 61%	-	4%	-[e]
14	**1b**	**3a**	-	**4i**, 54%	-	-	-
15	**1b**	**3e**	-	**4j**, 55%			-[d]
16	**1c**	**3e**	-	**4k**, 40%; **4k'**, 14%	-	-	-[f]
17	**1d**	**2c**	-	**4l**, 69%	4%[c]	4%	-[d]
18	**1d**	**2d**	-	**4m**, 86%; **4m'**, 5%	-	3%	-
19	**1d**	**3e**	-	**4n**, 64%; **4n'**, 6%	-	-	-
20	**1d**	**2f**	-	**4o**, 73%; **4o'**, 8%	3%[c]	-	-
21	**1d**	**2g**	-	**4p**, 53%; **4p'**, 9%	-	3%	-
22	**1e**	**3e**	-	**4q**, 36%	-	3%	3%[d]
23	**1f**	**3e**	-	**4r**, 50%; **4r'**, 26%	-	-	-
24	**1g**	**2c**	-	**4s**, 55%	-	-	-
25	**1g**	**3e**	-	**4t**, 58%	-	-	-
26	**1a**	**2i**	-	-	**5a**, 43%[g]	-	-
27	**1h**	**2b**	-	5%[h]	**5b**, 68%	18%	-
28	**1h**	**2a**	-	**4u**, 79%	-	4%	-
29	**1h**	**2i**	-	-	**5c**, 11%	85%	-
30	**1i**	**3e**	-	**4v**, 15%	-	8%	**6a**, 39%; **10**, 10%
31	**1i**	**3b**	-	-	-	28%	**6b**, 23%[i]

a - ONSH (Oxidative Nucleophilic Substitution of Hydrogen) type product was formed, pure analytical sample was not available
b – due to sample contamination yield was not established
c – pure analytical sample was not available
d – ≤5% of isomeric products, pure analytical samples were not available
e – 9% of 2-((1-methyl-4-piperidinyl)methyl)-5-nitropyridine was formed, pure analytical sample was not available
f – ca. 17% of *(E)*-oct-1-enylated products were formed, pure analytical samples were not available
g – ≤5% of isomeric σ-adducts were formed, pure analytical samples were not available
h – 4-ethyl-3-nitroquinoline was formed, pure analytical sample was not available
i – ca. 18% of 2-vinyl-4-chloronitrobenzene was formed, pure analytical sample was not available

3.3. Informations concerning isolation of nitroarenes

- Reactions were carried out in a Schlenk flask (⌀ 30 × 92 mm) and stirred with magnetic bar (fish, ⌀ 10 × 20 mm) at 300 rpm,
- Solution of KHMDS in THF was added rapidly (during ca. 3-5 s) into vortex of a stirred reaction mixture. For concentrations of commercially-available solutions of KHMDS in THF used in this study see the General Information,
- Nitroarenes **1b**, **1d**, and **1h** precipitate from DMF while cooling to -40 °C, however addition of KHMDS in THF makes the reaction mixture homogenous,
- In most cases it is crucial to use cyclohexane/toluene (for isolation of less polar products) or toluene/ethyl acetate (for isolation of more polar products) mixtures as eluent in column chromatography, while other popular eluents like cyclohexane, cyclohexane/ethyl acetate, cyclohexane/diethyl ether are less efficient (even if they might provide better separation at TLC),
- In general, products alkylated *para* to the NO_2 group are eluted faster (are less polar) than *orto* isomers (eluted in cyclohexane/toluene and toluene/ethyl acetate mixtures).

3.4. Characterization of the alkylated products

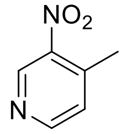

4-methyl-3-nitropyridine, **4a**

Yield:
56 mg (0.41 mmol, **20%**) from **1a** (251 mg, 2.02 mmol) and **3a**
35 mg (0.25 mmol, **12%**) from **1a** (252 mg, 2.03 mmol) and **2a**
104 mg (0.76 mmol, **38%**) from **1a** (248 mg, 2.00 mmol) and **3a** at **-60 °C, 3 min**

yellow oil
eluent: toluene/ethyl acetate 50:1 to 2:1

^{1}H NMR (400 MHz, $CDCl_3$): δ = 9.13 (s, 1H), 8.62 (d, *J* = 5.0 Hz, 1H), 7.29 (d, *J* = 5.0 Hz, 1H), 2.62 (s, 3H).

^{13}C NMR (100 MHz, $CDCl_3$): δ = 152.9, 145.8 (ovl), 142.9, 127.1, 19.9.

^{1}H NMR spectrum was consistent with that reported in the literature, while ^{13}C NMR spectrum was moderately consistent.[21]

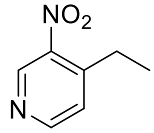

4-ethyl-3-nitropyridine, **4b**

Yield:
83 mg (0.55 mmol, **28%**) from **1a** (248 mg, 2.00 mmol) and **2b**
107 mg (0.70 mmol, **35%**) from **1a** (251 mg, 2.02 mmol) and **2b** at **-40 °C, 3 min**

pale yellow oil
eluent: toluene/ethyl acetate 50:1 to 2:1

^{1}H NMR (400 MHz, $CDCl_3$): δ = 9.05 (s, 1H), 8.65 (d, *J* = 5.1 Hz, 1H), 7.31 (d, *J* = 5.1 Hz, 1H), 2.94 (q, *J* = 7.5 Hz, 2H), 1.28 (t, *J* = 7.5 Hz, 3H).

^{13}C NMR (100 MHz, $CDCl_3$): δ = 152.9, 148.0, 145.8, 145.6, 125.2, 25.5, 13.7.

^{1}H NMR, ^{13}C NMR spectra were consistent with those reported in the literature.[21]

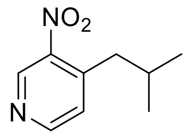

4-isobutyl-3-nitropyridine, **4c**

Yield:
257 mg (1.43 mmol, **71%**) from **1a** (250 mg, 2.01 mmol) and **2c**
678 mg (3.76 mmol, **73%**) from **1a** (636 mg, 5.13 mmol) and **3c**

orange oil
eluent: toluene/ethyl acetate 50:1 to 4:1

^{1}H NMR (400 MHz, $CDCl_3$): δ = 9.00 (s, 1H), 8.59 (d, *J* = 5.1 Hz, 1H), 7.20 (d, *J* = 5.1 Hz, 1H), 2.76 (d, *J* = 7.2 Hz, 2H), 1.95 – 1.80 (m, 1H), 0.86 (d, *J* = 6.7 Hz, 6H).

MS (EI): m/z (%): 181 (4, $[M+H]^+$), 163 (15, $[M-OH]^+$), 138 (37, $[M-C_3H_6]^+$), 121 (100, $[M-OC_3H_7]^+$), 44 (86, $[C_3H_8]^+$).

HRMS (EI): m/z $[M]^+$ calcd for $C_9H_{12}N_2O_2$: 180.0899; found: 180.0892.

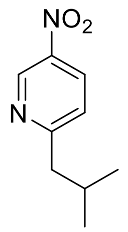

2-isobutyl-5-nitropyridine, **4c'**

Yield:
38 mg (0.21 mmol, **10%**) from **1a** (250 mg, 2.01 mmol) and **2c**
79 mg (0.44 mmol, **9%**) from **1a** (636 mg, 5.13 mmol) and **3c**

pale yellow oil
eluent: toluene/ethyl acetate 50:1 to 4:1

^{1}H NMR (400 MHz, $CDCl_3$): δ = 9.33 (d, *J* = 2.5 Hz, 1H), 8.35 (dd, *J* = 8.6, 2.6 Hz, 1H), 7.28 (d, *J* = 8.6 Hz, 1H), 2.76 (d, *J* = 7.2 Hz, 2H), 2.20 – 2.06 (m, 1H), 0.92 (d, *J* = 6.7 Hz, 6H).

^{13}C NMR (100 MHz, $CDCl_3$): δ = 168.5, 144.7, 142.5, 131.0, 123.5, 47.5, 29.2, 22.3.

MS (EI): m/z (%): 181 (11, $[M+H]^+$), 180 (4, $[M]^+$), 165 (39, $[M-CH_3]^+$), 138 (100, $[M-C_3H_6]^+$).

HRMS (EI): m/z $[M+H]^+$ calcd for $C_9H_{13}N_2O_2$: 181.0977; found: 181.0972.

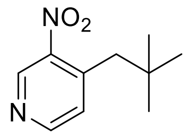

4-(tert-butylmethyl)-3-nitropyridine, **4d**

Yield:
276 mg (1.42 mmol, **70%**) from **1a** (253 mg, 2.04 mmol)

yellow oil
eluent: toluene/ethyl acetate 50:1 to 4:1

^{1}H NMR (400 MHz, $CDCl_3$): δ = 8.91 (s, 1H), 8.56 (d, *J* = 5.1 Hz, 1H), 7.14 (d, *J* = 5.1 Hz, 1H), 2.90 (s, 2H), 0.79 (s, 9H).

^{13}C NMR (100 MHz, $CDCl_3$): δ = 151.6, 147.3, 145.3, 142.8, 127.7, 43.7, 33.1, 28.9.

MS (EI): m/z (%): 195 (2, $[M+H]^+$), 179 (9, $[M-CH_3]^+$), 138 (37, $[M-C_4H_8]^+$), 121 (22, $[M-OC_4H_9]^+$), 57 (100, $[C_4H_9]^+$).

HRMS (EI): m/z $[M+H]^+$ calcd for $C_{10}H_{15}N_2O_2$: 195.1134; found: 195.1138.

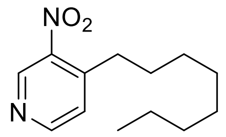

4-octyl-3-nitropyridine, **4e**

Yield:
335 mg (1.42 mmol, **71%**) from **1a** (248 mg, 2.00 mmol) and **3e**
256 mg (1.08 mmol, **53%**) from **1a** (251 mg, 2.02 mmol) and **2e**

red liquid
eluent: toluene, then toluene/ethyl acetate 100:1 to 3:1

^{1}H NMR (400 MHz, $CDCl_3$): δ = 9.02 (s, 1H), 8.60 (d, *J* = 5.1 Hz, 1H), 7.26 (d, *J* = 5.1, 1H), 2.89 – 2.83 (m, 2H), 1.64 – 1.54 (m, 2H), 1.38 – 1.13 (m, 10H), 0.81 (t, *J* = 6.9 Hz, 3H).

^{13}C NMR (100 MHz, $CDCl_3$): δ = 152.7, 146.7, 145.8, 145.7, 125.8, 32.2, 31.7, 29.7, 29.4, 29.1, 29.0, 22.5, 14.0.

MS (EI): m/z (%): 237 (2, $[M+H]^+$), 236 (3, $[M]^+$), 219 (89, $[M-OH]^+$), 44 (100, $[C_3H_8]^+$).

HRMS (EI): m/z $[M]^+$ calcd for $C_{13}H_{20}N_2O_2$: 236.1525; found: 236.1530.

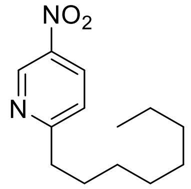

2-octyl-5-nitropyridine, **4e'**

Yield:
43 mg (0.18 mmol, **9%**) from **1a** (248 mg, 2.00 mmol) and **3e**
51 mg (0.21 mmol, **10%**) from **1a** (251 mg, 2.02 mmol) and **2e**

yellowish oil
eluent: toluene, then toluene/ethyl acetate 100:1 to 3:1

^{1}H NMR (400 MHz, $CDCl_3$): δ = 9.32 (d, *J* = 2.5 Hz, 1H), 8.35 (dd, *J* = 8.6, 2.7 Hz, 1H), 7.31 (d, *J* = 8.5 Hz, 1H), 2.92 – 2.86 (m, 2H), 1.78 – 1.69 (m, 2H), 1.38 – 1.16 (m, 10H), 0.85 (t, *J* = 6.9 Hz, 3H).

^{13}C NMR (100 MHz, $CDCl_3$): δ = 169.4, 144.6, 142.5, 131.3, 122.8, 38.5, 31.8, 29.5, 29.32, 29.25, 29.1, 22.6, 14.1.

MS (EI): m/z (%): 237 (1, $[M+H]^+$), 236 (4, $[M]^+$), 165 (33, $[M-C_5H_{11}]^+$), 151 (49, $[M-C_6H_{13}]^+$), 138 (100, $[M-C_7H_{14}]^+$),

HRMS (EI): m/z $[M]^+$ calcd for $C_{13}H_{20}N_2O_2$: 236.1525; found: 236.1527.

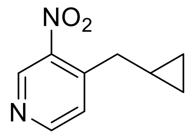

4-(cyclopropylmethyl)-3-nitropyridine, **4f**

Yield:
258 mg (1.45 mmol, **72%**) from **1a** (248 mg, 2.00 mmol)

light orange oil
eluent: toluene/ethyl acetate 50:1 to 1:1

^{1}H NMR (400 MHz, CDCl$_3$): δ = 9.00 (s, 1H), 8.63 (d, *J* = 5.1 Hz, 1H), 7.50 (d, *J* = 5.1, 1H), 2.81 (d, *J* = 7.0 Hz, 2H), 1.03 – 0.92 (m, 1H), 0.58 – 0.52 (m, 2H), 0.21 – 0.15 (m, 2H).

^{13}C NMR (100 MHz, CDCl$_3$): δ = 152.9, 146.1, 145.6, 145.4, 125.1, 36.1, 9.5, 4.8.

MS (EI): m/z (%): 179 (65, $[M+H]^+$), 161 (19, $[M-OH]^+$), 135 (100, $[M-C_3H_7]^+$).

HRMS (EI): m/z $[M+H]^+$ calcd for $C_9H_{11}N_2O_2$: 179.0821; found: 179.0820.

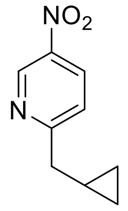

2-(cyclopropylmethyl)-5-nitropyridine, **4f’**

Yield:
32 mg (0.18 mmol, **9%**) form **1a** (248 mg, 2.00 mmol)

yellowish oil
eluent: toluene/ethyl acetate 50:1 to 1:1

^{1}H NMR (400 MHz, CDCl$_3$): δ = 9.33 (d, *J* = 2.6 Hz, 1H), 8.38 (dd, *J* = 8.6, 2.7 Hz, 1H), 7.47 (d, *J* = 8.5 Hz, 1H), 2.82 (d, *J* = 7.1 Hz, 2H), 1.15 – 1.04 (m, 1H), 0.62 – 0.56 (m, 2H), 0.28 – 0.23 (m, 2H).

^{13}C NMR (100 MHz, CDCl$_3$): δ = 168.8, 144.6, 142.6, 131.3, 122.4, 42.9, 10.2, 4.8.

MS (EI): m/z (%): 179 (9, $[M+H]^+$), 178 (48, $[M]^+$), 177 (100, $[M-H]^+$), 163 (68, $[M-CH_3]^+$), 131 (57, $[M-HNO_2]^+$).

HRMS (EI): m/z $[M+H]^+$ calcd for $C_9H_{11}N_2O_2$: 179.0821; found: 179.0814.

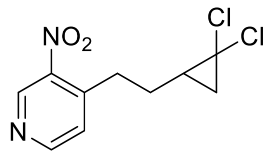

4-(2-(2,2-dichlorocyclopropyl)ethyl)-3-nitropyridine, **4g**

Yield:
336 mg (1.29 mmol, **64%**) from **1a** (250 mg, 2.01 mmol)

brown oil
eluent: toluene/ethyl acetate 20:1 to 5:1

^{1}H NMR (400 MHz, CDCl$_3$): δ = 9.09 (s, 1H), 8.66 (d, *J* = 5.1 Hz, 1H), 7.32 (d, *J* = 5.1 Hz, 1H), 3.19 – 3.01 (m, 2H), 2.00 – 1.79 (m, 3H), 1.63 – 1.50 (m, 2H), 1.12 – 1.03 (m, 1H).

^{13}C NMR (100 MHz, CDCl$_3$): δ = 153.1, 146.0, 145.6, 145.1, 126.2, 60.7, 31.4, 30.5, 29.9, 26.5.

MS (EI): m/z (%) = 265, 263, 261 (1, 5, 7, [M]$^+$), 121, 119 (31, 100, [OC_5H_8Cl]$^+$).

HRMS (EI): m/z [M]$^+$ calcd for $C_{10}H_{11}O_2N_2{}^{35}Cl_2$: 261.0198; found: 261.0192.

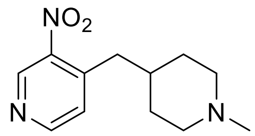

4-((1-methyl-4-piperidinyl)methyl)-3-nitropyridine, **4h**

Yield:
290 mg (1.23 mmol, **61%**) from **1a** (252 mg, 2.03 mmol)

orange oil
eluent: ethyl acetate then ethyl acetate/MeOH 50:1 to 10:1 (all with 2% v/v NEt_3)

^{1}H NMR (400 MHz, CDCl$_3$): δ = 9.11 (s, 1H), 8.65 (d, *J* = 5.0 Hz, 1H), 7.22 (d, *J* = 5.0 Hz, 1H), 3.07 (d, *J* = 12.3 Hz, 2H), 2.89 (d, *J* = 5.6 Hz, 2H), 2.42 (s, 3H), 2.23 – 2.13 (m, 2H), 1.71 – 1.63 (m, 5H).

^{13}C NMR (100 MHz, CDCl$_3$): δ = 152.9, 146.3, 145.7, 144.0, 127.1, 54.9, 45.1, 39.0, 34.9, 30.7.

MS (EI): m/z (%): 235 (27, [M]$^+$), 234 (55, [M-H]$^+$), 218 (48, [M-OH]$^+$), 98 (87, [$C_6H_{12}N$]$^+$), 96 (100, [$C_6H_{10}N$]$^+$).

HRMS (EI): m/z [M]$^+$ calcd for $C_{12}H_{17}N_3O_2$: 235.1321; found: 235.1313.

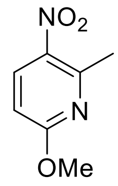

6-methoxy-2-methyl-3-nitropyridine, **4i**

Yield:
183 mg (1.09 mmol, **54%**) from **1b** (309 mg, 2.00 mmol)

white crystals, **mp** 67.5 – 68.5 °C
eluent: cyclohexane/toluene 3:1 to 1:1, then toluene, then toluene/ethyl acetate 20:1 to 5:1

^{1}H NMR (400 MHz, $CDCl_3$): δ = 8.19 (d, *J* = 9.0 Hz, 1H), 6.60 (d, *J* = 9.0 Hz, 1H), 3.95 (s, 3H), 2.74 (s, 3H).

^{13}C NMR (100 MHz, $CDCl_3$): δ = 164.7, 154.5, 139.6, 135.8, 108.9, 54.3, 24.5.

MS (EI): m/z (%): 169 (22, $[M+H]^+$), 168 (100, $[M]^+$), 167 (90, $[M-H]^+$), 151 (73, $[M-OH]^+$), 138 (50, $[M-OCH_2]^+$), 107 (87, $[M-CH_3NO_2]^+$).

HRMS (EI): m/z $[M]^+$ calcd for $C_7H_8N_2O_3$: 168.0535; found: 168.0533.

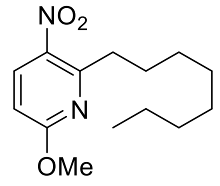

6-methoxy-2-octyl-3-nitropyridine, **4j**

Yield:
295 mg (1.11 mmol, **55%**) from **1b** (309 mg, 2.01 mmol)

white crystals, **mp** 56.5 – 57.5 °C
eluent: cyclohexane/toluene 5:1 to 1:1, then toluene, then toluene/ethyl acetate 20:1 to 5:1

^{1}H NMR (400 MHz, $CDCl_3$): δ = 8.15 (d, *J* = 8.9 Hz, 1H), 6.59 (d, *J* = 8.9 Hz, 1H), 3.97 (s, 3H), 3.07 – 3.01 (m, 2H), 1.78 – 1.68 (m, 2H), 1.41 – 1.17 (m, 10H), 0.83 (t, *J* = 6.9 Hz, 3H).

^{13}C NMR (100 MHz, $CDCl_3$): δ = 164.7, 157.9, 139.8, 135.9, 108.6, 54.2, 35.9, 31.8, 29.5, 29.3, 29.2, 28.2, 22.6, 14.0.

MS (EI): m/z (%): 266 (1, $[M]^+$), 265 (2, $[M-H]^+$), 249 (15, $[M-OH]^+$), 195 (69, $[M-C_5H_{11}]^+$), 168 (100, $[M-C_7H_{14}]^+$),.

HRMS (EI): m/z $[M]^+$ calcd for $C_{14}H_{22}N_2O_3$: 266.1630; found: 266.1619.

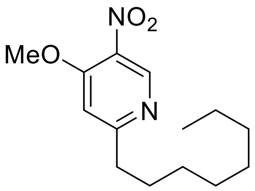

4-methoxy-2-octyl-5-nitropyridine, **4k**

Yield:
211 mg (0.79 mmol, **40%**) from **1c** (309 mg, 2.00 mmol)

light brown oil
eluent: cyclohexane/toluene 5:1 to 1:1, then toluene, then toluene/ethyl acetate 5:1

^{1}H NMR (400 MHz, $CDCl_3$): δ = 8.88 (s, 1H), 6.80 (s, 1H), 3.96 (s, 3H), 2.78 – 2.72 (m, 2H), 1.72 – 1.62 (m, 2H), 1.35 – 1.13 (m, 10H), 0.80 (t, *J* = 6.9 Hz, 3H).

^{13}C NMR (100 MHz, $CDCl_3$): δ = 169.6, 159.1, 146.7, 134.5, 106.8, 56.4, 38.8, 31.7, 29.4, 29.23, 29.19, 29.0, 22.5, 14.0.

MS (EI): m/z (%): 267 (3, $[M+H]^+$), 266 (5, $[M]^+$), 265 (3, $[M-H]^+$), 181 (39, $[M-C_6H_{13}]^+$), 168 (100, $[M-C_7H_{14}]^+$).

HRMS (EI): m/z $[M]^+$ calcd for $C_{14}H_{22}N_2O_3$: 266.1630; found: 266.1631.

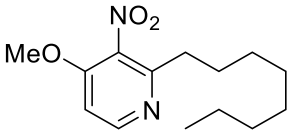

4-methoxy-2-octyl-3-nitropyridine, **4k’**

Yield:
73 mg (0.28 mmol, **14%**) from **1c** (309 mg, 2.00 mmol)

dark orange oil
eluent: cyclohexane/toluene 5:1 to 1:1, then toluene, then toluene/ethyl acetate 5:1

^{1}H NMR (400 MHz, $CDCl_3$): δ = 8.45 (d, *J* = 5.8 Hz, 1H), 6.80 (d, *J* = 5.8 Hz, 1H), 3.91 (s, *J* = 2.2 Hz, 3H), 2.70 – 2.64 (m, 2H), 1.74 – 1.63 (m, 2H), 1.34 – 1.12 (m, 10H), 0.83 (t, *J* = 6.9 Hz, 3H).

^{13}C NMR (100 MHz, $CDCl_3$): δ = 157.0, 154.8, 151.5, 138.5, 105.5, 56.4, 33.5, 31.7, 29.3, 29.2, 29.1, 28.9, 22.6, 14.0.

HRMS (ESI): m/z $[M+H]^+$ calcd for $C_{14}H_{23}N_2O_3$: 267.1709; found: 267.1708.

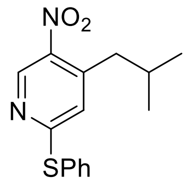

2-thiophenoxy-4-isobutyl-5-nitropyridine, **4l**

Yield:
405 mg (1.40 mmol, **69%**) from **1d** (467 mg, 2.01 mmol)

orange solid, **mp** 47.0 – 50.0 °C
eluent: cyclohexane/toluene 5:1 to 1:1, then toluene, then toluene/ethyl acetate 20:1 to 5:1

^{1}H NMR (400 MHz, $CDCl_3$): δ = 8.91 (s, 1H), 7.59 – 7.53 (m, 2H), 7.48 – 7.41 (m, 3H), 6.65 (s, 1H), 2.65 (d, *J* = 7.1 Hz, 2H), 1.84 – 1.70 (m, 1H), 0.81 (d, *J* = 6.7 Hz, 6H).

^{13}C NMR (100 MHz, $CDCl_3$): δ = 167.2, 146.2, 146.0, 142.6, 135.2, 130.0, 129.8, 128.8, 122.4, 41.3, 28.7, 22.1.

MS (EI): m/z (%): 289 (22, $[M+H]^+$), 288 (56, $[M]^+$), 287 (100, $[M-H]^+$), 199 (40, $[M-C_3H_7NO_2]^+$).

HRMS (EI): m/z $[M]^+$ calcd for $C_{15}H_{16}N_2O_2S$: 288.0932; found: 288.0922.

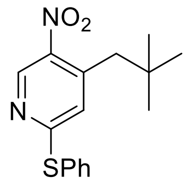

2-thiophenoxy-4-(tert-butylmethyl)-5-nitropyridine, **4m**

Yield:
526 mg (1.73 mmol, **86%**) from **1d** (469 mg, 2.02 mmol)

pale yellow solid, **mp** 60.0 – 62.5 °C
eluent: cyclohexane/toluene 3:1 to 1:1, then toluene, then toluene/ethyl acetate 5:1

^{1}H NMR (400 MHz, $CDCl_3$): δ = 8.81 (s, 1H), 7.59 – 7.52 (m, 2H), 7.47 – 7.39 (m, 3H), 6.55 (s, 1H), 2.79 (s, 2H), 0.73 (s, 9H).

^{13}C NMR (100 MHz, $CDCl_3$): δ = 166.4, 145.6, 143.9, 143.5, 135.3, 130.0, 129.8, 128.8, 123.4, 43.8, 33.2, 28.9.

MS (EI): m/z (%): 303 (33, $[M+H]^+$), 302 (76, $[M]^+$), 301 (100, $[M-H]^+$), 287 (26, $[M-CH_3]^+$), 245 (28, $[M-C_4H_9]^+$), 229 (37, $[M-OC_4H_9]^+$), 199 (25, $[M-C_4H_9NO_2]^+$), 58 (70, $[C_4H_{10}]^+$).

HRMS (EI): m/z $[M]^+$ calcd for $C_{16}H_{18}N_2O_2S$: 302.1089; found: 302.1089.

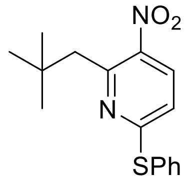

6-thiophenoxy-2-(tert-butylmethyl)-3-nitropyridine, **4m’**

Yield:
28 mg (0.09 mmol, **5%**) from **1d** (469 mg, 2.02 mmol)

yellow oil
eluent: cyclohexane/toluene 3:1 to 1:1, then toluene, then toluene/ethyl acetate 5:1

^{1}H NMR (400 MHz, $CDCl_3$): δ = 7.90 (d, *J* = 8.7 Hz, 1H), 7.62 – 7.56 (m, 2H), 7.49 – 7.42 (m, 3H), 6.76 (d, *J* = 8.7 Hz, 1H), 3.09 (s, 2H), 0.85 (s, 9H).

^{13}C NMR (100 MHz, $CDCl_3$): δ = 165.9, 155.1, 143.9, 135.8, 132.8, 130.0, 129.9, 128.9, 117.8, 46.3, 33.6, 29.5.

MS (EI): m/z (%): 303 (14, $[M+H]^+$), 302 (26, $[M]^+$), 287 (39, $[M-CH_3]^+$), 272 (84, $[M-CH_3-CH_3]^+$), 246 (94, $[M-C_4H_8]^+$), 229 (53, $[M-OC_4H_9]^+$), 57 (100, $[C_4H_9]^+$).

HRMS (EI): m/z $[M]^+$ calcd for $C_{16}H_{18}N_2O_2S$: 302.1089; found: 302.1100.

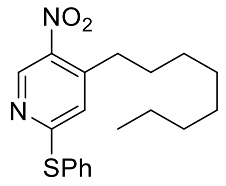

2-thiophenoxy-4-octyl-5-nitropyridine, **4n**

Yield:
442 mg (1.28 mmol, **64%**) from **1d** (467 mg, 2.01 mmol)

orange oil
eluent: cyclohexane/toluene 5:1 to 1:1, then toluene, then toluene/ethyl acetate 20:1 to 5:1

^{1}H NMR (400 MHz, $CDCl_3$): δ = 8.93 (s, 1H), 7.61 – 7.56 (m, 2H), 7.51 – 7.43 (m, 3H), 6.73 (s, 1H), 2.81 – 2.74 (m, 2H), 1.52 – 1.42 (m, 2H), 1.32 – 1.17 (m, 10H), 0.86 (t, *J* = 7.0 Hz, 3H).

^{13}C NMR (100 MHz, $CDCl_3$): δ = 167.5, 147.5, 146.3, 142.5, 135.4, 130.1, 129.9, 128.9, 121.7, 32.7, 31.7, 29.4, 29.3, 29.1, 29.0, 22.6, 14.1.

MS (EI): m/z (%): 345 (25, $[M+H]^+$), 344 (73, $[M]^+$), 343 (100, $[M-H]^+$), 327 (52, $[M-OH]^+$), 245 (100, $[M-C_7H_{15}]^+$), 199 (69, $[M-C_7H_{15}NO_2]^+$).

HRMS (EI): m/z $[M+H]^+$ calcd for $C_{19}H_{25}N_2O_2S$: 345.1637; found: 345.1638.

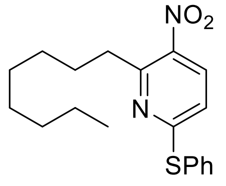

6-thiophenoxy-2-octyl-3-nitropyridine, **4n'**

Yield:
41 mg (0.12 mmol, **6%**) from **1d** (467 mg, 2.01 mmol)

yellowish oil
eluent: cyclohexane/toluene 5:1 to 1:1, then toluene, then toluene/ethyl acetate 20:1 to 5:1

^{1}H NMR (400 MHz, $CDCl_3$): δ = 7.98 (d, *J* = 8.7 Hz, 1H), 7.62 – 7.57 (m, 2H), 7.51 – 7.43 (m, 3H), 6.70 (d, *J* = 8.7 Hz, 1H), 3.07 – 3.01 (m, 2H), 1.72 – 1.61 (m, 2H), 1.40 – 1.19 (m, 10H), 0.87 (t, *J* = 6.9 Hz, 3H).

^{13}C NMR (100 MHz, $CDCl_3$): δ = 167.3, 157.8, 142.1, 135.7, 133.1, 130.1, 130.0, 128.9, 117.6, 36.0, 31.9, 29.5, 29.3, 29.2, 28.5, 22.7, 14.1.

MS (EI): m/z (%): 345 (10, $[M+H]^+$), 344 (26, $[M]^+$), 327 (18, $[M-OH]^+$), 273 (100, $[M-C_5H_{11}]^+$), 229 (72, $[M-OC_7H_{15}]^+$).

HRMS (EI): m/z $[M]^+$ calcd for $C_{19}H_{24}N_2O_2S$: 344.1559; found: 344.1563.

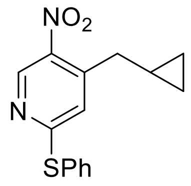

2-thiophenoxy-4-cyclopropylmethyl-5-nitropyridine, **4o**

Yield:
417 mg (1.46 mmol, **73%**) form **1d** (466 mg, 2.01 mmol)

light red solid, **mp** 48.0 – 50.5 °C
eluent: cyclohexane/toluene 3:1 to 1:1, then toluene, then toluene/ethyl acetate 20:1 to 5:1

^{1}H NMR (400 MHz, $CDCl_3$): δ = 8.90 (s, 1H), 7.61 – 7.54 (m, 2H), 7.48 – 7.41 (m, 3H), 7.00 (s, 1H), 2.73 (d, *J* = 7.0 Hz, 2H), 0.80 – 0.69 (m, 1H), 0.41 – 0.35 (m, 2H), 0.07 – 0.00 (m, 2H).

^{13}C NMR (100 MHz, $CDCl_3$): δ = 168.1, 146.8, 145.8, 142.0, 135.3, 130.0, 129.8, 128.8, 120.8, 36.5, 9.1, 4.6.

MS (EI): m/z (%): 287 (25, $[M+H]^+$), 286 (73, $[M]^+$), 285 (100, $[M-H]^+$), 231 (32, $[M-C_4H_7]^+$).

HRMS (EI): m/z $[M]^+$ calcd for $C_{15}H_{14}N_2O_2S$: 286.0776; found: 286.0772.

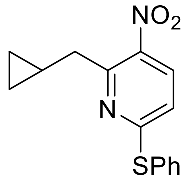

6-thiophenoxy-2-cyclopropylmethyl-3-nitropyridine, **4o'**

Yield:
43 mg (0.15 mmol, **8%**) form **1d** (466 mg, 2.01 mmol)

yellow oil
eluent: cyclohexane/toluene 3:1 to 1:1, then toluene, then toluene/ethyl acetate 20:1 to 5:1

^{1}H NMR (400 MHz, $CDCl_3$): δ = 8.00 (d, *J* = 8.7 Hz, 1H), 7.63 – 7.56 (m, 2H), 7.50 – 7.42 (m, 3H), 6.77 (d, *J* = 8.7 Hz, 1H), 2.96 (d, *J* = 7.0 Hz, 2H), 1.20 – 1.08 (m, 1H), 0.45 – 0.38 (m, 2H), 0.24 – 0.18 (m, 2H).

^{13}C NMR (100 MHz, $CDCl_3$): δ = 167.1, 157.1, 142.0, 135.6, 133.0, 130.0, 129.9, 128.9, 117.9, 40.4, 10.0, 4.5.

MS (EI): m/z (%): 287 (31, $[M+H]^+$), 286 (100, $[M]^+$), 271 (69, $[M-CH_3]^+$), 209 (83, $[M-C_6H_5]^+$).

HRMS (EI): m/z $[M]^+$ calcd for $C_{15}H_{14}N_2O_2S$: 286.0776; found: 286.0777.

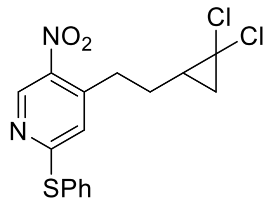

4-(2-(2,2-dichlorocyclopropyl)ethyl)-2-thiophenoxy-5-nitropyridine, **4p**

Yield:
394 mg (1.07 mmol, **53%**) from **1d** (467 mg, 2.01 mmol)

yellow oil
eluent: cyclohexane/toluene 3:1 to 1:1, then toluene, then toluene/ethyl acetate 20:1 to 5:1

^{1}H NMR (400 MHz, $CDCl_3$): δ = 8.99 (s, 1H), 7.61 – 7.56 (m, 2H), 7.51 – 7.43 (m, 3H), 6.76 (s, 1H), 3.09 (ddd, *J* = 13.2, 8.8, 6.1 Hz, 1H), 2.89 (ddd, *J* = 13.2, 9.1, 6.1 Hz, 1H), 1.94 – 1.81 (m, 1H), 1.76 – 1.64 (m, 1H), 1.57 – 1.43 (m, 2H), 1.06 – 1.01 (m, 1H).

^{13}C NMR (100 MHz, $CDCl_3$): δ = 168.3, 146.6, 145.9, 142.1, 135.5, 130.2, 130.0, 128.6, 122.0, 60.8, 32.0, 30.4, 29.9, 26.6.

MS (EI): m/z (%) = 372, 370, 368 (14, 65, 87, $[M]^+$), 371, 369, 367 (28, 89, 100, $[M-H]^+$), 335, 333 (41, 91, $[M-Cl]^+$), 227 (95, $[M-NO_2-C_2HCl_2]^+$).

HRMS (EI): m/z $[M]^+$ calcd for $C_{16}H_{14}O_2N_2S^{35}Cl_2$: 368.0153; found: 368.0150.

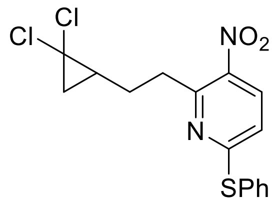

2-(2-(2,2-dichlorocyclopropyl)ethyl)-6-thiophenoxy-3-nitropyridine, **4p’**

Yield:
67 mg (0.18 mmol, **9%**) from **1d** (467 mg, 2.01 mmol)

yellow oil
eluent: cyclohexane/toluene 3:1 to 1:1, then toluene, then toluene/ethyl acetate 20:1 to 5:1

^{1}H NMR (400 MHz, $CDCl_3$): δ = 8.05 (d, *J* = 8.8 Hz, 1H), 7.61 – 7.55 (m, 2H), 7.51 – 7.43 (m, 3H), 6.81 (d, *J* = 8.8 Hz, 1H), 3.32 – 3.18 (m, 2H), 2.07 – 1.96 (m, 1H), 1.91 – 1.81 (m, 1H), 1.67 – 1.49 (m, 2H), 1.07 – 1.00 (m, 1H).

^{13}C NMR (100 MHz, $CDCl_3$): δ = 167.4, 156.0, 142.1, 135.7, 133.2, 130.2, 129.9, 128.6, 118.2, 61.4, 34.6, 30.2, 28.6, 26.7.

MS (EI): m/z (%) = 372, 370, 368 (0, 3, 5, $[M]^+$), 335, 333 (12, 29, $[M-Cl]^+$), 272 (100, $[M-C_2H_2Cl_2]^+$).

HRMS (EI): m/z $[M]^+$ calcd for $C_{16}H_{14}O_2N_2S^{35}Cl_2$: 368.0153; found: 368.0151.

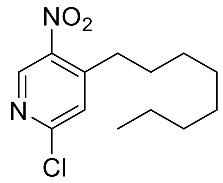

2-chloro-4-octyl-5-nitropyridine, **4q**

Yield:
197 mg (0.73 mmol, **36%**) from **1e** (320 mg, 2.02 mmol)

yellowish oil
eluent: cyclohexane/toluene 3:1 to 1:1, then toluene

^{1}H NMR (400 MHz, $CDCl_3$): δ = 8.87 (s, 1H), 7.30 (s, 1H), 2.92 – 2.85 (m, 2H), 1.66 – 1.55 (m, 2H), 1.41 – 1.17 (m, 10H), 0.84 (t, *J* = 7.0 Hz, 3H).

^{13}C NMR (100 MHz, $CDCl_3$): δ = 155.1, 150.0, 146.2, 144.8, 126.1, 32.4, 31.7, 29.6, 29.4, 29.1, 29.0, 22.6, 14.0.

MS (EI): m/z (%): 272, 270 (2, 4, $[M]^+$), 255, 253 (15, 45, $[M-OH]^+$), 235 (47, $[M-Cl]^+$), 171, 169 (17, 49, $[M-OC_6H_{13}]^+$), 44 (100, $[C_3H_8]^+$).

HRMS (EI): m/z $[M]^+$ calcd for $C_{13}H_{19}N_2O_2{}^{35}Cl$: 270.1135; found: 270.1134.

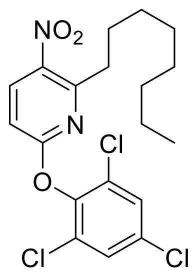

6-(2,4,6-trichlorophenoxy)-2-octyl-3-nitropyridine, **4r**

Yield:
435 mg (1.01 mmol, **50%**) from **1f** (642 mg, 2.01 mmol)

pale yellowish oil
eluent: cyclohexane/toluene 3:1 to 1:2, then toluene

^{1}H NMR (400 MHz, $CDCl_3$): δ = 8.35 (d, *J* = 8.9 Hz, 1H), 7.38 (s, 2H), 6.99 (d, *J* = 8.9 Hz, 1H), 2.92 (t, *J* = 7.3 Hz, 2H), 1.55 – 1.46 (m, 2H), 1.30 – 1.09 (m, 10H), 0.86 (t, *J* = 7.1 Hz, 3H).

^{13}C NMR (100 MHz, $CDCl_3$): δ = 161.7, 157.8, 144.8, 141.8, 137.3, 131.5, 130.1, 128.5, 108.2, 35.0, 31.8, 29.4, 29.1, 29.0, 27.4, 22.7, 14.1.

HRMS (ESI): m/z $[M+H]^+$ calcd for $C_{19}H_{22}N_2O_3{}^{35}Cl_3$: 431.0696; found: 431.0692.

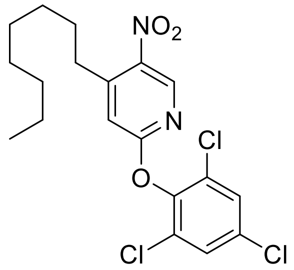

2-(2,4,6-trichlorophenoxy)-4-octyl-5-nitropyridine, **4r'**

Yield:
225 mg (0.52 mmol, **26%**) from **1f** (642 mg, 2.01 mmol)

off-white solid, **mp** 51.5 – 53.0 °C
eluent: cyclohexane/toluene 3:1 to 1:2, then toluene

^{1}H NMR (400 MHz, $CDCl_3$): δ = 8.70 (s, 1H), 7.40 (s, 2H), 7.02 (s, 1H), 3.01 – 2.96 (m, 2H), 1.73 – 1.63 (m, 2H), 1.46 – 1.22 (m, 10H), 0.87 (t, *J* = 7.0 Hz, 3H).

^{13}C NMR (100 MHz, $CDCl_3$): δ = 163.4, 152.0, 145.7, 144.5, 142.5, 131.9, 130.0, 128.8, 111.8, 33.1, 31.8, 29.51, 29.48, 29.2, 29.1, 22.6, 14.1.

HRMS (ESI): m/z $[M+H]^+$ calcd for $C_{19}H_{22}N_2O_3{}^{35}Cl_3$: 431.0696; found: 431.0681.

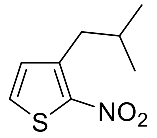

3-isobutyl-2-nitrothiophene, **4s**

Yield:
204 mg (1.10 mmol, **55%**) from **1g** (259 mg, 2.01 mmol)

orange oil
eluent: cyclohexane/toluene 5:1 to 1:2

^{1}H NMR (400 MHz, $CDCl_3$): δ = 7.38 (d, *J* = 5.4 Hz, 1H), 6.87 (d, *J* = 5.4 Hz, 1H), 2.90 (d, *J* = 7.2 Hz, 2H), 2.06 – 1.88 (m, 1H), 0.91 (d, *J* = 6.7 Hz, 6H).

^{13}C NMR (100 MHz, $CDCl_3$): δ = 147.0, 145.4, 131.0, 129.9, 38.2, 29.0, 22.4.

MS (EI): m/z (%): 186 (1, $[M+H]^+$), 185 (5, $[M]^+$), 168 (38, $[M\text{-}OH]^+$), 126 (100, $[M\text{-}OC_3H_7]^+$), 44 (67, $[C_3H_8]^+$).

HRMS (EI): m/z $[M]^+$ calcd for $C_8H_{11}NO_2S$: 185.0511; found: 185.0507.

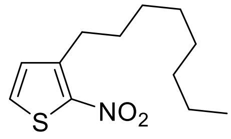

3-octyl-2-nitrothiophene, **4t**

Yield:
279 mg (1.16 mmol, **58%**) from **1g** (259 mg, 2.01 mmol)

yellow oil
eluent: cyclohexane/toluene 20:1 to 4:1

^{1}H NMR (400 MHz, $CDCl_3$): δ = 7.37 (d, *J* = 5.5 Hz, 1H), 6.90 (d, *J* = 5.5 Hz, 1H), 3.02 – 2.97 (m, 2H), 1.66 – 1.57 (m, 2H), 1.39 – 1.17 (m, 10H), 0.84 (t, *J* = 6.9 Hz, 3H).

^{13}C NMR (100 MHz, $CDCl_3$): δ = 146.6, 146.5, 130.2, 130.1, 31.7, 29.6, 29.4, 29.21, 29.19, 29.1, 22.5, 14.0.

MS (EI): m/z (%): 241 (1, $[M]^+$), 224 (45, $[M\text{-}OH]^+$), 97 (100, $[C_7H_{13}]^+$), 44 (55, $[C_3H_8]^+$).

HRMS (EI): m/z $[M]^+$ calcd for $C_{12}H_{19}NO_2S$: 241.1137; found: 241.1134.

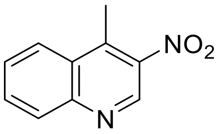

4-methyl-3-nitroquinoline, **4u**

Yield:
147 mg (0.78 mmol, **79%**) from **1h** (172 mg, 0.99 mmol)

white solid, **mp** 115.5 – 116.5 °C
eluent: toluene/ethyl acetate 50:1 to 5:1

^{1}H NMR (400 MHz, $CDCl_3$): δ = 9.16 (s, 1H), 8.15 – 8.10 (m, 2H), 7.82 (ddd, *J* = 8.4, 6.9, 1.4 Hz, 1H), 7.67 (ddd, *J* = 8.4, 6.9, 1.3 Hz, 1H), 2.86 (s, 3H).

^{13}C NMR (100 MHz, $CDCl_3$): δ = 148.3, 144.4, 143.3, 140.1, 131.9, 130.4, 128.4, 126.8, 125.4, 14.2.

MS (EI): m/z (%): 189 (18, $[M+H]^+$), 188 (78, $[M]^+$), 171 (70, $[M\text{-}OH]^+$), 142 (41, $[M\text{-}NO_2]^+$), 115 (100, $[M\text{-}HCN\text{-}NO_2]^+$).

HRMS (EI): m/z $[M]^+$ calcd for $C_{10}H_8N_2O_2$: 188.0586; found: 188.0591.

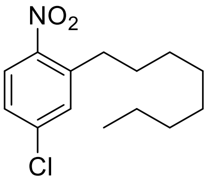

4-chloro-2-octylnitrobenzene, **4v**

Yield:
81 mg (0.30 mmol, **15%**) from **1i** (317 mg, 2.01 mmol)

orange oil
eluent: cyclohexane/toluene 20:1 to 5:1

^{1}H NMR (400 MHz, $CDCl_3$): δ = 7.83 (d, *J* = 8.6 Hz, 1H), 7.32 – 7.25 (m, 2H), 2.86 – 2.81 (m, 2H), 1.65 – 1.55 (m, 2H), 1.40 – 1.19 (m, 10H), 0.88 – 0.83 (t, *J* = 6.9 Hz, 3H).

^{13}C NMR (100 MHz, $CDCl_3$): δ = 147.5, 139.9, 139.0, 131.6, 126.9, 126.2, 33.0, 31.8, 30.5, 29.5, 29.2, 29.1, 22.6, 14.1.

MS (EI): m/z (%): 271, 269 (3, 9, $[M]^+$), 254, 252 (48, 100, $[M\text{-}OH]^+$), 234 (20, $[M\text{-}Cl]^+$), 156, 154 (35, 74, $[M\text{-}OC_7H_{15}]^+$).

HRMS (EI): m/z $[M]^+$ calcd for $C_{14}H_{20}NO_2{}^{35}Cl$: 269.1183; found: 269.1195.

3.5. Characterization of protonated σ^H-adducts

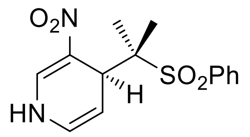

5a

Yield:
266 mg (0.86 mmol, **43%**) from **1a** (251 mg, 2.02 mmol)

orange solid, **mp** 157.0 °C (dec.)
eluent: cyclohexane/ethyl acetate 5:1 to 1:2, then ethyl acetate

^{1}H NMR (400 MHz, DMSO-*d$_6$*): δ = 9.68 (br s, 1H), 8.24 (ddd, *J* = 6.7, 1.6, 0.7 Hz, 1H), 7.89 – 7.84 (m, 2H), 7.82 – 7.76 (m, 1H), 7.71 – 7.65 (m, 2H), 6.56 (ddd, *J* = 7.6, 4.3, 0.7 Hz, 1H), 5.34 (ddd, *J* = 7.6, 6.3, 1.7 Hz, 1H), 4.64 (dd, *J* = 6.3, 1.6 Hz, 1H), 1.12 (s, 3H), 0.90 (s, 3H).

^{13}C NMR (100 MHz, DMSO-*d$_6$*): δ = 140.4, 136.1, 134.6, 130.5, 129.7, 126.8, 117.6, 106.6, 69.3, 36.6, 20.0, 17.4.

HRMS (ESI): m/z [M+Na]$^+$ calcd for $C_{14}H_{16}N_2O_4SNa$: 331.0728; found: 331.0725.

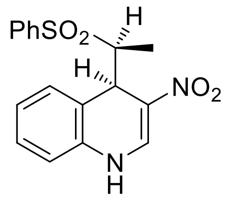

5b

Yield:
117 mg (0.34 mmol, **68%**) from **1h** (87 mg, 0.50 mmol)

yellow solid, **mp** 153.0 °C (dec.)
eluent: toluene/ethyl acetate 10:1 to 1:2, then ethyl acetate

^{1}H NMR (400 MHz, DMSO-*d$_6$*): δ = 10.65 (d, *J* = 6.8 Hz, 1H), 8.37 (dd, *J* = 6.8, 0.6 Hz, 1H), 7.88 – 7.83 (m, 2H), 7.78 – 7.72 (m, 1H), 7.70 – 7.64 (m, 2H), 7.55 (d, *J* = 7.0 Hz, 1H), 7.34 – 7.28 (m, 1H), 7.21 – 7.09 (m, 2H), 5.34 (s, 1H), 3.40 (qd, *J* = 7.1, 1.5 Hz, 1H), 0.83 (d, *J* = 7.1 Hz, 3H).

^{13}C NMR (100 MHz, DMSO-*d$_6$*): δ = 140.2, 139.2, 136.8, 134.4, 131.8, 130.0, 129.1, 128.5, 125.2, 119.9, 119.3, 117.1, 61.7, 36.1, 9.2.

MS (EI): m/z (%): 344 (5, [M]$^+$), 327 (2, [M-OH]$^+$), 202 (14, [M-$PhSO_2H$]$^+$), 175 (100, [M-$PhSO_2C_2H_4$]$^+$), 129 (56, [M-$PhSO_2C_2H_4$-NO_2]$^+$).

HRMS (EI): m/z [M]$^+$ calcd for $C_{17}H_{16}N_2O_4S$: 344.0831; found: 344.0840.

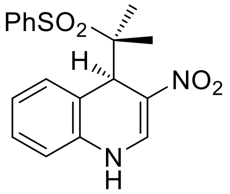

5c

Yield:
19 mg (0.05 mmol, **11%**) from **1h** (87 mg, 0.50 mmol)

yellow oil
eluent: toluene/ethyl acetate 20:1 to 1:1, then ethyl acetate

^{1}H NMR (400 MHz, CDCl$_3$): δ = 8.46 (s, 2H), 7.85 – 7.79 (m, 2H), 7.64 – 7.57 (m, 2H), 7.54 – 7.46 (m, 2H), 7.25 – 7.20 (m, 1H), 7.15 (td, J = 7.5, 1.2 Hz, 1H), 7.06 (d, J = 7.9 Hz, 1H), 5.55 (s, 1H), 1.06 (s, 3H), 0.96 (s, 3H).

^{13}C NMR (100 MHz, CDCl$_3$): δ = 139.3, 136.9, 136.4, 133.8, 132.2, 130.4, 128.8, 128.3, 124.8, 120.2, 119.2, 116.3, 68.6, 40.0, 20.4, 20.1.

HRMS (ESI): m/z $[M+Na]^+$ calcd for $C_{18}H_{18}N_2O_4SNa$: 381.0885; found: 381.0874.

For other components of reaction mixtures, from which adducts **5a-c** were isolated, see table at S-24 (entries 26, 27 and 29).

3.6. Characterization of side products

Alkylation of less electrophilic nitroarene **1i** resulted in the formation of the alkylated product (**4v**, 15%) along with two side products – dimer (**6a**, 39%) and ONSH product (**10**, 10%). Moreover substrate **1i** was recovered in 28%.

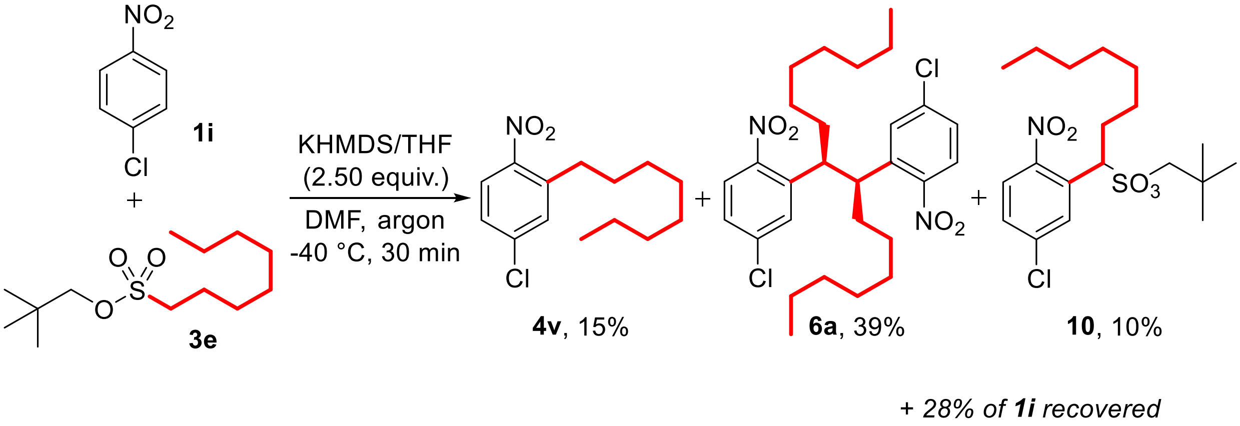

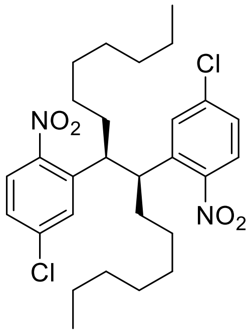

6a

Yield:
208 mg (0.39 mmol, **39%**) from **1i** (317 mg, 2.01 mmol)

orange oil
eluent: cyclohexane/toluene 20:1 to 1:1, then toluene, then toluene/ethyl acetate 50:1 to 20:1

^{1}H NMR (400 MHz, CDCl$_3$): δ = 7.39 (d, *J* = 8.7 Hz, 2H), 7.27 (d, *J* = 2.2 Hz, 2H), 7.10 (dd, *J* = 8.7, 2.2 Hz, 2H), 3.57 (s, 2H), 2.12 – 2.00 (m, 2H), 1.64 – 1.49 (m, 2H), 1.30 – 1.12 (m, 18H), 1.02 – 0.90 (m, 2H), 0.83 (t, *J* = 7.0 Hz, 6H).

^{13}C NMR (100 MHz, CDCl$_3$): δ = 149.0, 140.0, 139.1, 128.6, 127.2, 125.1, 45.1, 34.7, 31.7, 29.6, 29.0, 27.1, 22.5, 14.0.

HRMS (ESI): m/z $[M+Na]^+$ calcd for $C_{28}H_{38}N_2O_4{}^{35}Cl_2Na$: 559.2106; found: 559.2111.

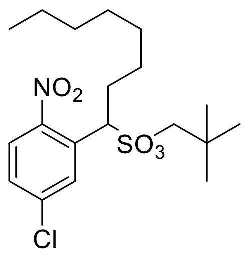

10

Yield:
82 mg (0.20 mmol, **10%**) from **1i** (317 mg, 2.01 mmol)

orange oil
eluent: cyclohexane/toluene 20:1 to 1:1, then toluene, then toluene/ethyl acetate 50:1 to 20:1

^{1}H NMR (400 MHz, CDCl$_3$): δ = 7.89 (d, *J* = 8.8 Hz, 1H), 7.77 (d, *J* = 2.2 Hz, 1H), 7.48 (dd, *J* = 8.8, 2.2 Hz, 1H), 5.35 (dd, *J* = 10.6, 4.4 Hz, 1H), 3.80 (d, *J* = 9.1 Hz, 1H), 3.73 (d, *J* = 9.1 Hz, 1H), 2.45 – 2.34 (m, 1H), 2.19 – 2.06 (m, 1H), 1.40 – 1.13 (m, 10H), 0.86 – 0.81 (m, 12H).

^{13}C NMR (100 MHz, CDCl$_3$): δ = 148.8, 139.8, 130.0, 129.79, 129.75, 126.5, 79.4, 58.7, 31.7, 31.6, 30.3, 28.9, 28.8, 26.3, 25.9, 22.5, 14.0.

HRMS (ESI): m/z [M+Na]$^+$ calcd for $C_{19}H_{30}NO_5S^{35}ClNa$: 442.1431; found: 442.1429.

From reaction of **1i** with **3b** under standard conditions we isolated dimer **6b**:

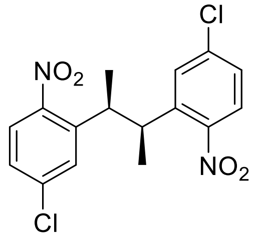

6b

Yield:
85 mg (0.23 mmol, **23%**) from **1i** (317 mg, 2.01 mmol)

white crystals, **mp** 153.0 °C (dec.)
eluent: cyclohexane/toluene 20:1 to 1:1, then toluene, then toluene/ethyl acetate 20:1 to 1:1

^{1}H NMR (400 MHz, CDCl$_3$): δ = 7.43 (d, *J* = 8.7 Hz, 2H), 7.25 (d, *J* = 2.2 Hz, 2H), 7.12 (dd, *J* = 8.7, 2.2 Hz, 2H), 3.63 – 3.53 (m, 2H), 1.45 – 1.42 (m, 6H).

^{13}C NMR (100 MHz, CDCl$_3$): δ = 147.9, 141.3, 139.2, 128.2, 127.3, 125.2, 40.0, 20.4.

HRMS (ESI): m/z [M+Na]$^+$ calcd for $C_{16}H_{14}N_2O_4{}^{35}Cl_2Na$: 391.0228; found: 391.0229.

Formation of products **6a** and **10** requires formal oxidation step, therefore reaction of **1i** with **3e** was repeated with excess of **1i**, which can serve as an oxidant. Surprisingly, in this experiment yields of alkylated nitroarene (**4v**, 15%) and its dimer (**6a**, 39%) remained unchanged, while yield of ONSH product (**10**) significantly increased (44% versus 10%).

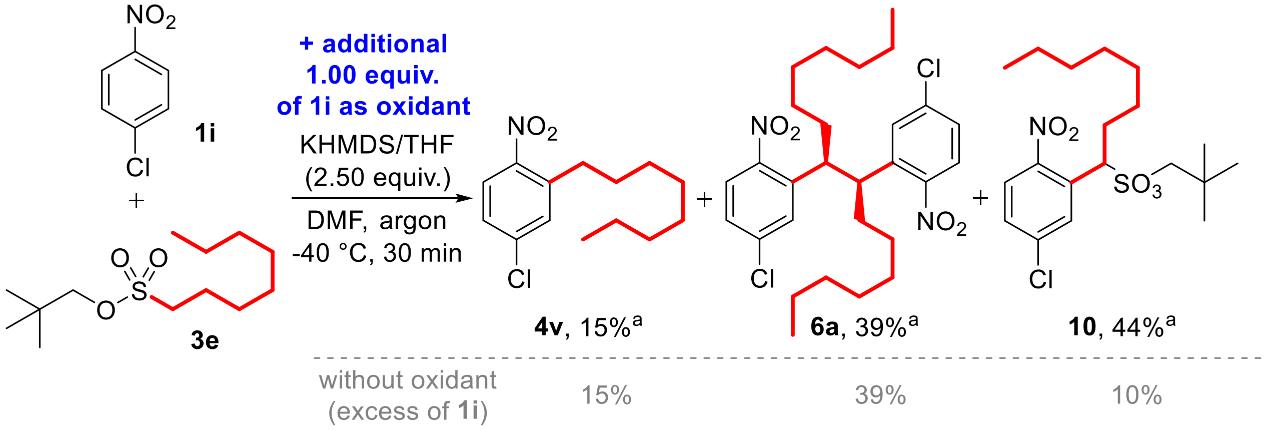

[a] - yield calculated on 1.00 equiv. of **1i**

To reveal mechanism of the dimerization process (**4v**→**6a**) reaction of **1i** with **3e** was repeated with 1.50 equiv. of TEMPO, as a radical scavenger. Again, surprisingly yield of dimer (**6a**, 39%) remained unchanged, while yield of the alkylated product (**4v**) slightly decreased (10% versus 15%), and yield of ONSH product (**10**) slightly increased (16% versus 10%).

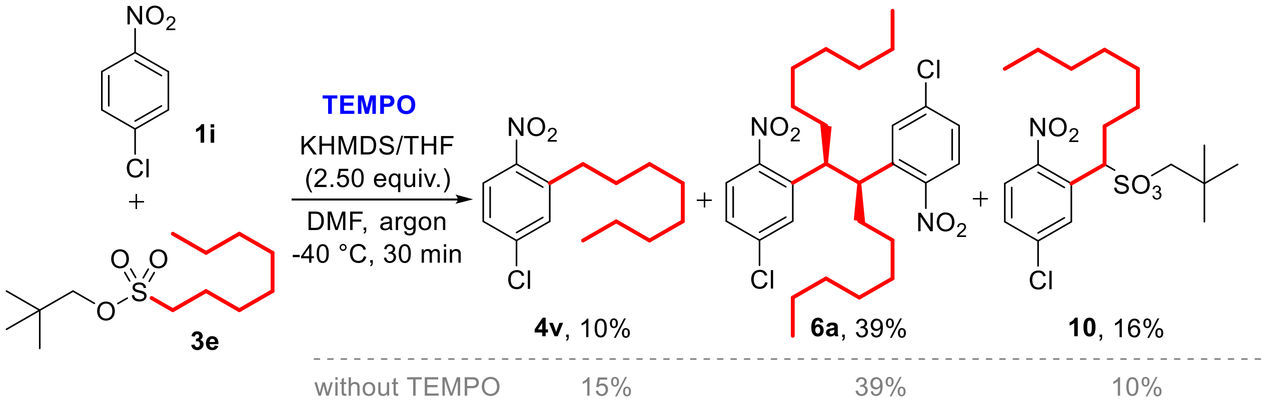

Configuration of dimer 6a

^{1}H NMR spectra measured for **6a** and **6b** (structure of **6b** confirmed by X-ray) are very similar, confirming that the same diastereoisomer was obtained, as a main product:

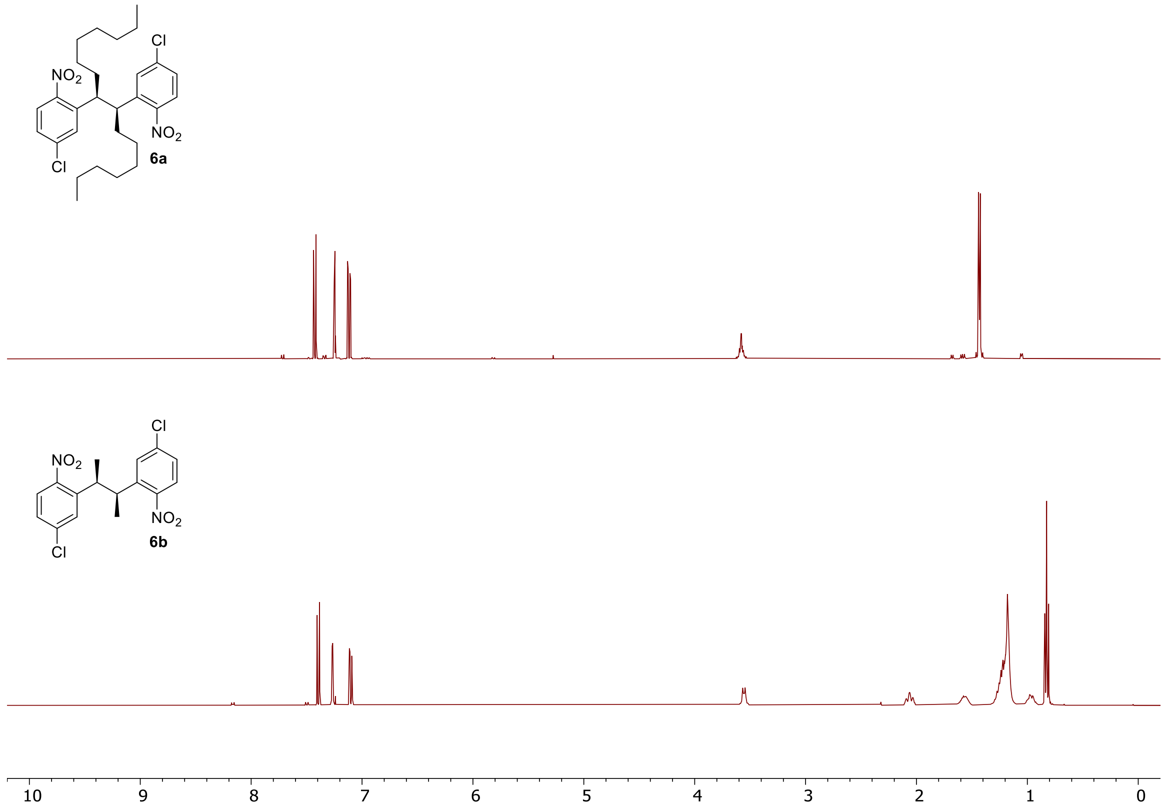

Especially, aromatic signals in 7.50 – 7.00 ppm range vary less than 0.03 ppm:

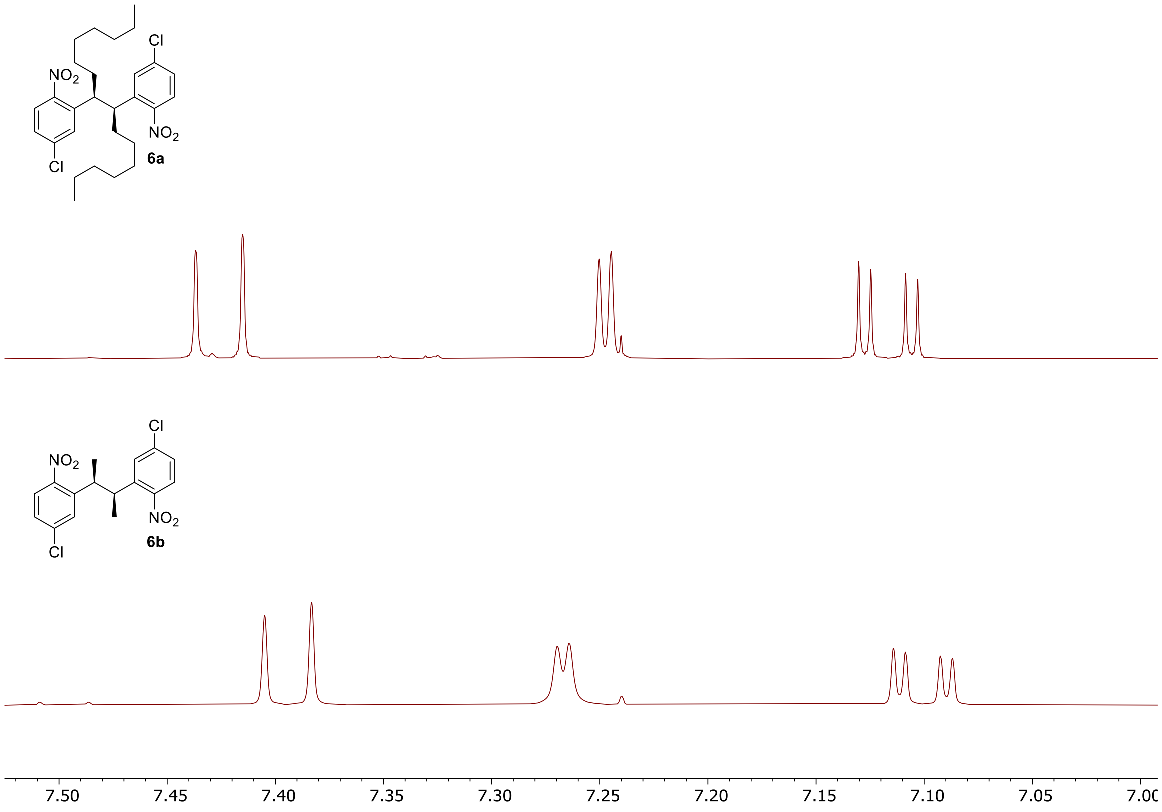

Second diastereoizomer of dimer

After column chromatography we observed small amount (<5% yield) of minor dimeric product (isomer) inseparable from **6b**. Stacked ^{1}H NMR spectra for pure (**6b**) and its mixture with the minor dimeric product are shown below (the mixture represents one fraction after chromatography, in which the second isomer was observed):

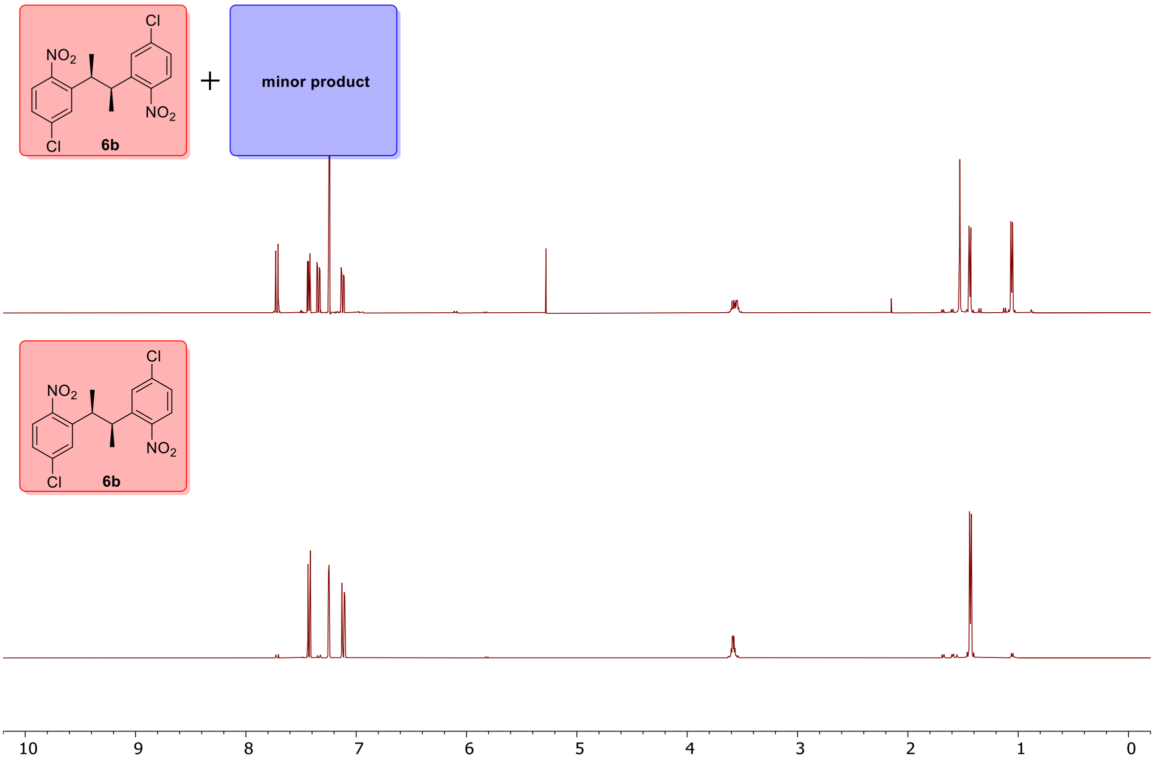

In 7.80 – 7.00 ppm range set of peaks (7.43, d; 7.25, d; 7.12, dd) arising from (**6b**) and set of peaks of minor dimeric product shifted ca. 0.25 ppm downfield (7.71, d; 7.44, d; 7.34; dd) were observed:

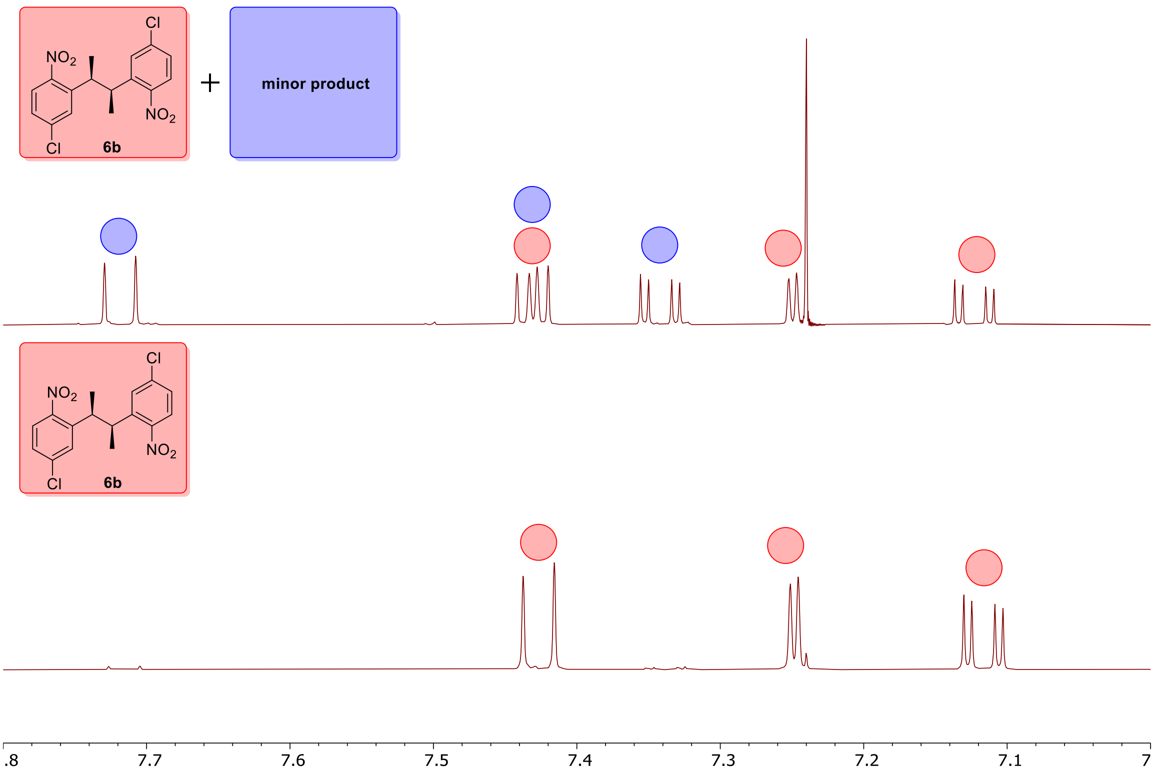

Multiplet around 3.55 ppm arising from minor dimeric product is similar to peak from benzyl protons of **6b**:

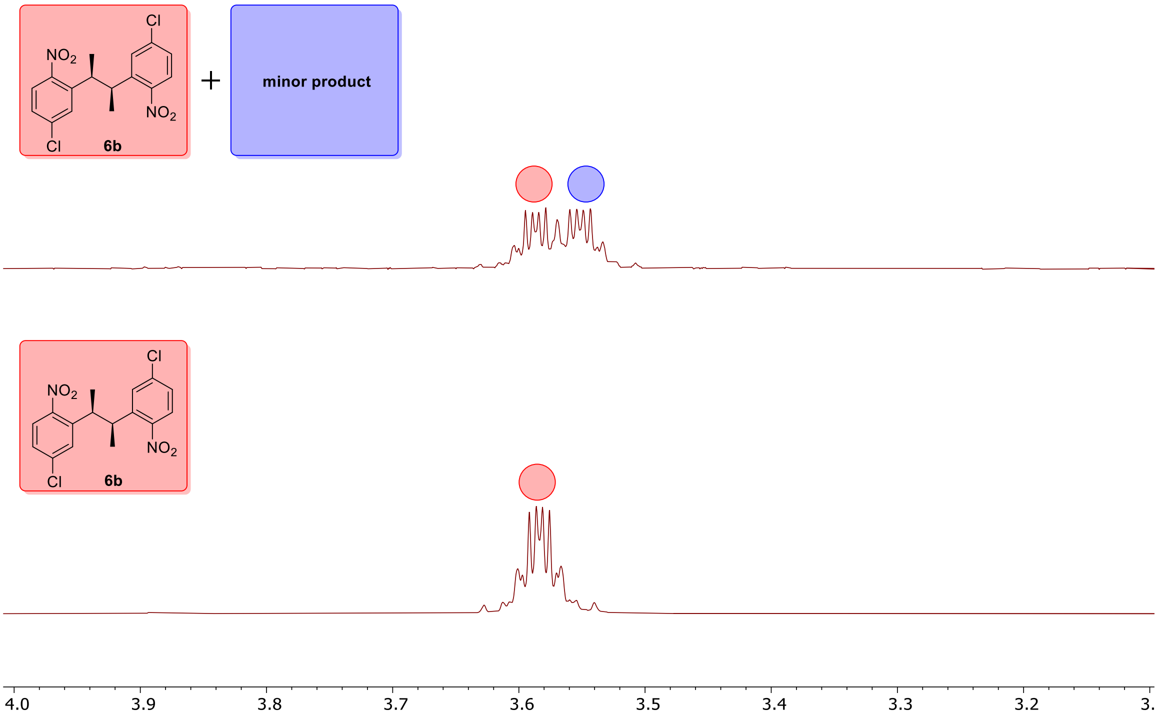

Signal from methyl groups with δ = 1.43 ppm from (**6b**) and similar peak with δ = 1.06 ppm from minor product were also observed:

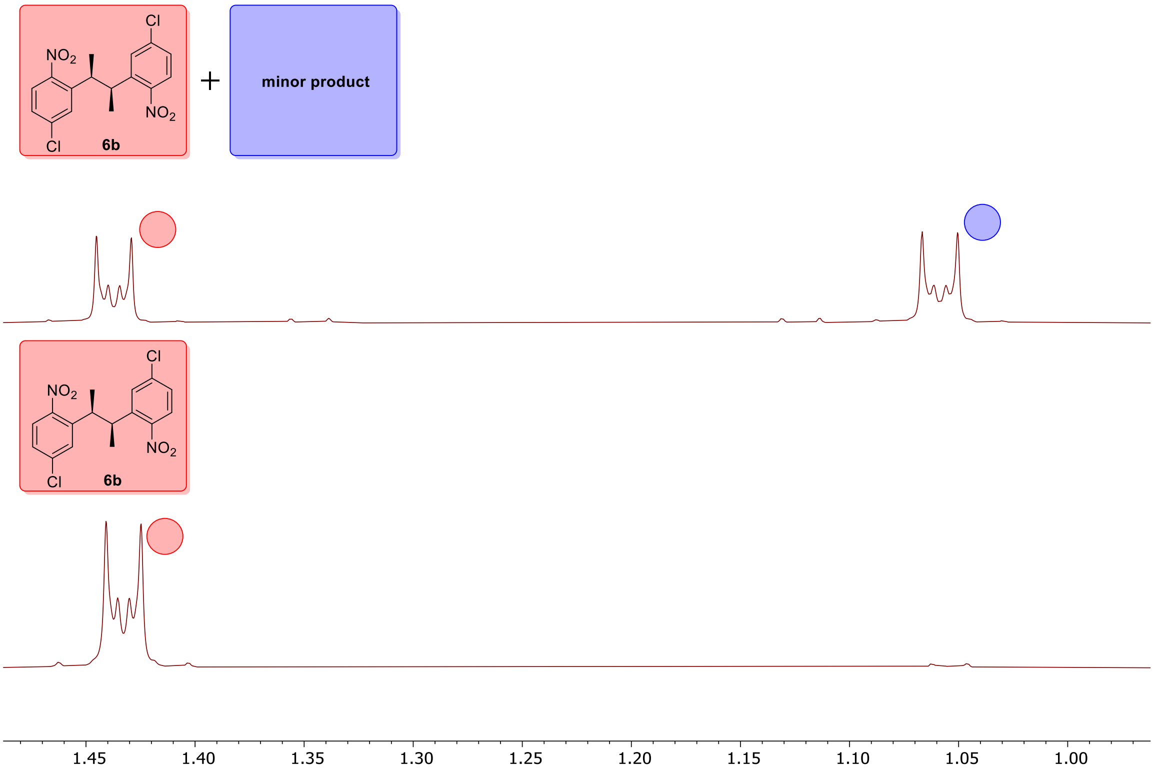

Based on the spectroscopic data we assume that the dimerization process gives two diastereoisomers of product, and one of them (characterized with X-ray) predominates in the mixture. The same conclusion applies to dimerization of octylated chloronitrobenzene **4v**.

In reaction of **1a** with **3a** under standard reaction conditions dimer **6c** was isolated, as a byproduct. For studies of dimerization of isolated sample of **4a**, see next chapter.

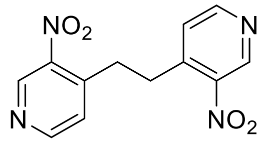

6c

Yield:
67 mg (0.24 mmol, **24%**) from **1a** (251 mg, 2.02 mmol) and **3a**

dark yellow solid, **mp** 164.0 °C (dec.)
eluent: cyclohexane/ethyl acetate 5:1 to 1:2, then ethyl acetate, then dichloromethane/methanol 50:1 to 10:1

^{1}H NMR (400 MHz, $CDCl_3$): δ = 9.19 (s, 2H), 8.73 (d, *J* = 5.0 Hz, 2H), 7.37 (d, *J* = 5.0 Hz, 2H), 3.29 (s, 4H).

^{13}C NMR (100 MHz, $CDCl_3$): δ = 153.7, 146.3, 145.3, 144.1, 126.2, 32.9.

HRMS (ESI): m/z $[M+H]^+$ calcd for $C_{12}H_{11}N_4O_4$: 275.0780; found: 275.0766.

In reactions of **1a** with **2e** and with **3e** under standard reaction conditions dimer **6d** was isolated, as a byproduct. For studies of dimerization of isolated sample of **4e**, see next chapter.

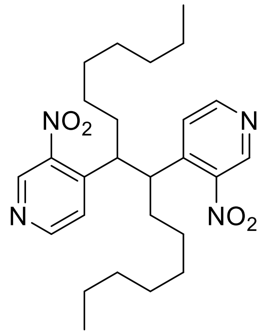

6d

Yield:
21 mg (0.04 mmol, **4%**) from **1a** (248 mg, 2.00 mmol) and **3e**
7 mg (0.02 mmol, **2%**) from **1a** (251 mg, 2.02 mmol) and **2e**

orange oil
eluent: toluene/ethyl acetate 100:1 to 3:1, then ethyl acetate

^{1}H NMR (400 MHz, $CDCl_3$): δ = 8.70 (s, 2H), 8.55 (d, *J* = 5.3 Hz, 2H), 7.29 (d, *J* = 5.3 Hz, 2H), 3.75 – 3.64 (m, 2H), 2.15 – 2.04 (m, 2H), 1.66 – 1.53 (m, 2H), 1.32 – 1.10 (m, 18H), 0.96 – 0.86 (m, 2H), 0.82 (t, *J* = 7.0 Hz, 6H).

^{13}C NMR (100 MHz, $CDCl_3$): δ = 153.0, 147.2, 146.8, 145.0, 122.5, 44.1, 34.7, 31.7, 29.6, 28.9, 26.9, 22.5, 14.0.

HRMS (ESI): m/z $[M+Na]^+$ calcd for $C_{26}H_{38}N_4O_4Na$: 493.2791; found: 493.2793.

4. Further studies

4.1. Stability of the alkylated products anions

In order to determine stability of the alkylated nitroarenes anions we tested 4-chloro-2-octylnitrobenzene (**4v**), 3-nitro-4-octylpyridine (**4e**), 4-methyl-3-nitropyridine (**4a**), and 4-isobutyl-3-nitropyridine (**4c**) under standard reaction conditions (General Procedure; DMF, -40 °C, 30 min). Yields of dimers and recovered alkylated nitroarenes are given below.

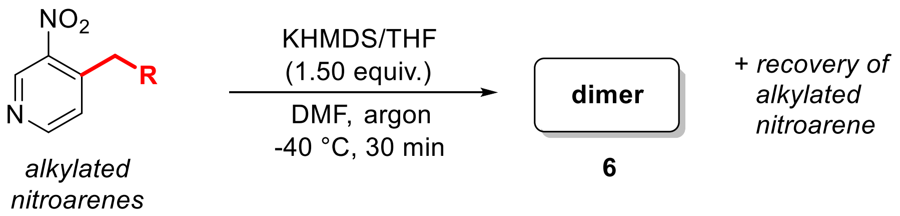

4

A 30 mL Schlenk flask was charged with alkylated alkylated nitroarene (**4**) (1.00 mmol) and flushed with argon. Dry DMF (3 mL) was added, and after dissolution of **4** mixture was cooled to -40 °C. To the stirred reaction mixture KHMDS (1.50 mmol, solution in THF*) was added rapidly in one portion. After 30 min reaction mixture was quenched with NH_4Cl_{aq} (5 mL, 10% w/v). Mixture was transferred to separatory funnel, NH_4Cl_{aq} (50 mL, 10% w/v) was added, mixture was extracted with ethyl acetate (3 × 50 mL), combined organic phases were washed with $LiCl_{aq}$ (50 mL, 1.0 M), H_2O (50 mL), brine (50 mL), and dried over anhydrous $MgSO_4$. Mixture was filtered, evaporated and separated with column chromatography.

* - for concentrations of commercially-available solutions of KHMDS in THF used in this study see the General Information

For entries 1 and 2 crude reaction mixtures after quench were analyzed by ^{1}H NMR.

entry	alkylated nitroarene	alkylated nitroarene recovery	dimer yield
1	**4v**	34% [a]	**6a**, 66%[a]
2	**4e**	95%[a]	**6d**, 5%[a]
3[b]	**4a**	36%	**6c**, 39%
4[b]	**4c**	97%	-

a – ^{1}H NMR conversions
b – 1.00 equiv. of KHMDS/THF was used

4.2. Experiment with radical scavenger (TEMPO)

In order to exclude radical pathway of the alkylation reaction, experiment with 1.5 equiv. of TEMPO (2,2,6,6-Tetramethylpiperidine 1-oxyl) was carried out according to the General Procedure.

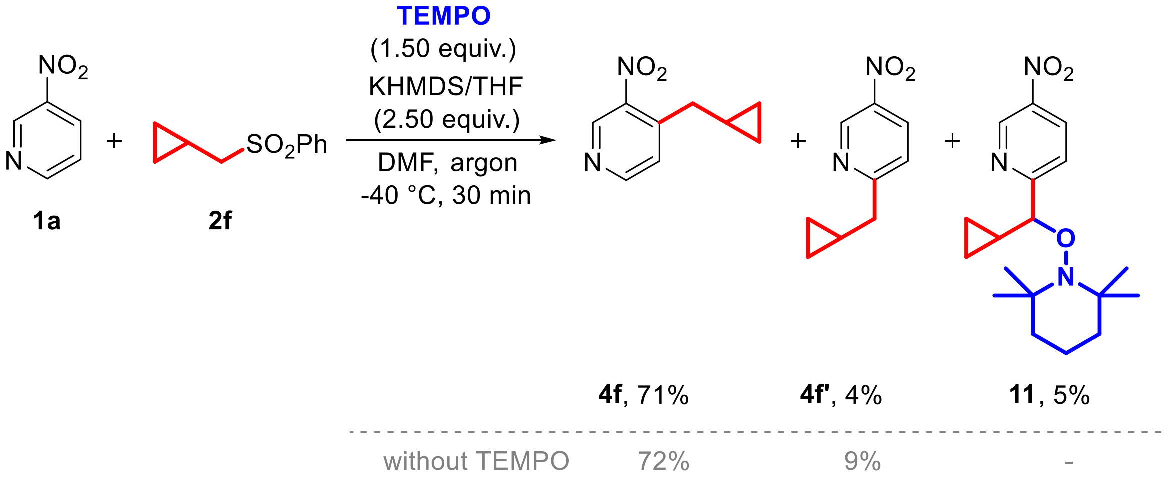

Yield of main product, 4-isobutyl-3-nitropyridine (**4f**) remained unchanged, compared to the reaction without TEMPO. Yield of 2-isobutyl-5-nitropyridine (**4f'**) decreased by 5% (from 9% to 4%), and 5% of the corresponding adduct of minor isomer with TEMPO (**11**) was formed.

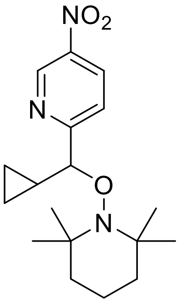

11

Yield:
39 mg (0.12 mmol, **5%**) from **1a** (248 mg, 2.00 mmol)

light yellow solid, **mp** 54.5 – 56.5 °C
eluent: toluene/ethyl acetate 50:1 to 1:1, then ethyl acetate

^{1}H NMR (400 MHz, $CDCl_3$): δ = 9.32 (d, *J* = 2.6 Hz, 1H), 8.44 (dd, *J* = 8.6, 2.6 Hz, 1H), 7.62 (d, *J* = 8.6 Hz, 1H), 4.41 (d, *J* = 8.3 Hz, 1H), 1.62 – 0.90 (m, 18H), 0.78 – 0.67 (m, 1H), 0.67 – 0.54 (m, 3H), 0.40 – 0.31 (m, 1H), 0.30 – 0.19 (m, 1H).

^{13}C NMR (100 MHz, $CDCl_3$): δ = 169.3, 143.8, 142.9, 131.0, 121.8, 90.8, 60.0, 40.2, 34.4, 33.8, 20.4, 20.1, 17.0, 16.9, 7.5, 1.8.

HRMS (ESI): m/z $[M+H]^+$ calcd for $C_{18}H_{28}N_3O_3$: 334.2125; found: 334.2121.

5. One-pot alkylation-fluorination of nitroarenes

Reaction was performed according to the General Procedure. After 30 min solution of Selectfluor in DMF was added and stirred for additional 60 min, before quenching with NH_4Cl_{aq}.[22]

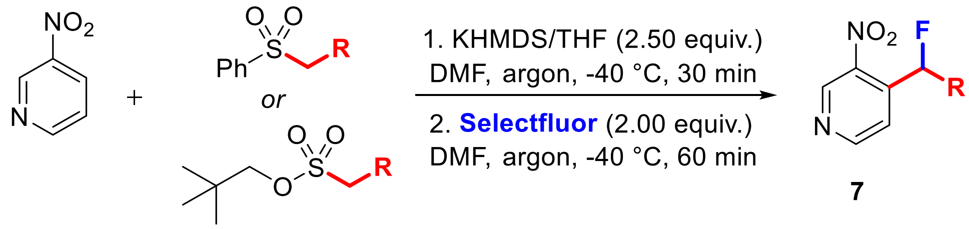

7

nitroarene

1

alkyl sulfone
or sulfonate

2 or **3**

A 30 mL Schlenk flask was charged with nitroarene (**1**, 1.00 mmol), carbanion precursor (**2** or **3**, 1.20 mmol), and flushed with argon. Dry DMF (3 mL) was added and after dissolution of substrates mixture was cooled to -40 °C. To the stirred reaction mixture KHMDS (2.50 mmol, solution in THF*) was added rapidly in one portion. After 30 min solution of Selectfluor (2.00 mmol) in dry DMF (3 mL) was slowly added, and after additional 1 h reaction mixture was quenched with NH_4Cl_{aq} (10 mL, 10% w/v). Mixture was transferred to separatory funnel, NH_4Cl_{aq} (50 mL, 10% w/v) was added, mixture was extracted with ethyl acetate (3 × 50 mL), combined organic phases were washed with $LiCl_{aq}$ (50 mL, 1.0 M), H_2O (50 mL), brine (50 mL), and dried over anhydrous $MgSO_4$. Mixture was filtered, evaporated and separated with column chromatography.

* - for concentrations of commercially-available solutions of KHMDS in THF used in this study see the General Information

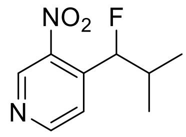

7a

Yield:
102 mg (0.52 mmol, **50%**) from **1a** (129 mg, 1.04 mmol) and **3c**

orange oil
eluent: toluene/ethyl acetate 100:1 to 1:1, then ethyl acetate

^{1}H NMR (400 MHz, CDCl$_3$): δ = 9.23 (s, 1H), 8.81 (d, *J* = 5.2 Hz, 1H), 7.60 (d, *J* = 5.2 Hz, 1H), 6.09 (dd, *J* = 49.0, 3.2 Hz, 1H), 2.15 – 1.96 (m, 1H), 1.11 (d, *J* = 7.0 Hz, 3H), 0.81 (dd, *J* = 7.0, 1.0 Hz, 3H).

^{13}C NMR (100 MHz, CDCl$_3$): δ = 153.7 (d, *J* = 2.1 Hz), 146.1 (d, *J* = 1.5 Hz), 144.8 (d, *J* = 23.2 Hz), 128.7 (d, *J* = 70.6 Hz), 121.7 (d, *J* = 14.3 Hz), 92.6 (d, *J* = 179.1 Hz), 33.1 (d, *J* = 21.8 Hz), 19.4 (d, *J* = 3.3 Hz), 14.9 (d, *J* = 6.1 Hz).

^{19}F NMR (376 MHz, CDCl$_3$): δ = -198.2 (dd, *J* = 49.0, 28.4 Hz).

HRMS (ESI): m/z $[M+H]^+$ calcd for $C_9H_{12}N_2O_2F$: 199.0883; found: 199.0889.

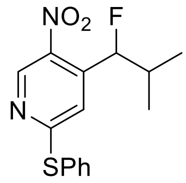

7b

Yield:
343 mg (1.12 mmol, **56%**) from **1d** (468 mg, 2.02 mmol) and **3c**

yellow oil
eluent: cyclohexane/toluene 5:1 to 1:2, then toluene, then toluene/ethyl acetate 50:1 to 5:1

^{1}H NMR (400 MHz, CDCl$_3$): δ = 9.24 (s, 1H), 7.78 – 7.71 (m, 2H), 7.68 – 7.57 (m, 3H), 7.24 (s, 1H), 6.21 (dd, *J* = 49.1, 2.7 Hz, 1H), 2.26 – 2.06 (m, 1H), 1.26 (d, *J* = 6.9 Hz, 3H), 0.88 (d, *J* = 6.9 Hz, 3H).

^{13}C NMR (100 MHz, CDCl$_3$): δ = 169.3 (d, *J* = 2.2 Hz), 146.6 (d, *J* = 1.7 Hz), 145.0 (d, *J* = 22.4 Hz), 138.9 (d, *J* = 4.7 Hz), 135.3, 130.3, 129.9, 128.4, 117.5 (d, *J* = 16.5 Hz), 92.6 (d, *J* = 180.0 Hz), 32.8 (d, *J* = 22.0 Hz), 19.5 (d, *J* = 2.7 Hz), 14.5 (d, *J* = 6.3 Hz).

^{19}F NMR (376 MHz, CDCl$_3$): δ = -199.3 (dd, *J* = 49.1, 29.7 Hz).

MS (EI): m/z (%): 306 (65, $[M]^+$), 305 (100, $[M-H]^+$), 217 (29, $[M-C_3H_7NO_2]^+$).

HRMS (EI): m/z $[M]^+$ calcd for $C_{15}H_{15}N_2O_2FS$: 306.0838; found: 306.0824.

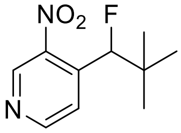

7c

Yield:
218 mg (1.03 mmol, **51%**) from **1a** (249 mg, 2.01 mmol) and **2d**

yellowish oil
eluent: toluene, then toluene/ethyl acetate 50:1 to 20:1, then ethyl acetate

^{1}H NMR (400 MHz, CDCl$_3$): δ = 9.13 (s, 1H), 8.82 (d, *J* = 5.2 Hz, 1H), 7.59 (d, *J* = 5.2 Hz, 1H), 6.23 (d, *J* = 46.7 Hz, 1H), 0.91 (d, *J* = 1.5 Hz, 9H).

^{13}C NMR (100 MHz, CDCl$_3$): δ = 153.0 (d, *J* = 1.5 Hz), 145.5 (d, *J* = 1.3 Hz), 144.10, 141.7 (d, *J* = 24.4 Hz), 122.8 (d, *J* = 12.5 Hz), 93.3 (d, *J* = 179.6 Hz), 36.8 (d, *J* = 21.1 Hz), 24.8 (d, *J* = 4.6 Hz).

^{19}F NMR (376 MHz, CDCl$_3$): δ = -189.5 (d, *J* = 46.7 Hz).

HRMS (ESI): m/z $[M+H]^+$ calcd for $C_{10}H_{14}N_2O_2F$: 213.1039; found: 213.1037.

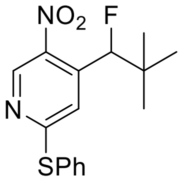

7d

Yield:
390 mg (1.22 mmol, **61%**) form **1d** (466 mg, 2.01 mmol) and **2d**

light yellow solid, **mp** 46.0 – 48.0 °C
eluent: cyclohexane/toluene 2:1 to 1:2, then toluene, then toluene/ethyl acetate 50:1 to 20:1

^{1}H NMR (400 MHz, CDCl$_3$): δ = 8.96 (s, 1H), 7.62 – 7.55 (m, 2H), 7.52 – 7.43 (m, 3H), 6.98 (s, 1H), 6.20 (d, *J* = 46.9 Hz, 1H), 0.80 (d, *J* = 1.7 Hz, 9H).

^{13}C NMR (100 MHz, CDCl$_3$): δ = 168.5 (d, *J* = 1.4 Hz), 145.9 (d, *J* = 1.4 Hz), 142.1 (d, *J* = 23.5 Hz), 140.6 (d, *J* = 4.9 Hz), 135.4, 130.3, 130.0, 128.5, 118.7 (d, *J* = 13.7 Hz), 93.1 (d, *J* = 179.9 Hz), 36.8 (d, *J* = 21.2 Hz), 24.7 (d, *J* = 4.6 Hz).

^{19}F NMR (376 MHz, CDCl$_3$): δ = -189.0 (d, *J* = 47.2 Hz).

MS (EI): m/z (%) = 321 (29, $[M+H]^+$), 320 (82, $[M]^+$), 319 (100, $[M-H]^+$), 247 (32, $[M-OC_4H_9]^+$), 186 (47, $[M-C_5H_9FNO_2]^+$), 58 (90, $[C_4H_{10}]^+$).

HRMS (EI): m/z $[M]^+$ calcd for $C_{16}H_{17}N_2O_2FS$: 320.0995; found: 320.0981.

6. Postsynthetic transformations of the alkylated pyridines

6.1. Reduction of the NO_2 group with subsequent acylation

Reduction of the NO_2 group was performed using H_2 with Pd/C in MeOH. Crude product was acylated with Ac_2O.

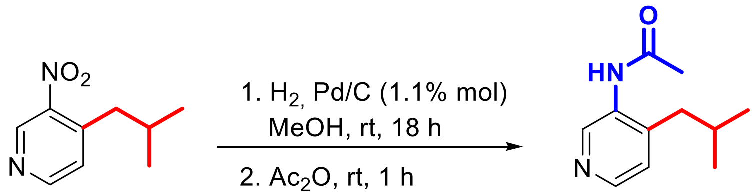

4c **8**, 98%

A 25 mL round-bottom flask was charged with 4-isobutyl-3-nitropyridine (**4c**, 181 mg, 1.00 mmol, 1.00 equiv.), Pd/C (12 mg, 10% w/w, 0.01 mmol, 1.1 mol%), evacuated, and supplied with H_2 balloon. MeOH (4 mL) was added, and reaction was stirred at 1000 rpm. After 18 h reaction mixture was filtered through a pad of Celite and concentrated. Crude product was placed in 25 mL round-bottom flask, acetic anhydride (4 mL) was added, and reaction mixture was stirred for 1 h. Then, reaction mixture was poured on H_2O (50 mL) cooled in ice/water bath (2-3 °C), and K_2CO_3 (12.5 g) was added portionwise to pH ca. 10. Mixture was transferred to separatory funnel, extracted with dichloromethane (3 × 50 mL), combined organic phases were washed with brine (50 mL), and dried over anhydrous $MgSO_4$. Mixture was filtered, evaporated and dried under vacuum yielding **8** (190 mg, 0.99 mmol, **98%**) as an off-white solid.

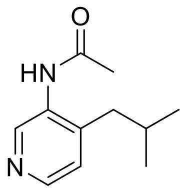

8

Yield:
189.7 mg (0.987 mmol, **98%**) from **4c** (180.8 mg, 1.003 mmol)

off-white solid, **mp** 97.0 – 99.0 °C

^{1}H NMR (400 MHz, $CDCl_3$): δ = 8.64 (s, 1H), 8.54 (s, 1H), 8.18 (d, *J* = 5.0 Hz, 1H), 7.00 (d, *J* = 5.0 Hz, 1H), 2.35 (d, *J* = 7.2 Hz, 2H), 2.05 (s, 3H), 1.85 – 1.72 (m, 1H), 0.79 (d, *J* = 6.7 Hz, 6H).

^{13}C NMR (100 MHz, $CDCl_3$): δ = 169.5, 147.0, 146.0, 144.7, 132.8, 124.9, 39.7, 28.3, 23.3, 22.2.

MS (EI): m/z (%): 192 (75, $[M]^+$), 177 (29, $[M-CH_3]^+$), 150 (100, $[M-C_3H_6]^+$), 135 (56, $[M-C_4H_9]^+$), 107 (94, $[M-C_3H_6-CH_3CO]^+$).

HRMS (EI): m/z $[M]^+$ calcd for $C_{11}H_{16}N_2O$: 192.1263; found: 192.1262.

6.2. Synthesis of azaindole under Bartoli conditions

Transformation of (**4q**) into 4-azaindole (**9**) was performed according to the literature procedure.[23]

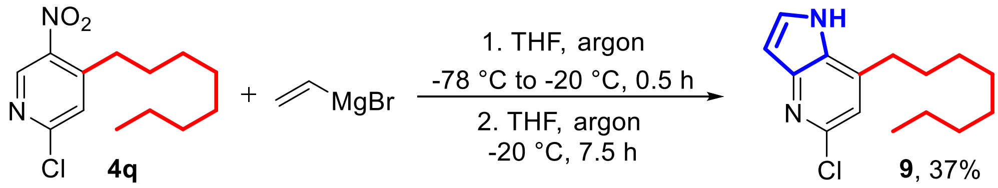

A 30 mL Schlenk flask was charged with 2-chloro-4-octyl-5-nitropyridine (**4q**) (240 mg, 0.89 mmol, 1.00 equiv.), and flushed with argon. Dry THF (5.5 mL) was added and after dissolution of substrate mixture was cooled to -78 °C. To the stirred reaction mixture vinylmagnesium bromide (2.82 mL, 2.82 mmol, 1.0 M in THF, 3.18 equiv.) was added slowly (ca. 15 s), and then mixture was slowly warmed to -20 °C over 30 min. Mixture was stirred for 7.5 h at -20 °C, and then quenched with NH_4Cl_{aq} (5 mL, 10% w/v). Mixture was transferred to separatory funnel, NH_4Cl_{aq} (50 mL, 10% w/v) was added, mixture was extracted with ethyl acetate (3 × 50 mL), combined organic phases were washed with H_2O (50 mL), brine (50 mL), and dried over anhydrous $MgSO_4$. Mixture was filtered, evaporated and separated with column chromatography yielding **9** (87 mg, 0.33 mmol, **37%**) as an off-white solid.

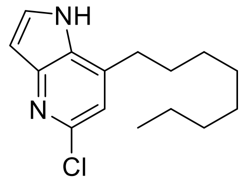

9

Yield: 87 mg (0.33 mmol, **37%**) from **4q** (240 mg, 0.89 mmol)

off-white solid, **mp** 98.5 – 101.5 °C
eluent: cyclohexane/ethyl acetate 20:1 to 1:2

^{1}H NMR (400 MHz, $CDCl_3$): δ = 9.58 (br s, 1H), 7.44 (t, *J* = 3.2 Hz, 1H), 6.95 (s, 1H), 6.59 (dd, *J* = 3.2, 2.0 Hz, 1H), 2.85 – 2.78 (m, 2H), 1.75 – 1.62 (m, 2H), 1.32 – 1.13 (m, 10H), 0.84 (t, *J* = 7.0 Hz, 3H).

^{13}C NMR (100 MHz, $CDCl_3$): δ = 145.2, 144.2, 137.6, 128.6, 127.3, 116.3, 102.8, 31.8, 30.9, 29.4, 29.3, 29.1, 29.0, 22.6, 14.1.

MS (EI): m/z (%): 264 (34, $[M]^+$), 166 (100, $[M-C_7H_{14}]^+$),

HRMS (EI): m/z $[M]^+$ calcd for $C_{15}H_{21}N_2{}^{35}Cl$: 264.1393; found: 264.1399.

7. References

[1] D. Antoniak, M. Barbasiewicz, *Org. Lett.* **2019**, *21*, 9320–9325.

[2] P. K. Shyam, H. Y. Jang, *J. Org. Chem.* **2017**, *82*, 1761–1767.

[3] I. T. Phan, G. J. Gilbert, G. W. O'Neil, *Synlett* **2015**, *26*, 1867–1871.

[4] D. Nawrot, M. Kolenič, J. Kuneš, F. Kostelansky, M. Miletin, V. Novakova, P. Zimcik, *Tetrahedron* **2018**, *74*, 594–599.

[5] H. G. Viehe, S. Y. Delavarenne, *Chem. Ber.* **1970**, *103*, 1216–1224.

[6] W. E. Parham, L. D. Edwards, *J. Org. Chem.* **1968**, *33*, 4150–4154.

[7] B. Górski, D. Basiak, Ł. Grzesiński, M. Barbasiewicz, *Org. Biomol. Chem.* **2019**, *17*, 7660–7663.

[8] X. Marset, G. Guillena, D. J. Ramón, *Chem. Eur. J.* **2017**, *23*, 10522–10526.

[9] Y. G. Borisova, G. Z. Raskildina, S. S. Zlotskii, *Russ. J. Gen. Chem.* **2016**, *86*, 1954–1956.

[10] S. Mannathan, C. H. Cheng, *Chem. Eur. J.* **2012**, *18*, 11771–11777.

[11] B. Y. Hao, J. Q. Liu, W. H. Zhang, X. Z. Chen, *Synthesis* **2011**, 1208–1212.

[12] N. Margraf, G. Manolikakes, *J. Org. Chem.* **2015**, *80*, 2582–2600.

[13] B. Górski, A. Talko, T. Basak, M. Barbasiewicz, *Org. Lett.* **2017**, *19*, 1756–1759.

[14] W. E. Truce, D. J. Vrencur, *J. Org. Chem.* **1970**, *35*, 1226–1227.

[15] B. Sreedhar, P. Surendra Reddy, M. Amarnath Reddy, *Synthesis* **2009**, 1732–1738.

[16] H. C. Winter, F. E. Reinhart, *J. Am. Chem. Soc.* **1940**, *62*, 3508–3511.

[17] T. Kuramochi, A. Kakefuda, H. Yamada, I. Sato, T. Taguchi, S. Sakamoto, *Bioorg. Med. Chem.* **2004**, *12*, 5039–5056.

[18] K. R. Reddy, C. U. Maheswari, M. Venkateshwar, M. L. Kantam, *Adv. Synth. Catal.* **2009**, *351*, 93–96.

[19] P. J. Amal Joseph, S. Priyadarshini, M. Lakshmi Kantam, H. Maheswaran, *Tetrahedron Lett.* **2012**, *53*, 1511–1513.

[20] D. H. G. Crout, J. R. Penton, K. Schofield, *J. Chem. Soc. B* **1971**, 1254–1256.

[21] A. R. Katritzky, E. F. V. Scriven, S. Majumder, R. G. Akhmedova, A. V. Vakulenko, N. G. Akhmedov, R. Murugan, K. A. Abboud, *Org. Biomol. Chem.* **2005**, *3*, 538–541.

[22] F. Y. Al-Mkhaizim, M. F. Greaney, *Synlett* **2020**, 1094–1096.

[23] Z. Zhang, Z.Yang, N. A. Meanwell, J. F. Kadow, T. Wang, *J. Org. Chem.* **2002**, *67*, 2345–2347.

8. Reproductions of NMR spectra

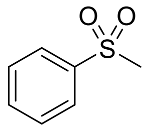

2a

^{1}H NMR (400 MHz, $CDCl_3$)

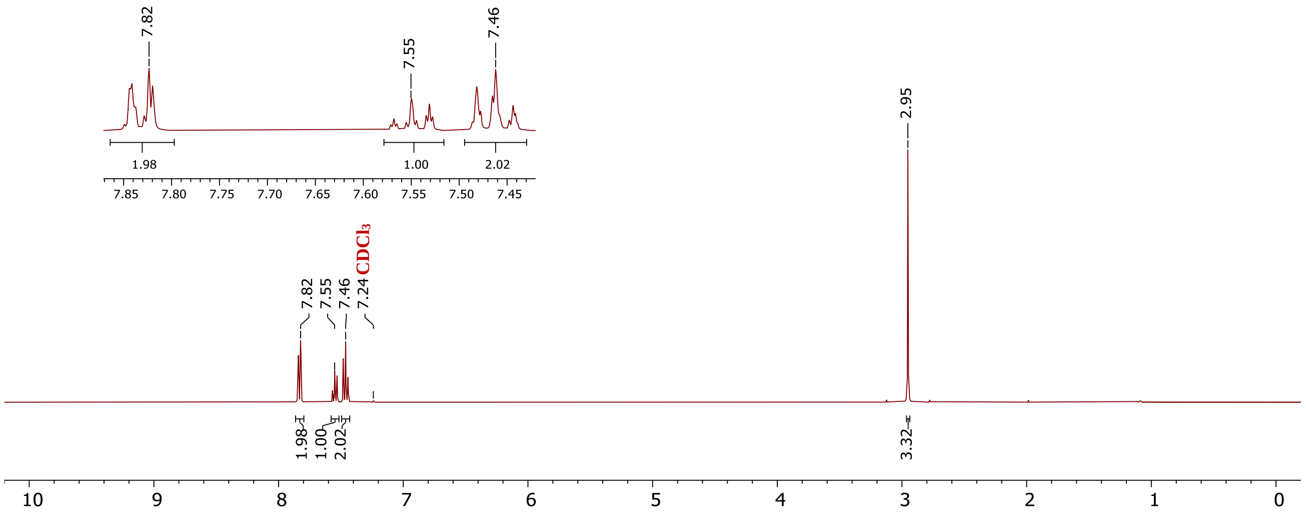

^{13}C NMR (100 MHz, $CDCl_3$)

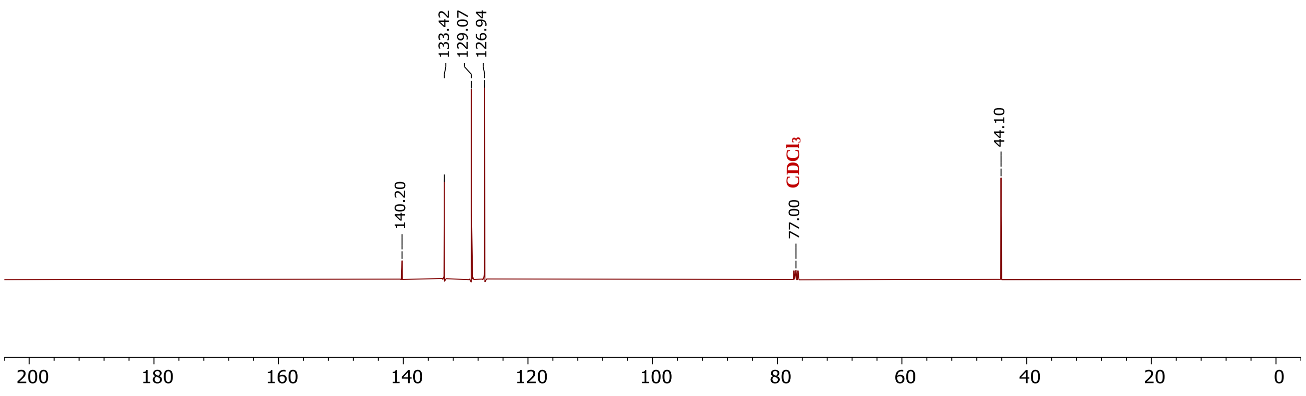

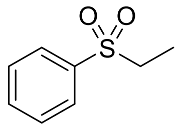

2b

^{1}H NMR (400 MHz, $CDCl_3$)

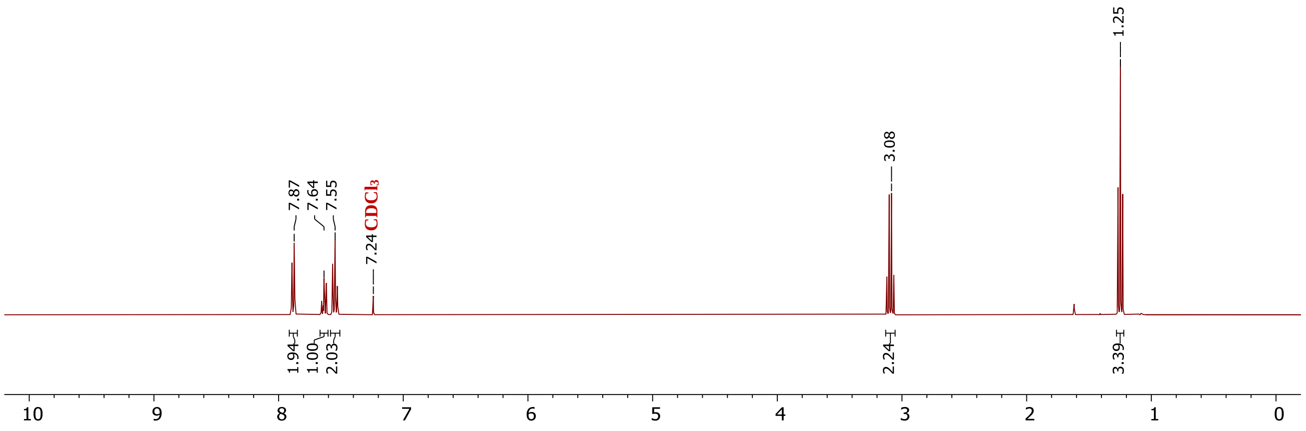

^{13}C NMR (100 MHz, $CDCl_3$)

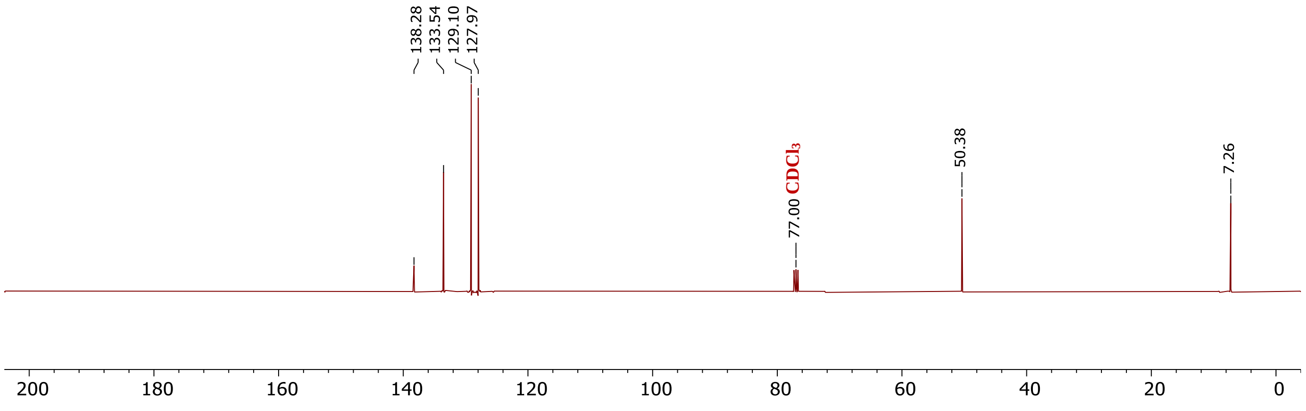

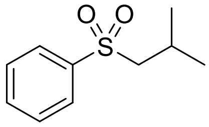

2c

^{1}H NMR (400 MHz, $CDCl_3$)

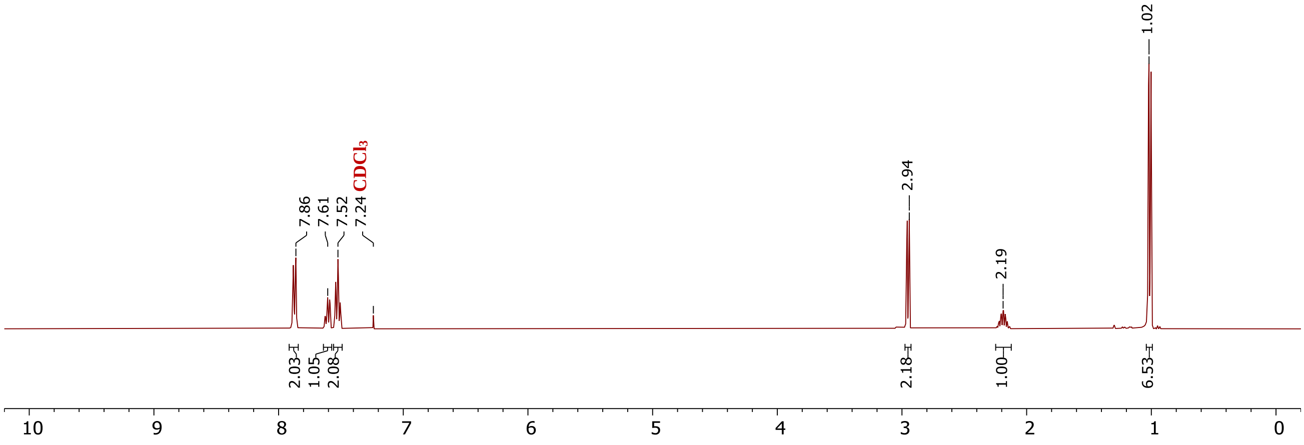

^{13}C NMR (100 MHz, $CDCl_3$)

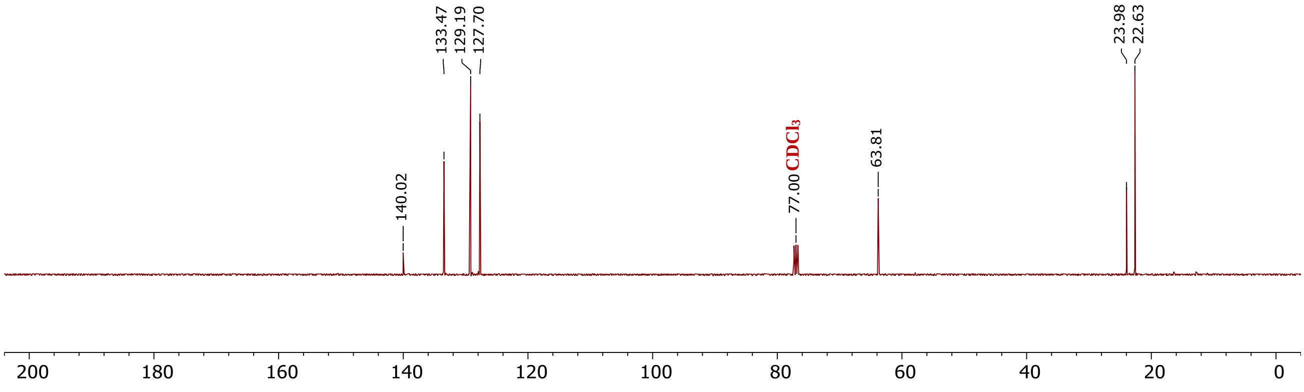

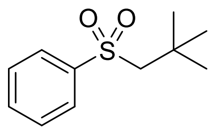

2d

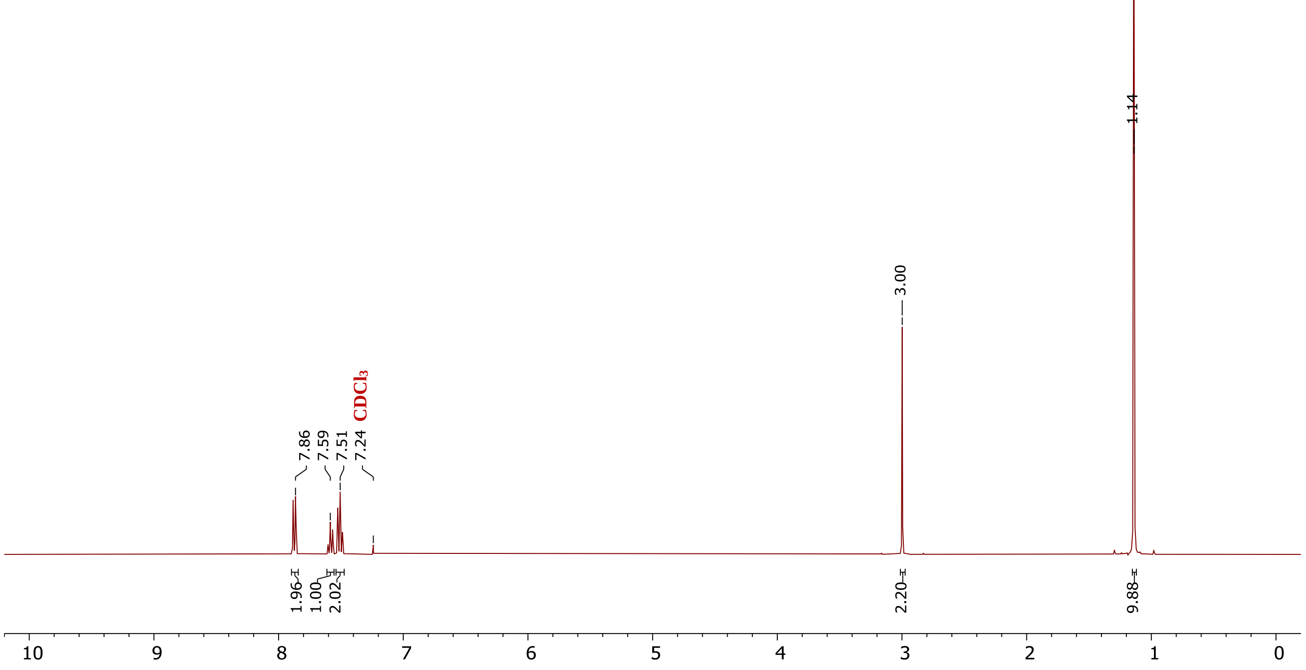

^{1}H NMR (400 MHz, CDCl$_3$)
7.86
7.59
7.51
7.24
CDCl$_3$
3.00
1.14
1.96
1.00
2.02
2.20
9.88
10
9
8
7
6
5
4
3
2
1
0

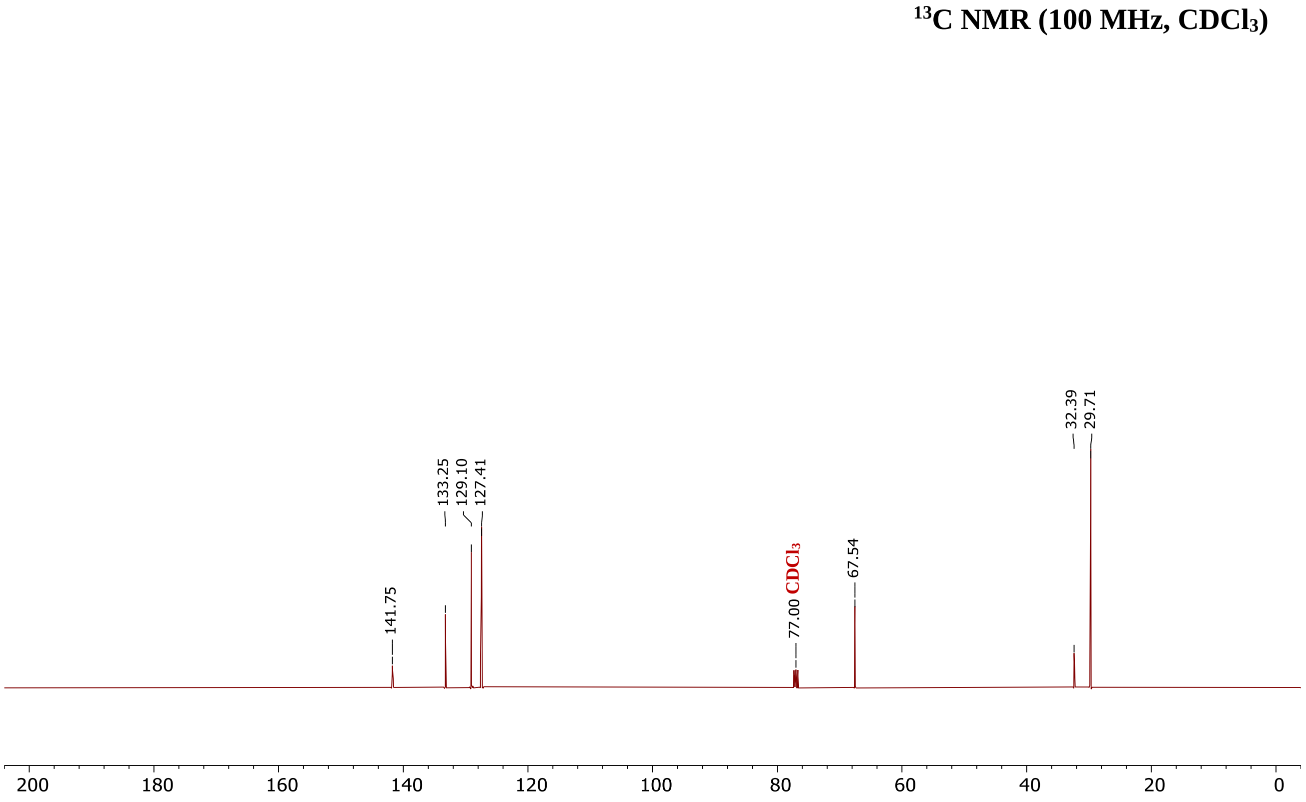

^{13}C NMR (100 MHz, CDCl$_3$)
141.75
133.25
129.10
127.41
77.00 CDCl$_3$
67.54
32.39
29.71
200
180
160
140
120
100
80
60
40
20
0

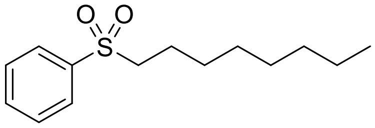

2e

^{1}H NMR (400 MHz, CDCl$_3$)

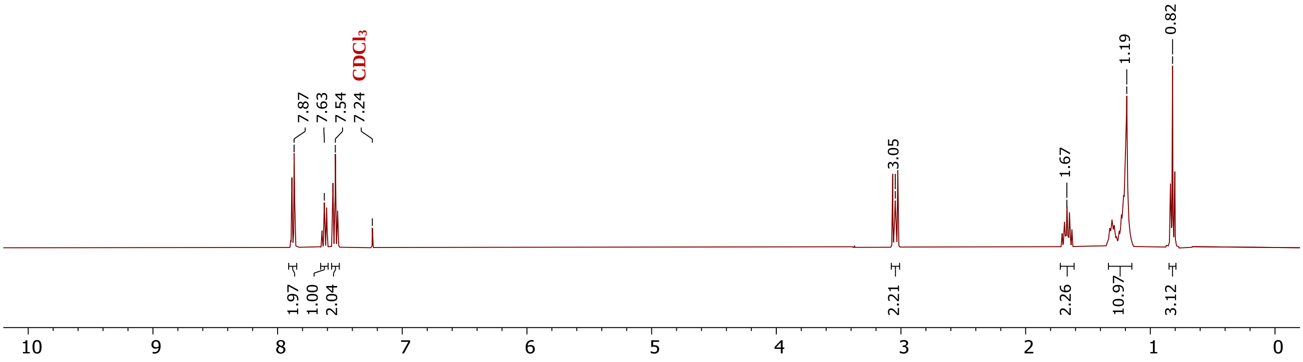

^{13}C NMR (100 MHz, CDCl$_3$)

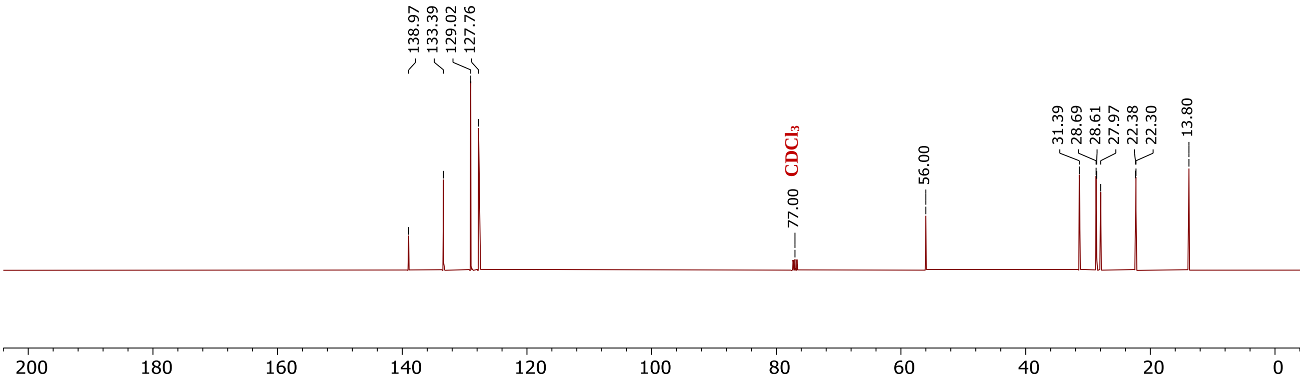

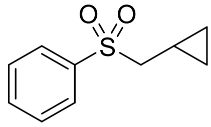

2f

^{1}H NMR (400 MHz, $CDCl_3$)

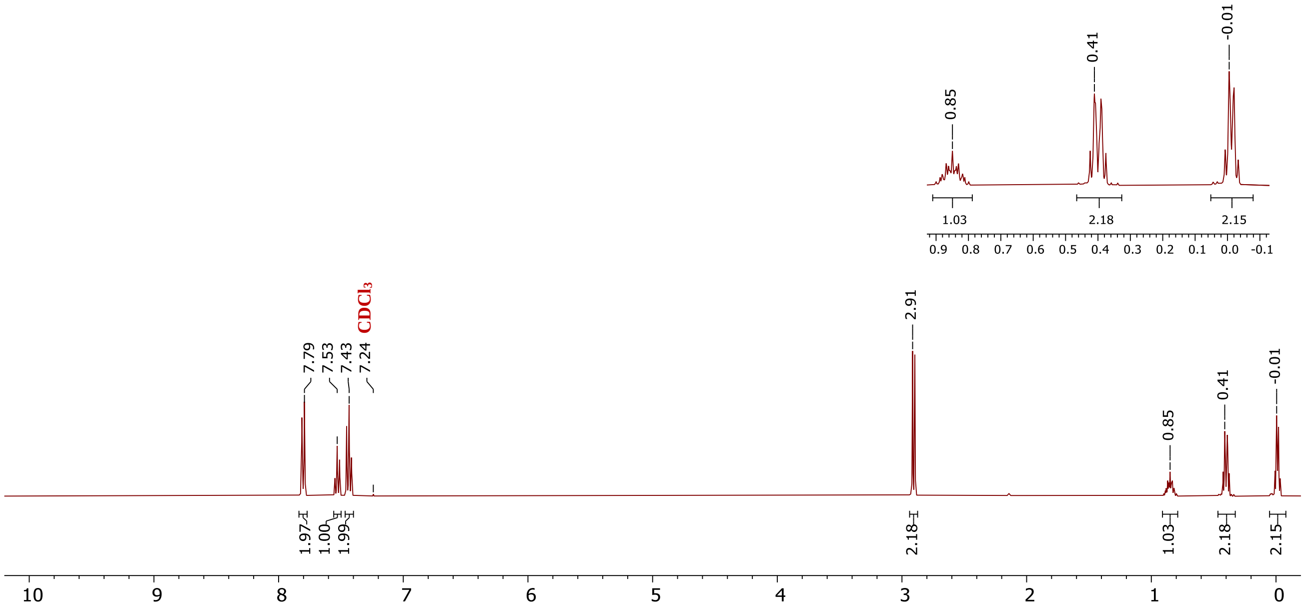

^{13}C NMR (100 MHz, $CDCl_3$)

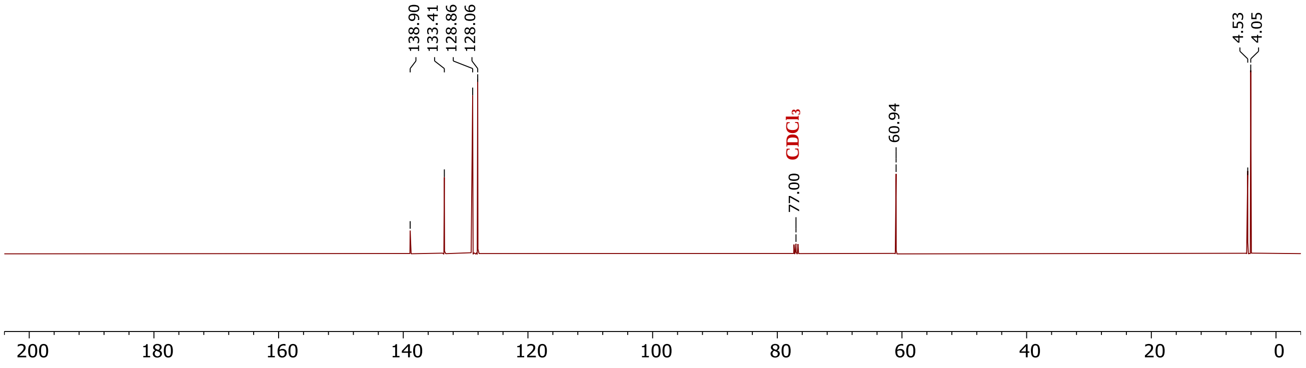

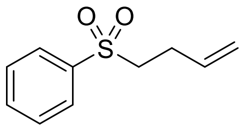

2g'

^{1}H NMR (400 MHz, $CDCl_3$)

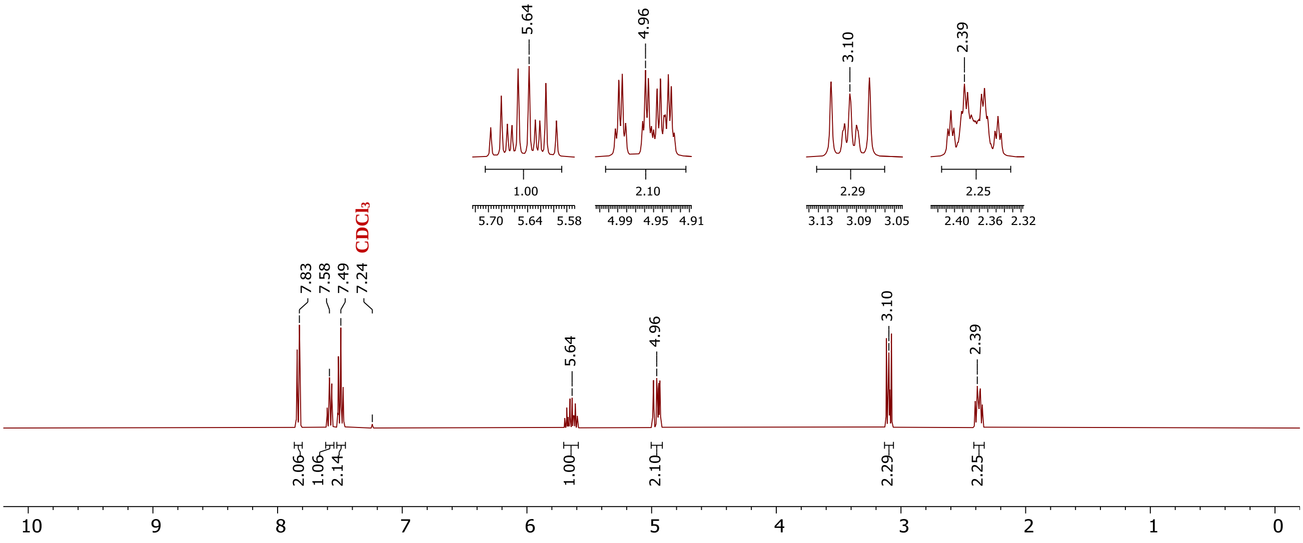

^{13}C NMR (100 MHz, $CDCl_3$)

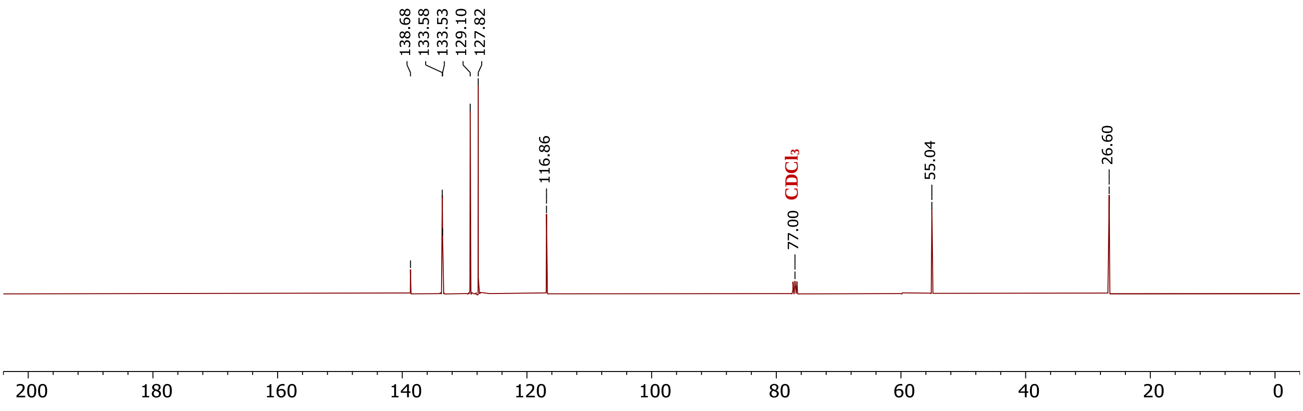

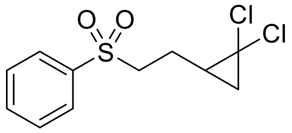

2g

^{1}H NMR (400 MHz, CDCl$_3$)

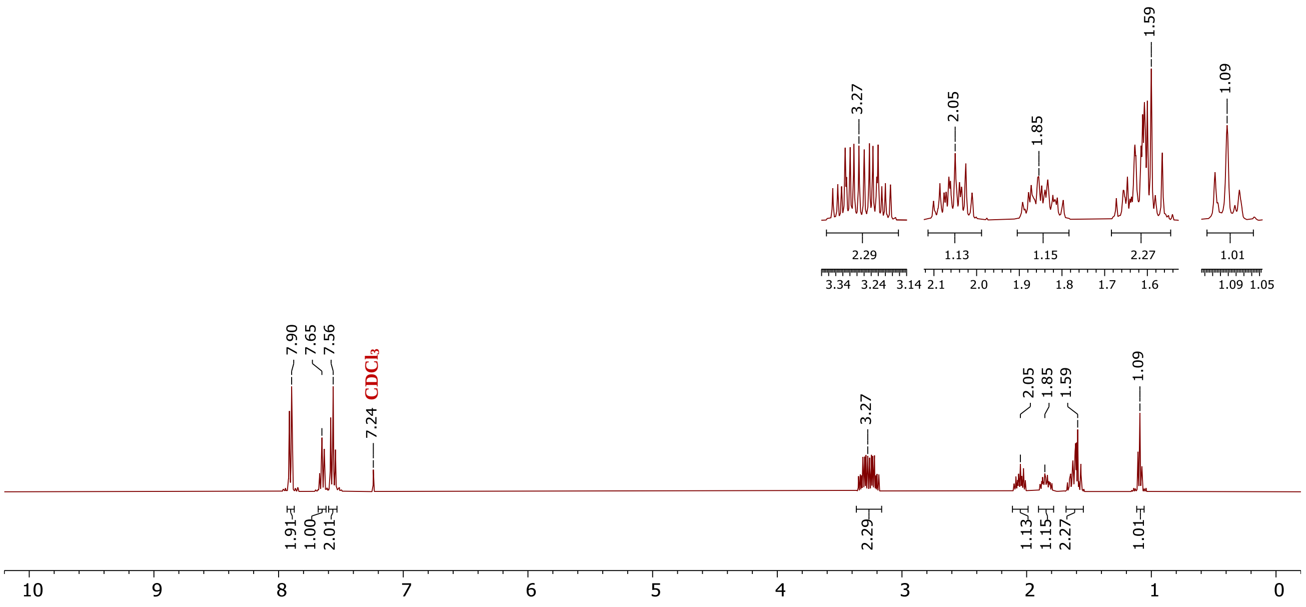

^{13}C NMR (100 MHz, CDCl$_3$)

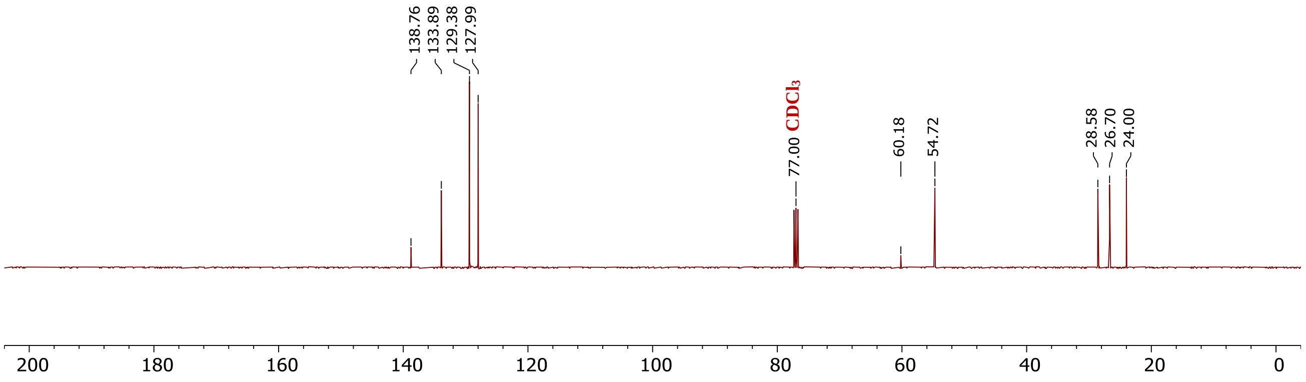

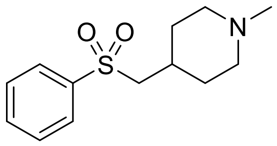

2h

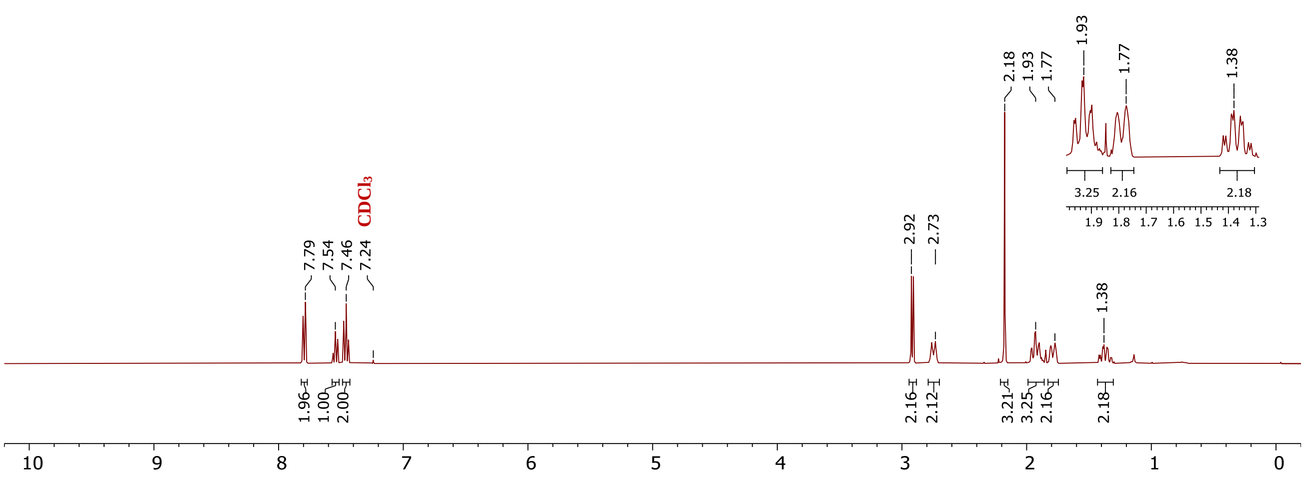
¹H NMR (400 MHz, CDCl₃)
CDCl₃
7.79
7.54
7.46
7.24
2.92
2.73
2.18
1.93
1.77
1.38
1.96
1.00
2.00
2.16
2.12
3.21
3.25
2.16
2.18

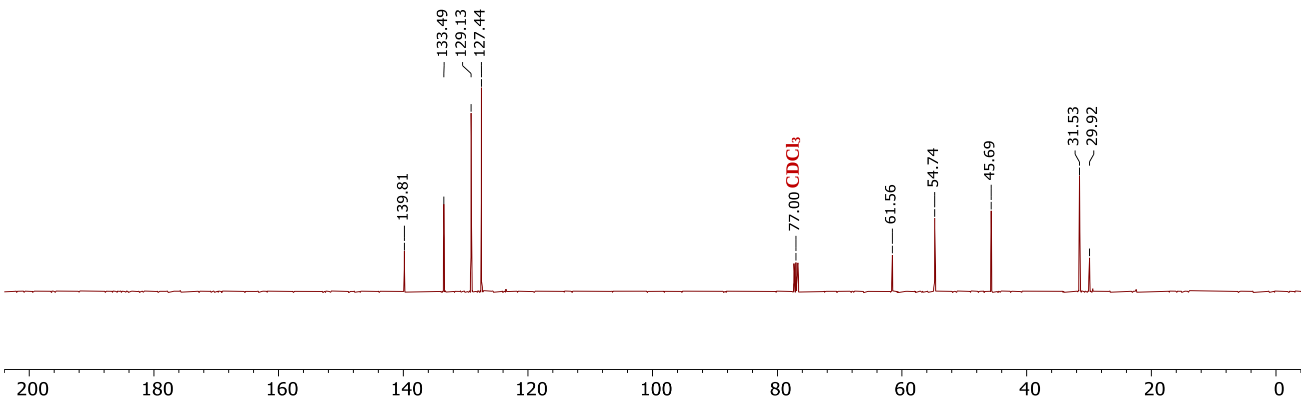
¹³C NMR (100 MHz, CDCl₃)
139.81
133.49
129.13
127.44
77.00 CDCl₃
61.56
54.74
45.69
31.53
29.92

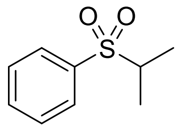

2i

^{1}H NMR (400 MHz, $CDCl_3$)

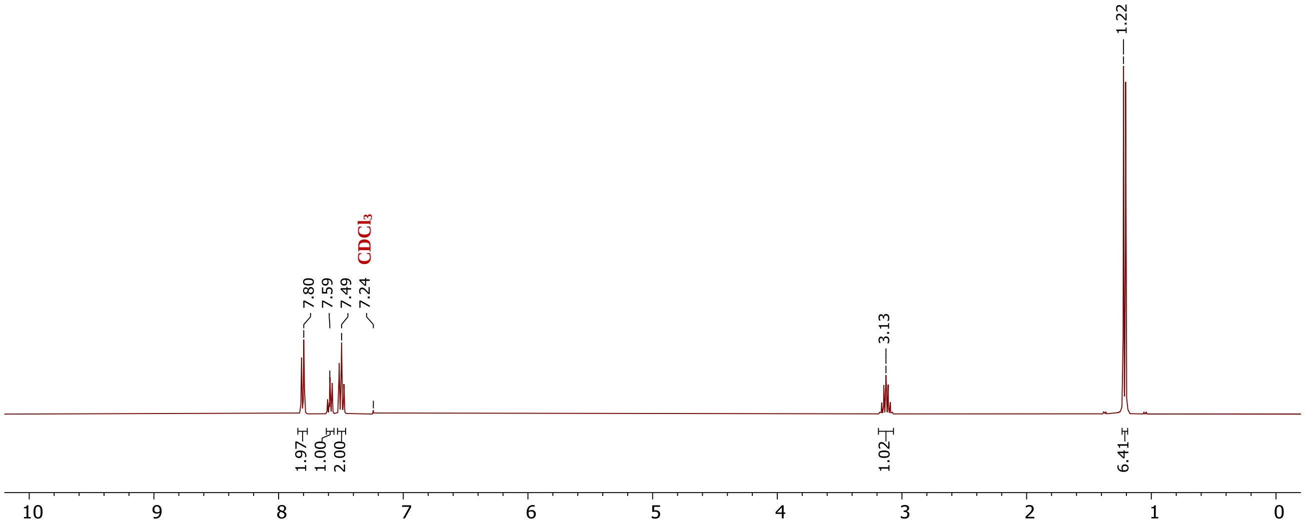

^{13}C NMR (100 MHz, $CDCl_3$)

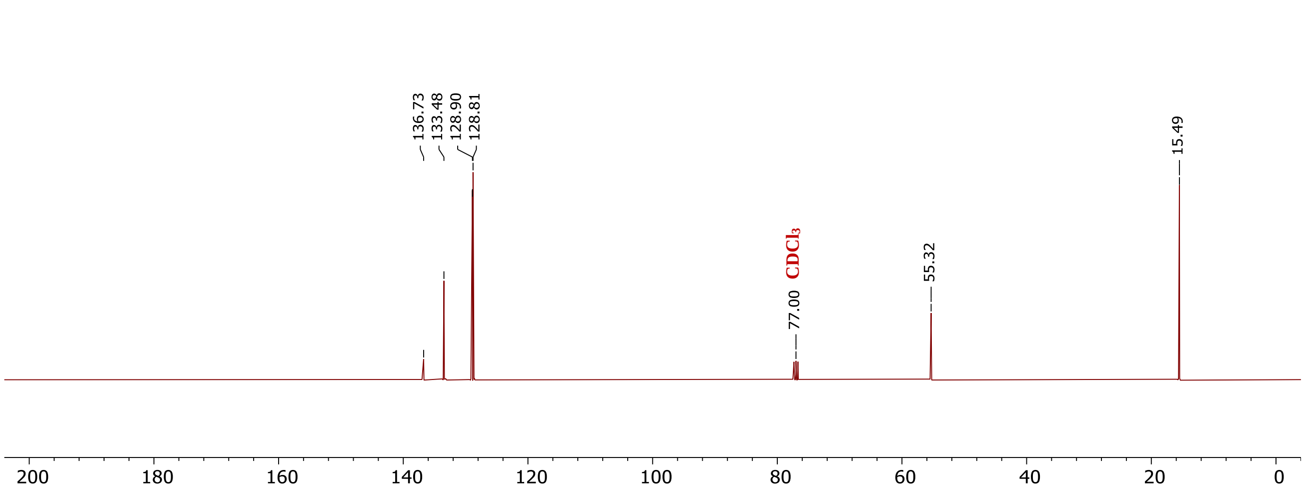

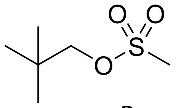

3a

^{1}H NMR (400 MHz, $CDCl_3$)

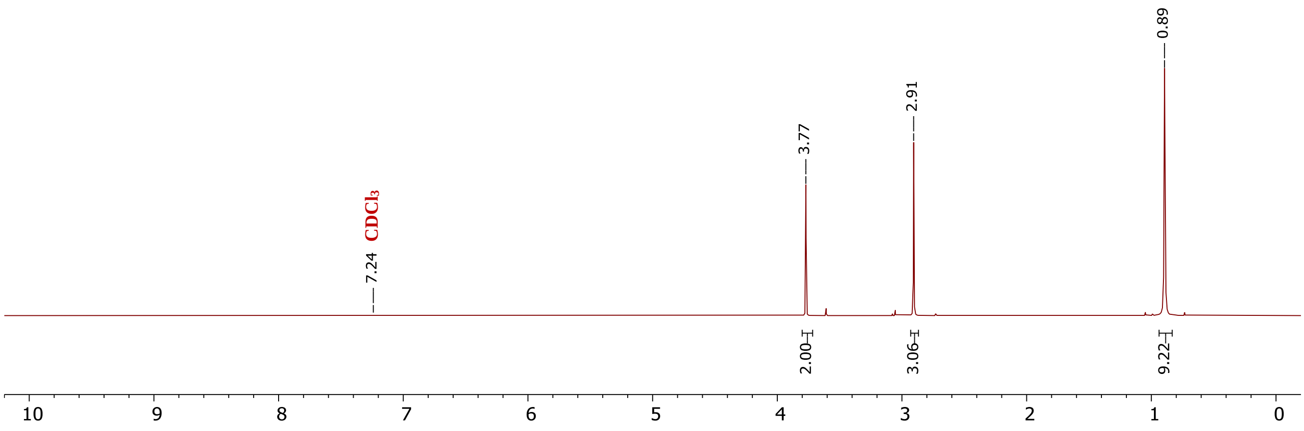

^{13}C NMR (100 MHz, $CDCl_3$)

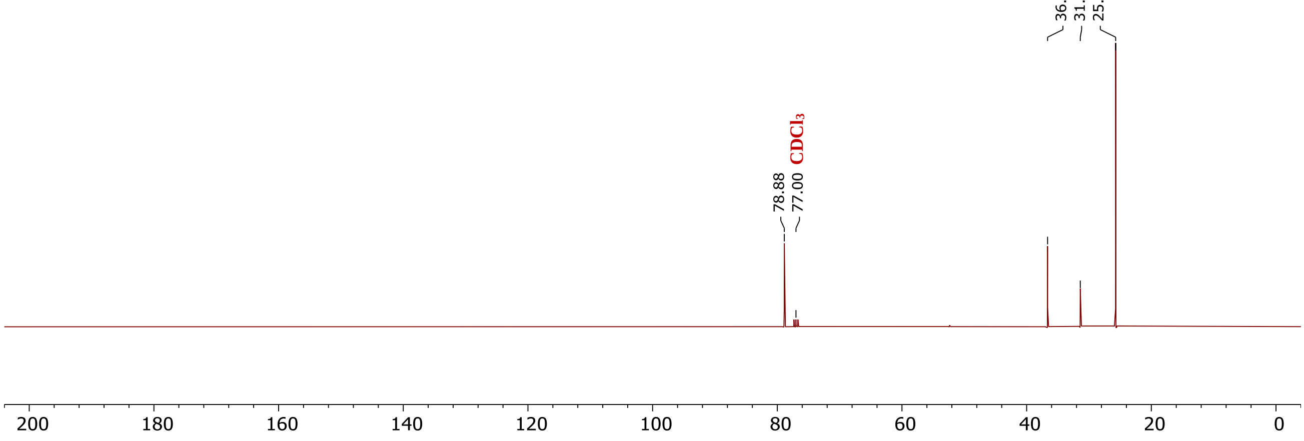

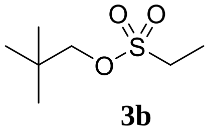

3b

^{1}H NMR (400 MHz, $CDCl_3$)

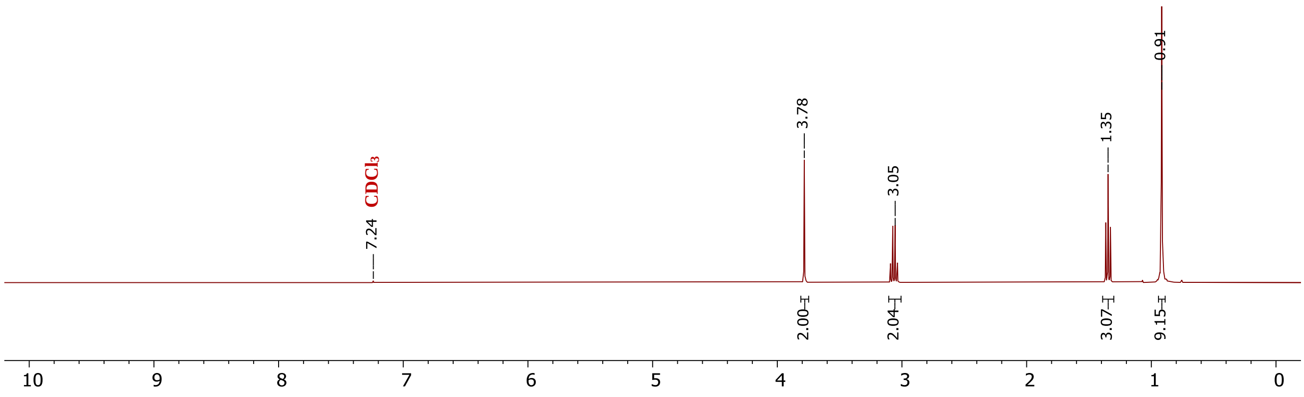

^{13}C NMR (100 MHz, $CDCl_3$)

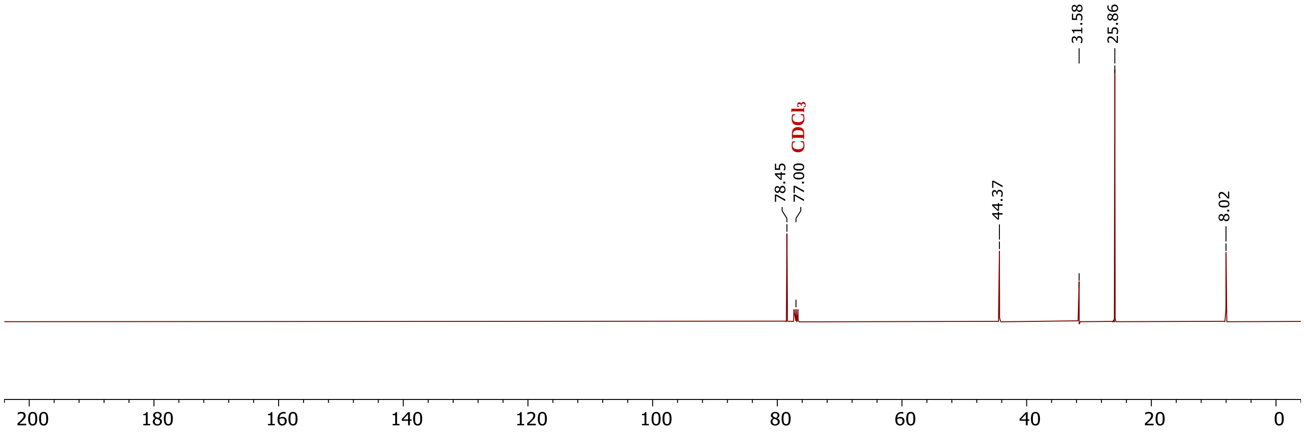

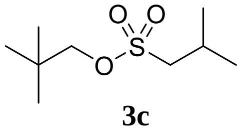

3c

^{1}H NMR (400 MHz, $CDCl_3$)

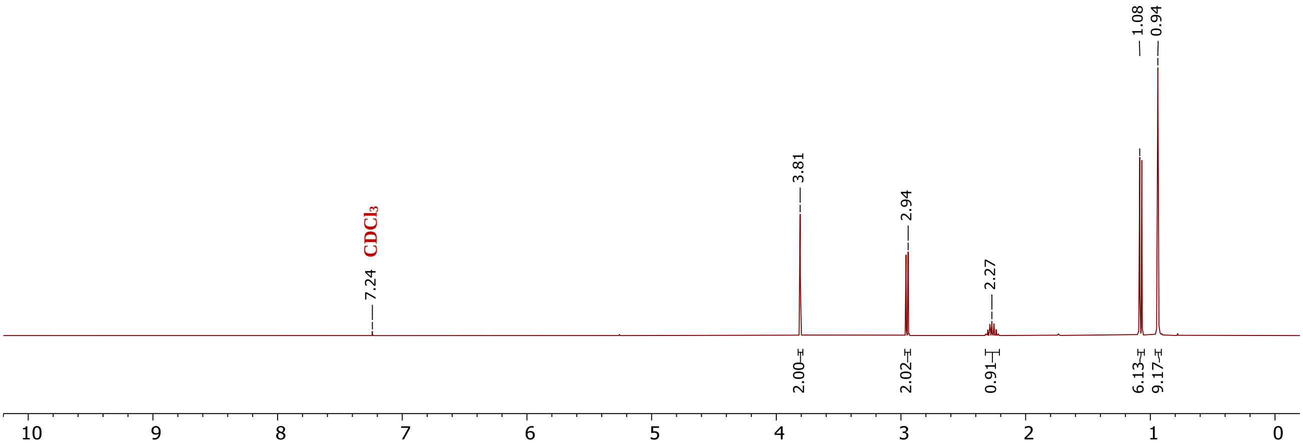

^{13}C NMR (100 MHz, $CDCl_3$)

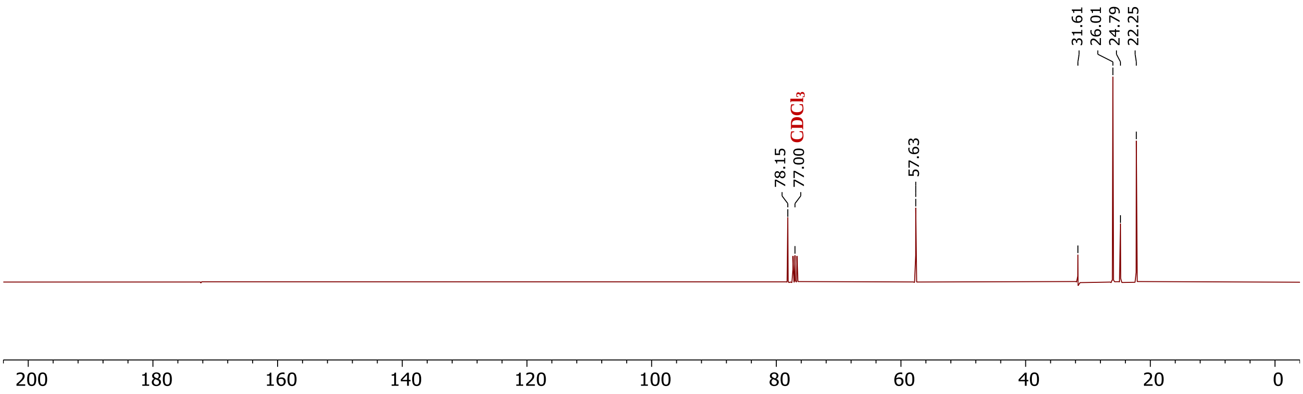

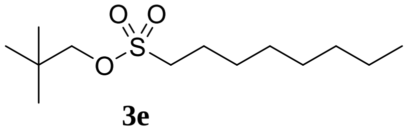

3e

^{1}H NMR (400 MHz, $CDCl_3$)

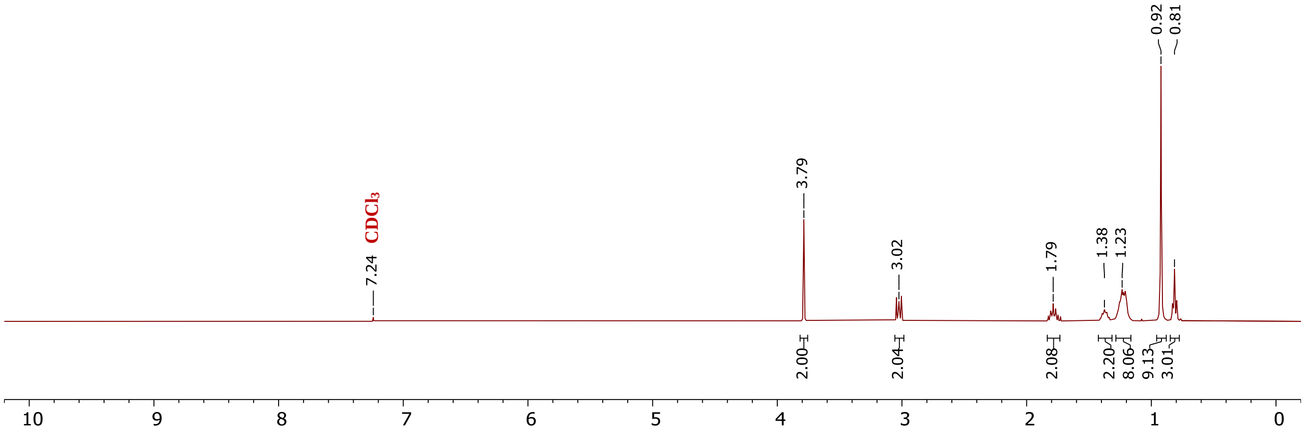

^{13}C NMR (100 MHz, $CDCl_3$)

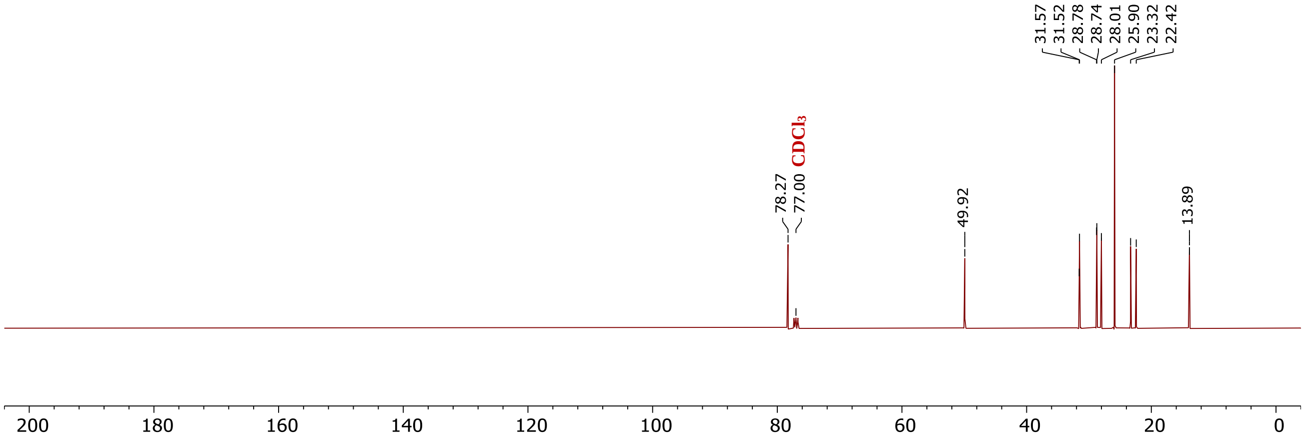

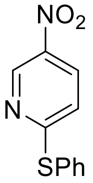
NO2
N
SPh
1d

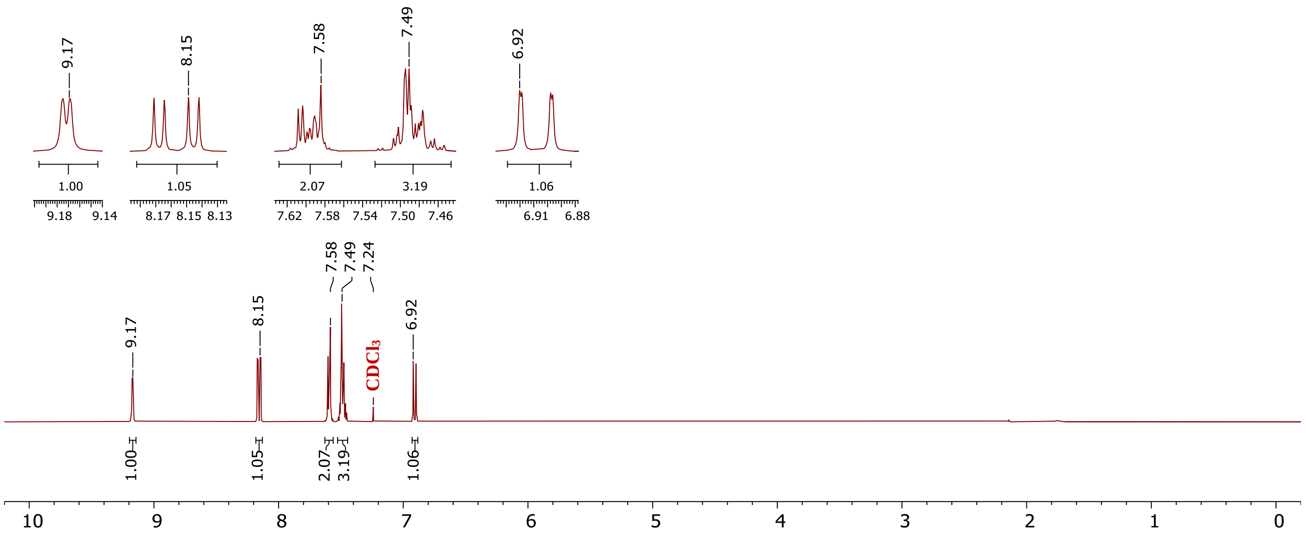
1H NMR (400 MHz, CDCl3)
9.17
8.15
7.58
7.49
6.92
7.24
CDCl3
1.00
1.05
2.07
3.19
1.06

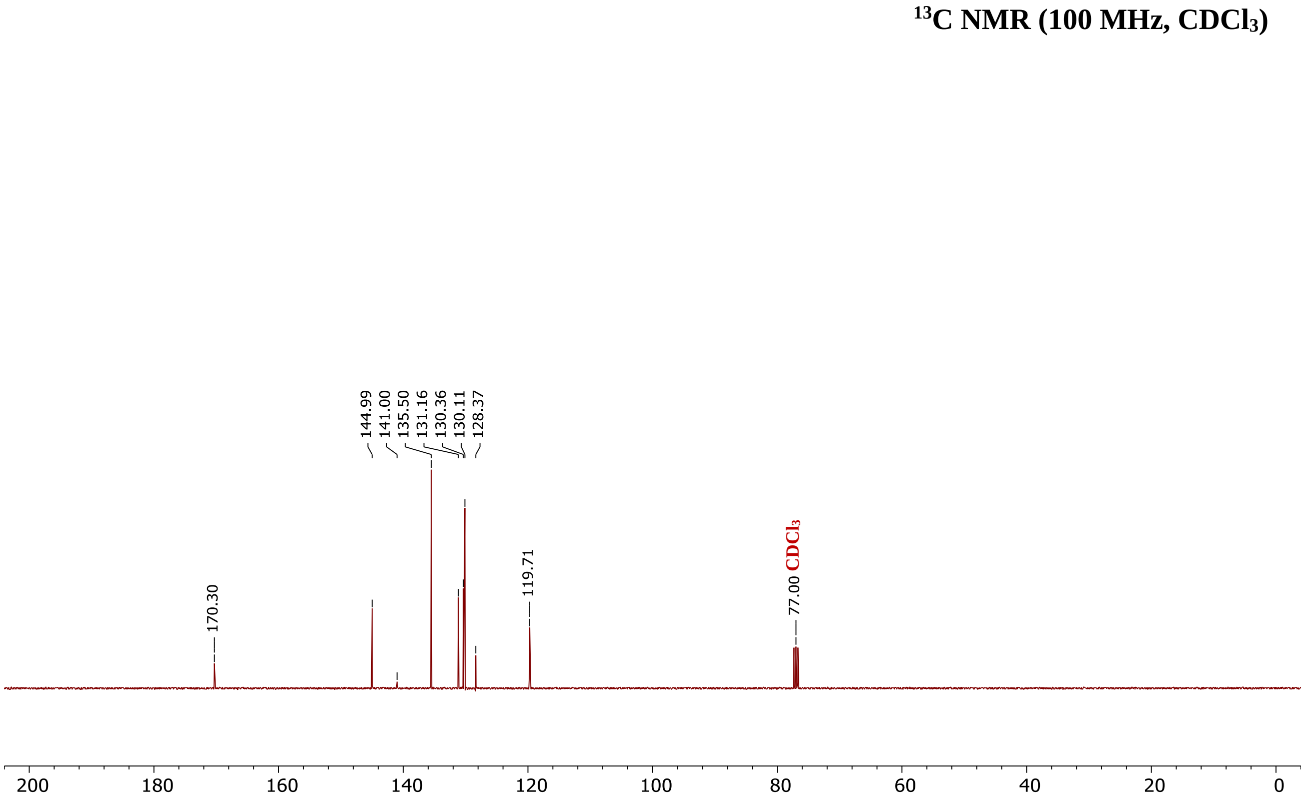
13C NMR (100 MHz, CDCl3)
170.30
144.99
141.00
135.50
131.16
130.36
130.11
128.37
119.71
77.00 CDCl3

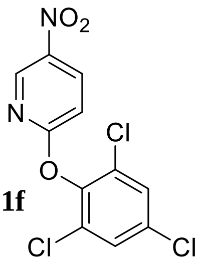

1f

^{1}H NMR (400 MHz, $CDCl_3$)

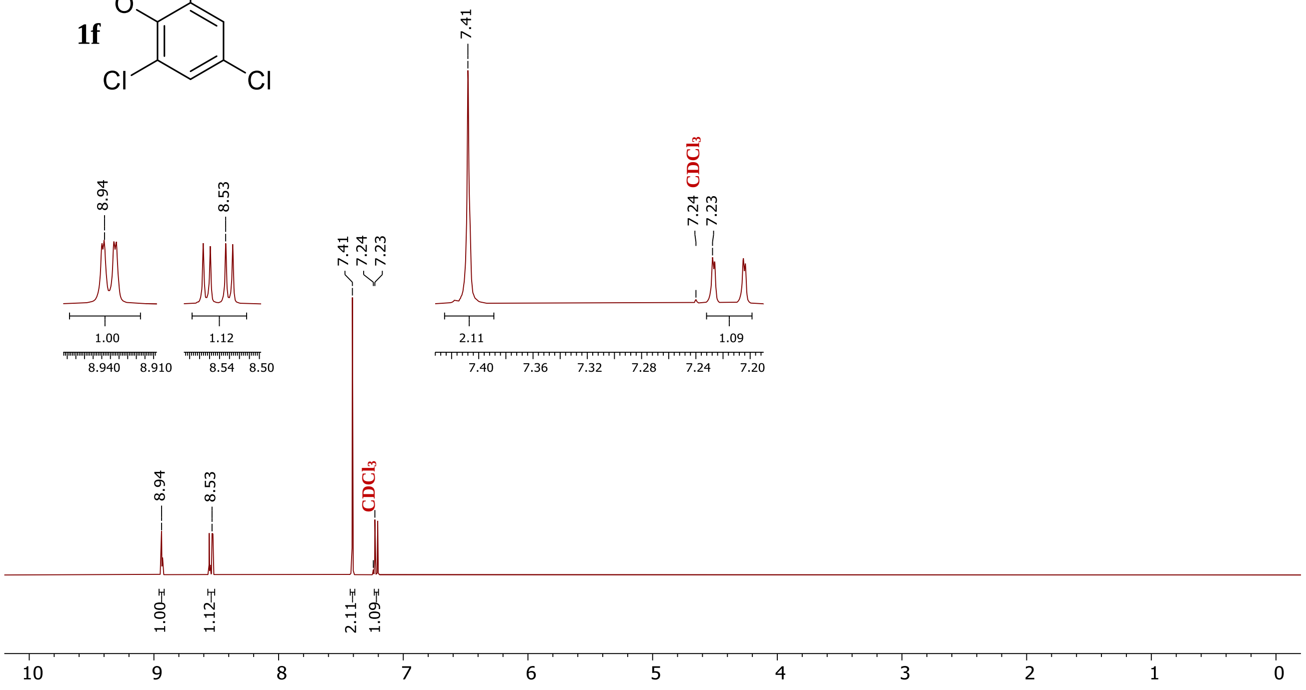

^{13}C NMR (100 MHz, $CDCl_3$)

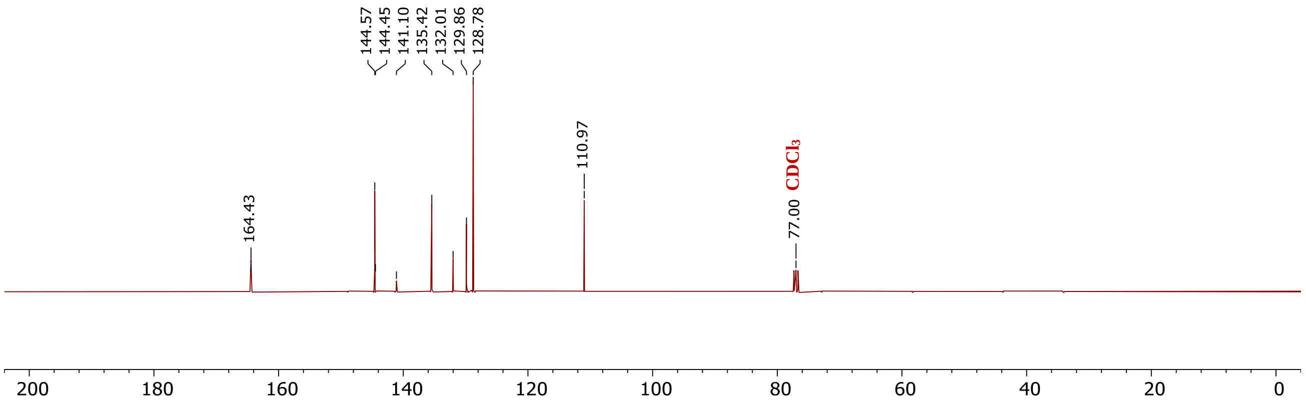

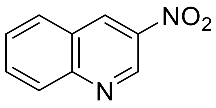

1h

^{1}H NMR (400 MHz, CDCl$_3$)

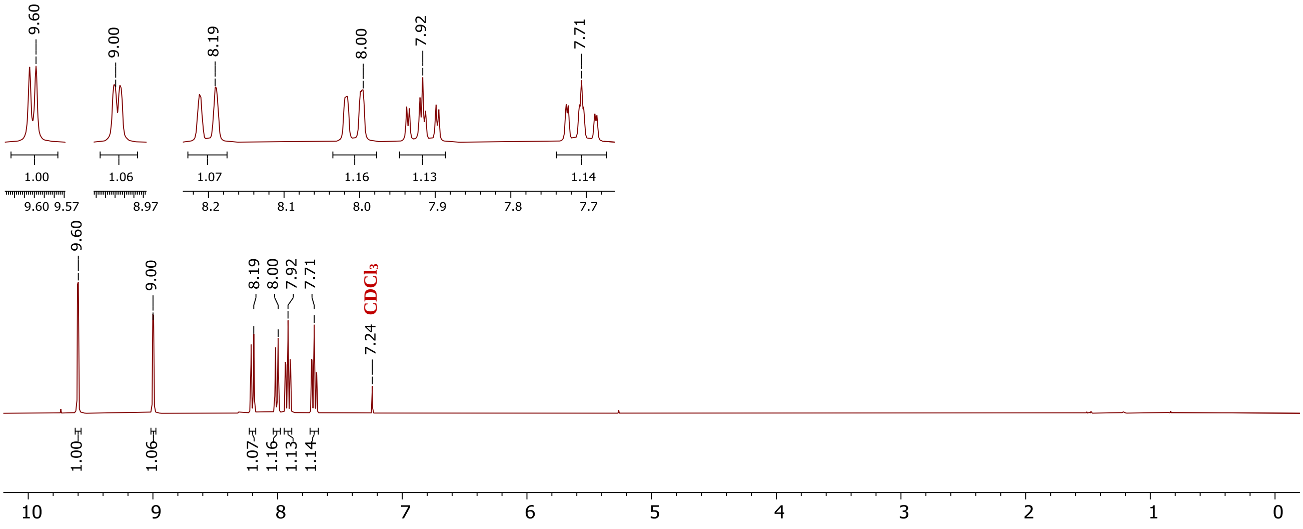

^{13}C NMR (100 MHz, CDCl$_3$)

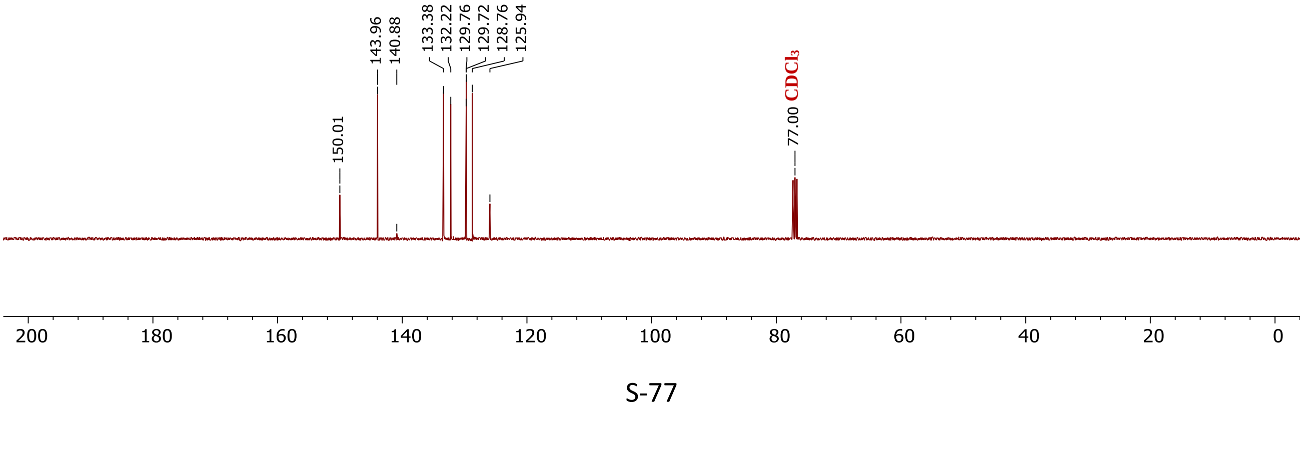

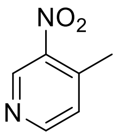

4a

^{1}H NMR (400 MHz, $CDCl_3$)

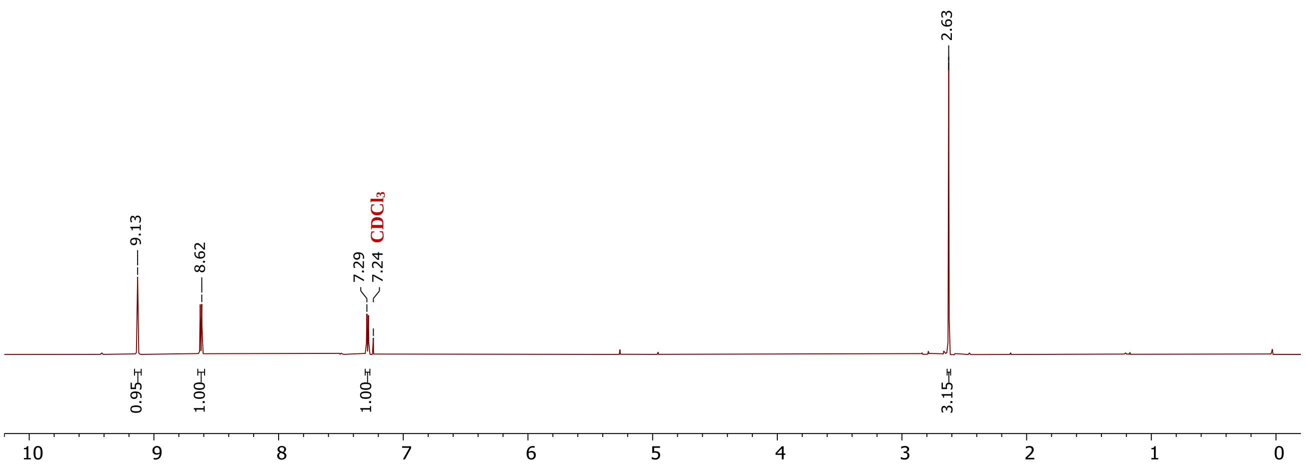

^{13}C NMR (100 MHz, $CDCl_3$)

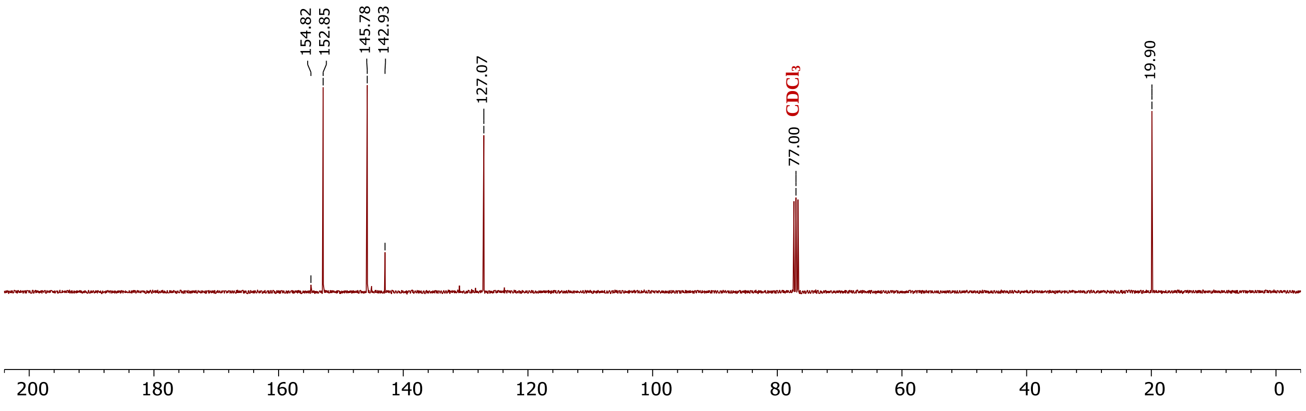

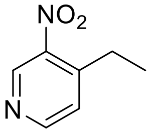

4b

^{1}H NMR (400 MHz, $CDCl_3$)

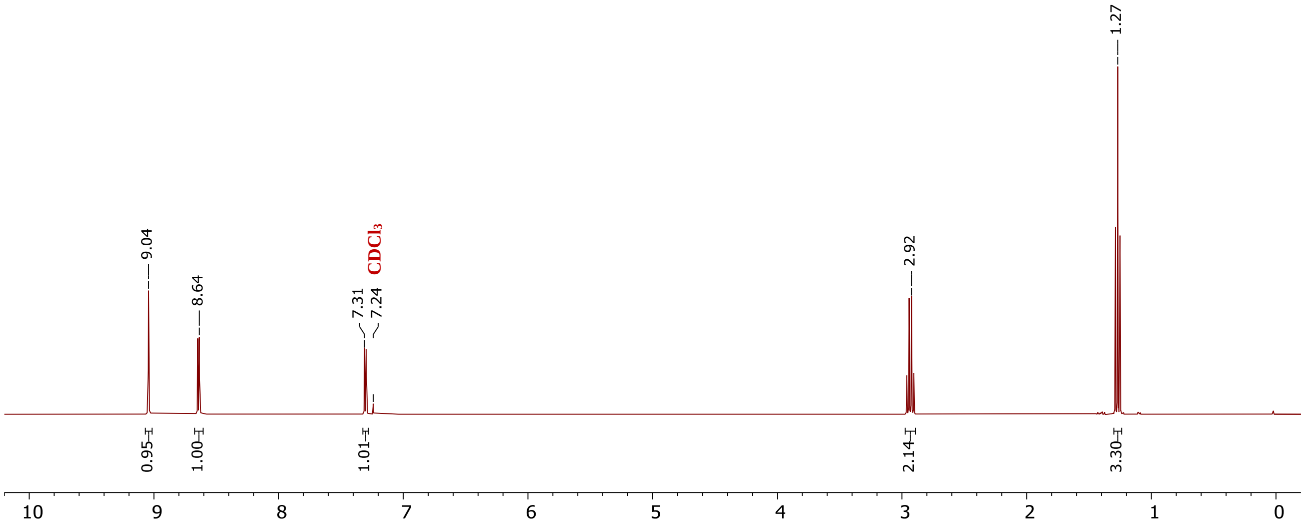

^{13}C NMR (100 MHz, $CDCl_3$)

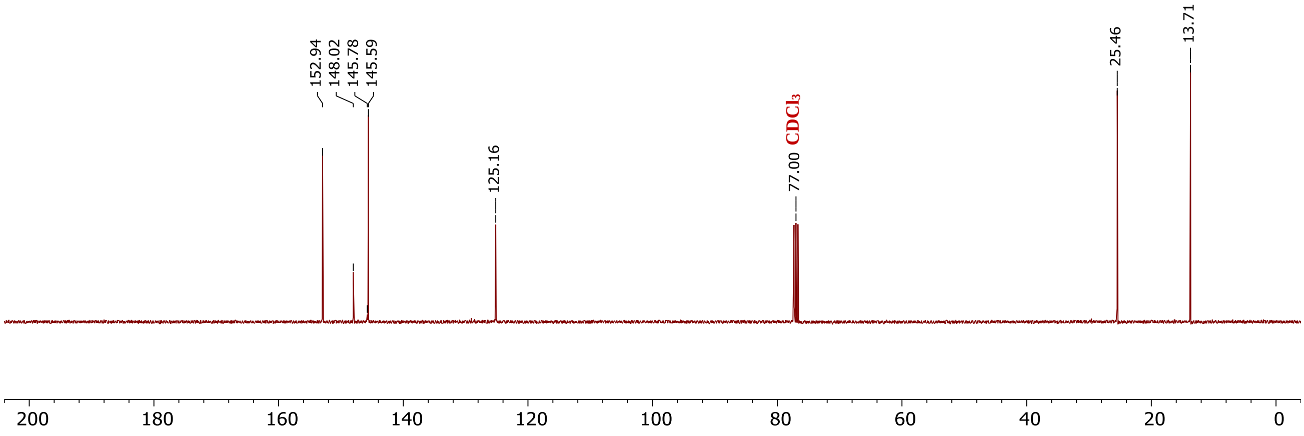

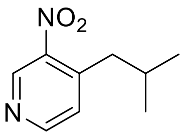

4c

^{1}H NMR (400 MHz, CDCl$_3$)

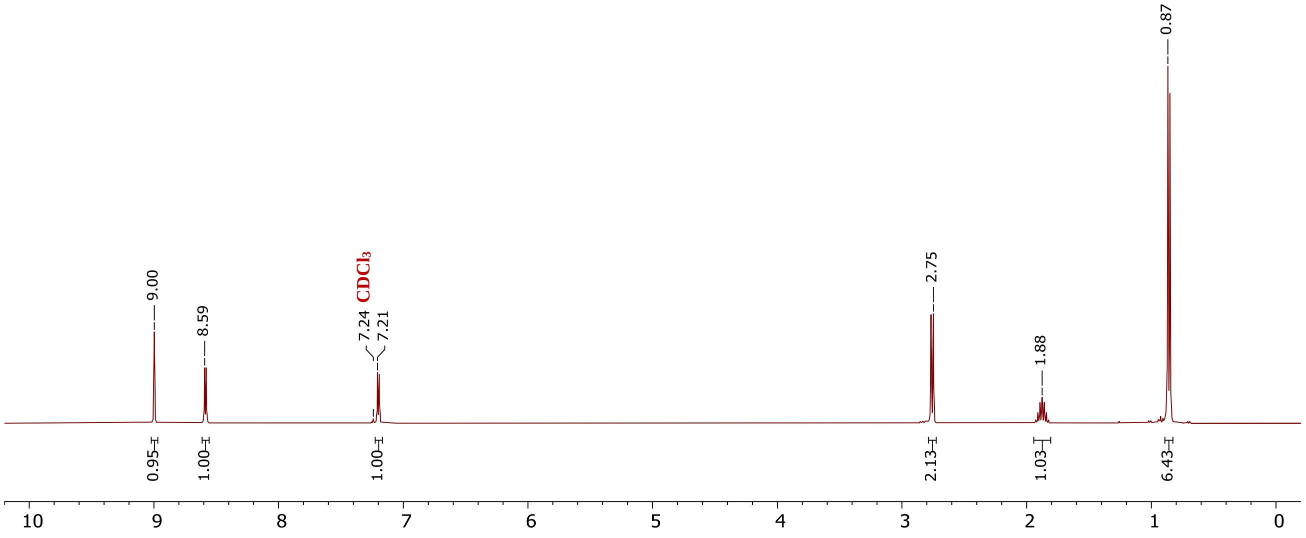

^{13}C NMR (100 MHz, CDCl$_3$)

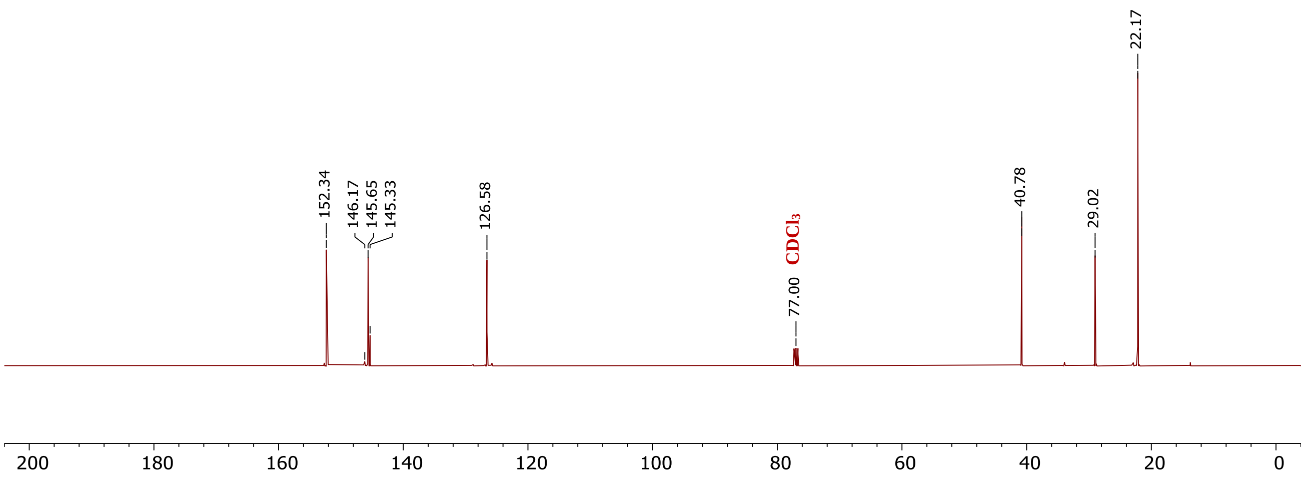

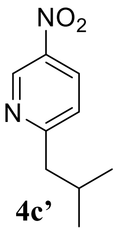

4c'

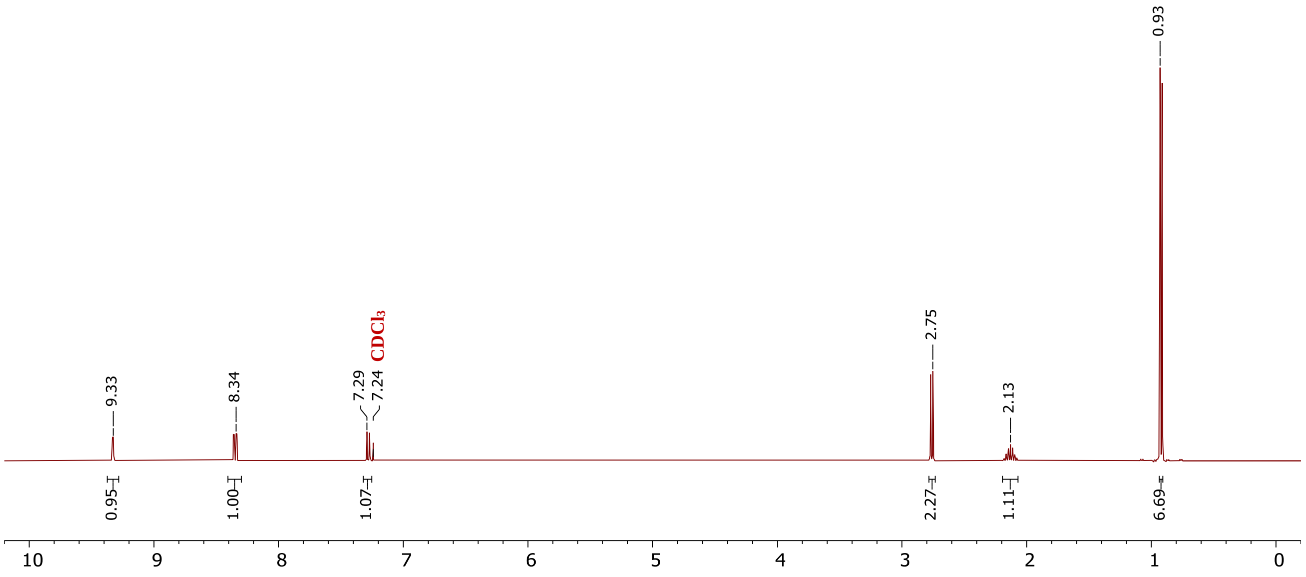

^{1}H NMR (400 MHz, CDCl$_3$)
9.33
8.34
7.29
7.24
CDCl3
2.75
2.13
0.93
0.95
1.00
1.07
2.27
1.11
6.69
10
9
8
7
6
5
4
3
2
1
0

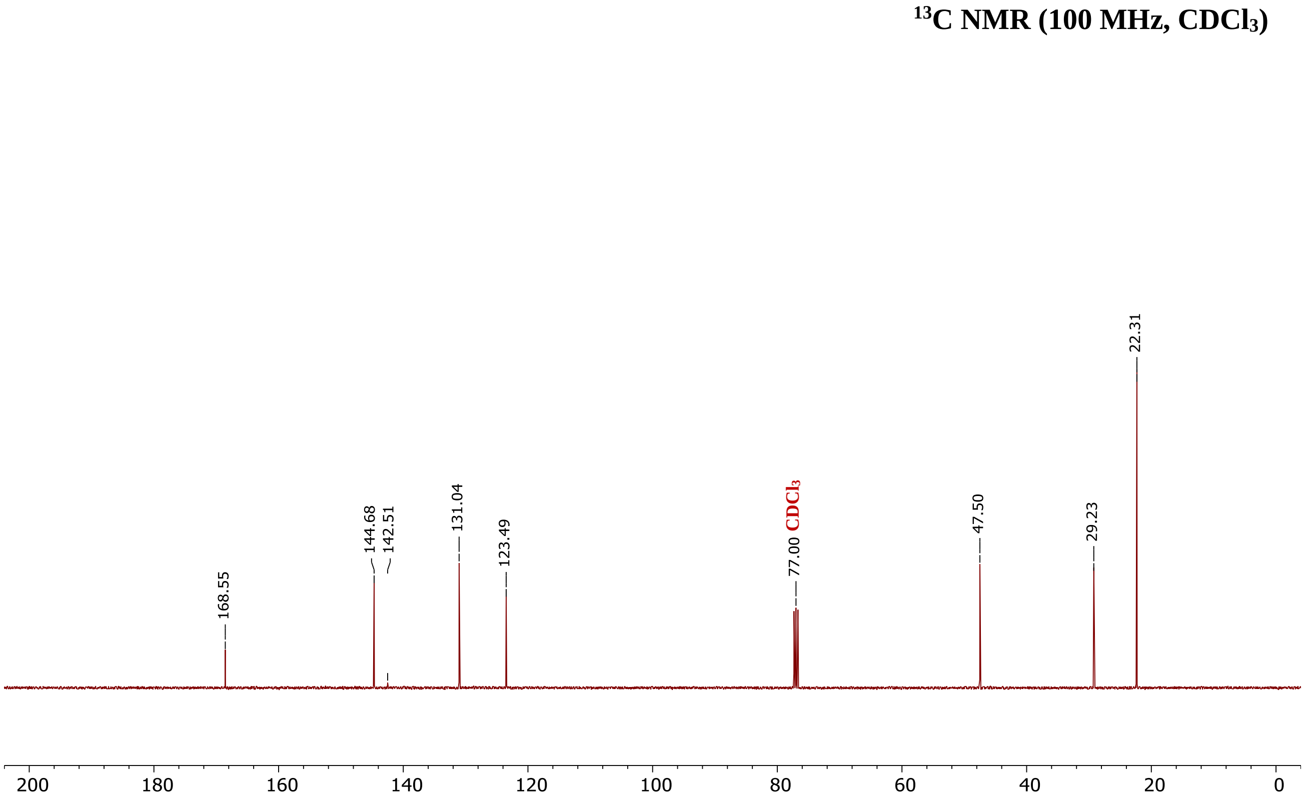

^{13}C NMR (100 MHz, CDCl$_3$)
168.55
144.68
142.51
131.04
123.49
77.00 CDCl3
47.50
29.23
22.31
200
180
160
140
120
100
80
60
40
20
0

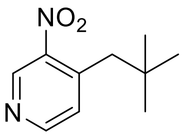

4d

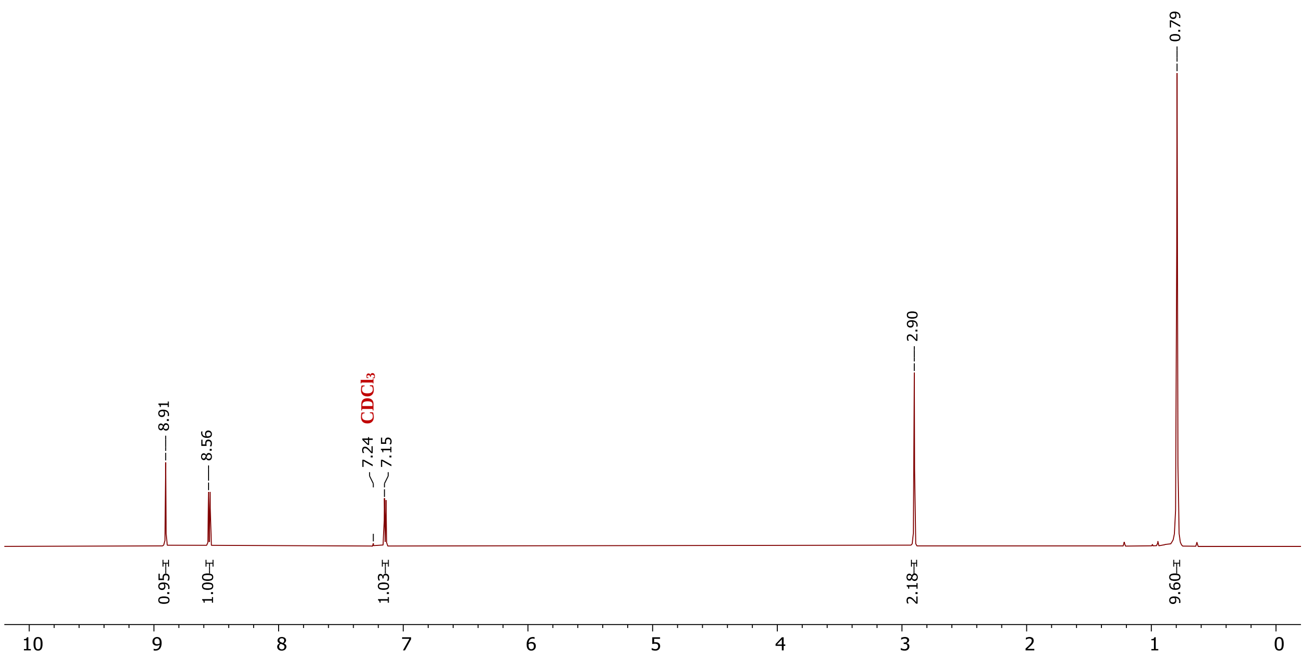
1H NMR (400 MHz, CDCl3)
8.91
8.56
CDCl3
7.24
7.15
2.90
0.79
0.95
1.00
1.03
2.18
9.60
10
9
8
7
6
5
4
3
2
1
0

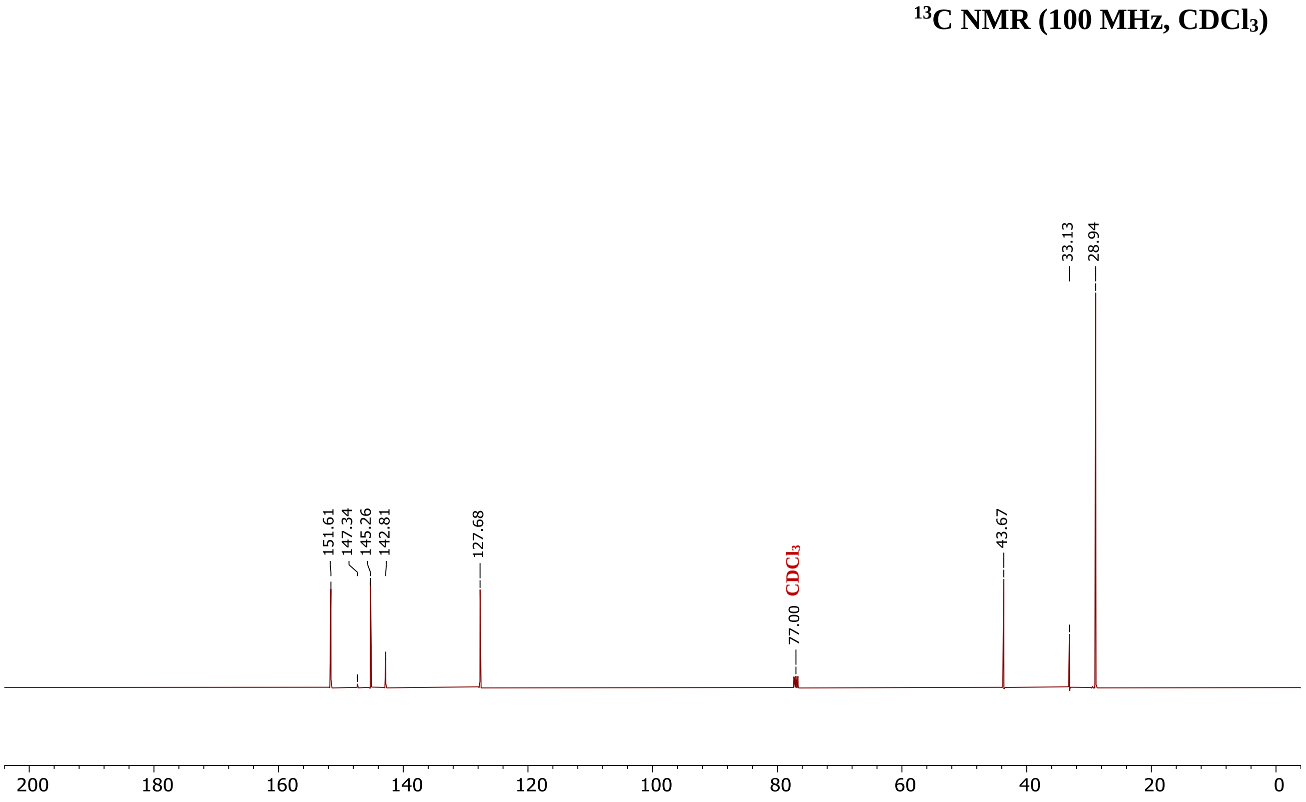
13C NMR (100 MHz, CDCl3)
151.61
147.34
145.26
142.81
127.68
CDCl3
77.00
43.67
33.13
28.94
200
180
160
140
120
100
80
60
40
20
0

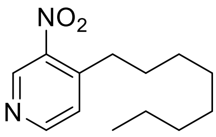

4e

^{1}H NMR (400 MHz, CDCl$_3$)

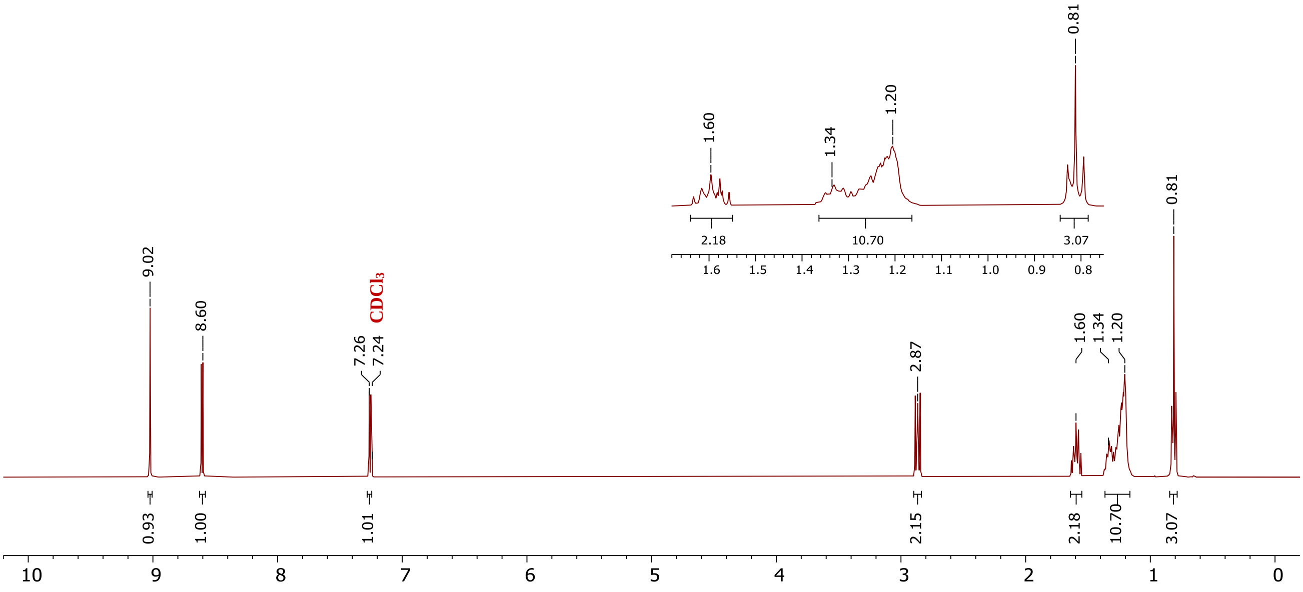

^{13}C NMR (100 MHz, CDCl$_3$)

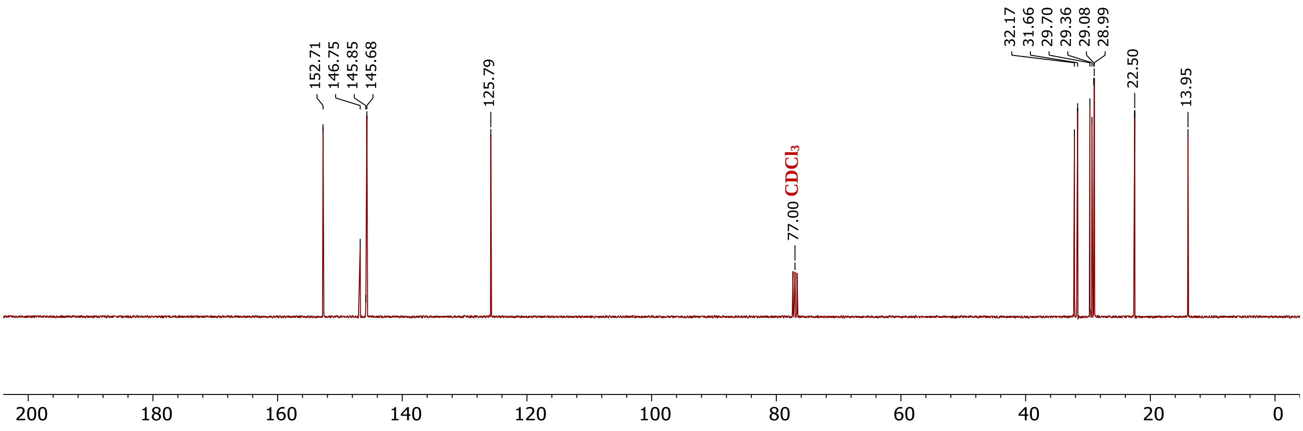

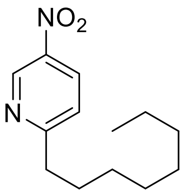

4e'

^{1}H NMR (400 MHz, CDCl3)

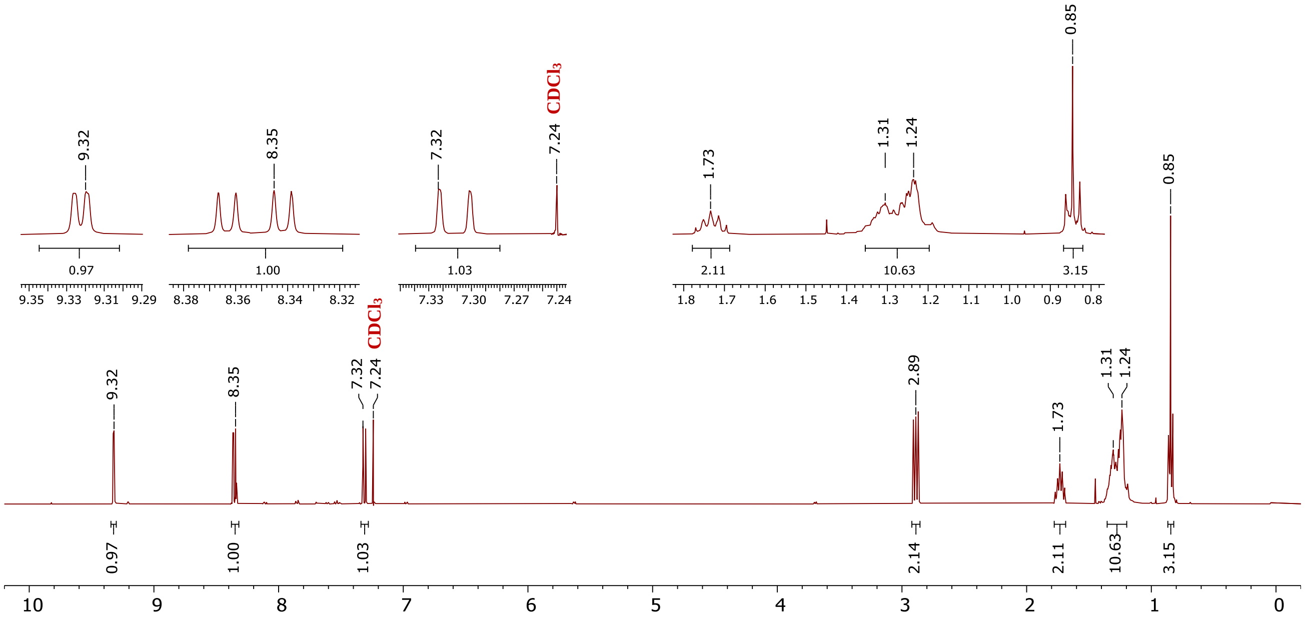

^{13}C NMR (100 MHz, CDCl3)

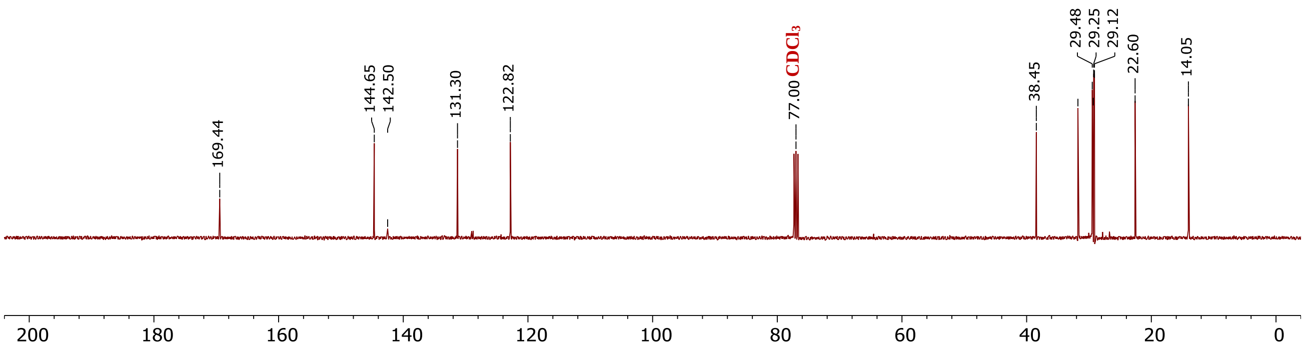

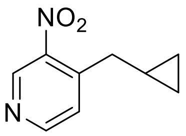

4f

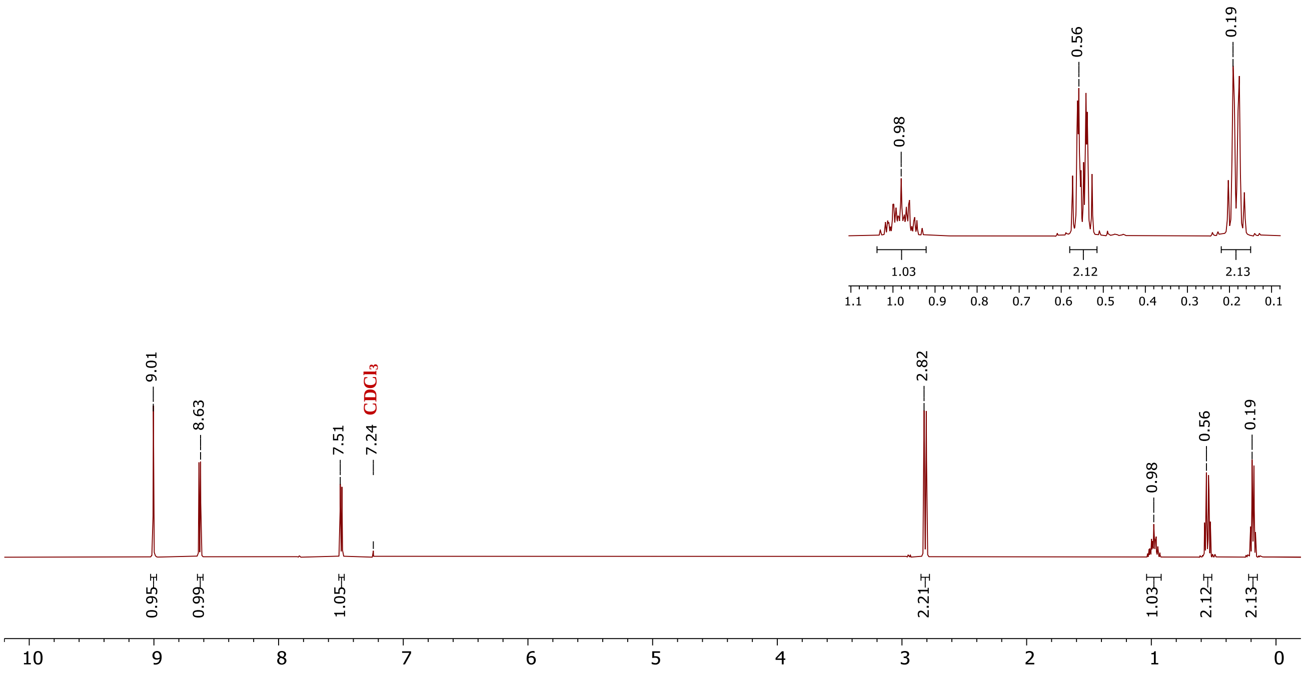

¹H NMR (400 MHz, CDCl₃)
9.01
8.63
7.51
7.24 CDCl₃
2.82
0.98
0.56
0.19
0.95
0.99
1.05
2.21
1.03
2.12
2.13

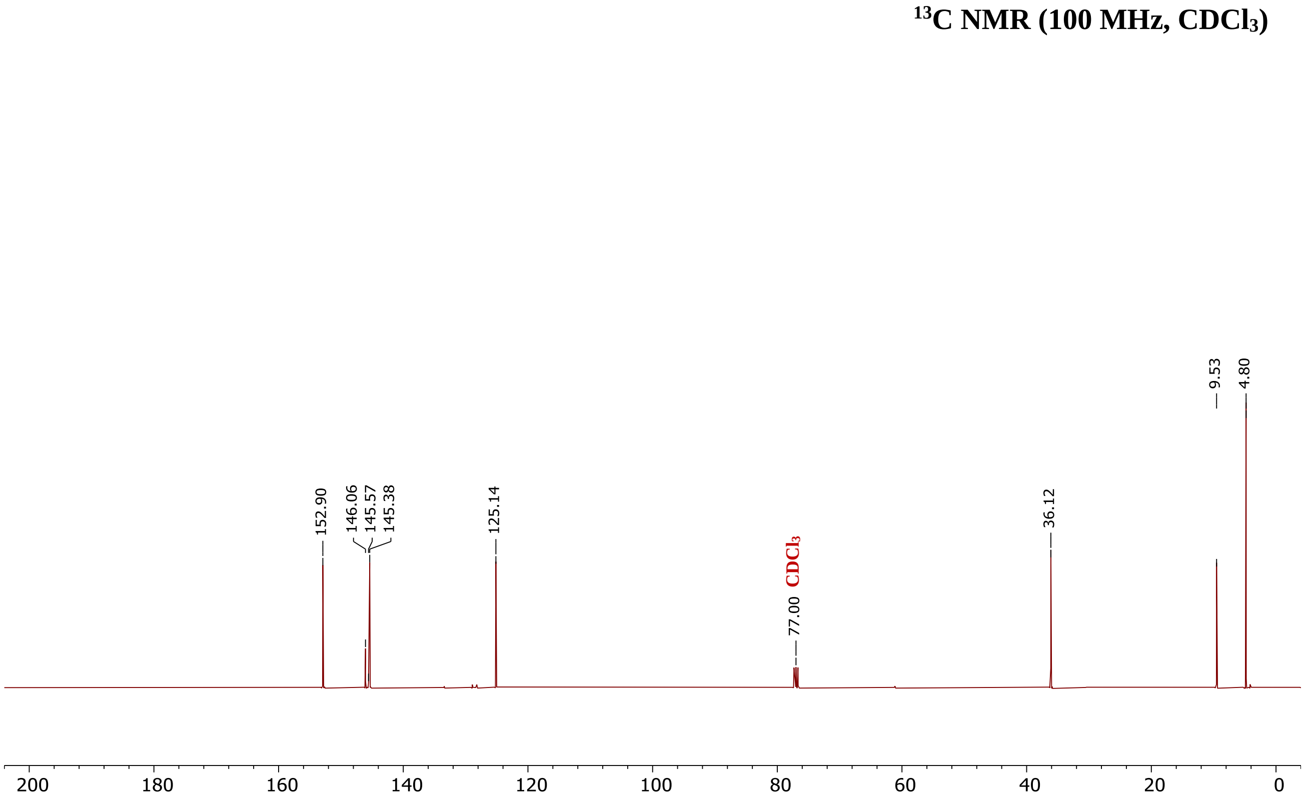

¹³C NMR (100 MHz, CDCl₃)
152.90
146.06
145.57
145.38
125.14
77.00 CDCl₃
36.12
9.53
4.80

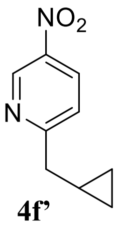

4f'

^{1}H NMR (400 MHz, CDCl$_3$)

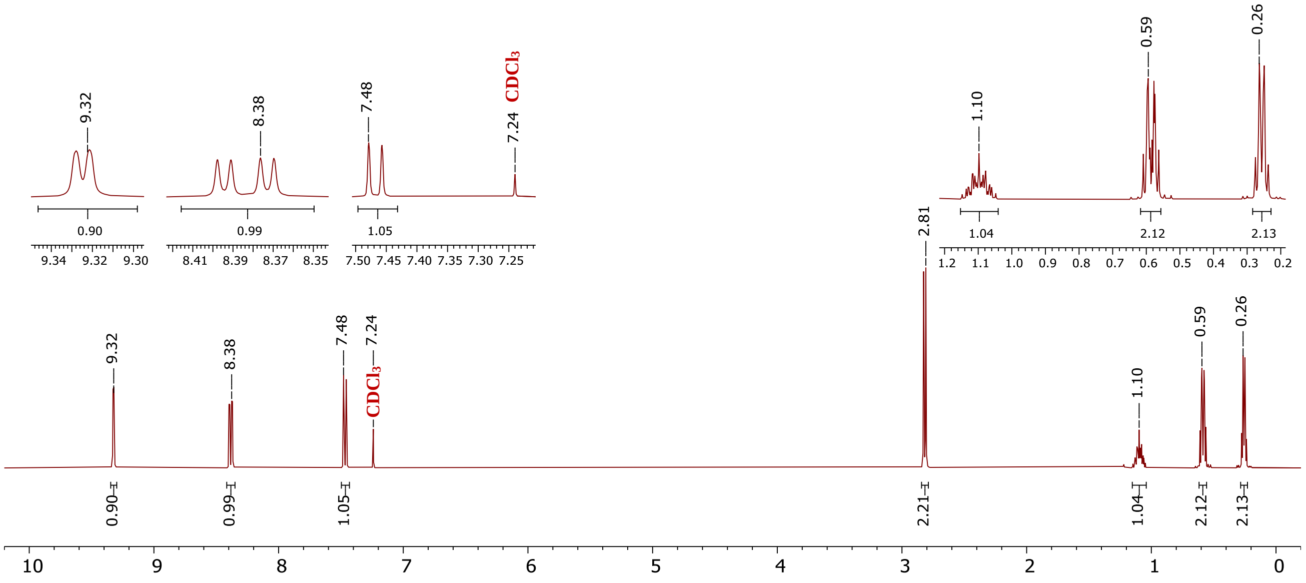

^{13}C NMR (100 MHz, CDCl$_3$)

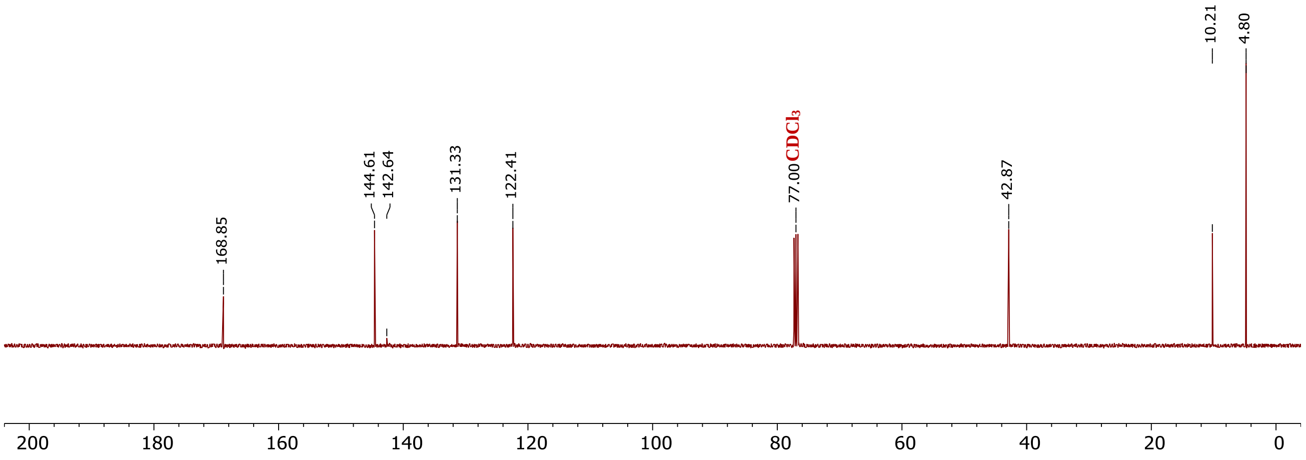

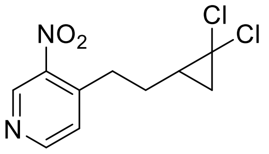

4g

^{1}H NMR (400 MHz, CDCl$_3$)

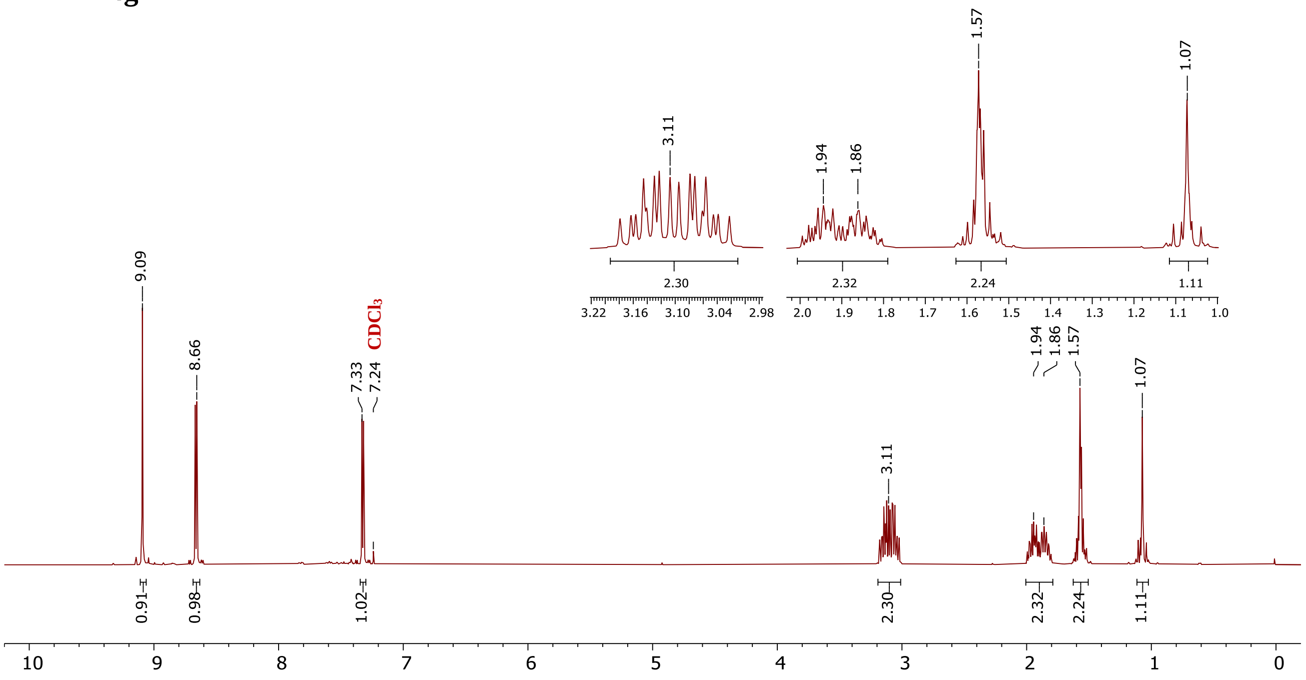

^{13}C NMR (100 MHz, CDCl$_3$)

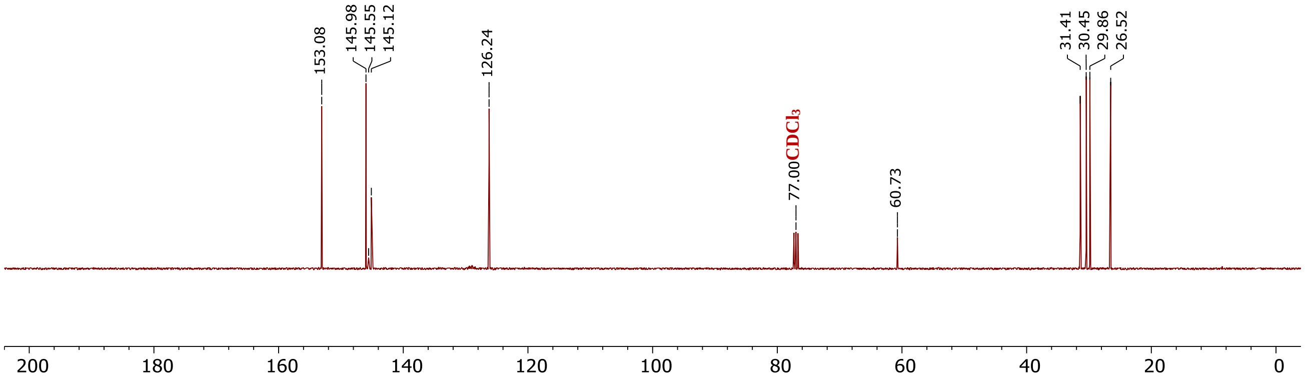

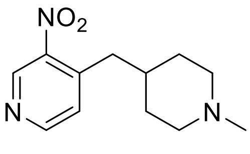

4h

^{1}H NMR (400 MHz, CDCl$_3$)

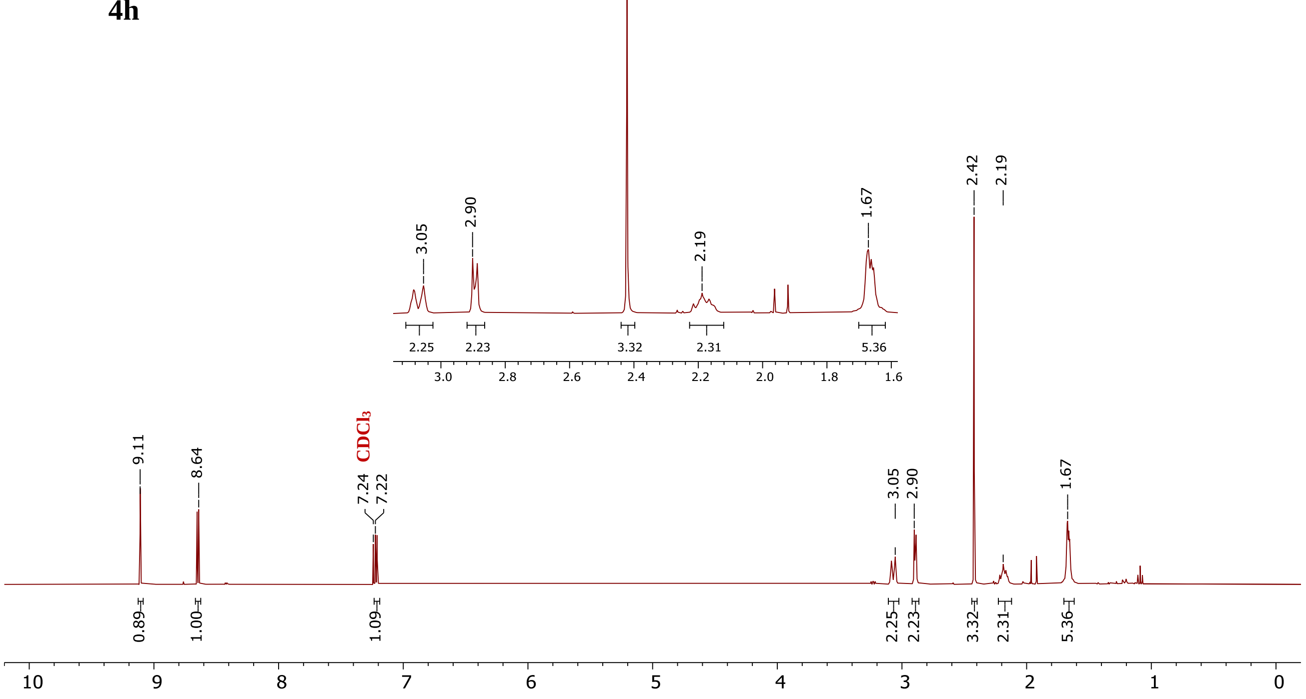

^{13}C NMR (100 MHz, CDCl$_3$)

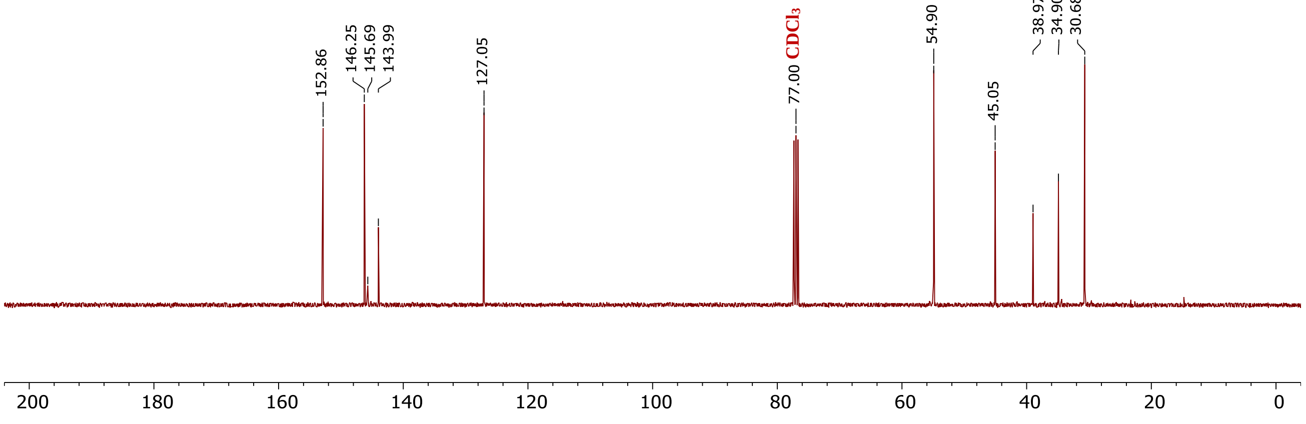

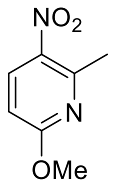
NO2
N
OMe

4i

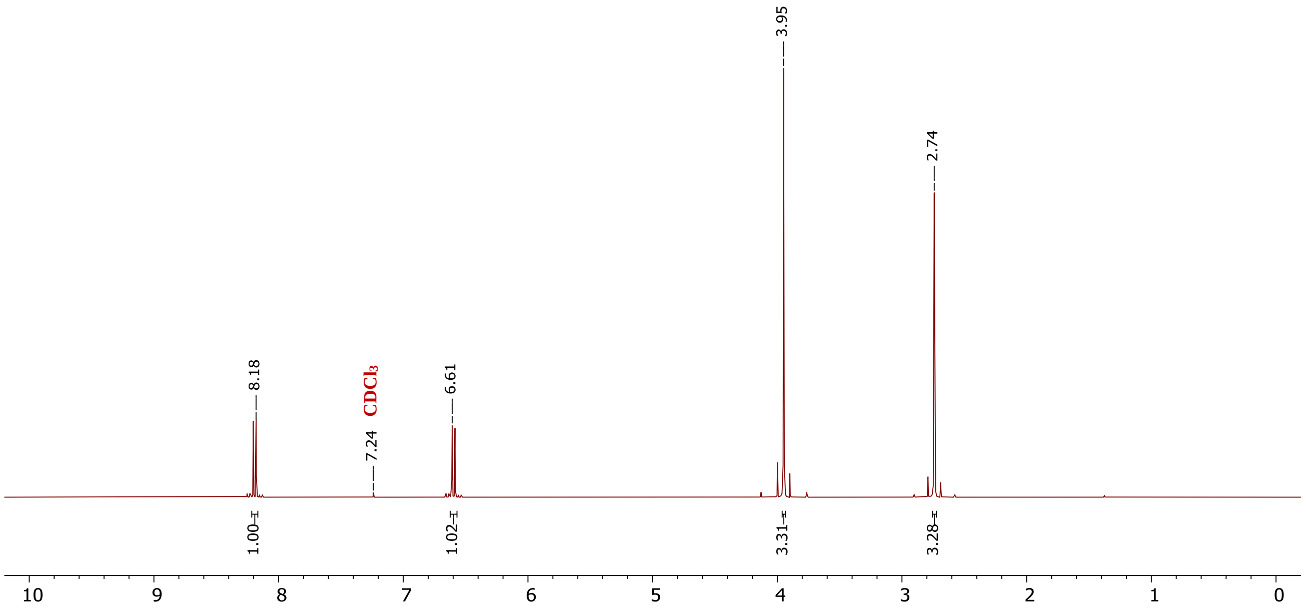
1H NMR (400 MHz, CDCl3)
8.18
CDCl3
7.24
6.61
3.95
2.74
1.00
1.02
3.31
3.28
10
9
8
7
6
5
4
3
2
1
0

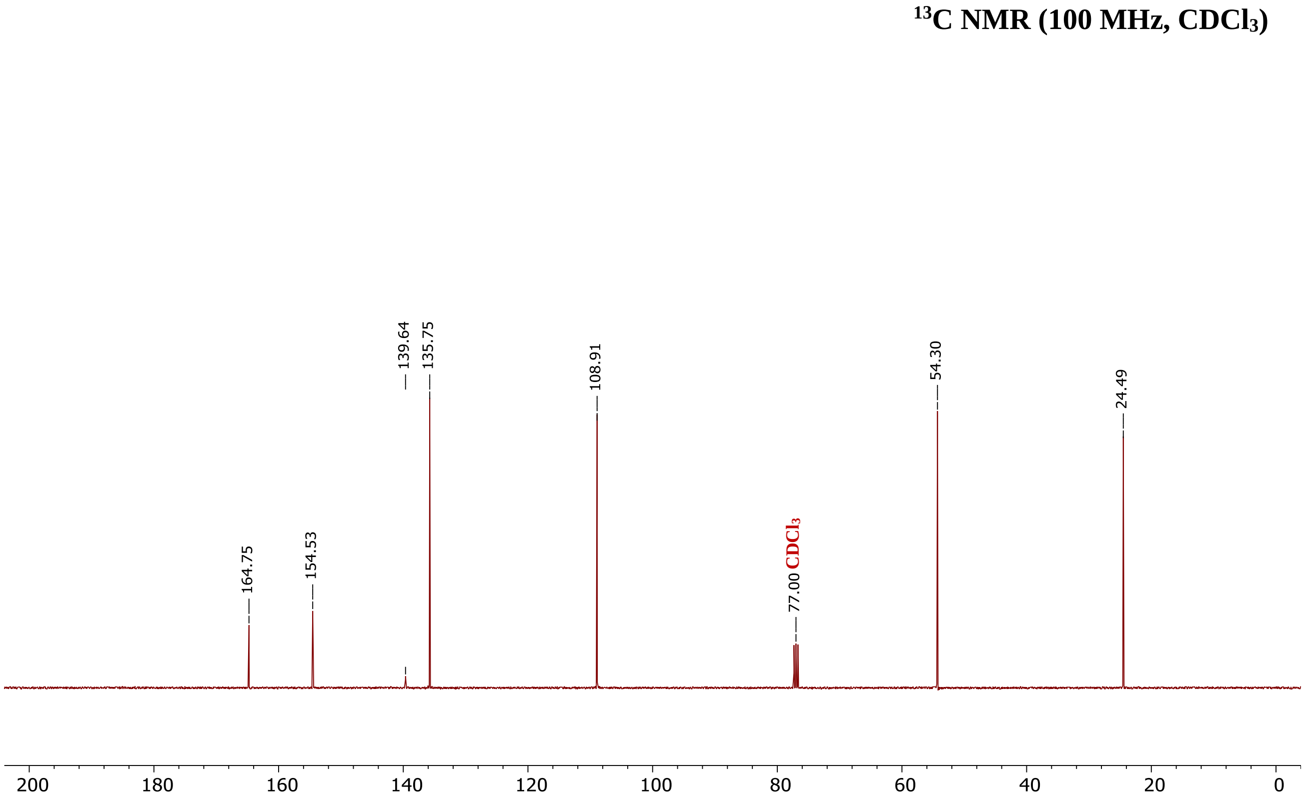
13C NMR (100 MHz, CDCl3)
164.75
154.53
139.64
135.75
108.91
77.00 CDCl3
54.30
24.49
200
180
160
140
120
100
80
60
40
20
0

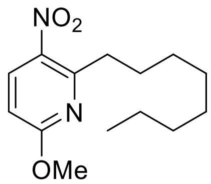

4j

^{1}H NMR (400 MHz, CDCl$_3$)

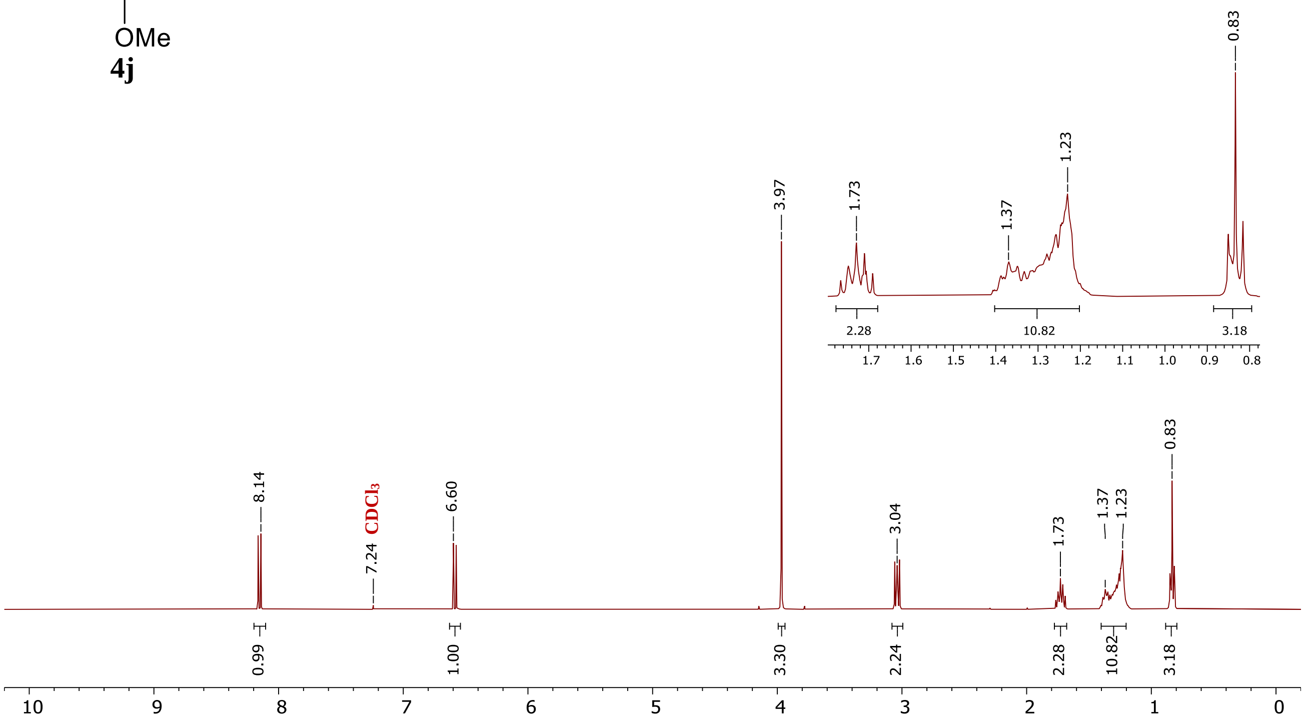

^{13}C NMR (100 MHz, CDCl$_3$)

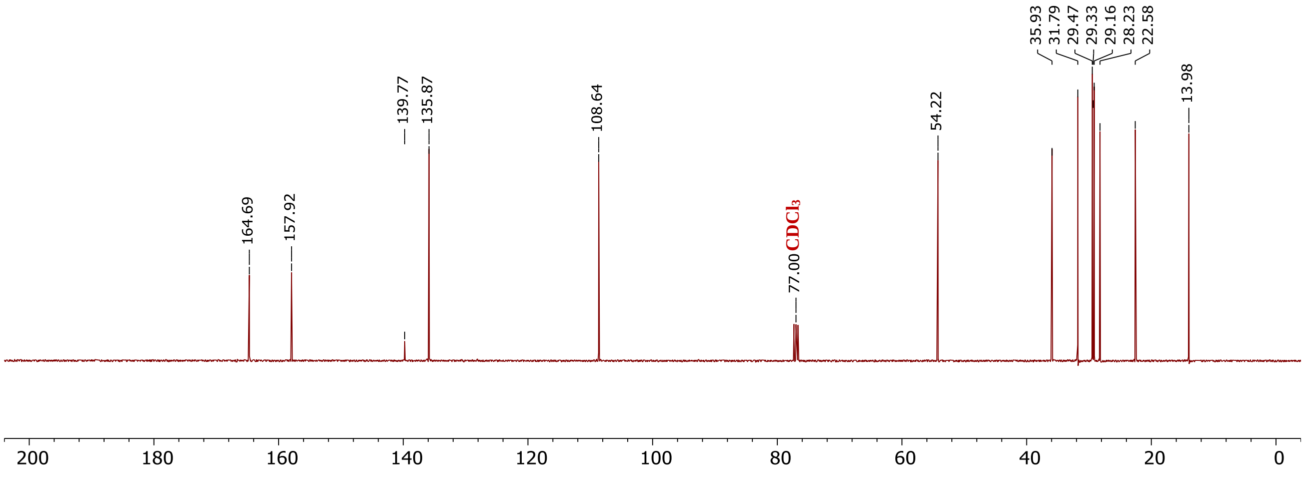

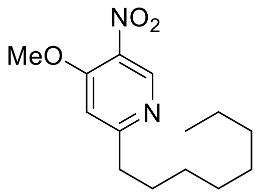

4k

^{1}H NMR (400 MHz, CDCl$_3$)

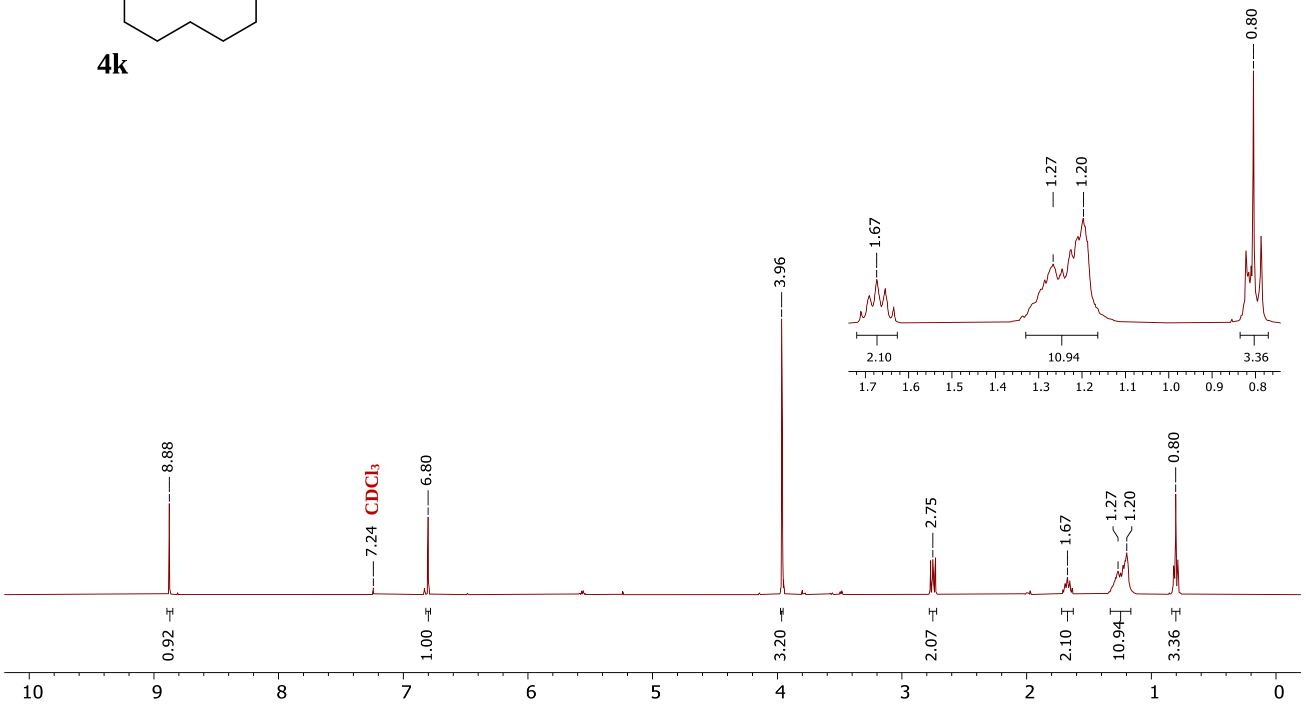

^{13}C NMR (100 MHz, CDCl$_3$)

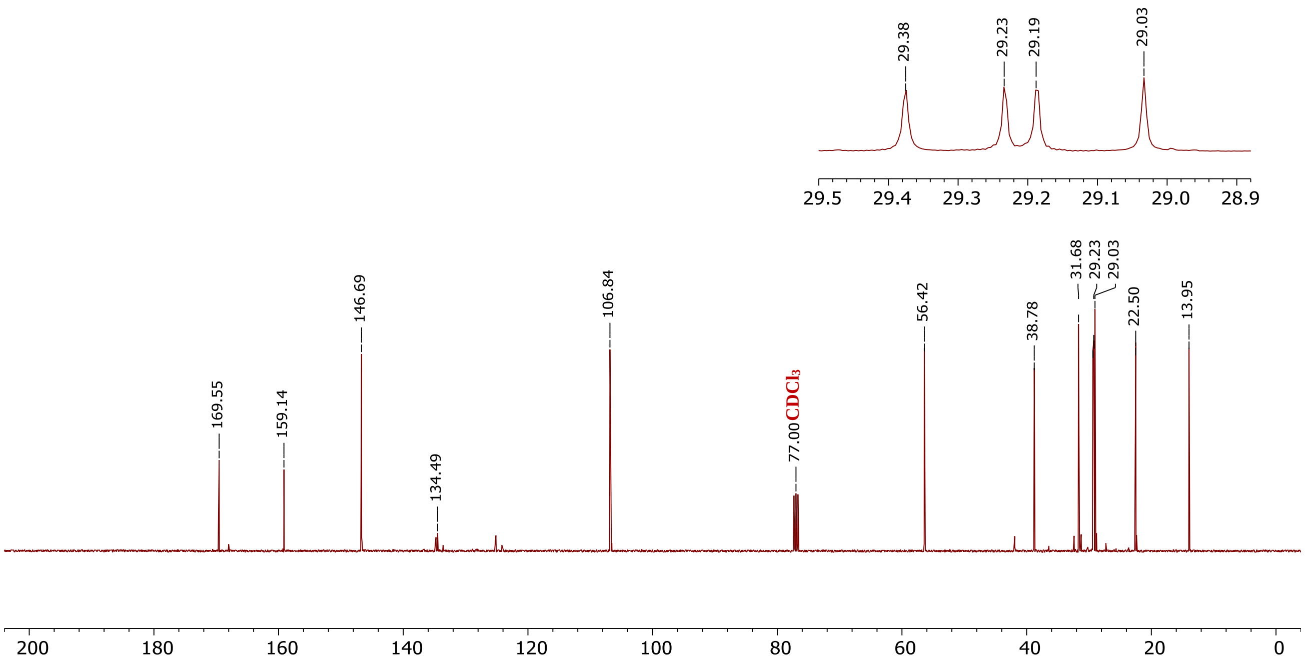

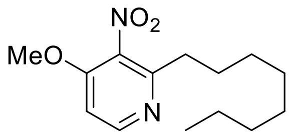

4k'

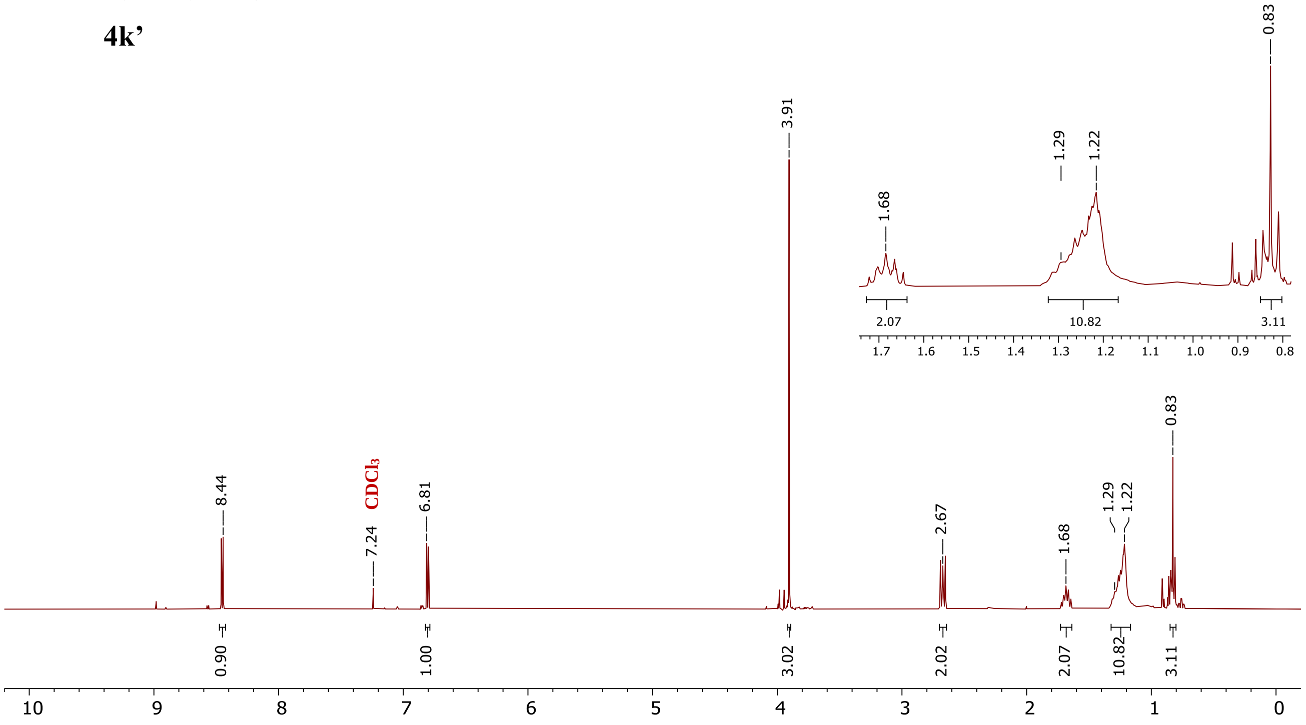

^{1}H NMR (400 MHz, CDCl3)
8.44
CDCl3
7.24
6.81
3.91
2.67
1.68
1.29
1.22
0.83
0.90
1.00
3.02
2.02
2.07
10.82
3.11

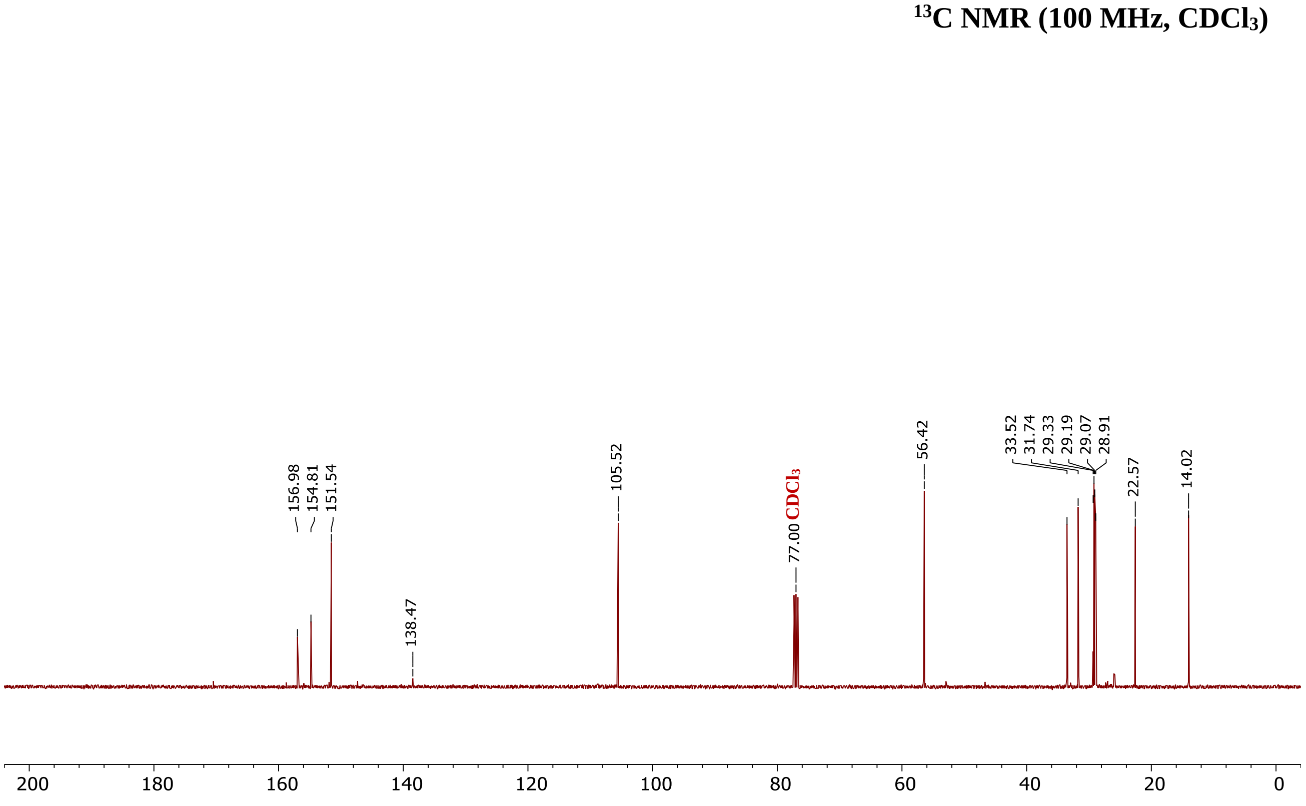

^{13}C NMR (100 MHz, CDCl3)
156.98
154.81
151.54
138.47
105.52
77.00 CDCl3
56.42
33.52
31.74
29.33
29.19
29.07
28.91
22.57
14.02

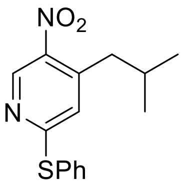

4l

^{1}H NMR (400 MHz, CDCl$_3$)

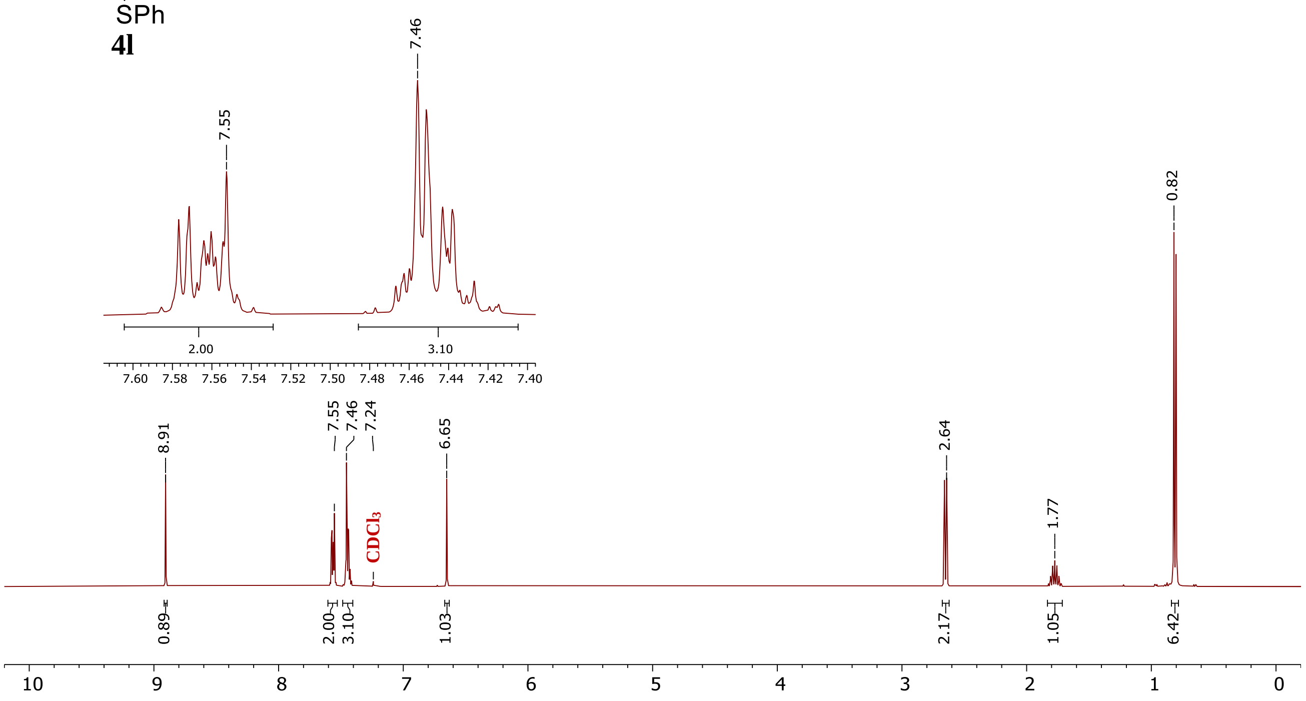

^{13}C NMR (100 MHz, CDCl$_3$)

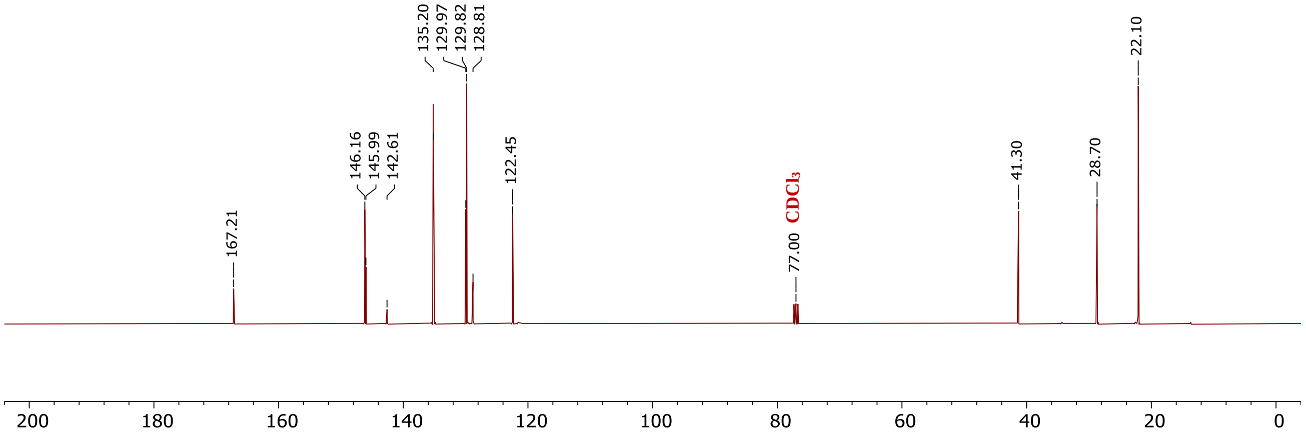

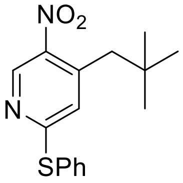

4m

^{1}H NMR (400 MHz, $CDCl_3$)

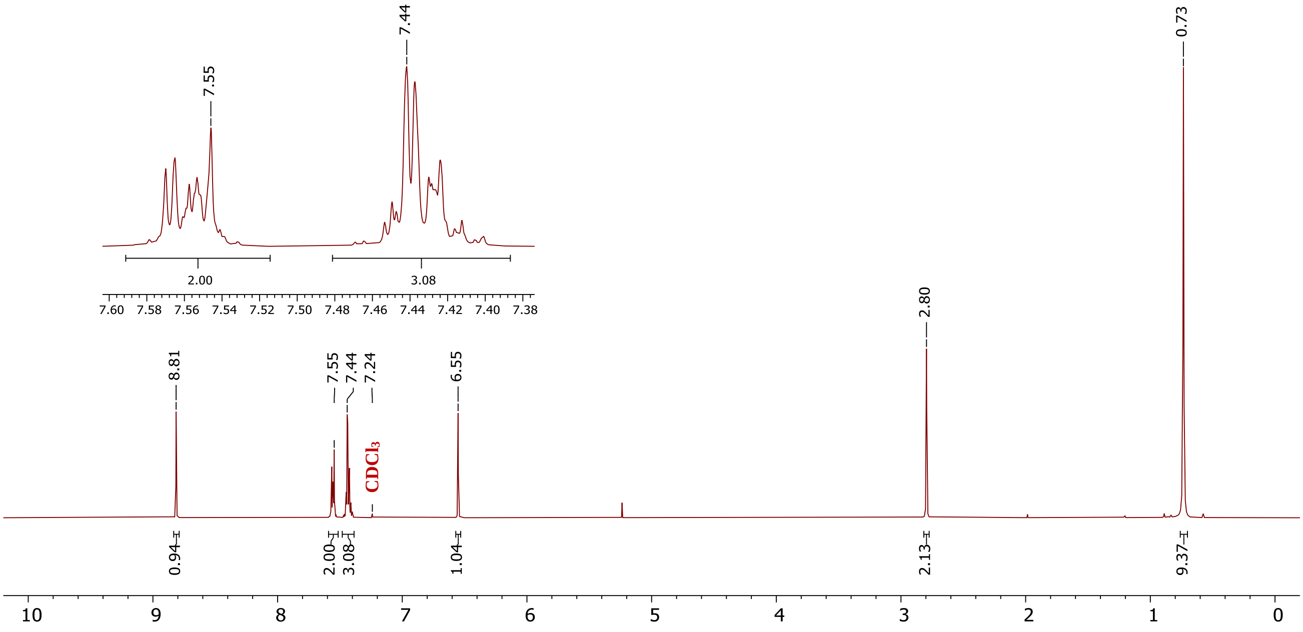

^{13}C NMR (100 MHz, $CDCl_3$)

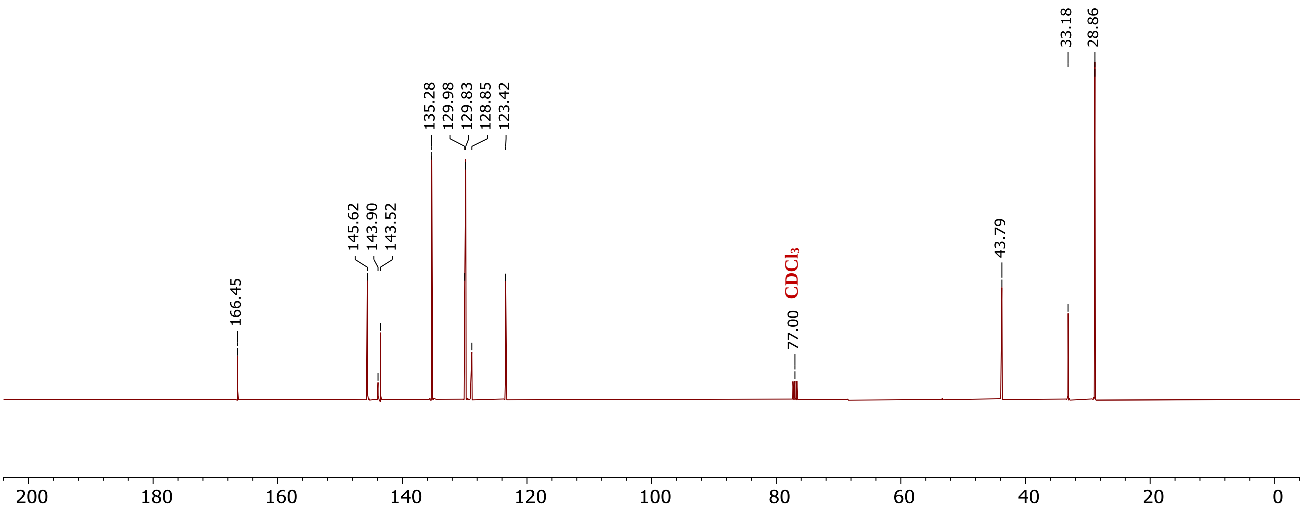

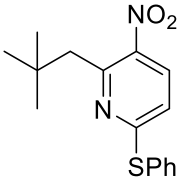

4m'

^{1}H NMR (400 MHz, $CDCl_3$)

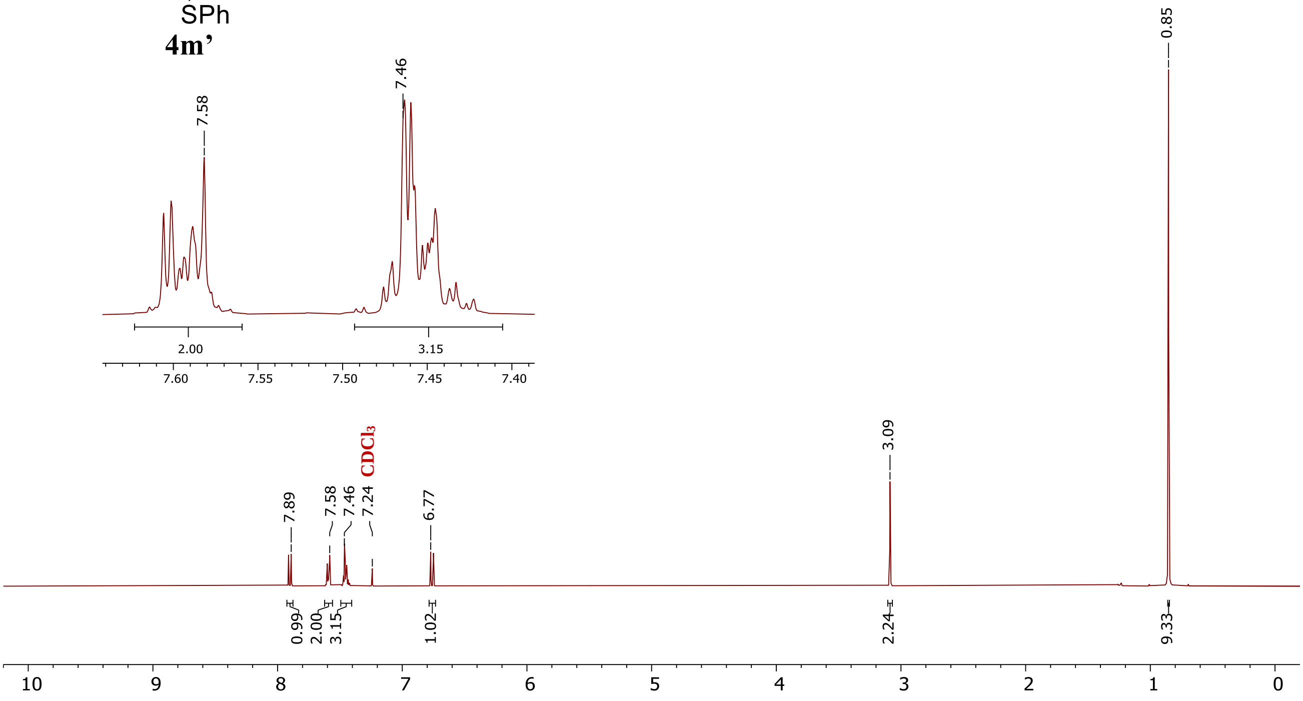

^{13}C NMR (100 MHz, $CDCl_3$)

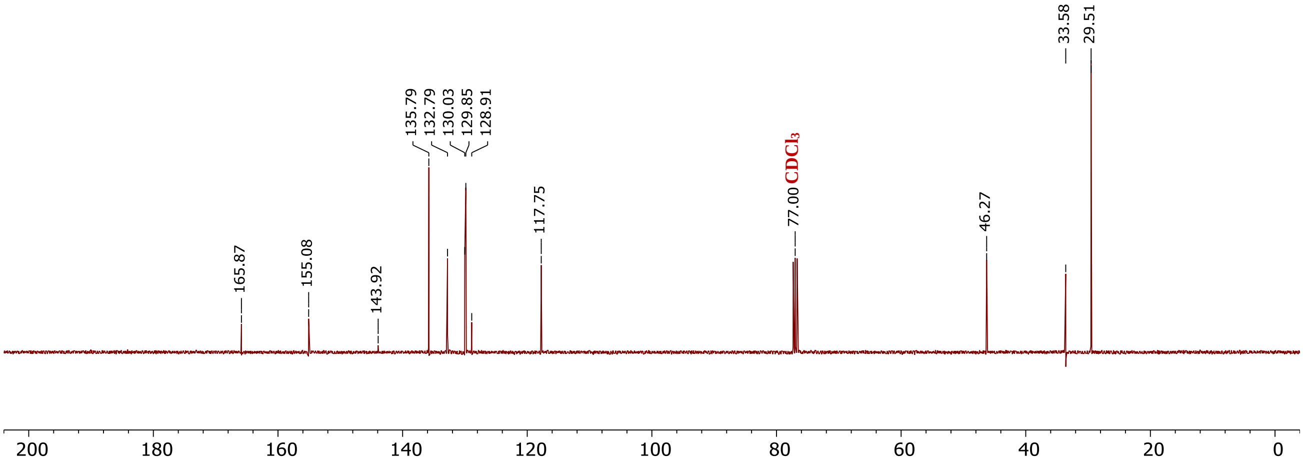

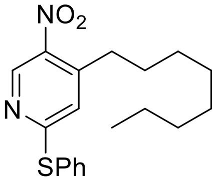

4n

^{1}H NMR (400 MHz, $CDCl_3$)

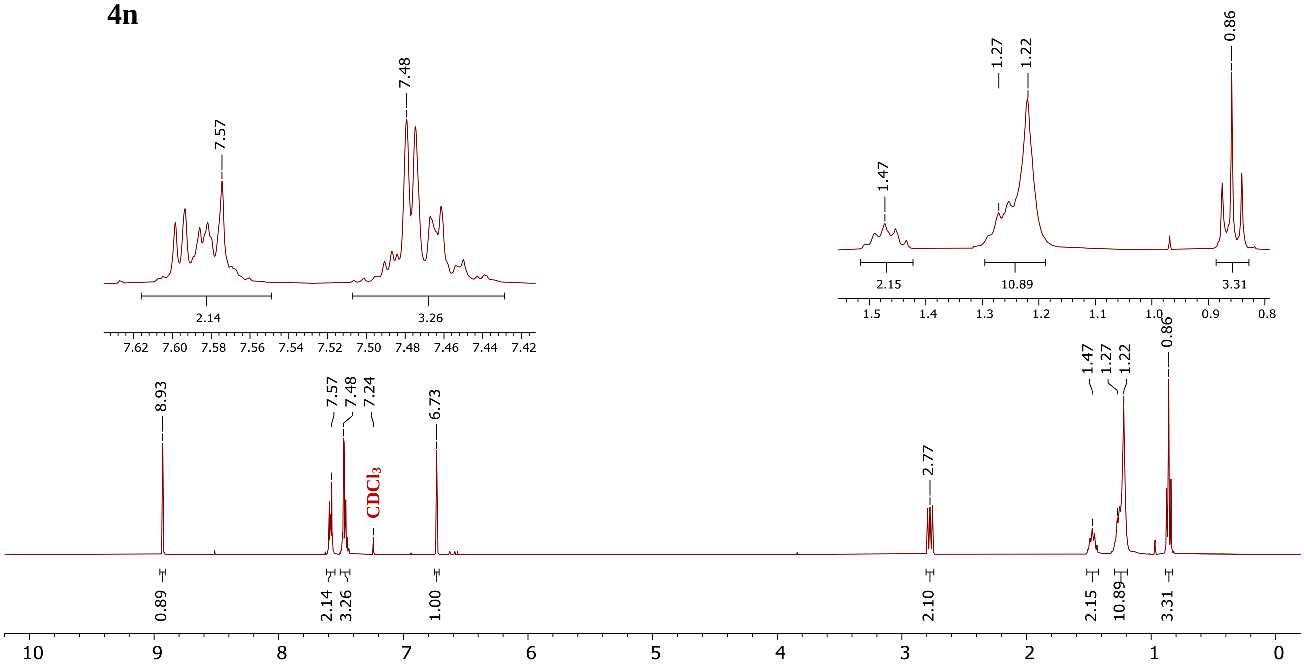

^{13}C NMR (100 MHz, $CDCl_3$)

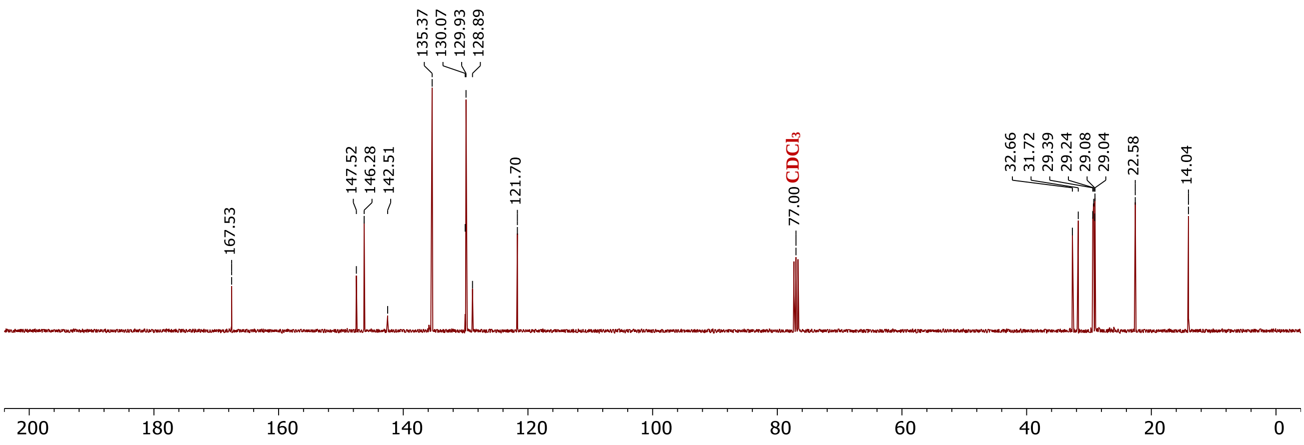

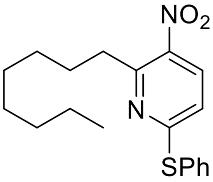

4n'

^{1}H NMR (400 MHz, CDCl$_3$)

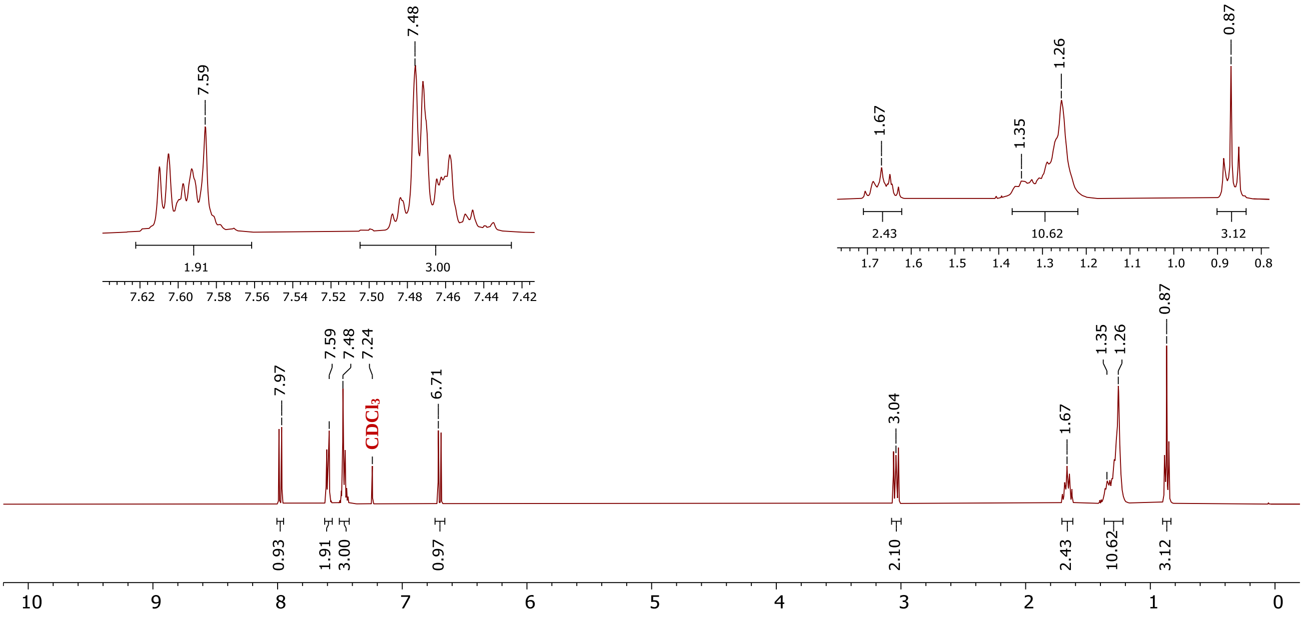

^{13}C NMR (100 MHz, CDCl$_3$)

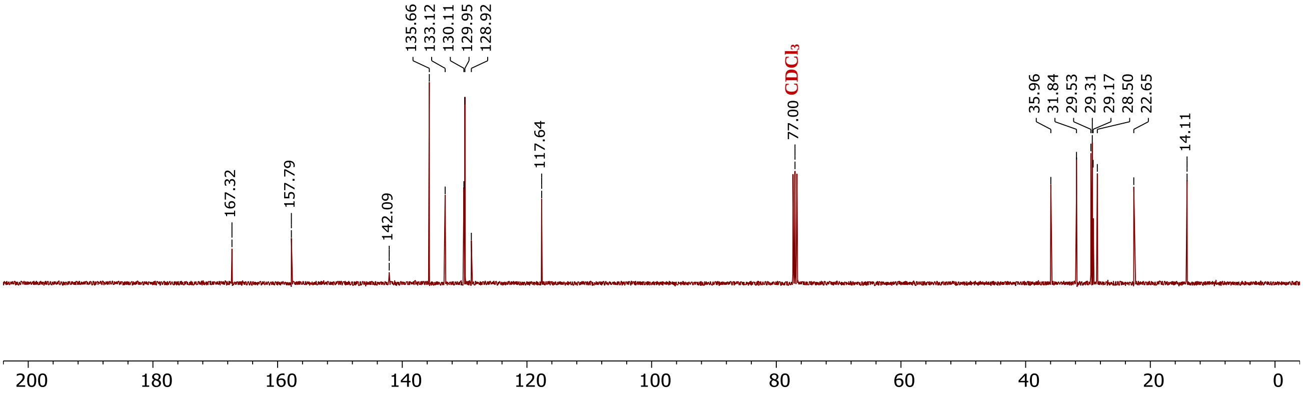

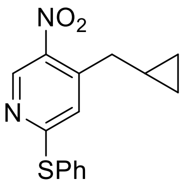

4o

^{1}H NMR (400 MHz, $CDCl_3$)

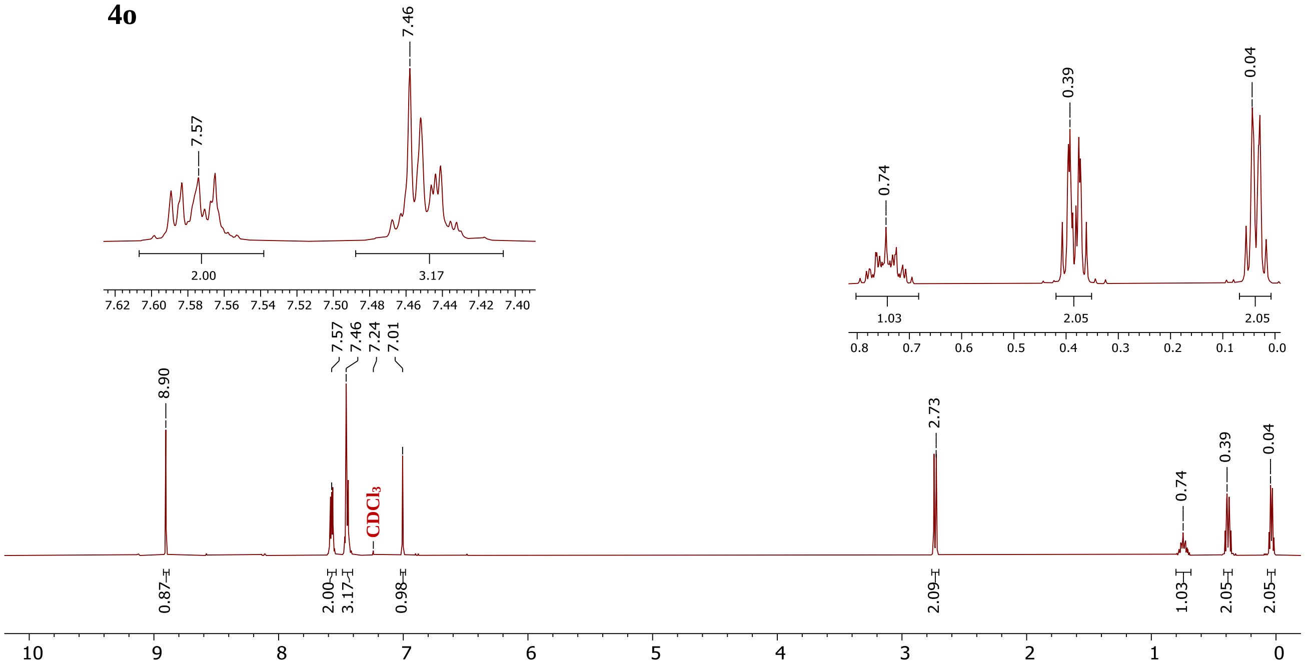

^{13}C NMR (100 MHz, $CDCl_3$)

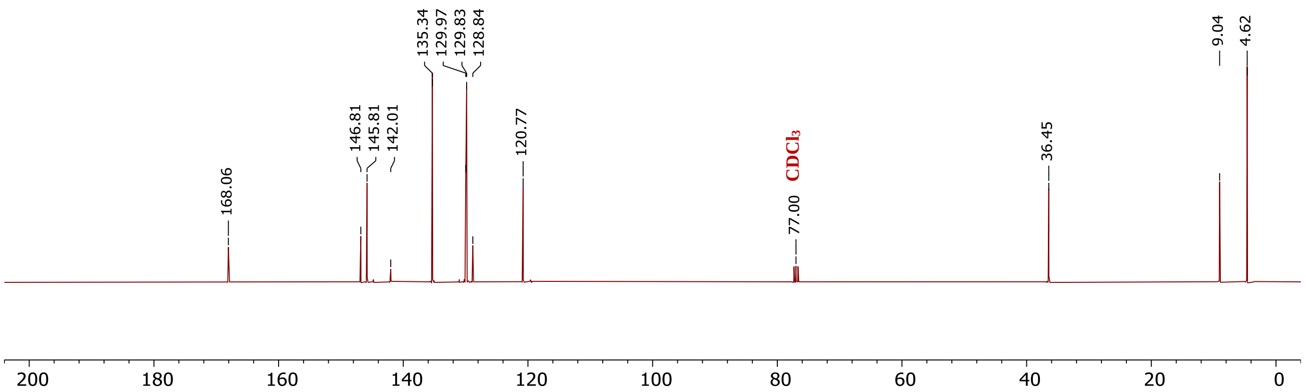

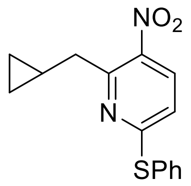

4o'

^{1}H NMR (400 MHz, $CDCl_3$)

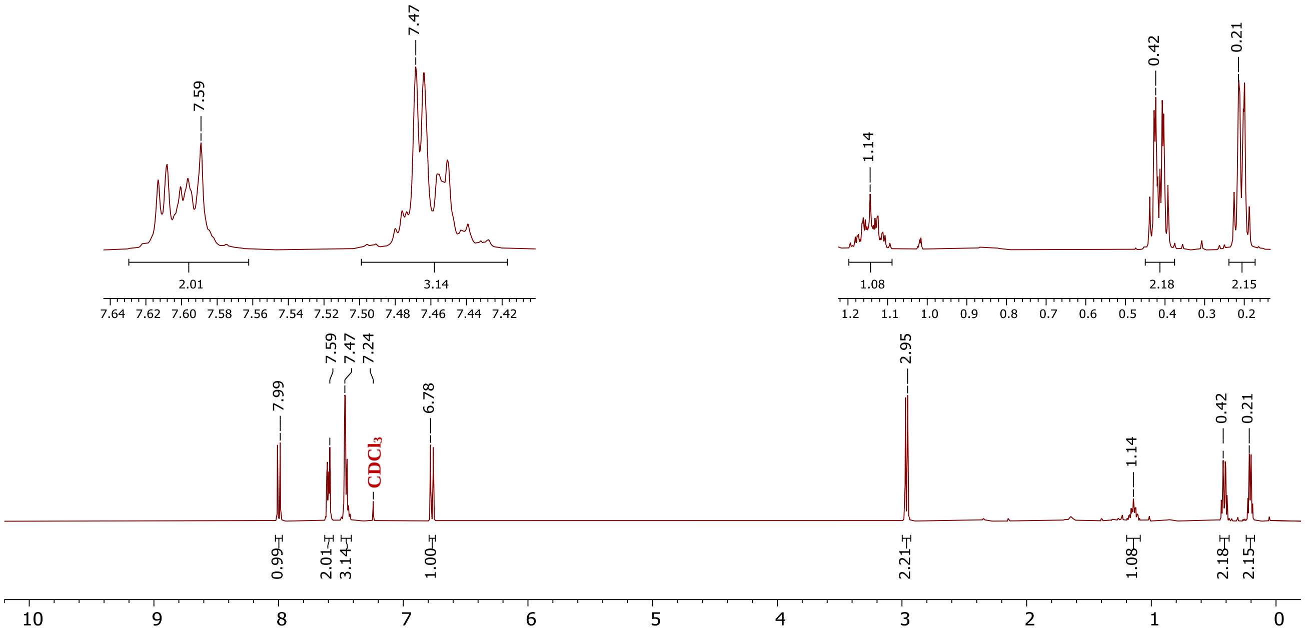

^{13}C NMR (100 MHz, $CDCl_3$)

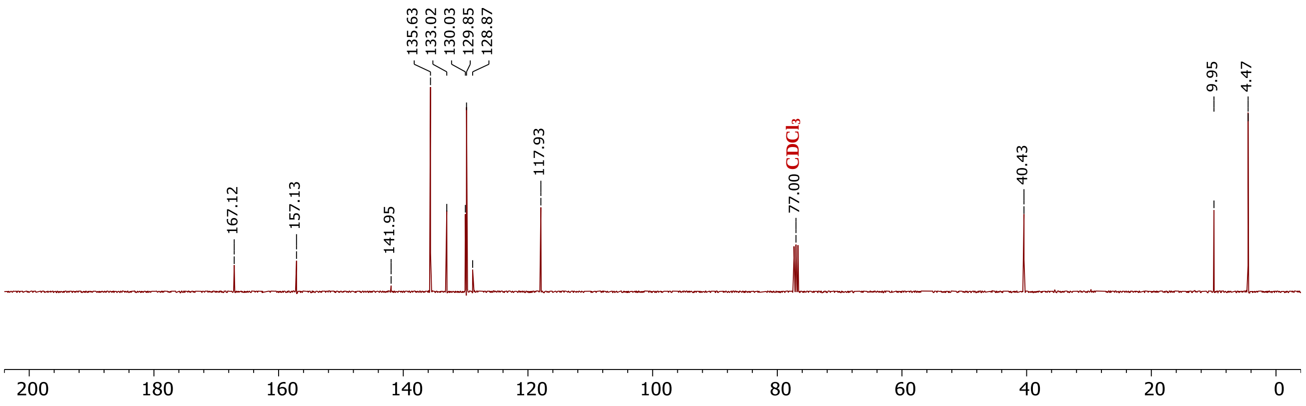

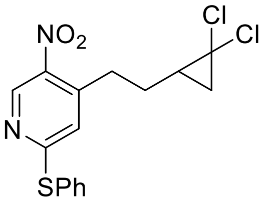

4p

^{1}H NMR (400 MHz, CDCl$_3$)

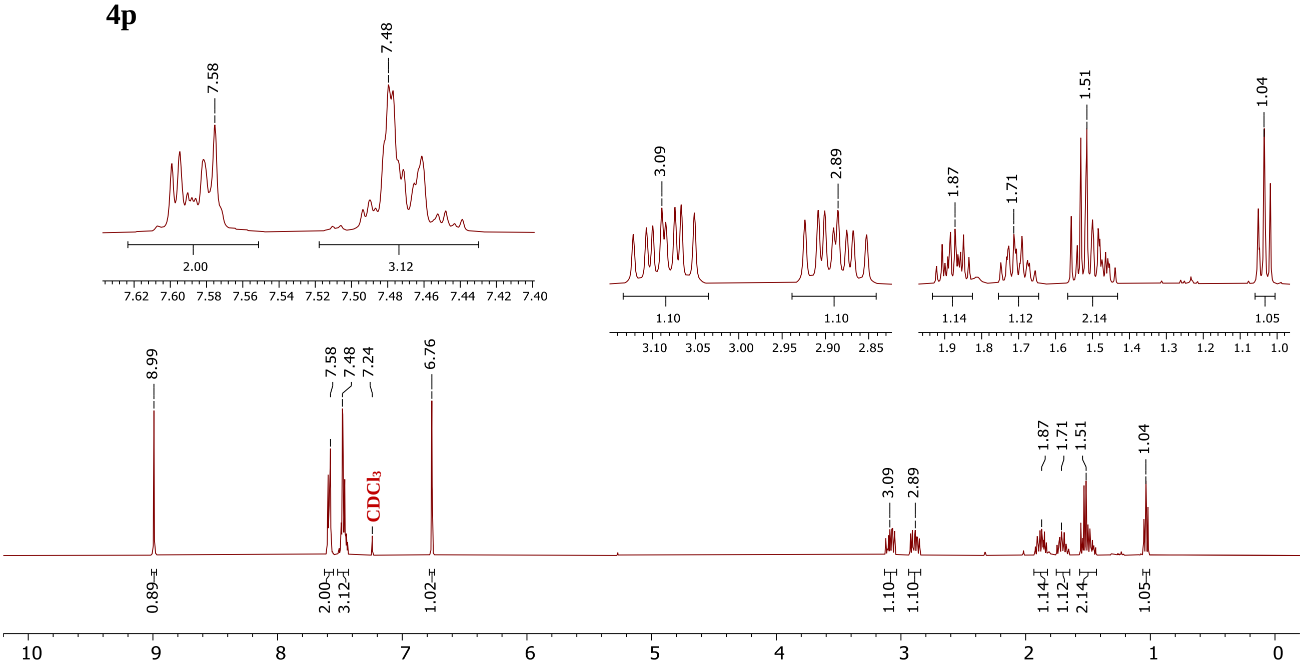

^{13}C NMR (100 MHz, CDCl$_3$)

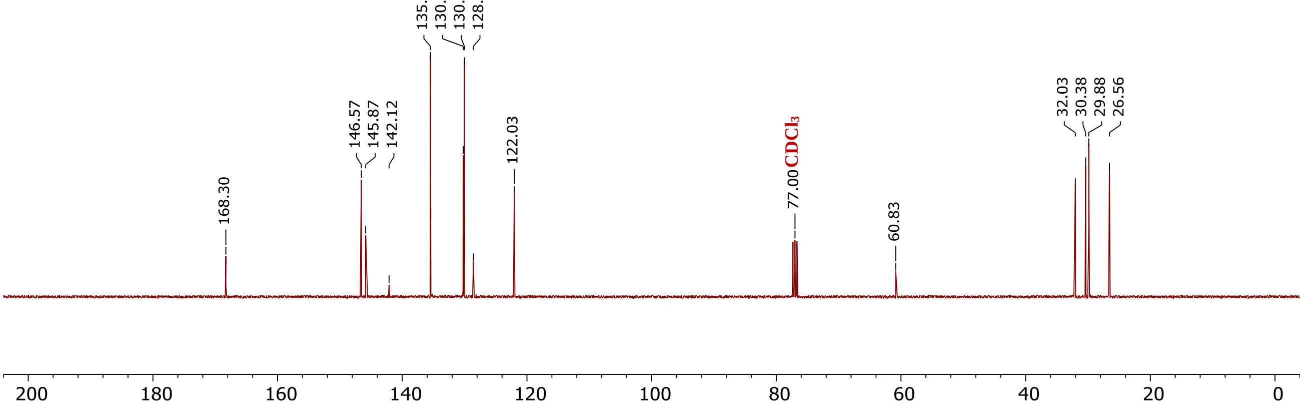

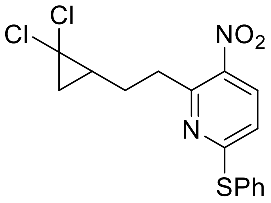

4p'

^{1}H NMR (400 MHz, CDCl3)

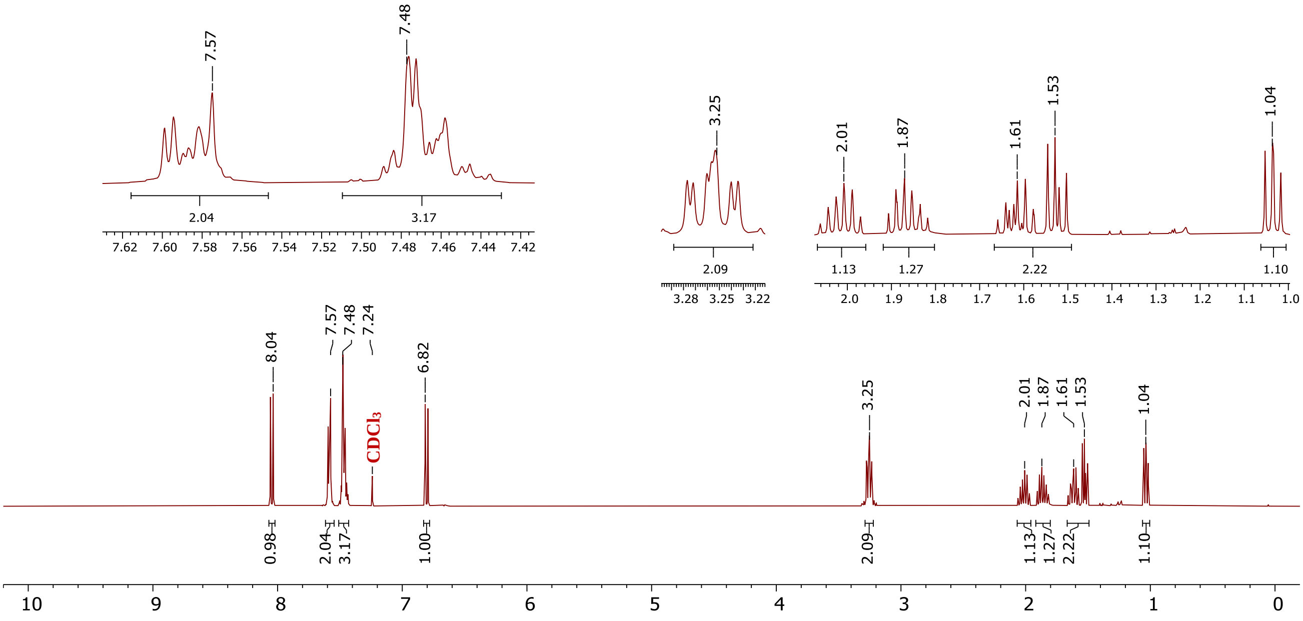

^{13}C NMR (100 MHz, CDCl3)

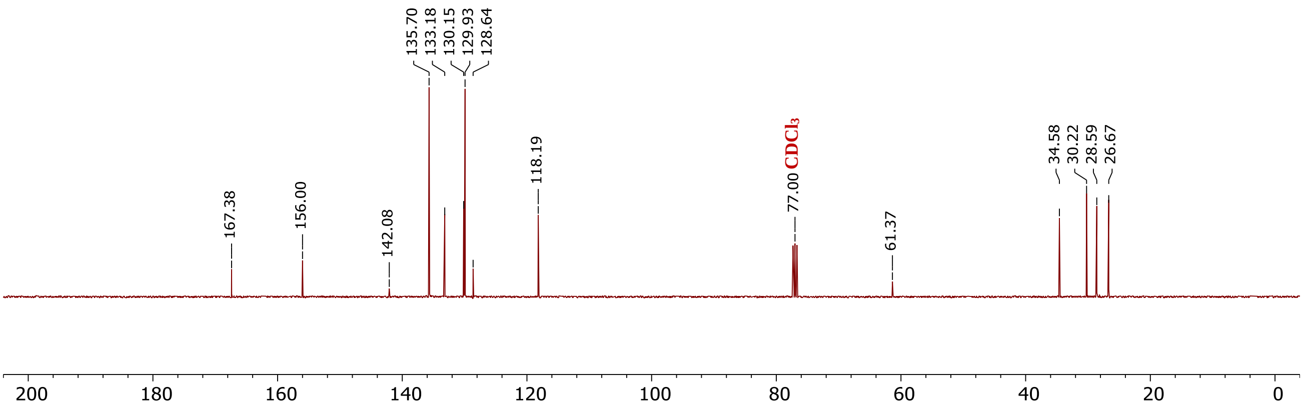

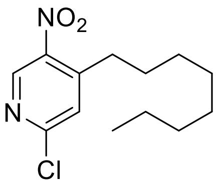

4q

^{1}H NMR (400 MHz, CDCl$_3$)

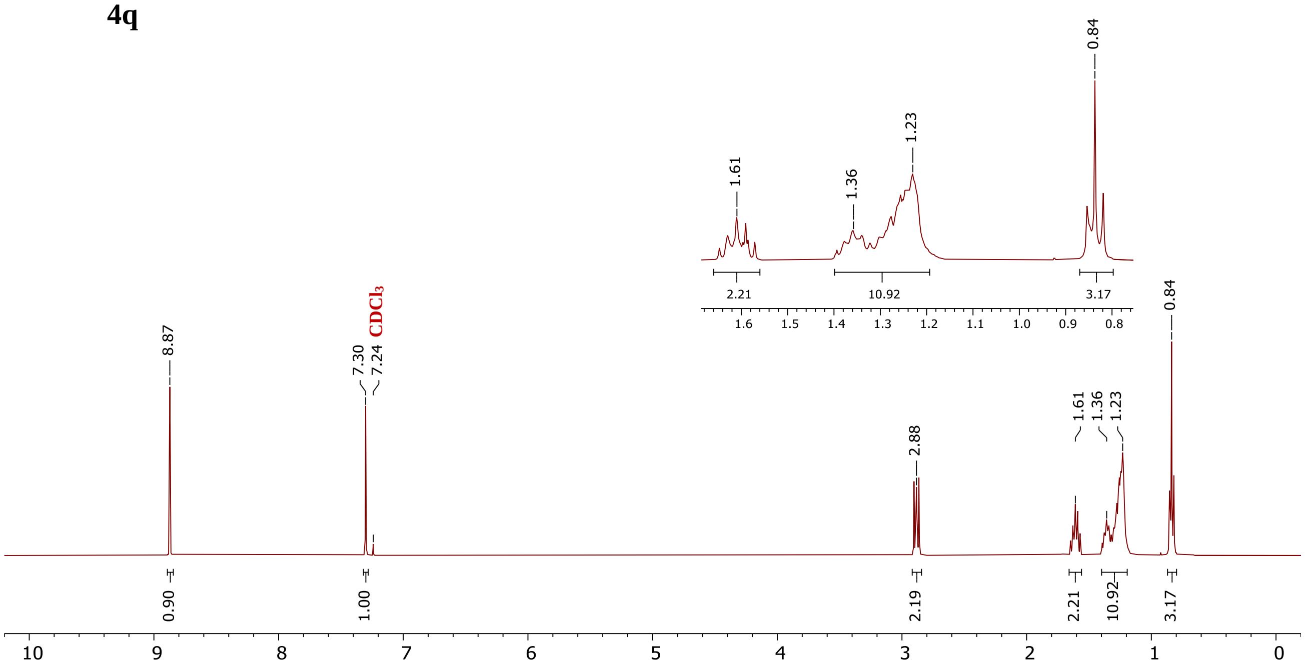

^{13}C NMR (100 MHz, CDCl$_3$)

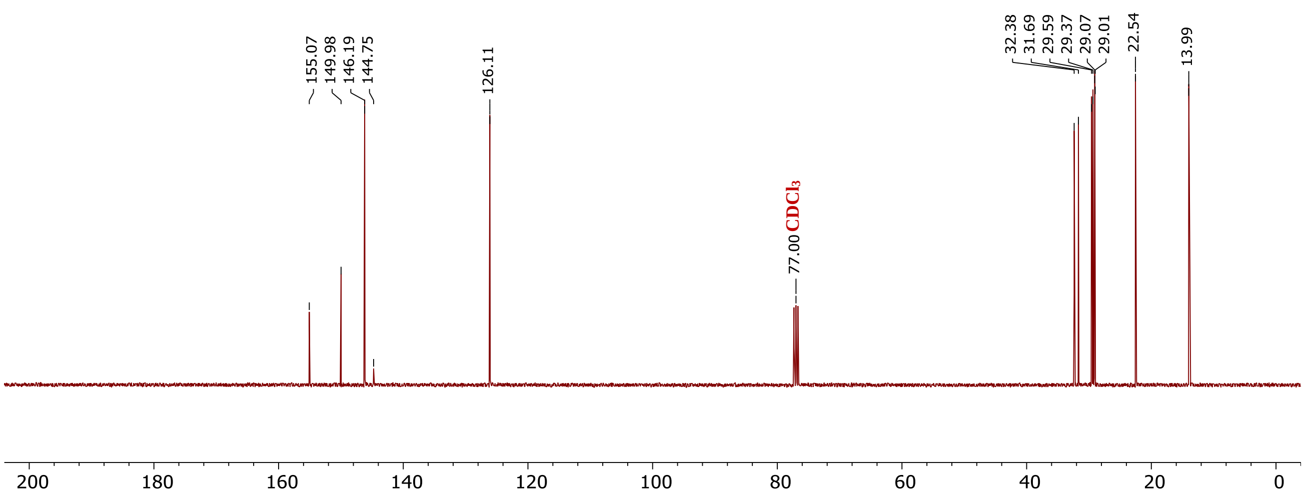

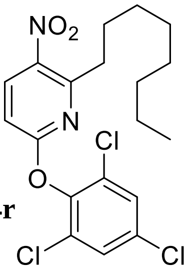

4r

^{1}H NMR (400 MHz, CDCl$_3$)

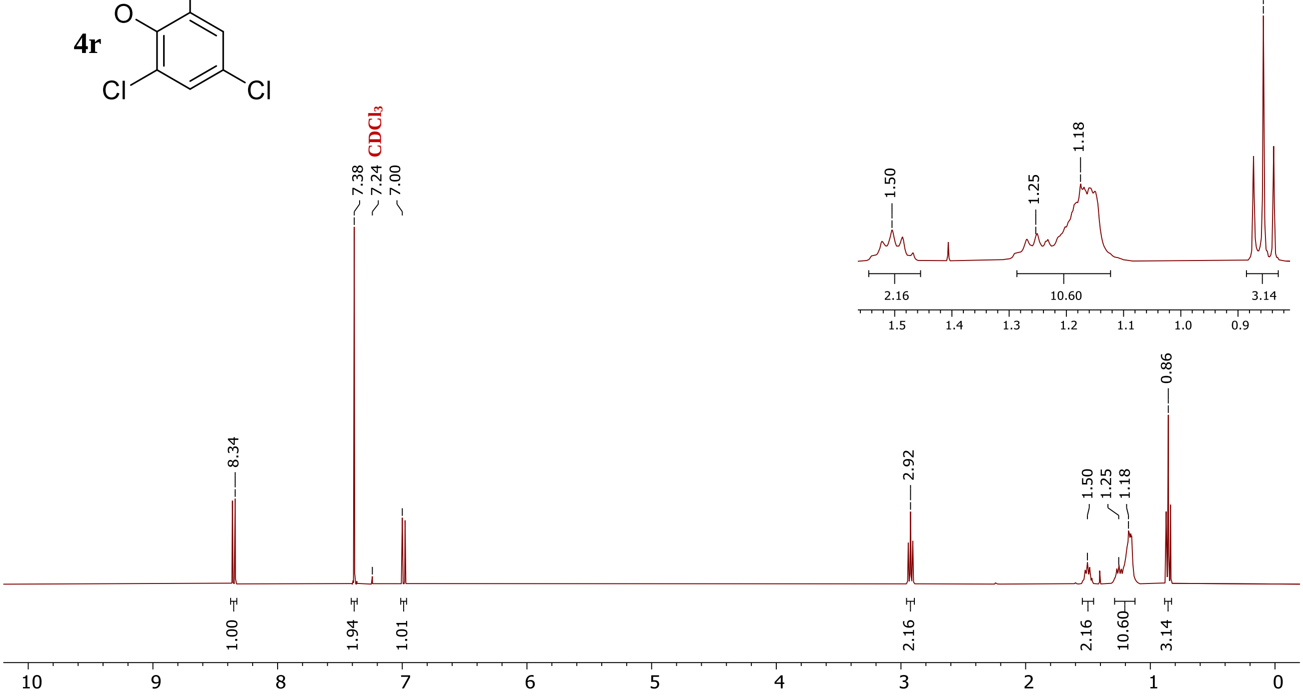

^{13}C NMR (100 MHz, CDCl$_3$)

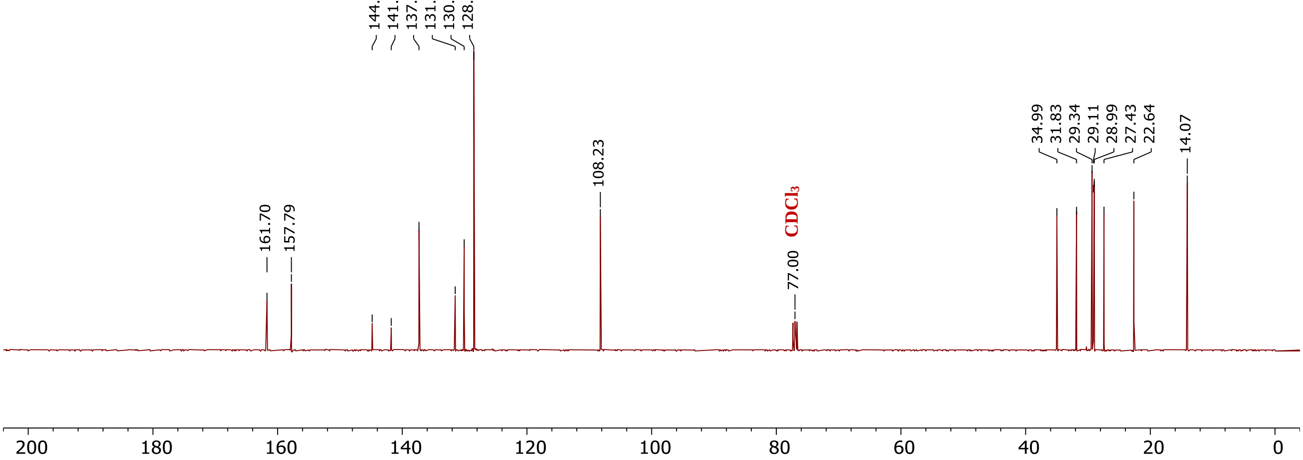

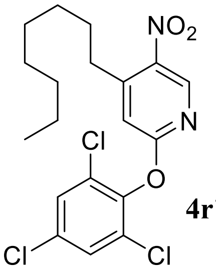

4r'

^{1}H NMR (400 MHz, CDCl$_3$)

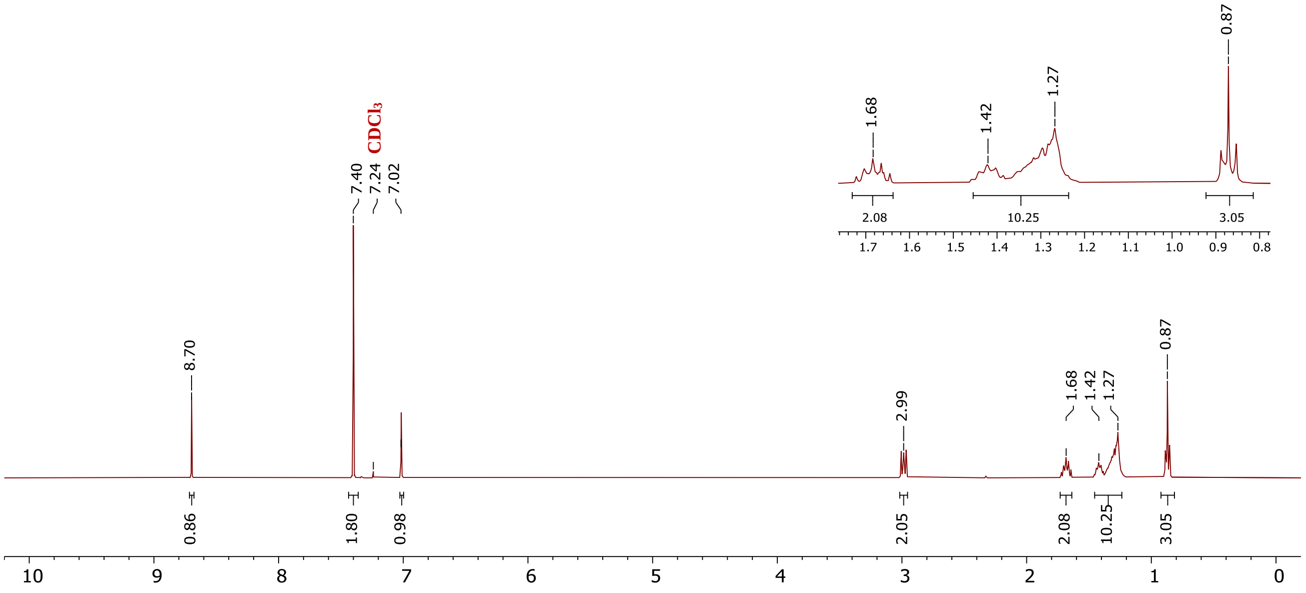

^{13}C NMR (100 MHz, CDCl$_3$)

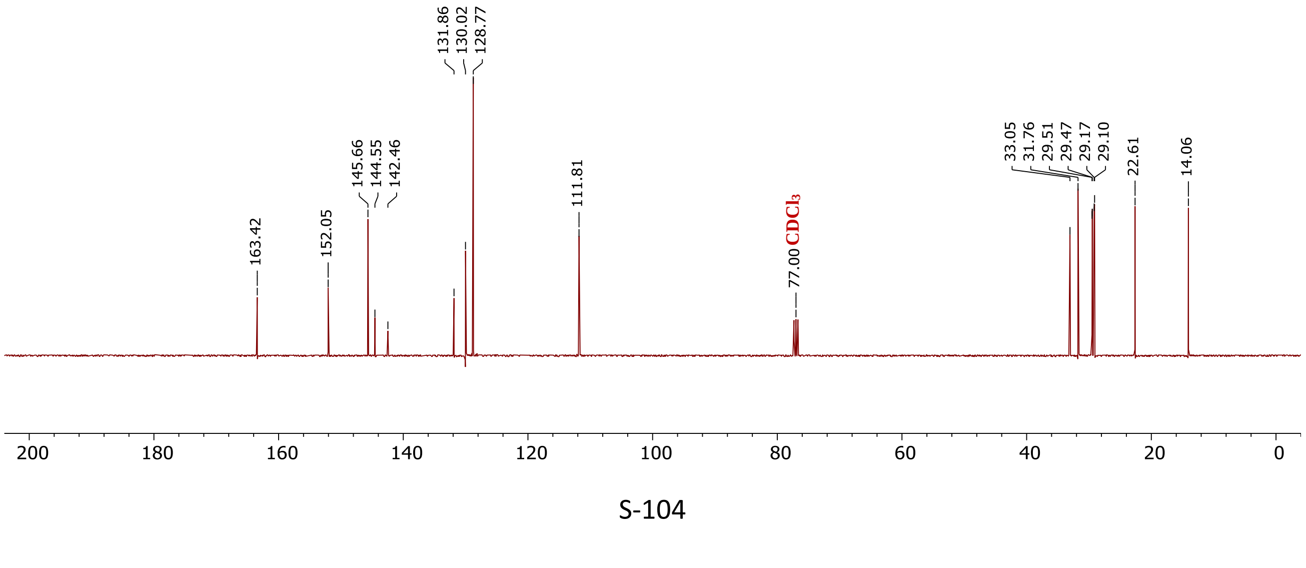

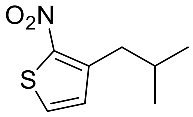

4s

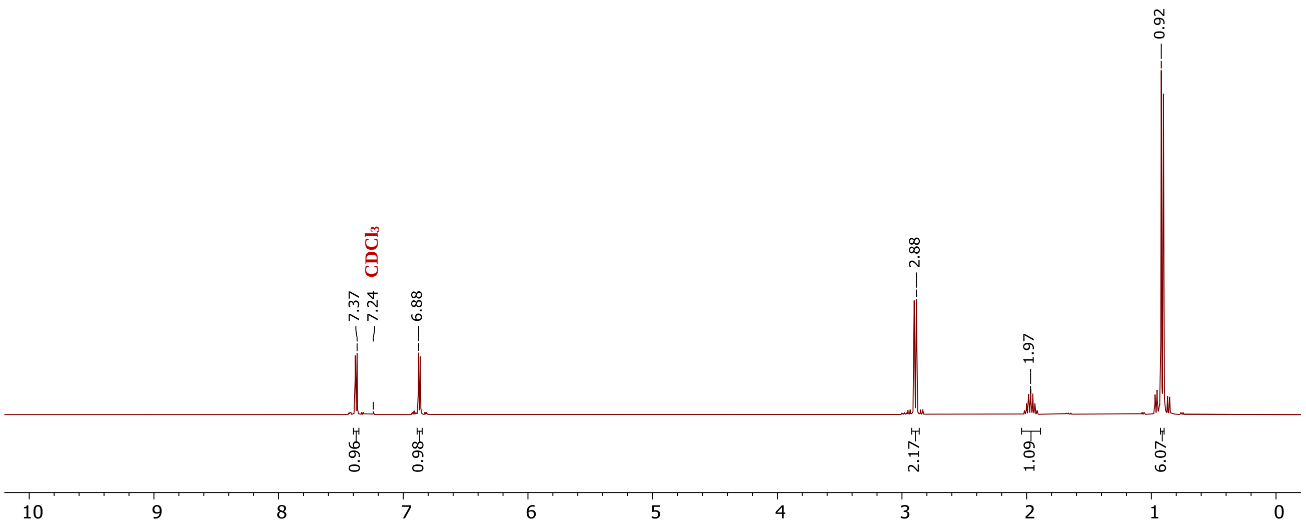

^{1}H NMR (400 MHz, CDCl$_3$)
CDCl₃
7.37
7.24
6.88
2.88
1.97
0.92
0.96
0.98
2.17
1.09
6.07
10
9
8
7
6
5
4
3
2
1
0

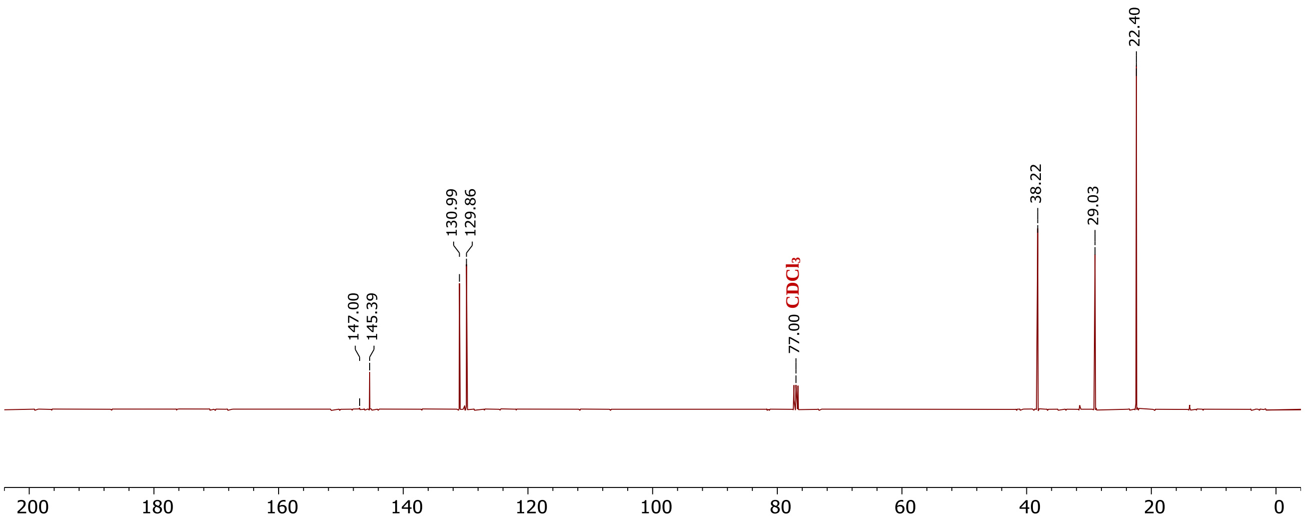

^{13}C NMR (100 MHz, CDCl$_3$)
147.00
145.39
130.99
129.86
77.00 CDCl₃
38.22
29.03
22.40
200
180
160
140
120
100
80
60
40
20
0

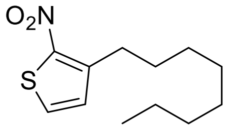

4t

^{1}H NMR (400 MHz, $CDCl_3$)

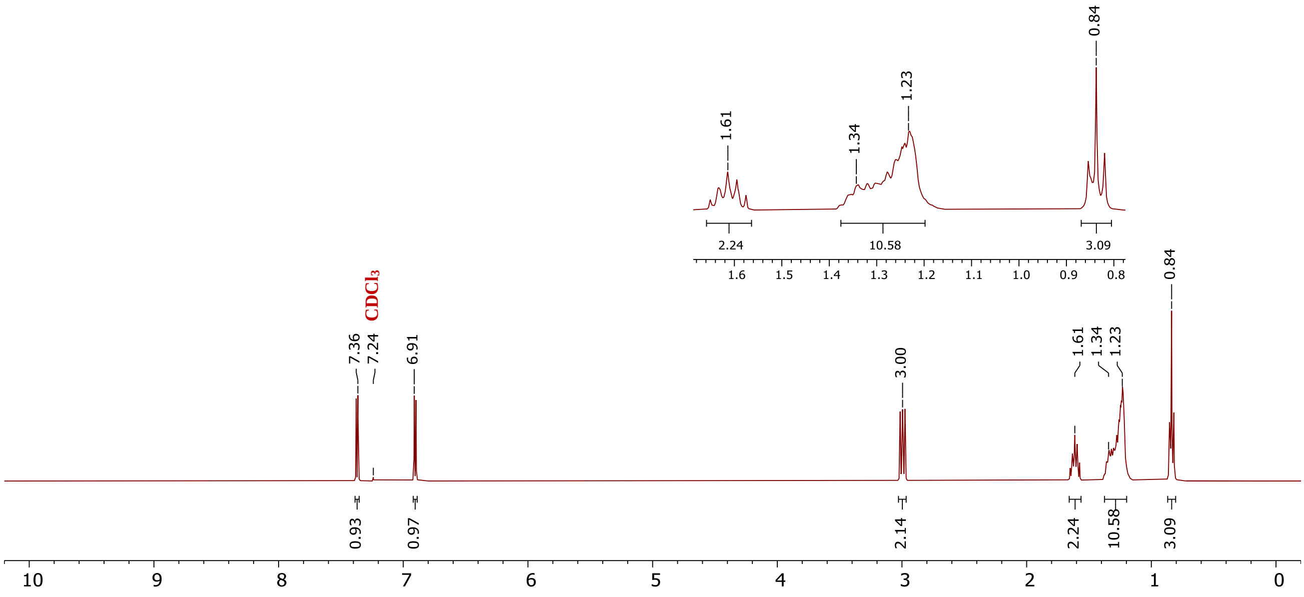

^{13}C NMR (100 MHz, $CDCl_3$)

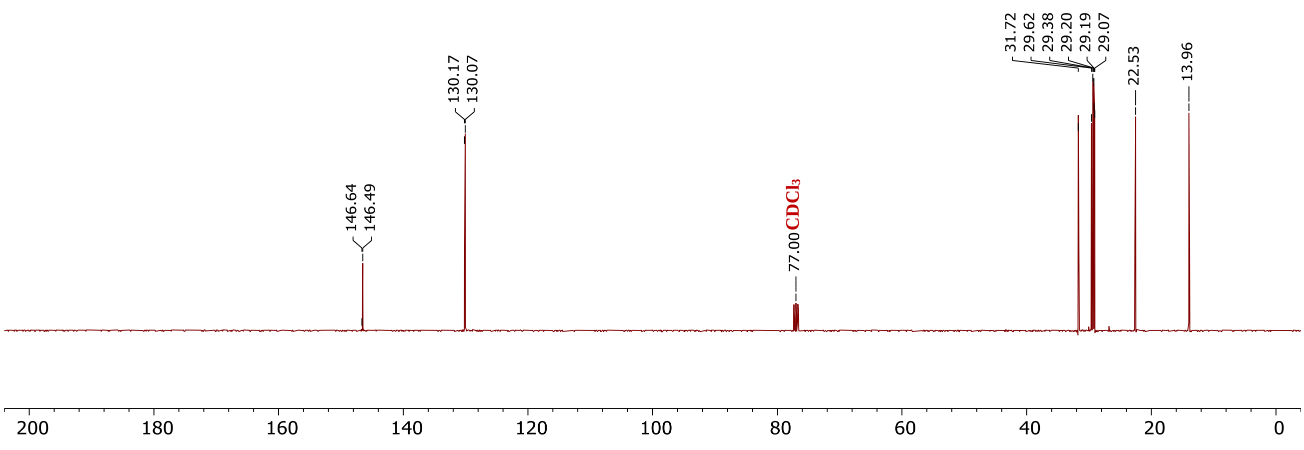

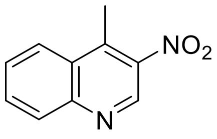

4u

^{1}H NMR (400 MHz, $CDCl_3$)

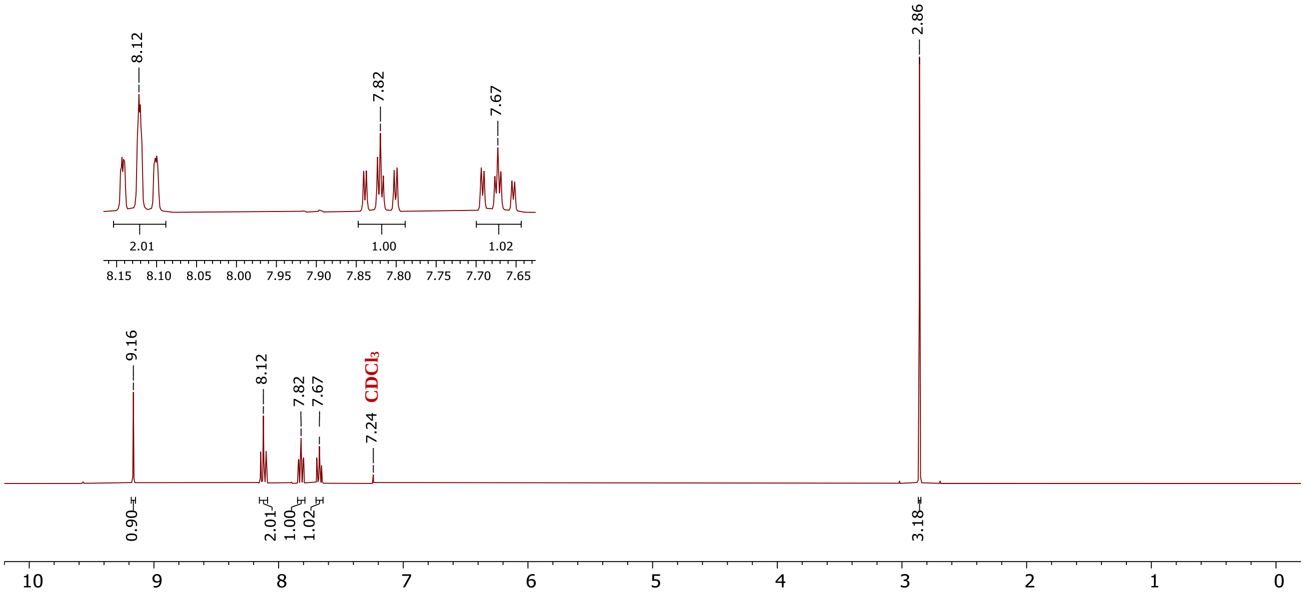

^{13}C NMR (100 MHz, $CDCl_3$)

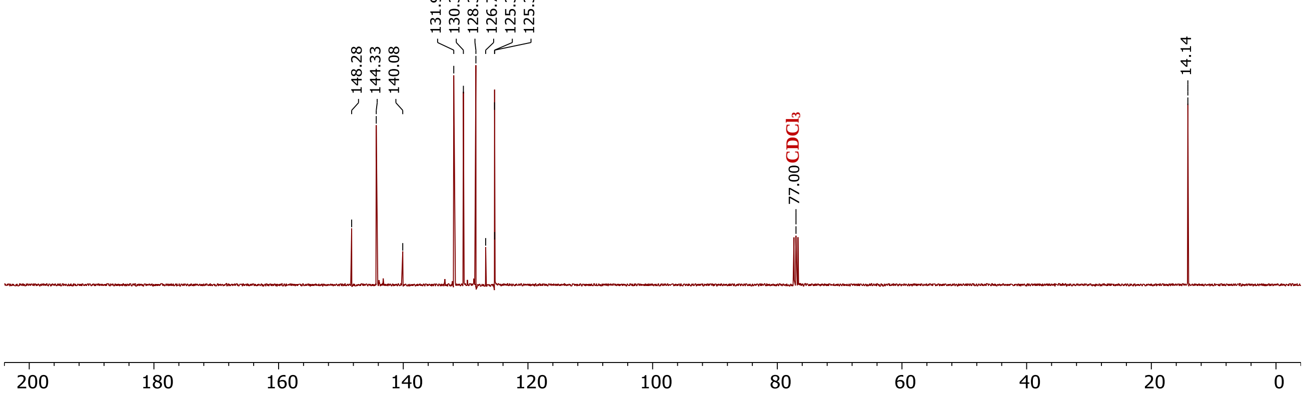

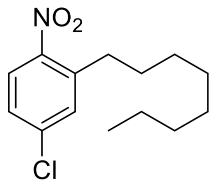

4v

^{1}H NMR (400 MHz, $CDCl_3$)

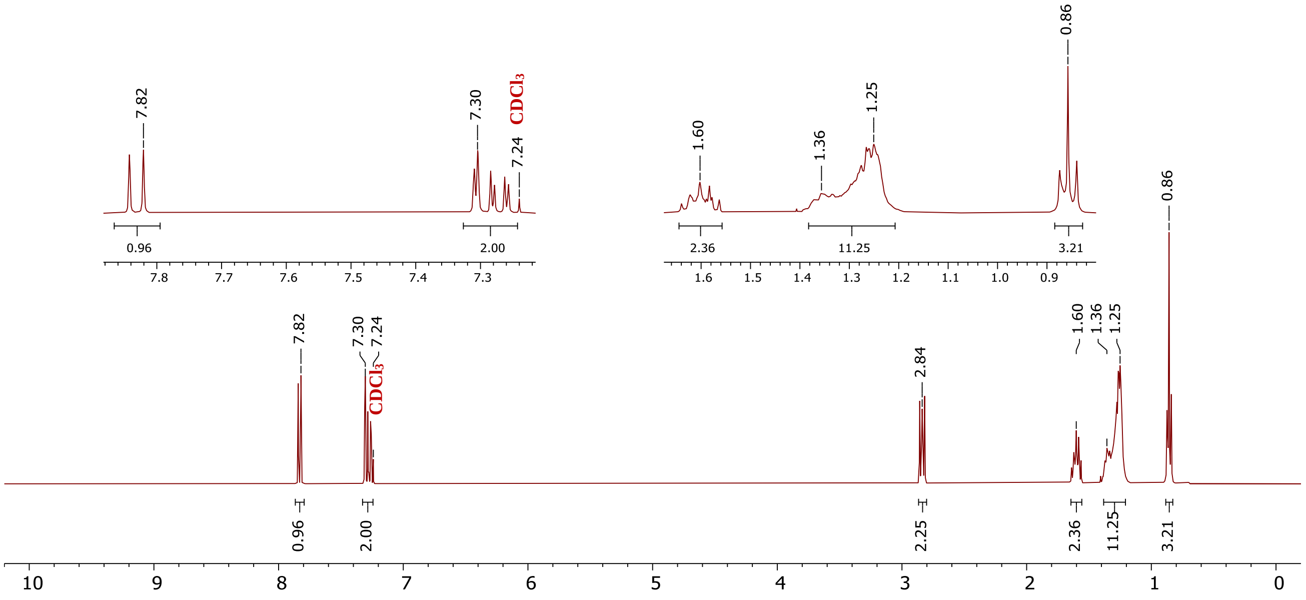

^{13}C NMR (100 MHz, $CDCl_3$)

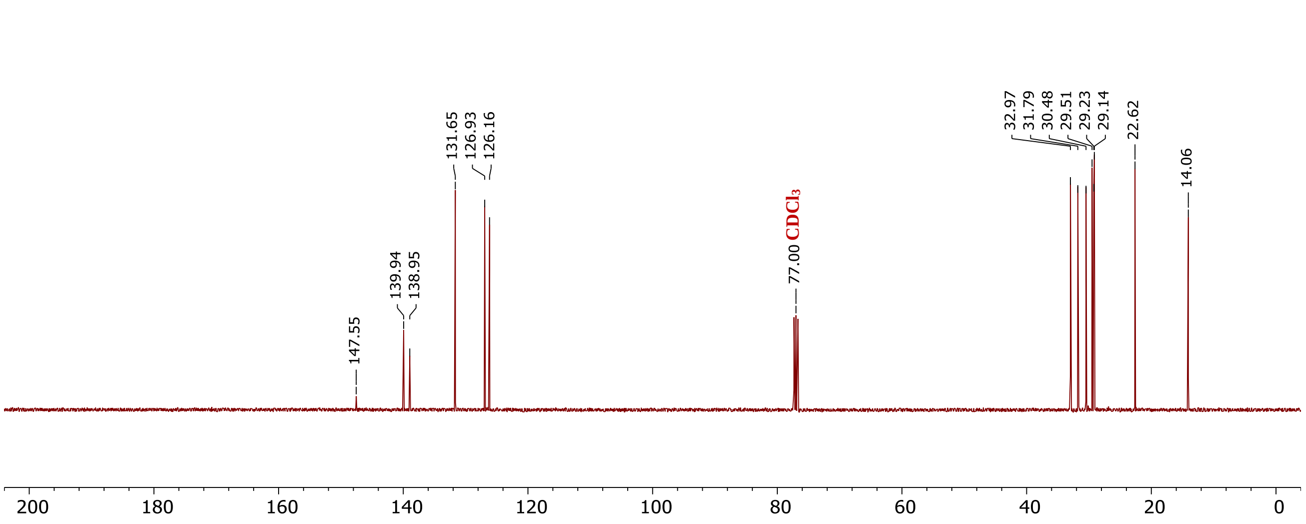

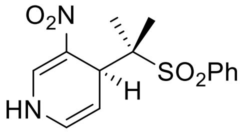

5a

^{1}H NMR (400 MHz, DMSO-d_6)

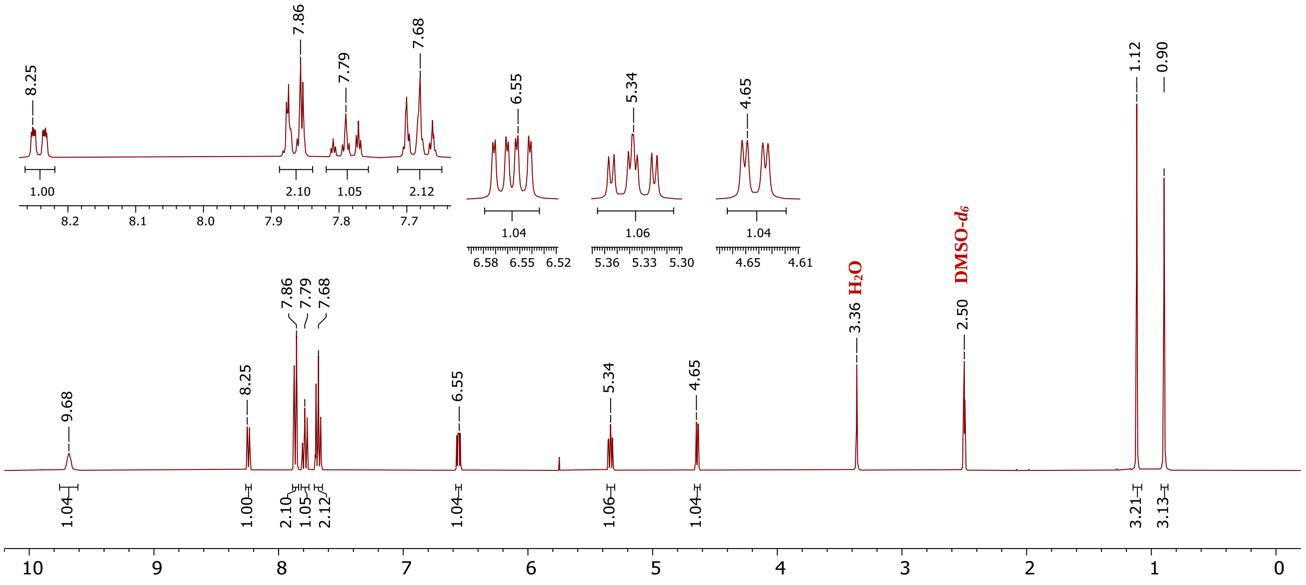

^{13}C NMR (100 MHz, DMSO-d_6)

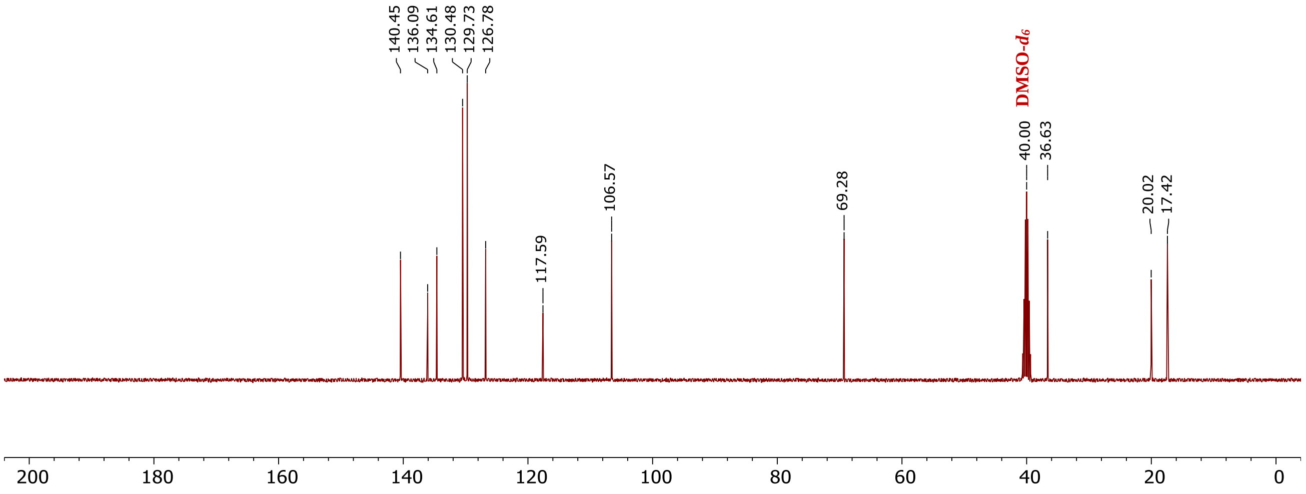

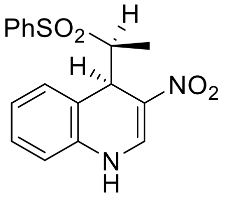

5b

^{1}H NMR (400 MHz, DMSO-d_6)

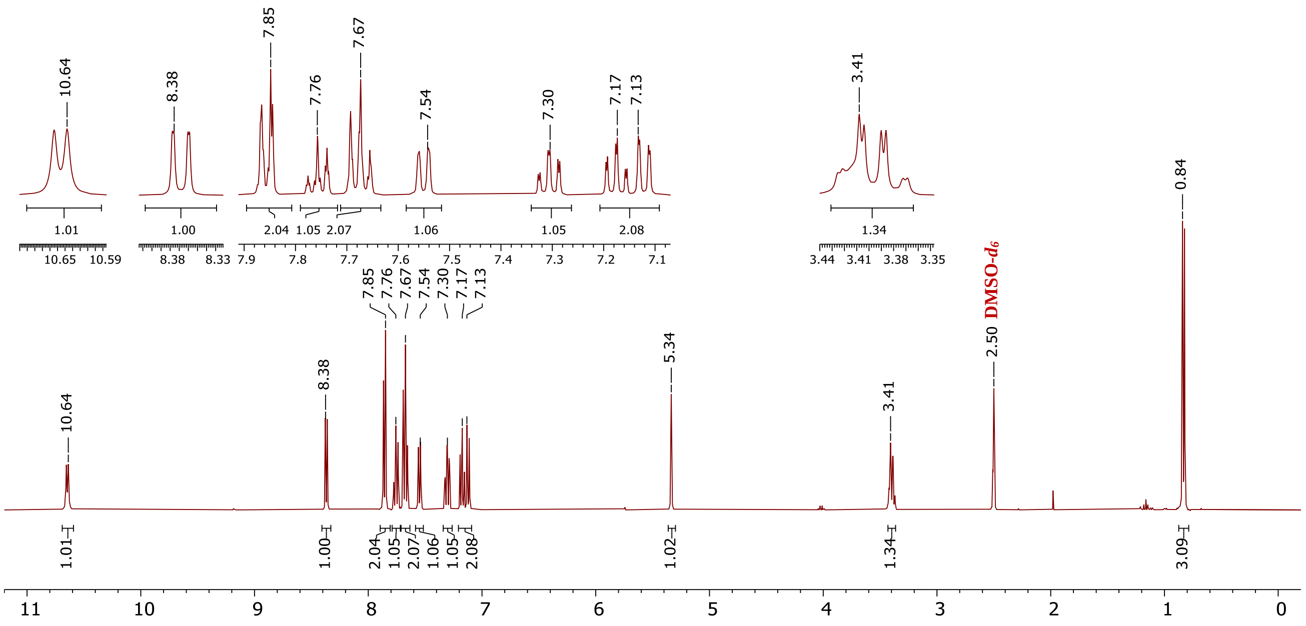

^{13}C NMR (100 MHz, DMSO-d_6)

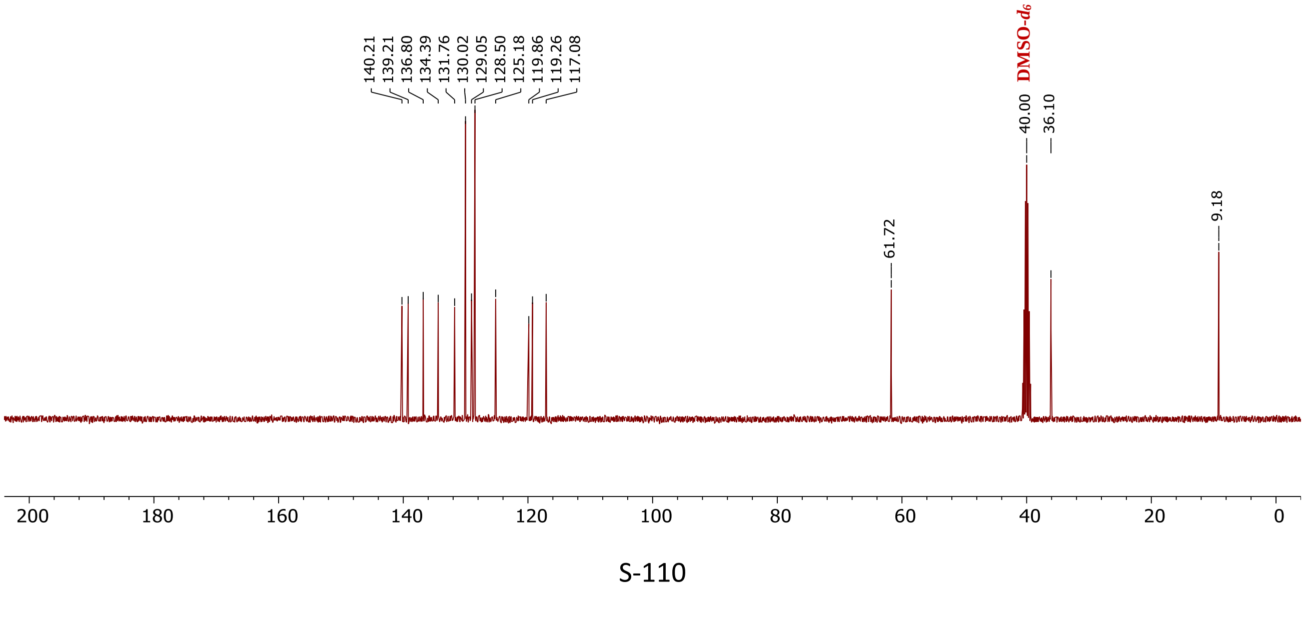

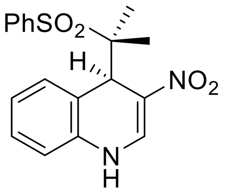

5c

^{1}H NMR (400 MHz, CDCl₃)

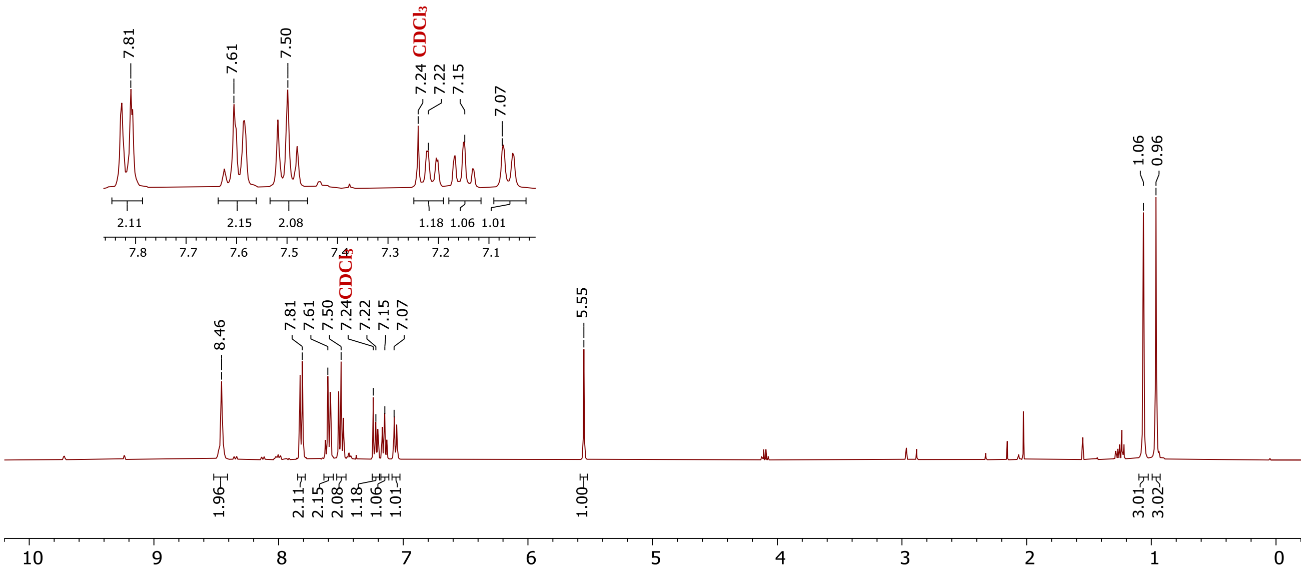

^{13}C NMR (100 MHz, CDCl₃)

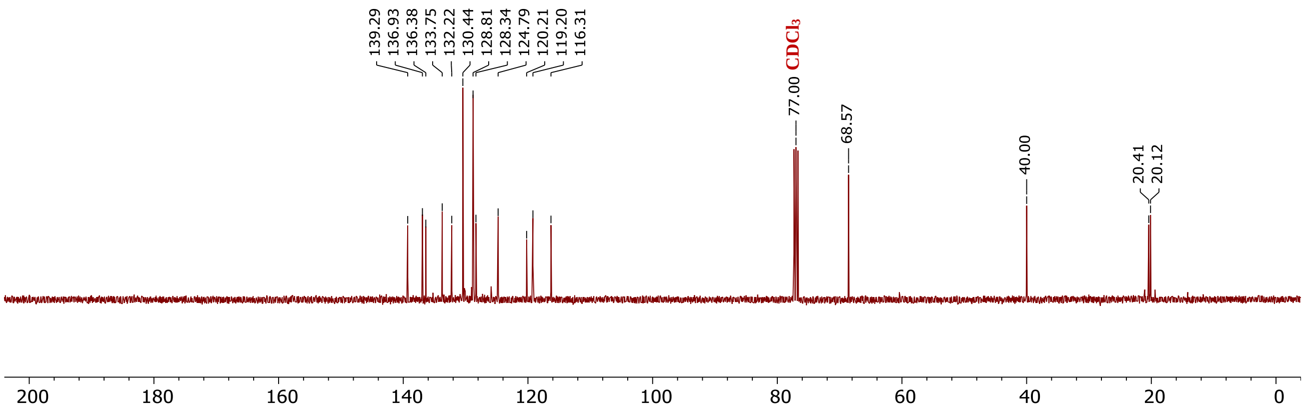

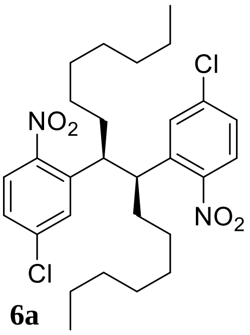

6a

^{1}H NMR (400 MHz, $CDCl_3$)

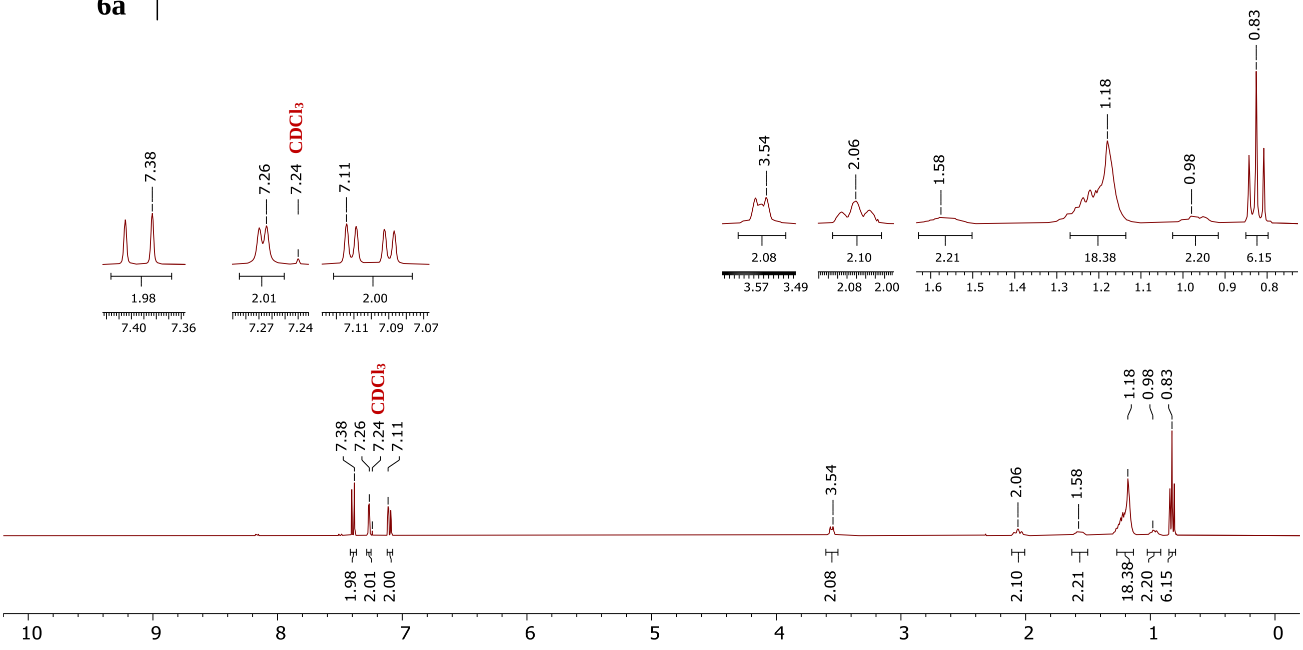

^{13}C NMR (100 MHz, $CDCl_3$)

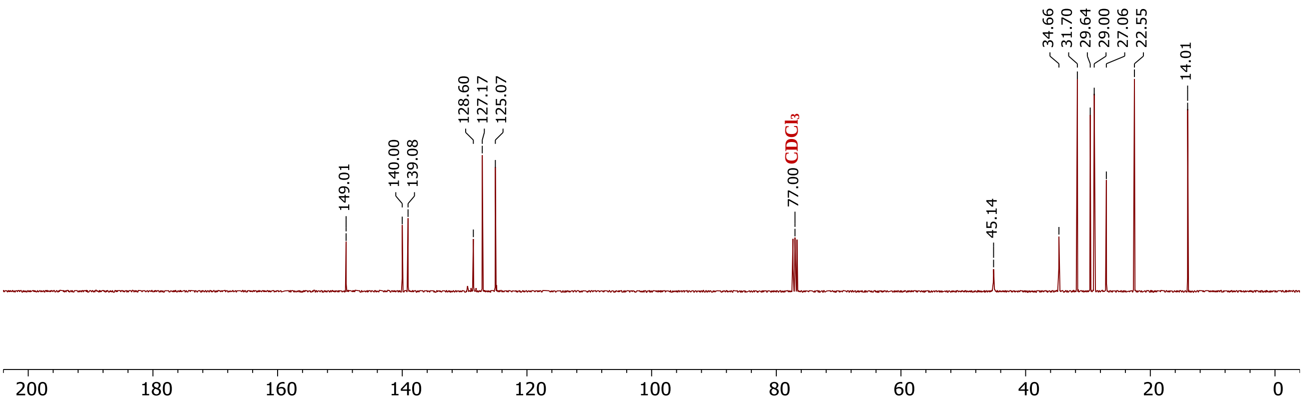

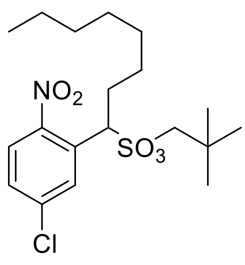

10

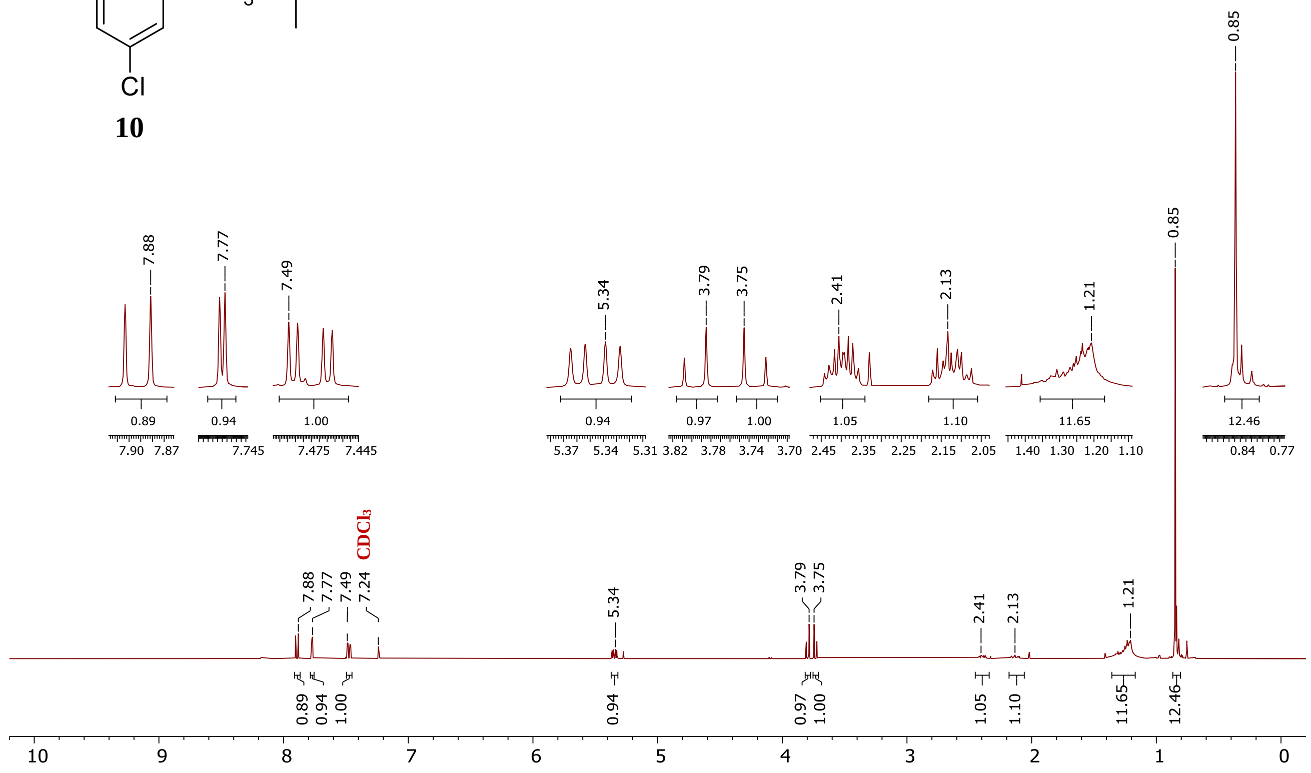

^{1}H NMR (400 MHz, CDCl$_3$)
0.85
7.88
7.77
7.49
5.34
3.79
3.75
2.41
2.13
1.21
0.89
0.94
1.00
0.94
0.97
1.00
1.05
1.10
11.65
12.46
CDCl$_3$
7.24

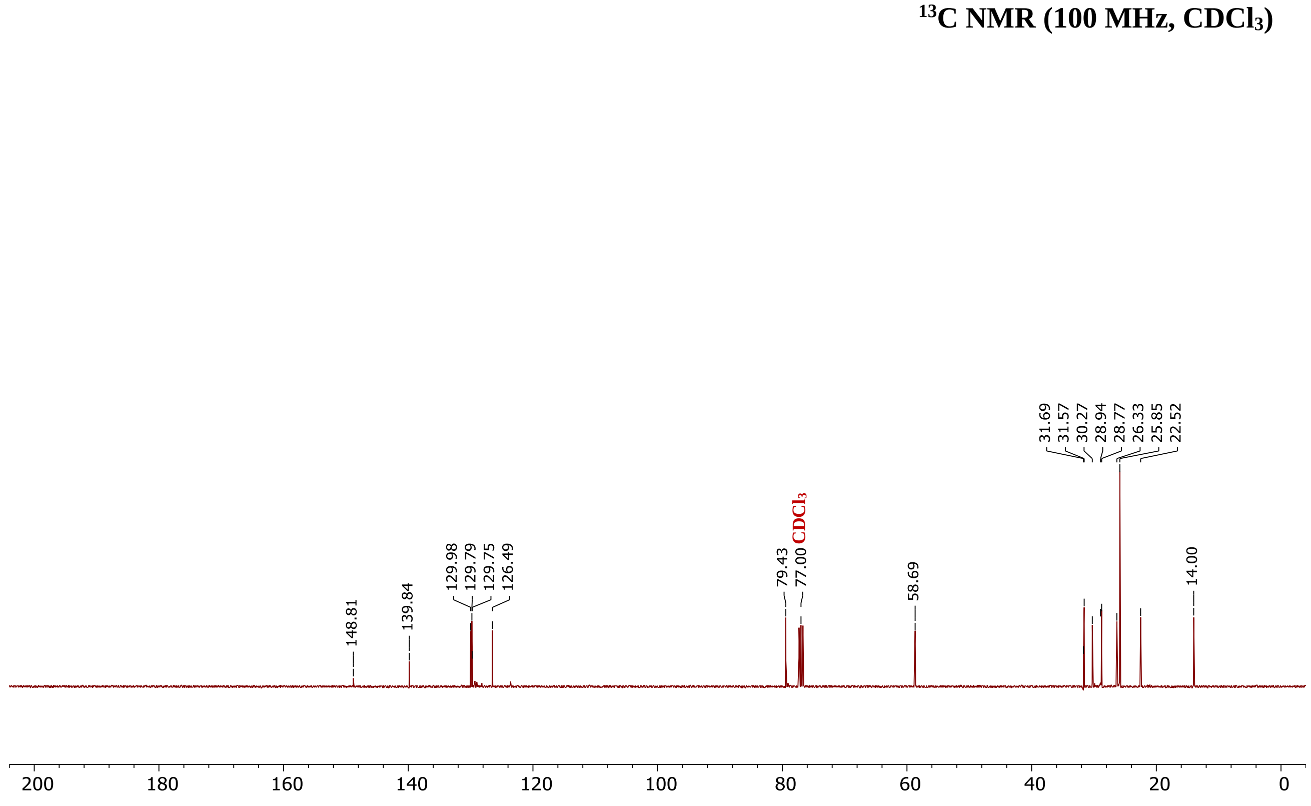

^{13}C NMR (100 MHz, CDCl$_3$)
148.81
139.84
129.98
129.79
129.75
126.49
79.43
77.00
CDCl$_3$
58.69
31.69
31.57
30.27
28.94
28.77
26.33
25.85
22.52
14.00

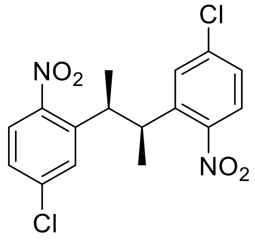

6b

^{1}H NMR (400 MHz, CDCl$_3$)

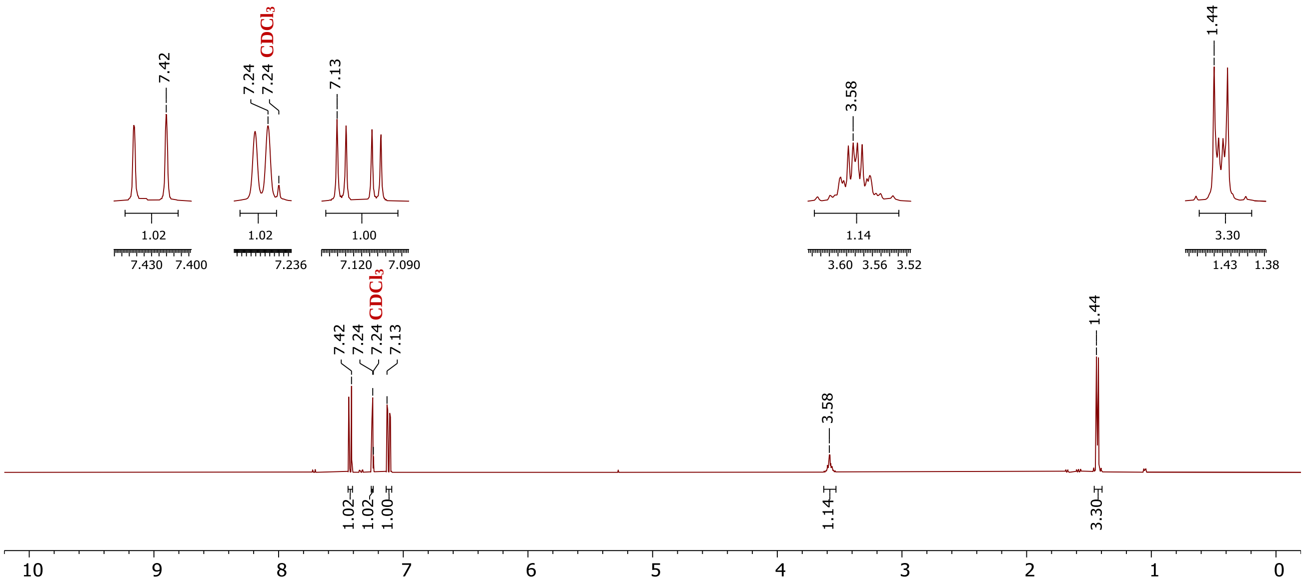

^{13}C NMR (100 MHz, CDCl$_3$)

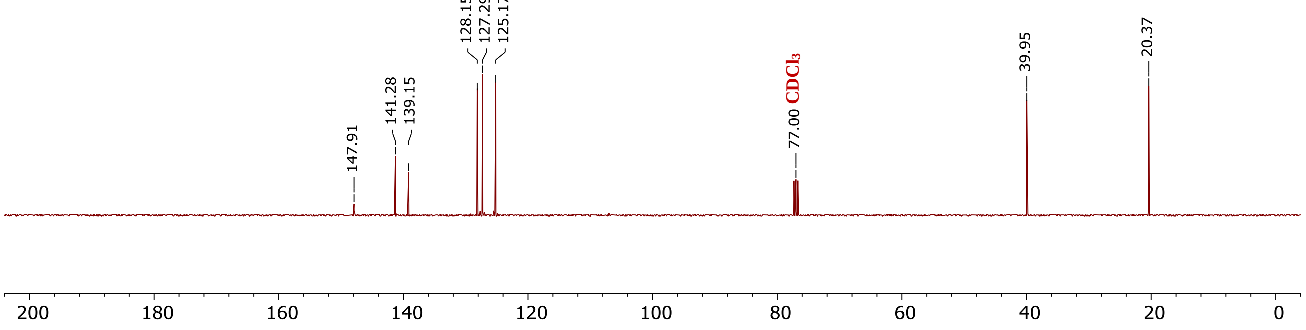

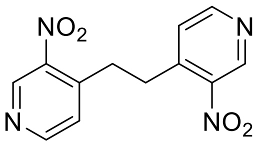

6c

^{1}H NMR (400 MHz, $CDCl_3$)

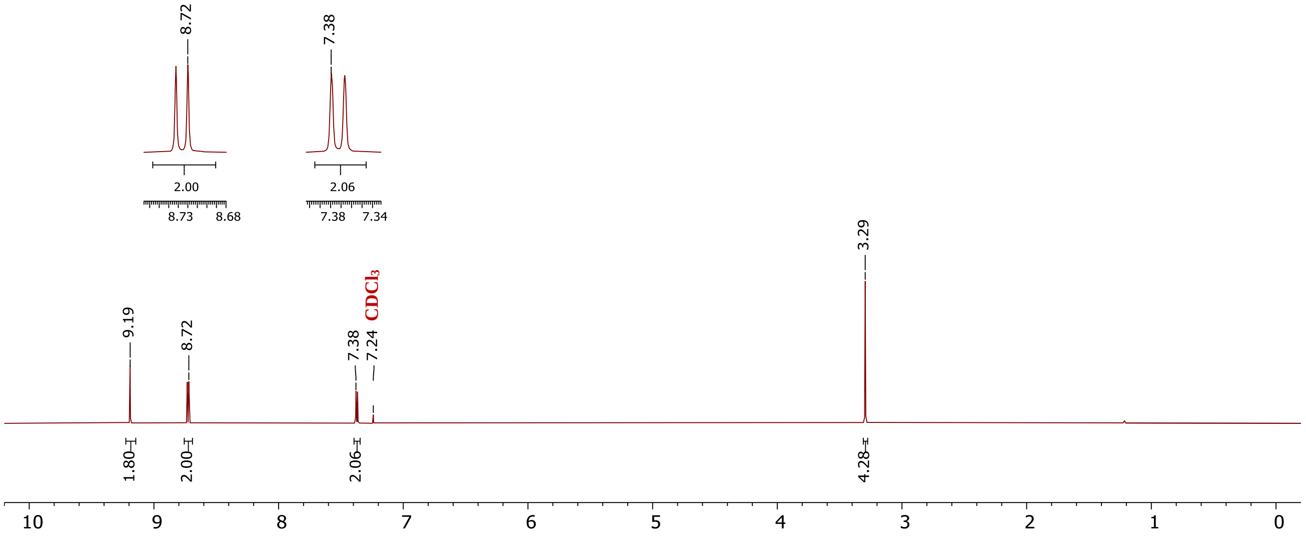

^{13}C NMR (100 MHz, $CDCl_3$)

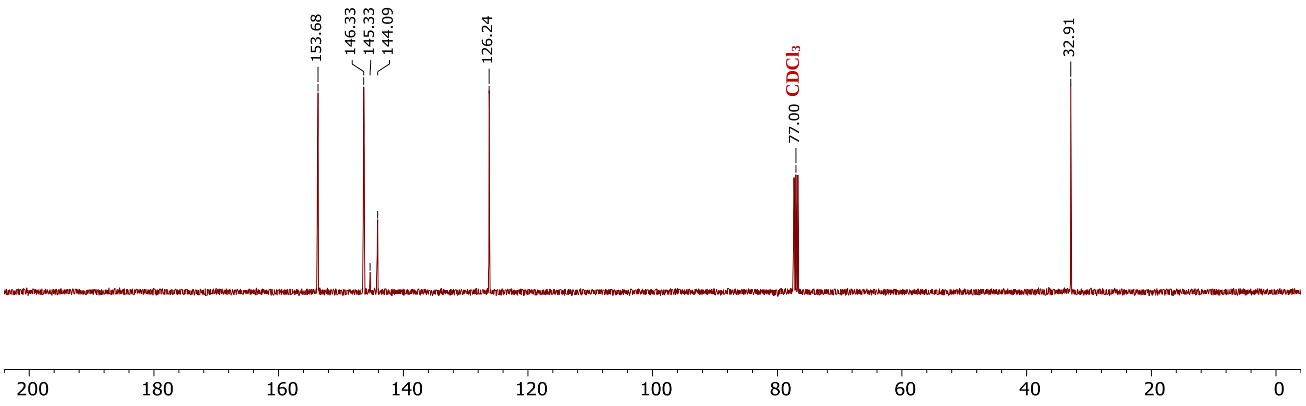

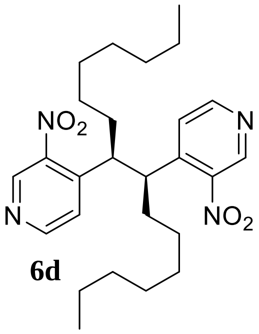

6d

^{1}H NMR (400 MHz, $CDCl_3$)

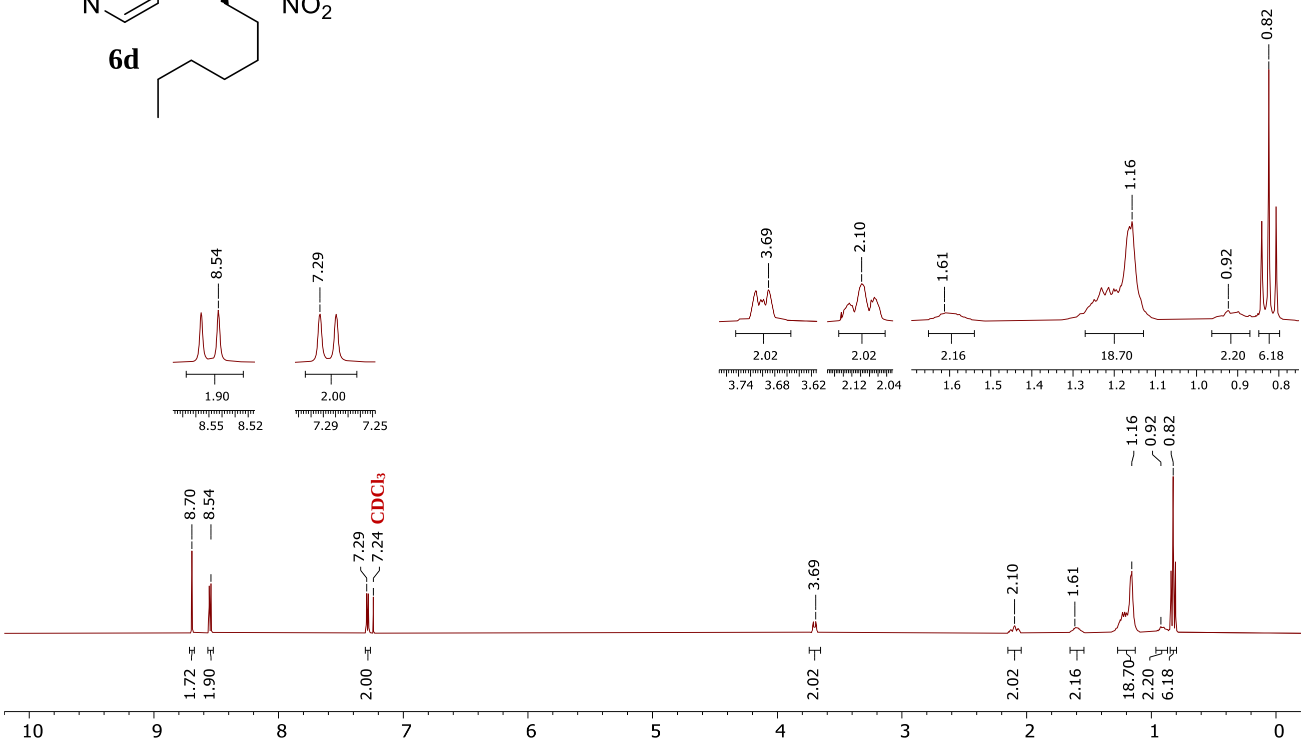

^{13}C NMR (100 MHz, $CDCl_3$)

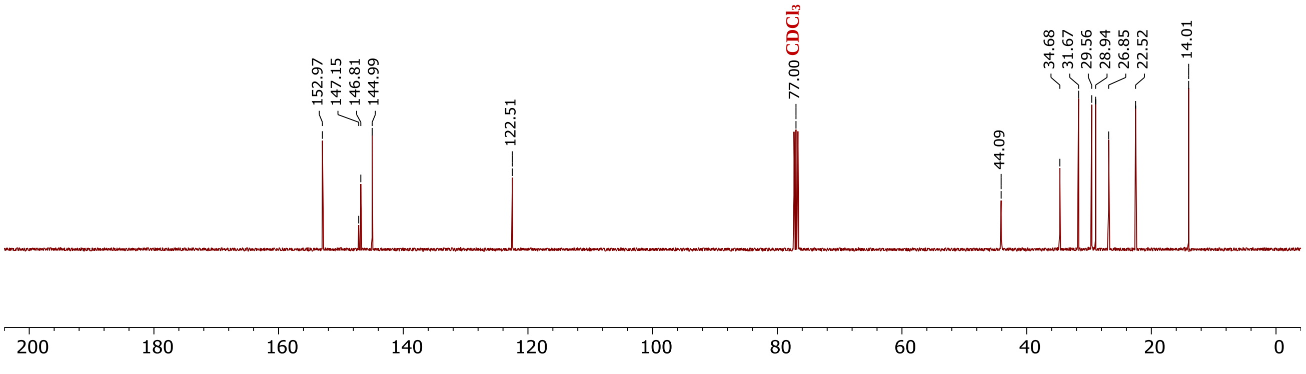

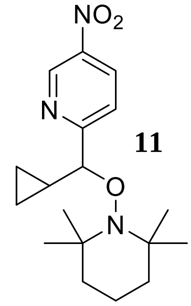

11

^{1}H NMR (400 MHz, CDCl$_3$)

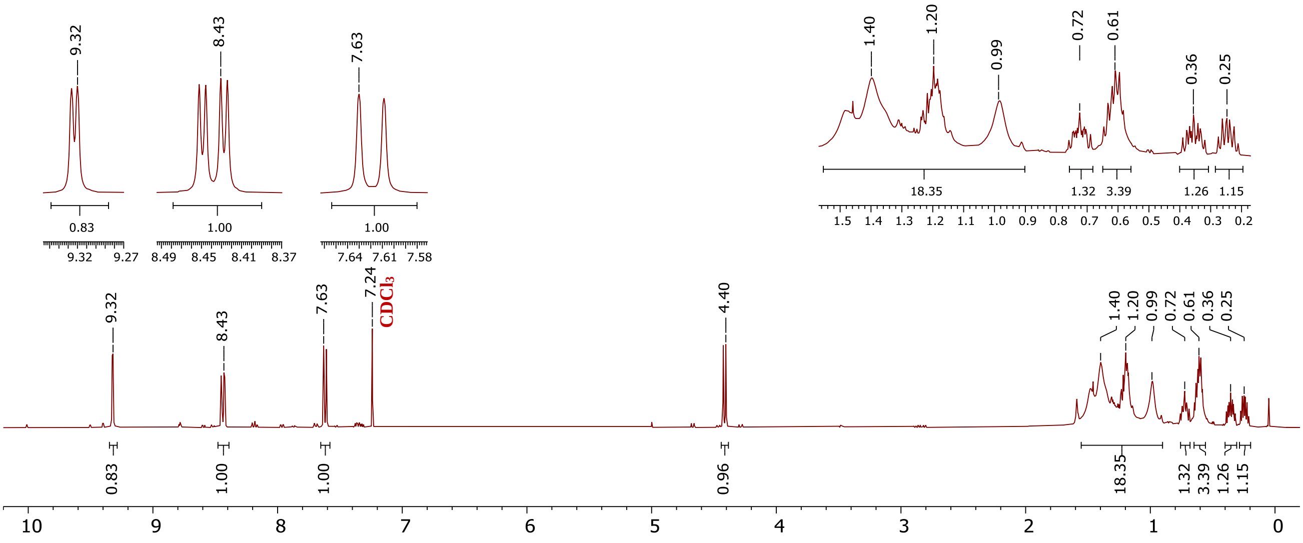

^{13}C NMR (100 MHz, CDCl$_3$)

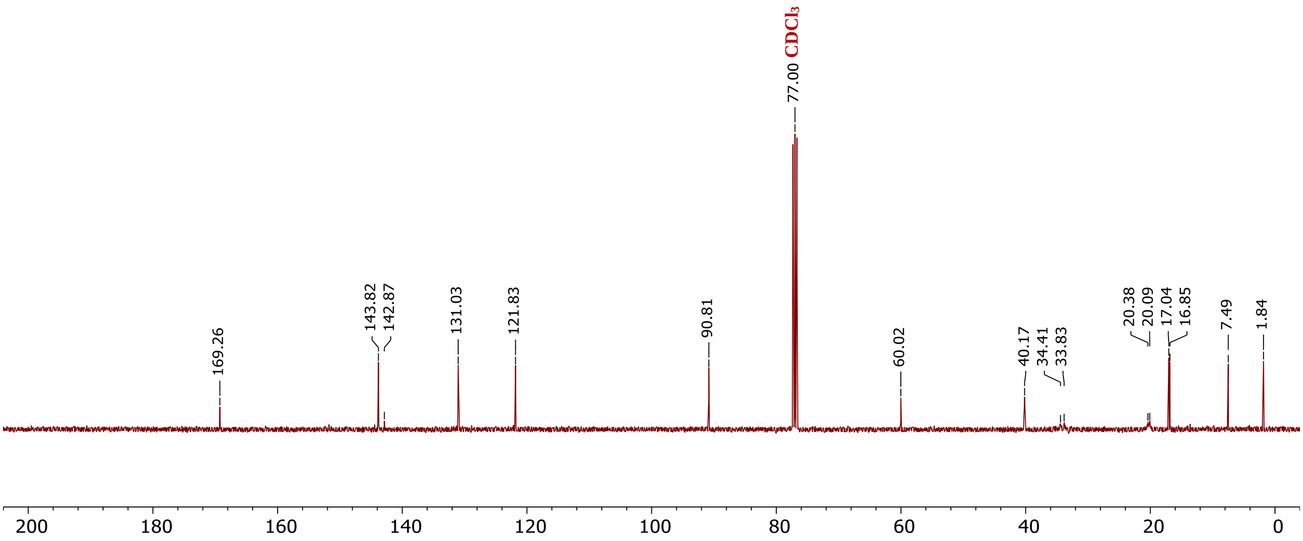

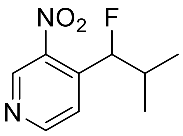

7a

^{1}H NMR (400 MHz, CDCl$_3$)

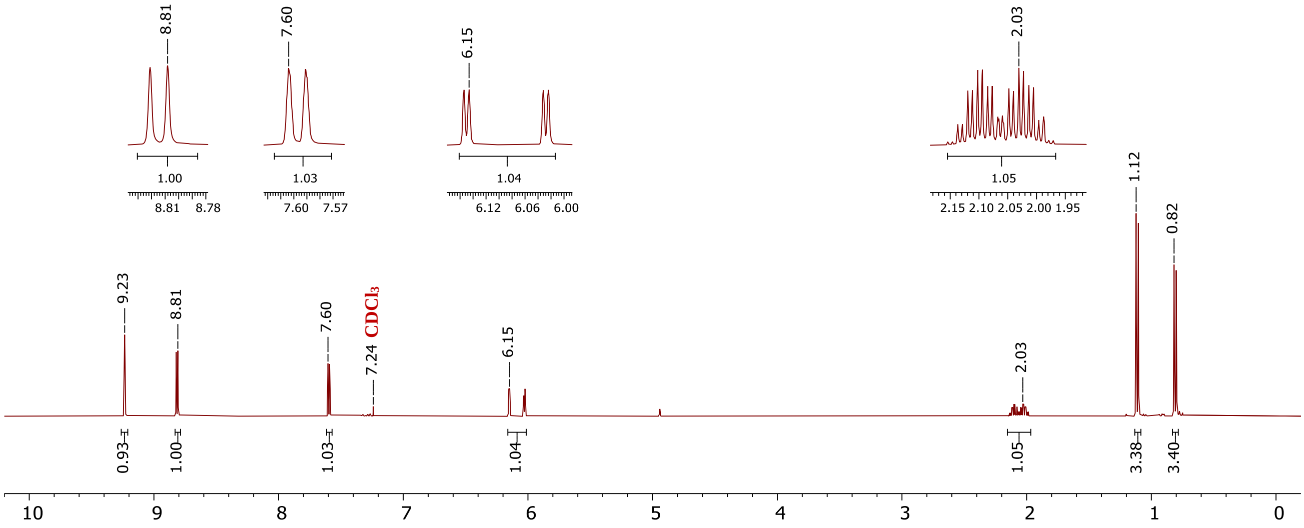

^{13}C NMR (100 MHz, CDCl$_3$)

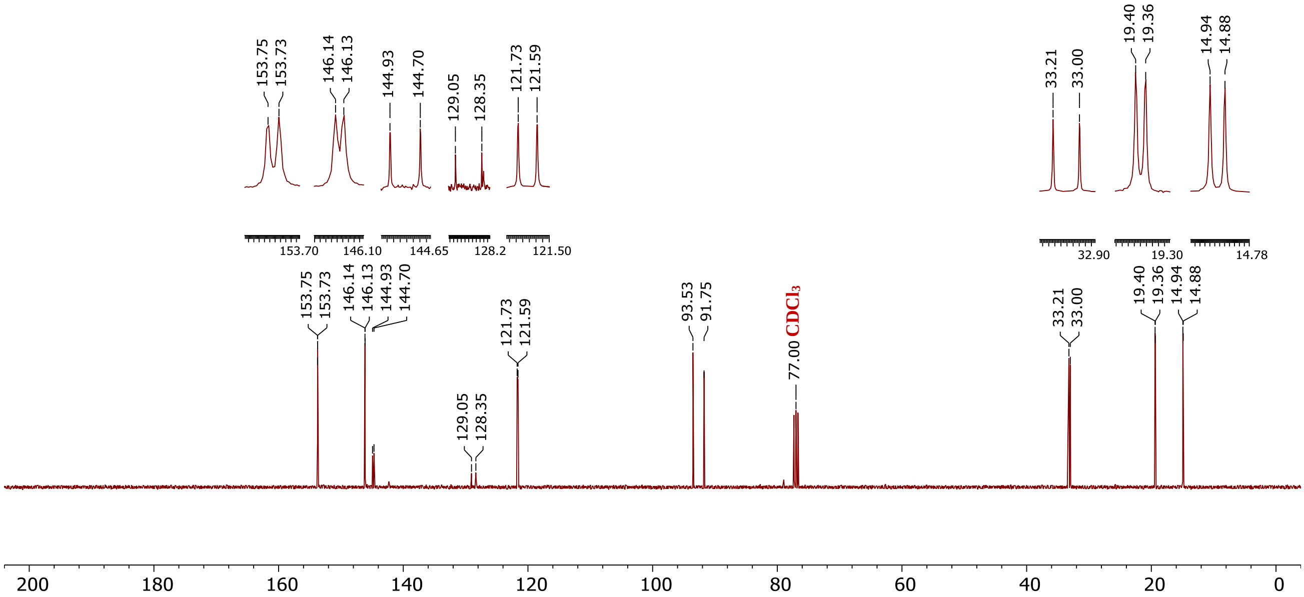

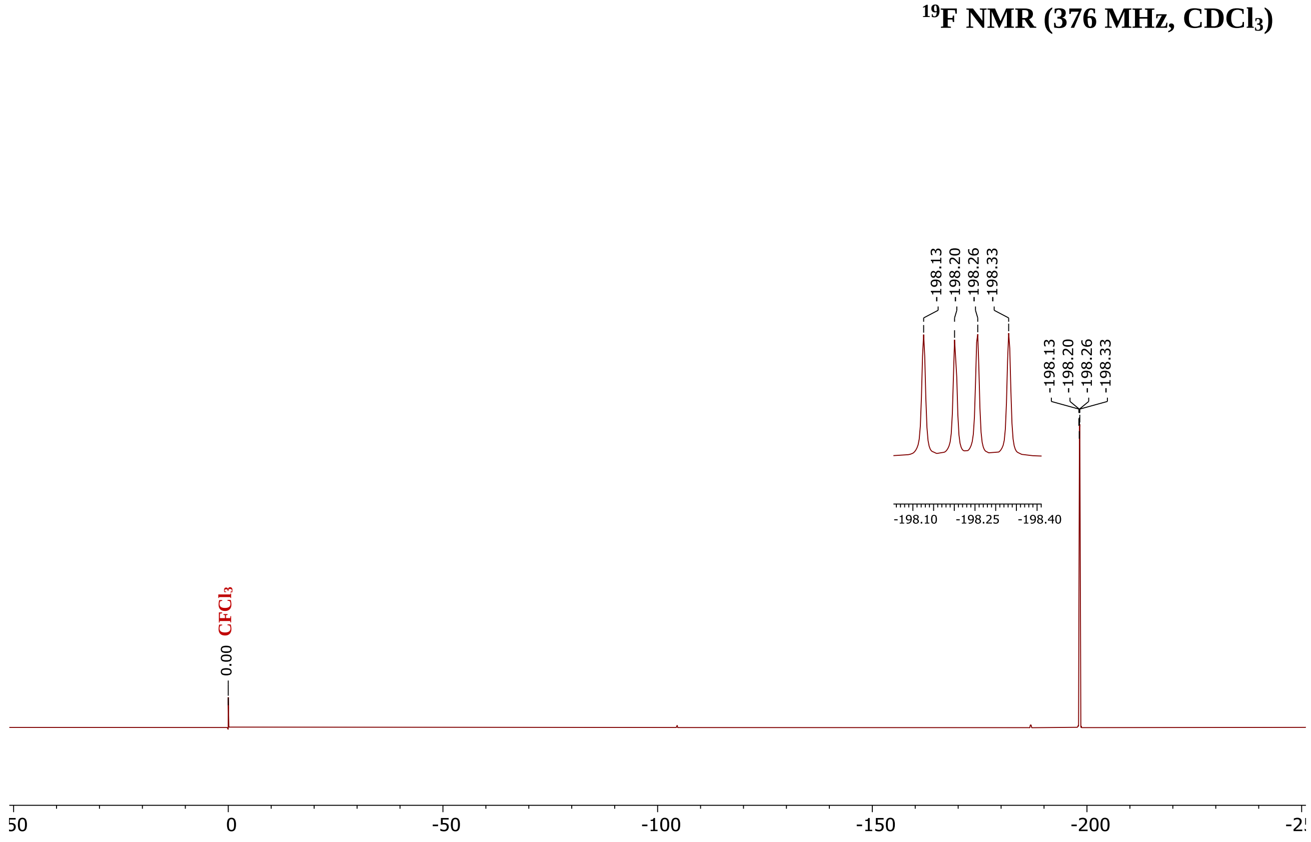
^{19}F NMR (376 MHz, CDCl$_3$)
-198.13
-198.20
-198.26
-198.33
-198.10
-198.25
-198.40
0.00
CFCl$_3$
50
0
-50
-100
-150
-200

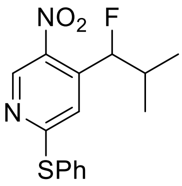

7b

^{1}H NMR (400 MHz, CDCl$_3$)

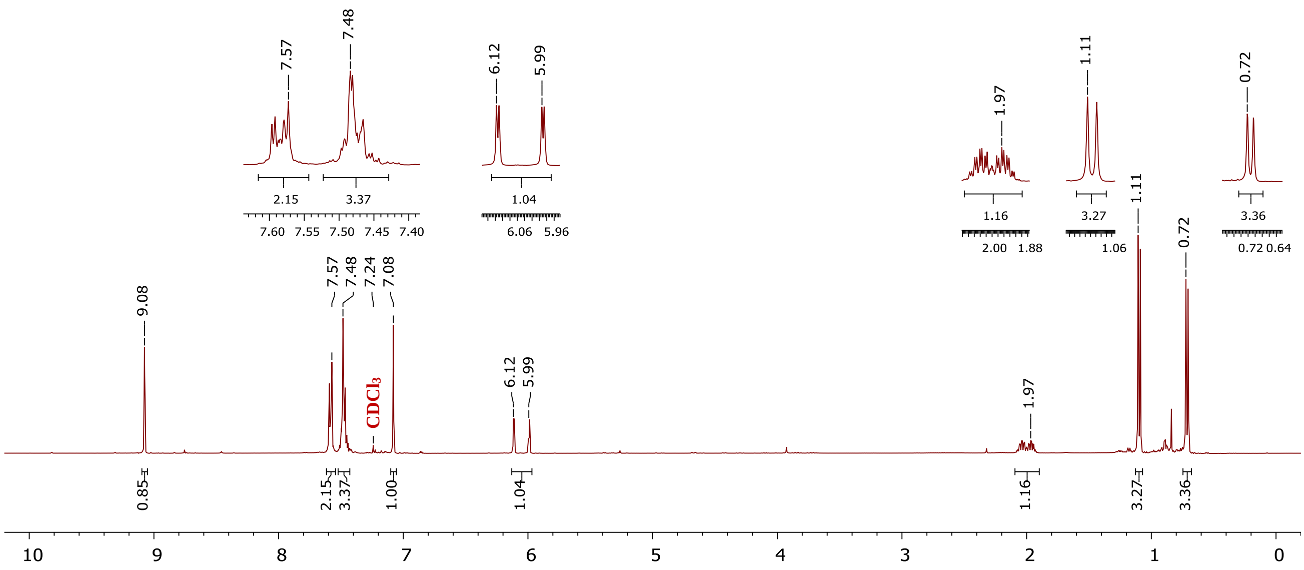

^{13}C NMR (100 MHz, CDCl$_3$)

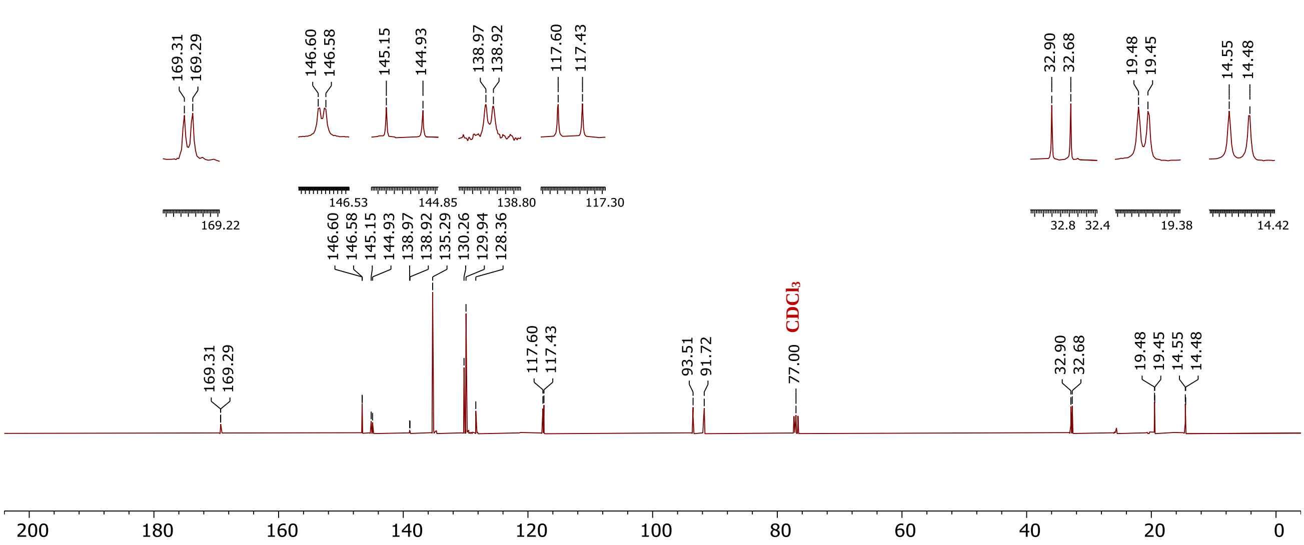

^{19}F NMR (376 MHz, $CDCl_3$)

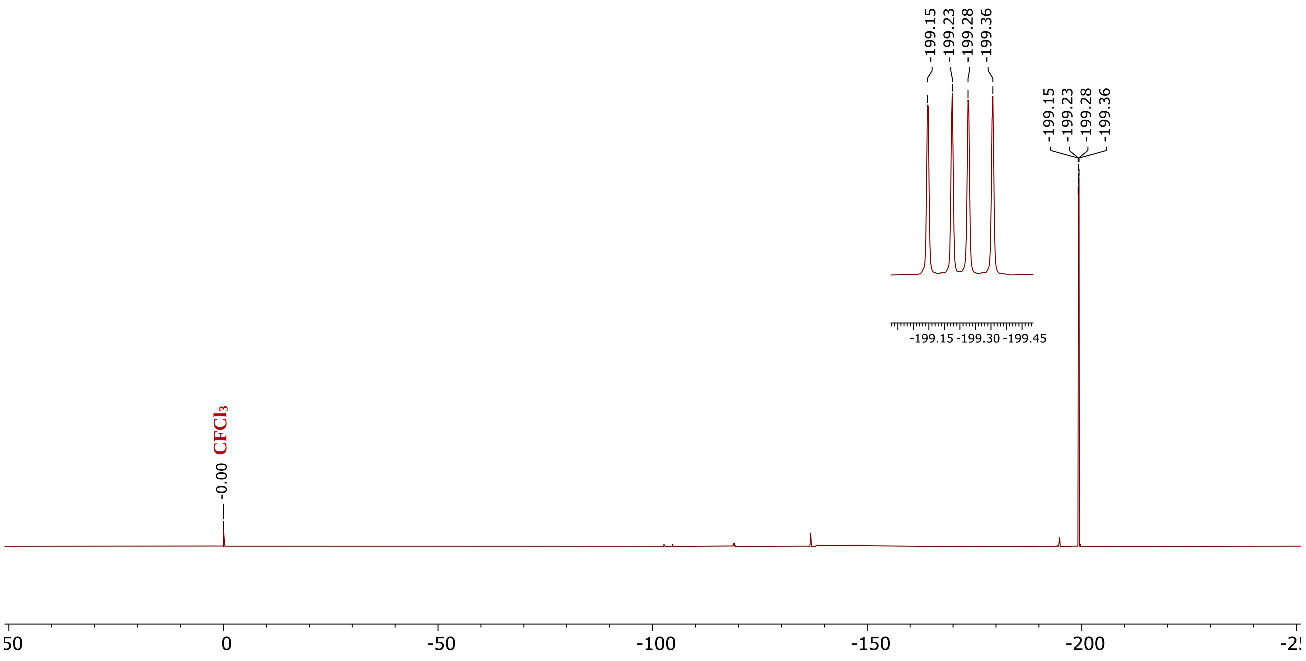

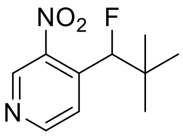

7c

^{1}H NMR (400 MHz, $CDCl_3$)

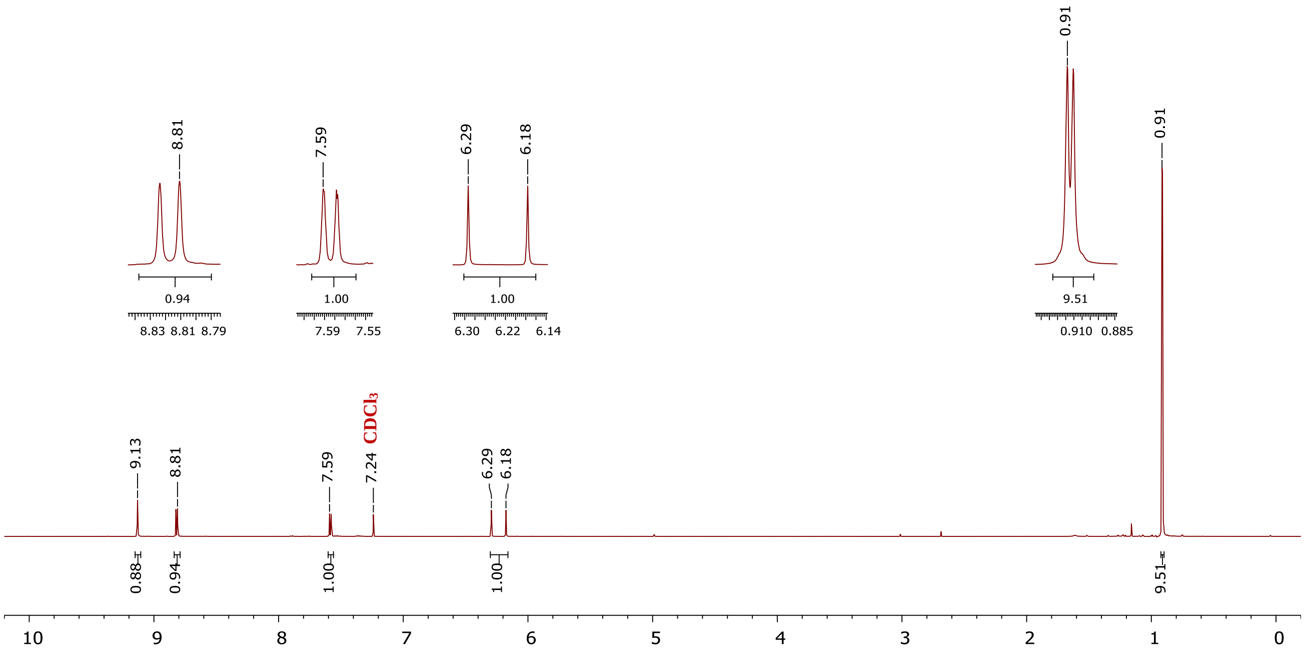

^{13}C NMR (100 MHz, $CDCl_3$)

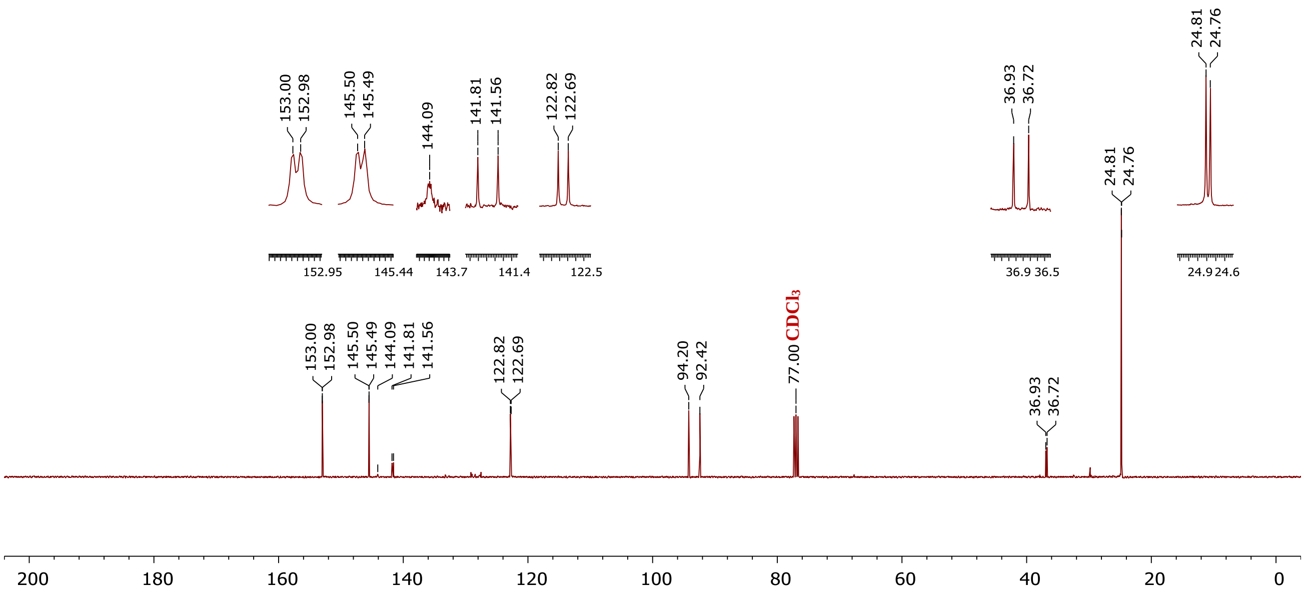

^{19}F NMR (376 MHz, $CDCl_3$)

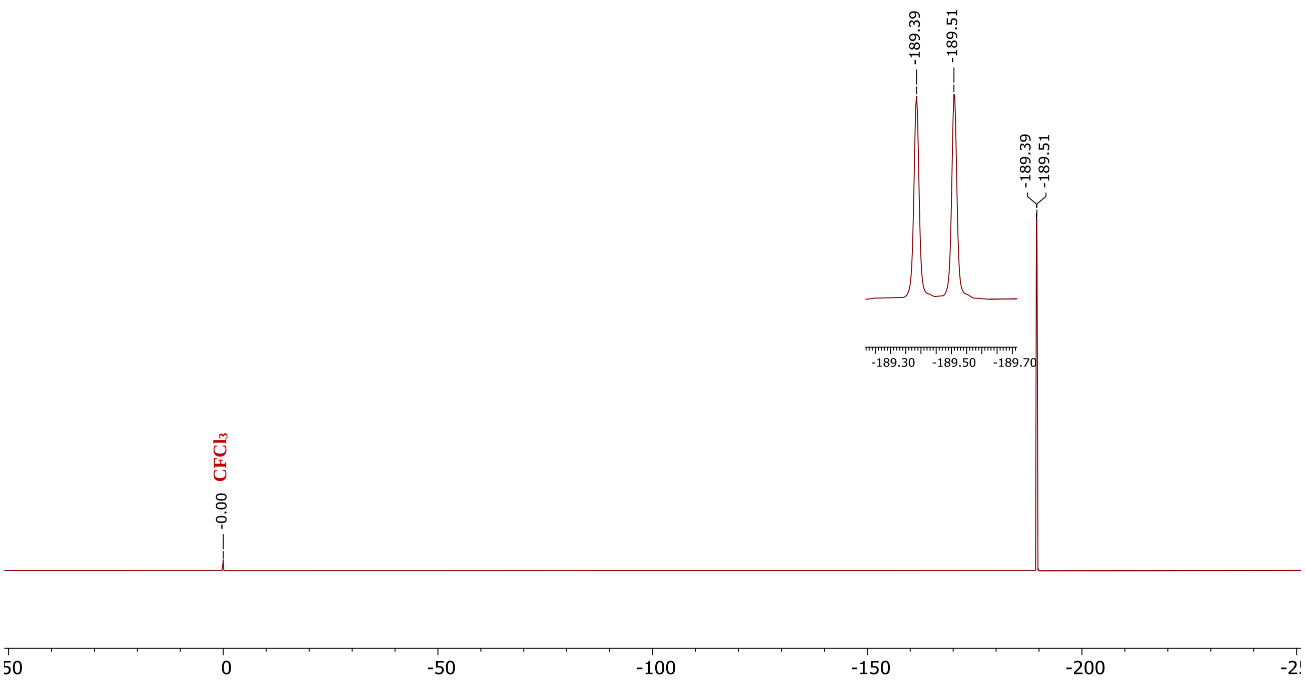

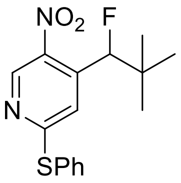

7d

^{1}H NMR (400 MHz, CDCl$_3$)

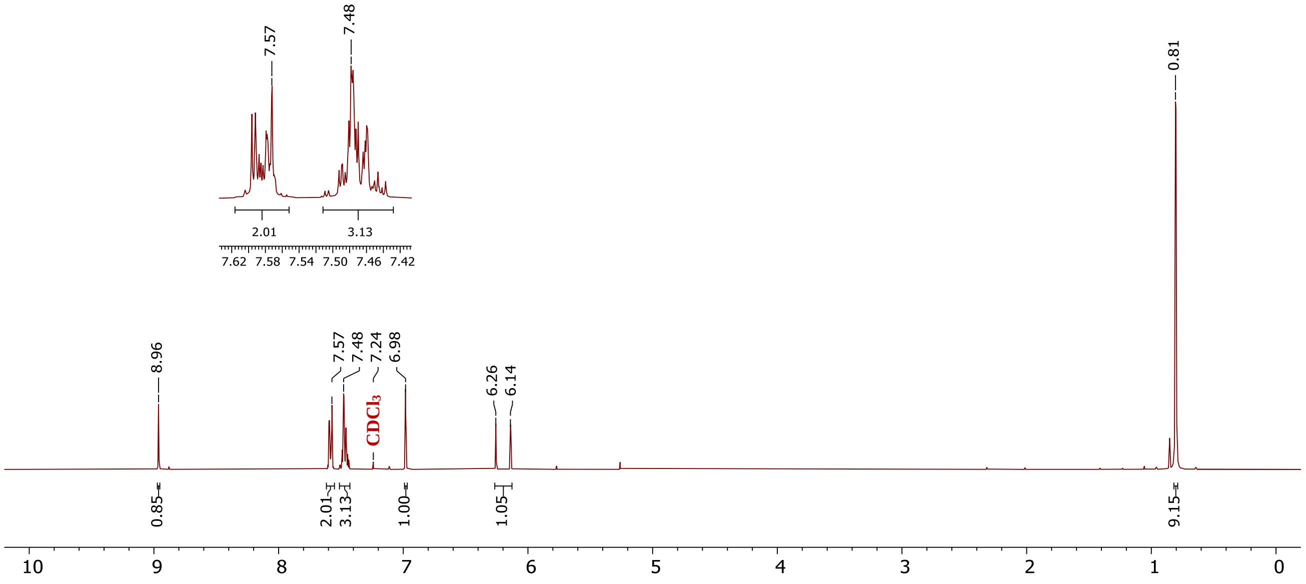

^{13}C NMR (100 MHz, CDCl$_3$)

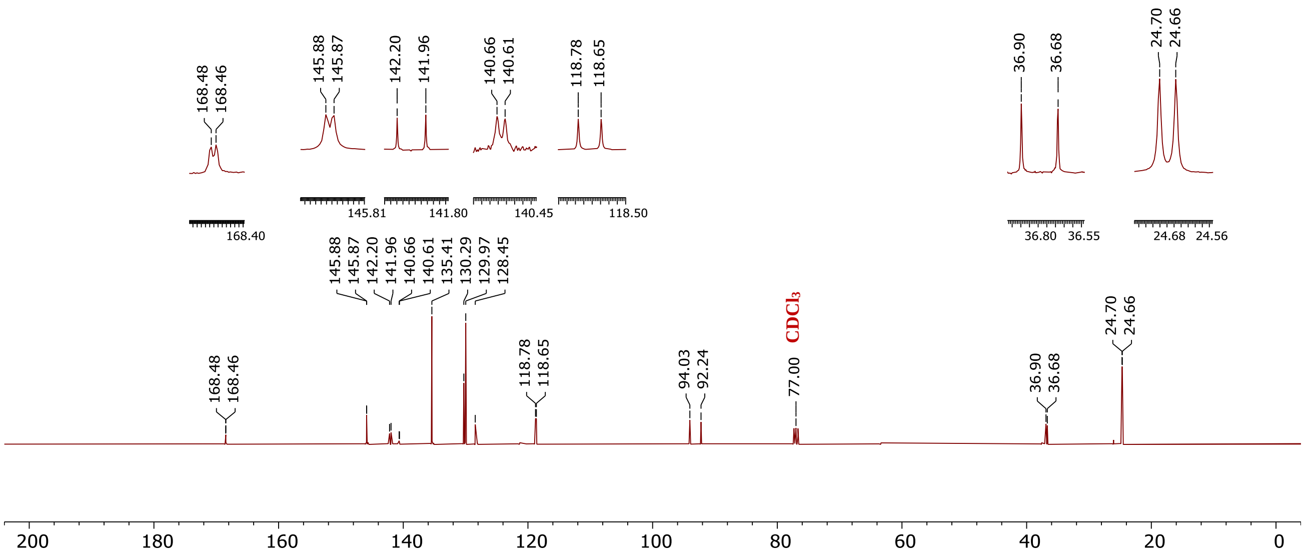

^{19}F NMR (376 MHz, $CDCl_3$)

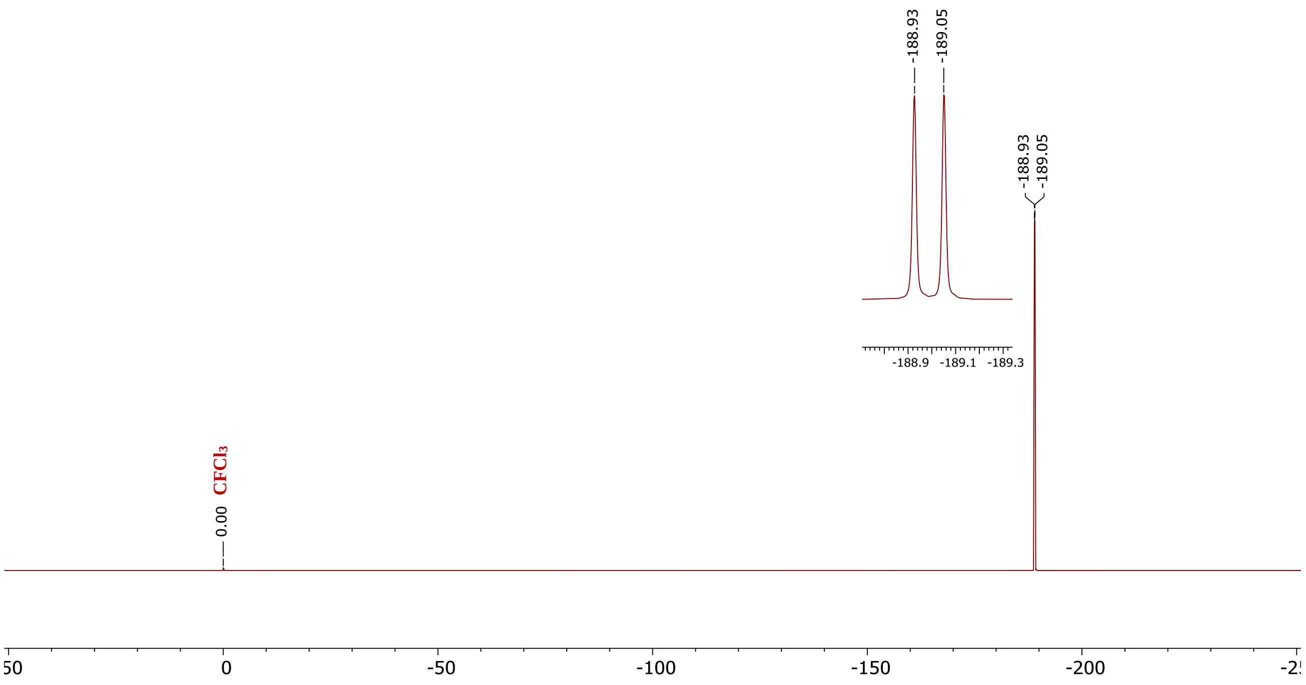

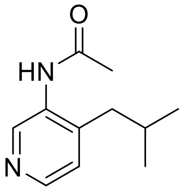

8

^{1}H NMR (400 MHz, $CDCl_3$)

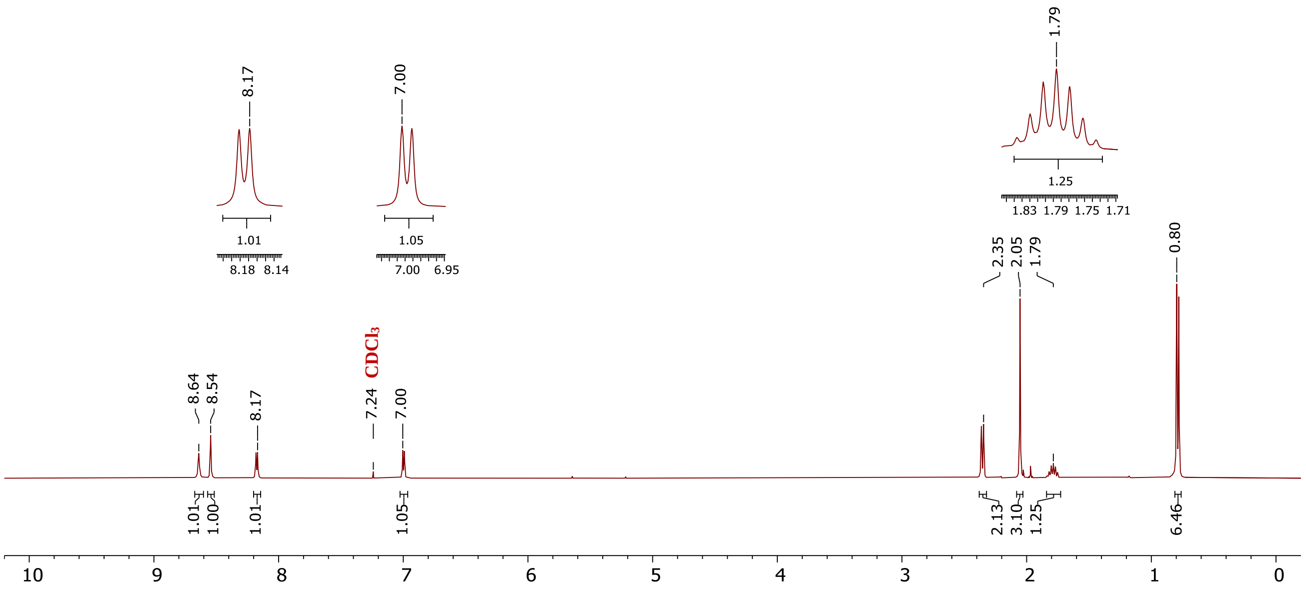

^{13}C NMR (100 MHz, $CDCl_3$)

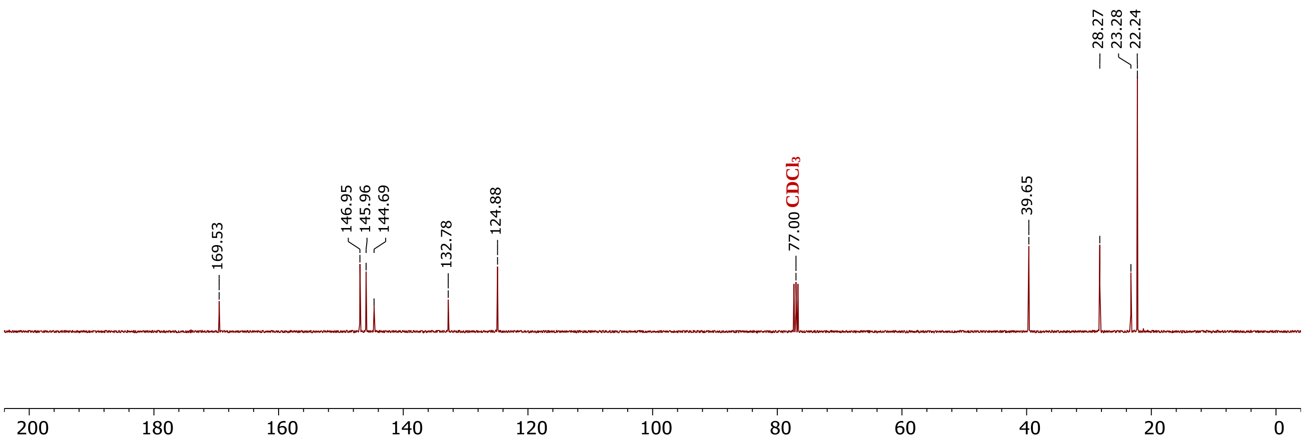

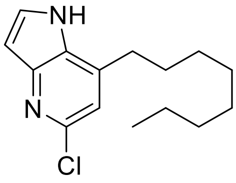

9

^{1}H NMR (400 MHz, $CDCl_3$)

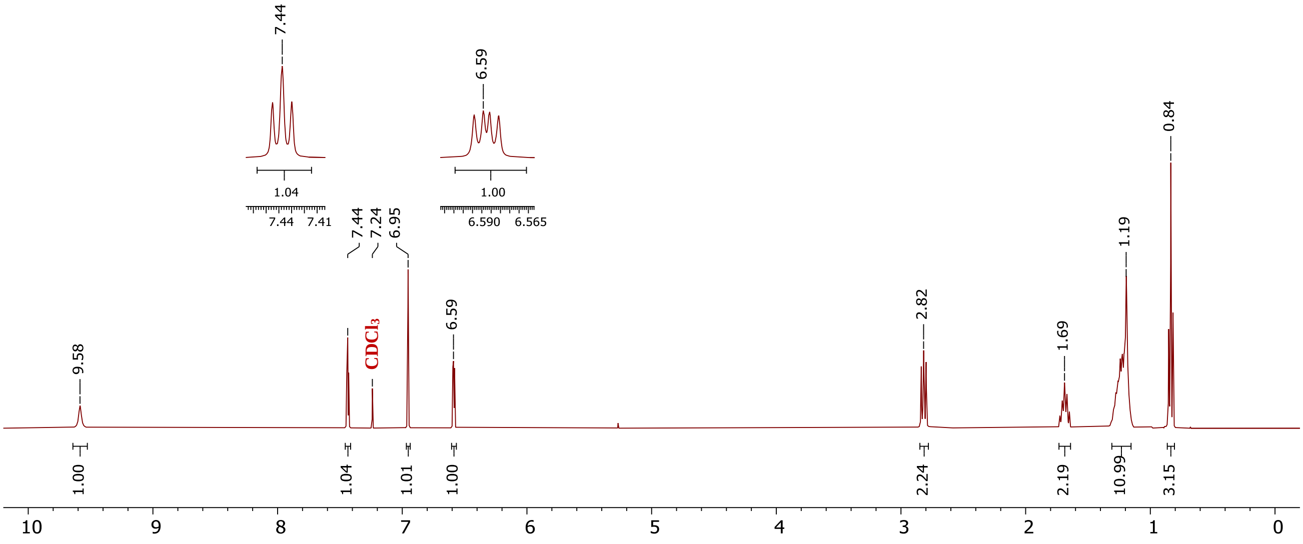

^{13}C NMR (100 MHz, $CDCl_3$)

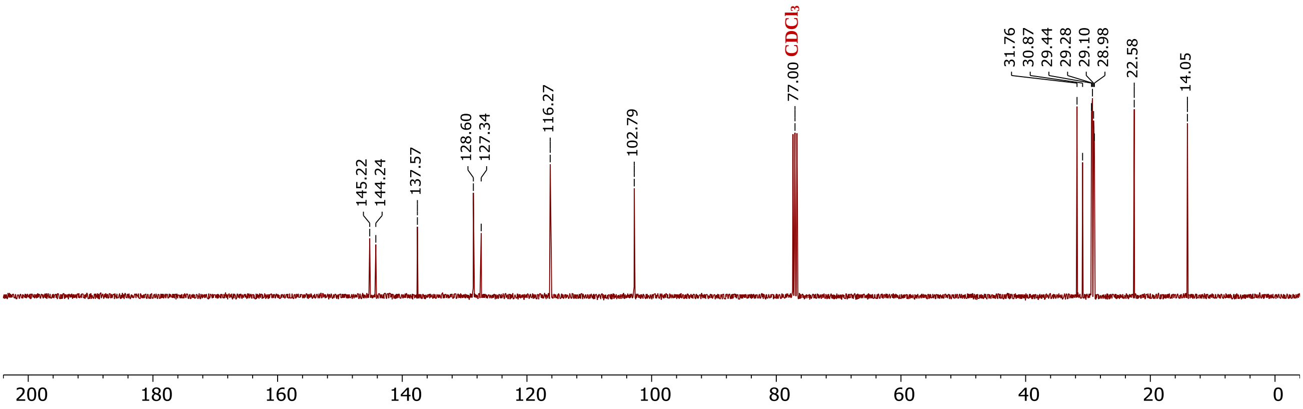

Supplement: Supplementary file 1 — ol1c03920_si_001.pdf [file ol1c03920_si_001.pdf]
